# Size‐Controlled Hapticity Switching in [Ln(C_9_H_9_)(C_8_H_8_)] Sandwiches

**DOI:** 10.1002/chem.202101599

**Published:** 2021-08-21

**Authors:** Maxime Tricoire, Luca Münzfeld, Jules Moutet, Nolwenn Mahieu, Léo La Droitte, Eufemio Moreno‐Pineda, Frédéric Gendron, Jeremy D. Hilgar, Jeffrey D. Rinehart, Mario Ruben, Boris Le Guennic, Olivier Cador, Peter W. Roesky, Grégory Nocton

**Affiliations:** ^1^ Laboratoire de Chimie Moléculaire (LCM) CNRS, Ecole polytechnique, Institut Polytechnique de Paris Route de Saclay 91120 Palaiseau France; ^2^ Institute of Inorganic Chemistry Karlsruhe Institute of Technology (KIT) Engesserstraße 15 76131 Karlsruhe Germany; ^3^ ISCR (Institut des Sciences Chimiques de Rennes)-UMR 6226 Université de Rennes, CNRS 35000 Rennes France; ^4^ Institute of Nanotechnology (INT) Karlsruhe Institute of Technology (KIT) Hermann-von-Helmholtz-Platz 1 76344 Eggenstein-Leopoldshafen Germany; ^5^ Depto. de Química-Física, Escuela de Química Facultad de Ciencias Naturales, Exactas y Tecnología, Universidad de Panamá Panam; ^6^ Panamanian National System of Investigators (SNI, SENACYT) Panama; ^7^ Department of Chemistry and Biochemistry University of California-San Diego La Jolla CA United-States; ^8^ Centre Européen de Science Quantique (CESQ), Institut de Science et d'Ingénierie Supramoléculaires (ISIS) Université de Strasbourg 8, Allée Gaspard Monge F-67000 Strasbourg France; ^9^ Institute of Quantum Materials and Technology (IQMT) Karlsruhe Institute of Technology (KIT) Hermann-von-Helmholtz-Platz 1 76344 Eggenstein-Leopoldshafen Germany

**Keywords:** cyclononatetraenyl, lanthanides, organometallics, magnetism, single molecule magnets

## Abstract

Sandwich complexes of lanthanides have recently attracted a considerable amount of interest due to their applications as Single Molecule Magnet (SMM). Herein, a comprehensive series of heteroleptic lanthanide sandwich complexes ligated by the cyclononatetraenyl (Cnt) and the cyclooctatetraenyl (Cot) ligand [Ln(Cot)(Cnt)] (Ln=Tb, Dy, Er, Ho, Yb, and Lu) is reported. The coordination behavior of the Cnt ligand has been investigated along the series and shows different coordination patterns in the solid‐state depending on the size of the corresponding lanthanide ion without altering its overall anisotropy. Besides the characterization in the solid state by single‐crystal X‐ray diffraction and in solution by ^1^H NMR, static magnetic studies and ab initio computational studies were performed.

## Graphical abstract

**A series of trivalent** lanthanide heteroleptic sandwich complexes with the cyclooctatetraenyl (Cot) and cyclononatetraenyl (Cnt) ligands shows a significant bending of the Cnt ligand when the size of the ion decreases, implying a size‐controlled hapticity switch.

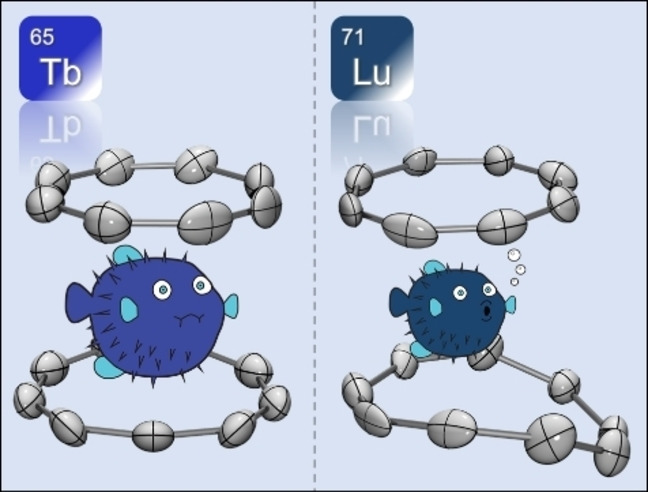

## Introduction

Many different applications of rare‐earth‐based compounds originate from their unique physical properties:^[1]^ strong spin‐orbit coupling, large magnetization and core 4 f‐orbitals.^[2]^ The specific design of compounds adapted to specific applications is facilitated by the small orbital contributions of the ligand‐field. This allows the prediction of fundamental physical properties with qualitative electrostatic models.^[3]^ These considerations have motivated a large number of fundamental structure/properties studies, especially for luminescent materials,^[4]^ MRI contrast agents^[5]^ and more recently for the design of high‐performance Single Molecule‐Magnets (SMMs).[^1a^, ^6^]

In this particular area, the gap from liquid helium (2 K) to liquid nitrogen (77 K) temperatures has recently been closed, and organometallic compounds of lanthanides have played a crucial role in this breakthrough.^[7]^ The possible geometries allowed by typical anionic cyclo‐aromatic ligands used in organometallic chemistry, such as cyclopentadienyl (Cp),^[6]^ cyclooctatetraenyl (Cot)^[8]^ and cyclononatetraenyl (Cnt),^[9]^ led to unusual arrangements, which can be tuned by the bulk of their substituents. When a linear geometry with localized charge density in the axial position is necessary to get interesting magnetic properties, as in the case of oblate ions such as Dy, small Cp ligands with large bulk are used to provide the localized charge and enforce a nearly linear geometry.[^7a^, ^7b^, ^7c^, ^10^] With prolate ions, such as Er, large ligands, such as the Cot dianion and the Cnt anion, are better suited to enhance equatorial ligand field contributions in sandwiched compounds.[^8b^, ^11^]

However, some of these specificities can be antagonistic, making the rational design of such compounds difficult resulting in a narrow edge, at which all the desired properties are maximized. Typically, a bulk increase of the Cp ligand favors linearity while driving away the point charge of the ligand from the metal ion.[^7c^, ^12^] It is thus extremely important to rationalize the dynamic coordination properties of these aromatic ligands in order to be able to control the metal‐ligand pair anisotropy.^[11c]^ Additionally, because magnetism and luminescent applications often require solid‐state measurements, the understanding of how packing forces influence the inner coordination as well as the exact arrangement of the closest neighbors in the crystal lattice is important. Recent studies led by some of us have underlined the importance of the orientation of the anisotropy axes in a series of binuclear Er(Cot) fragments^[11d]^ as well as the second sphere environment in divalent [Tm(Cot)_2_]^2−^ compounds, when designing performing SMMs.^[11e]^

**Scheme 1:**
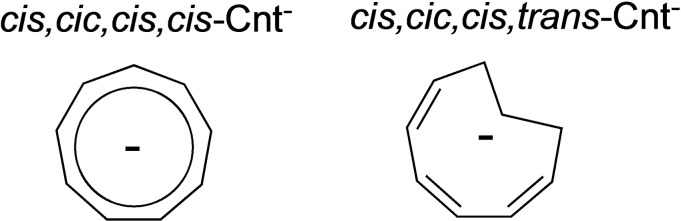
Isomers of the Cnt ligand

Among others, the cyclononatetraenyl ligand (Cnt) is a promising ligand for the rational design of SMMs: it allows the formation of perfectly linear homoleptic sandwiches with divalent lanthanides and exhibits labile coordination dynamics in coordinating solvents,^[9a]^ contrary to Cot and Cp ligands. In this work, the bis‐Cnt [Ln(Cnt)_2_] divalent complexes of Ln=Sm, Eu, Tm and Yb have been obtained in good yields through the use of a mixture of two isomers of the Cnt potassium salt: the *cis‐cis‐cis‐cis* and the *cis‐cis‐cis‐trans* forms (Scheme 1). A step further was the use of this mono‐anionic ligand in combination with the dianionic Cot ligand to form neutral sandwiches with linear geometry.^[13]^ In this series, the geometry is particularly well adapted for maximizing the anisotropy of the erbium complex and, indeed, the latter was reported to present interesting SMM properties, confirming the adapted Er‐Cnt/Cot pair anisotropy. Yet, the specific coordination of the Cnt ligand in various environments, with various bulk, and different lanthanide size remains to be studied to enlarge the scope of Cnt compounds with promising applications.

In the present work, a series of heteroleptic sandwich complexes, [Ln(Cot)(Cnt)], is presented with Ln=Tb, Dy, Ho, Er, Tm, Lu with a focus on their structural aspects, both in the solid‐state and in solution. This work reinforces the versatility of the Cnt ligand as a useful candidate for the design of specific geometries needed in organolanthanide chemistry for a given application including magnetism.

## Results and Discussion

### Synthesis

**Scheme 2:**
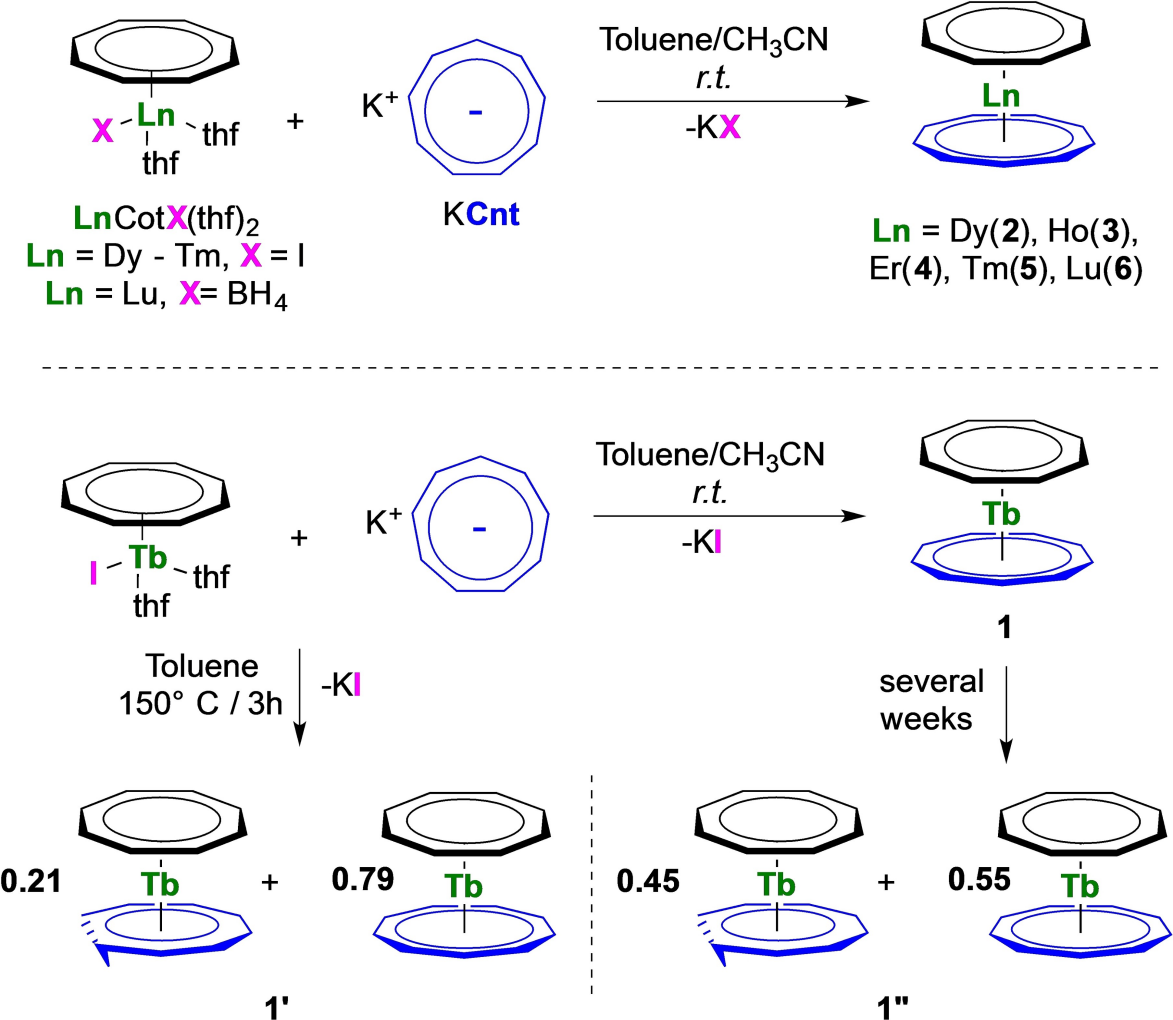
Synthesis of **1**–**6**.

The heteroleptic trivalent late lanthanide sandwich complexes [Ln(Cot)(Cnt)] (Ln=Tb (**1**), Dy (**2**), Ho (**3**), Er (**4**), Yb (**5**), Lu (**6**)) were synthesized from a toluene/acetonitrile mixture of KCnt and the corresponding [Ln(Cot)I(thf)_2_] complexes (Ln=Tb, Dy,^[13]^ Ho, Er,[^11c^, ^13^] Tm) at room temperature (Scheme 2). Due to the facile reduction of Yb^3+^ in the presence of the Cot ligand, the Yb compound is not accessible.^[14]^ [Ln(Cot)I(thf)_2_] were prepared from K_2_Cot and lanthanide tris‐iodides in thf,^[11c]^ a faster alternative compared to the elegant procedure proposed by Mashima et al. for the earlier lanthanides.^[15]^ The Lu complex is best prepared from the borohydride [Lu(BH_4_)_3_(thf)_3_] precursor^[16]^ to afford either the monomeric [Lu(Cot)(BH_4_)(thf)_2_] or the dimeric [Lu(Cot)(BH_4_)(thf)]_2_ complex as original compounds depending on the crystallization conditions, similar to the procedure of Ephritikhine for the synthesis of the Nd analogues.^[17]^ The three new crystal structures with Ln=Tb, Ho and Lu are discussed in the Supporting Information. The Tm structure was reported by Schumann et al. but was made from direct reaction of TmI_2_ with free Cot.^[18]^ In the case of Tb, Dy, Ho, Er, and Tm, the potassium salt of the Cnt ligand (both all‐*cis* and *cis‐cis‐cis‐trans* isomers can be used)[^9a^, ^19^] is best dissolved in acetonitrile before use. After 12 h of stirring, the suspensions turned to pale‐colored solutions with a gradual color change along the lanthanide series (pale yellow for Tb, yellow for Dy,^[13]^ pale orange for Ho, orange for Er,^[13]^ salmon orange for Tm). The reaction time of 12 h is necessary to complete the isomerization when the more soluble *cis‐cis‐cis‐trans* isomer is used. Synthesis of the lutetium complex [Lu(Cot)(Cnt)] (**6**) was best performed by heating KCnt and [Lu(Cot)(BH_4_)(thf)_2_] in toluene under reflux for 16 h. Another important step is the removal of all the volatiles and thorough drying of the resulting solid. Several steps of drying and re‐suspension in toluene are necessary to ensure complete de‐coordination of the acetonitrile (or remaining thf molecule in the case of lutetium). If this step is not complete, an acetonitrile adduct of [Ln(Cot)(Cnt)] may be isolated, in which the Cnt ligand binds in lower hapticity than η^9^. A crystal structure of such a dysprosium complex has been obtained with partial coordination of acetonitrile. The structure is discussed in the Supporting Information (Figure S19–S20). Once the coordinated solvent has been removed, extraction in a large amount of toluene followed by filtration, concentration and cooling (−40 °C) allows the crystallization of the title compounds (**1**–**6**) as crystals suitable for X‐ray diffraction. The erbium and dysprosium complexes, **2** and **4**, respectively, were already recently published using a different procedure and analyzed by multiple approaches. The η^9^‐Cnt coordination remained an open question because of a crystallographic disorder.^[13]^ For the Tb complex (**1**), several different crystals (crystallized at room temperature and grown over a week) of red color have been analyzed to reveal a mixture of the *cis‐cis‐cis‐cis* and of the *cis‐cis‐cis‐trans* Cnt ligand in a 79 : 21 ratio (**1′**). The structure is discussed in Supporting Information. However, when the terbium complex was prepared by an alternative method from hot toluene, the crystallized structure was similar to that of **1′** with a ratio of *cis‐cis‐cis‐cis:cis‐cis‐cis‐trans* ligand of 55 : 45 (**1′′**). Upon heating, all compounds degraded at higher temperature.

### Solution analysis

**Figure 1:**
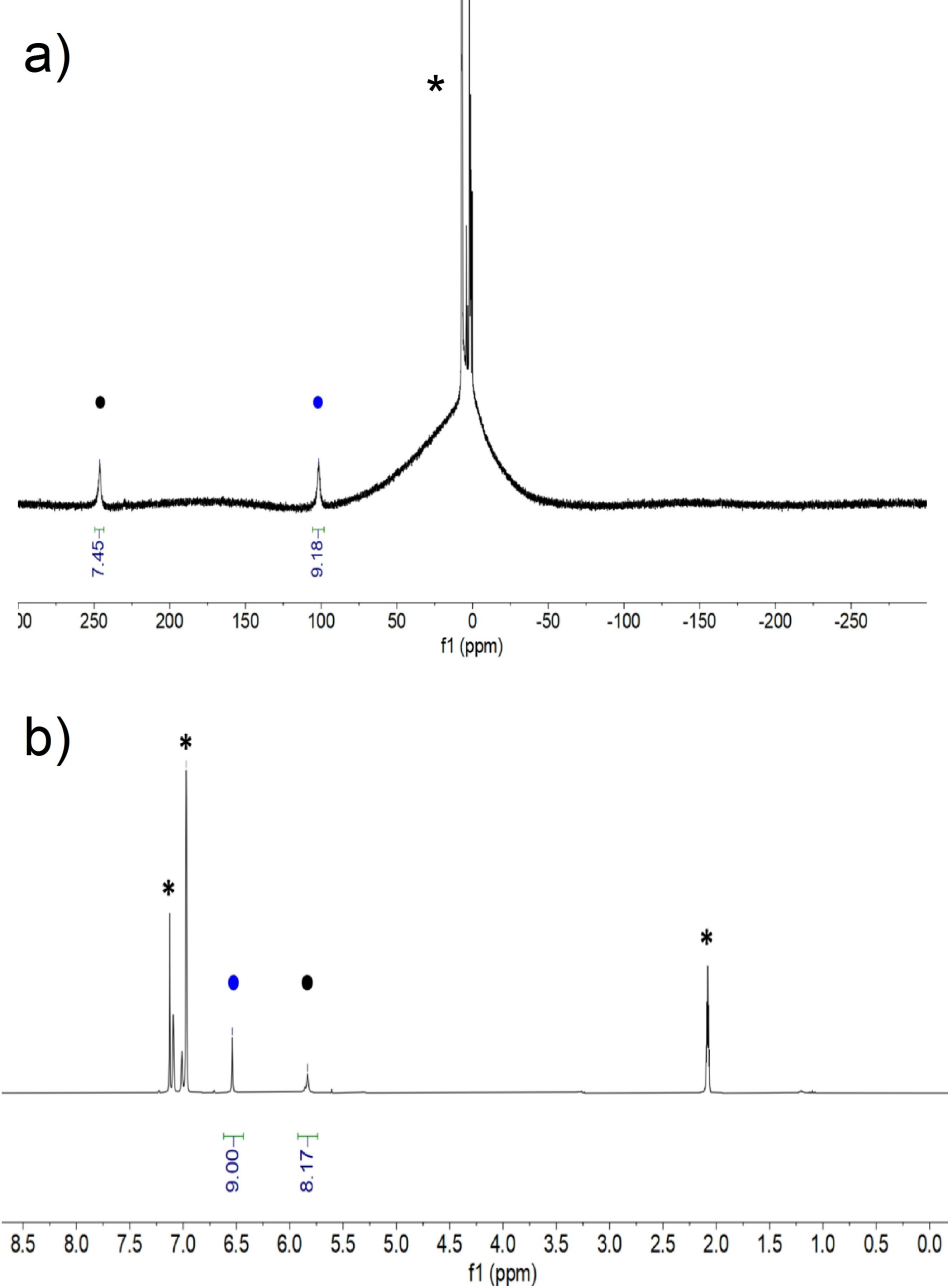
^1^H NMR of **1** (a) and **6** (b) at 293 K in toluene‐d_8_. Blue mark (•) is for the signals of the Cnt ligand and the black mark (•) for the Cot ligand. * residual protio signal of the solvent.

Compounds **1**–**6** have been analyzed by ^1^H NMR spectroscopy. Except for the Lu compound (**6**), which is diamagnetic, **1**–**5** are highly paramagnetic but signals have been obtained for all compounds (Figure 1, Supporting Information, Figure S1‐S14). The broad signals obtained for the Er (**4**) and Tm (**5**) complexes did not allow distinguishing the Cnt from the Cot ligand on the basis of their relative integrations. The signals appear at −5.01, −128.7 ppm and −23.2 and −235.8 ppm, for **4** and **5**, respectively, at room temperature. For **1–3** and **6**, ^1^H NMR spectra feature two similar signals with chemical shifts of 245.9, 118.7, 90.5 and 5.83 ppm for the Cot, respectively, and of 101.5, 72.9, 59.2, and 6.54 ppm for the Cnt, respectively. In **1–3**, the shapes of the f‐electron density in the lanthanide ions are oblate, while in **4**–**5**, they are prolate.^[3b]^ The modification in the anisotropy orientation between **3** and **4** is well reflected by the sign change of their chemical shifts for both ligands. Variable temperature ^1^H NMR was performed for **6** but no fluxional behavior^[20]^ for both ligands was observed in the range from +80 to −80 °C.

**Scheme 3:**
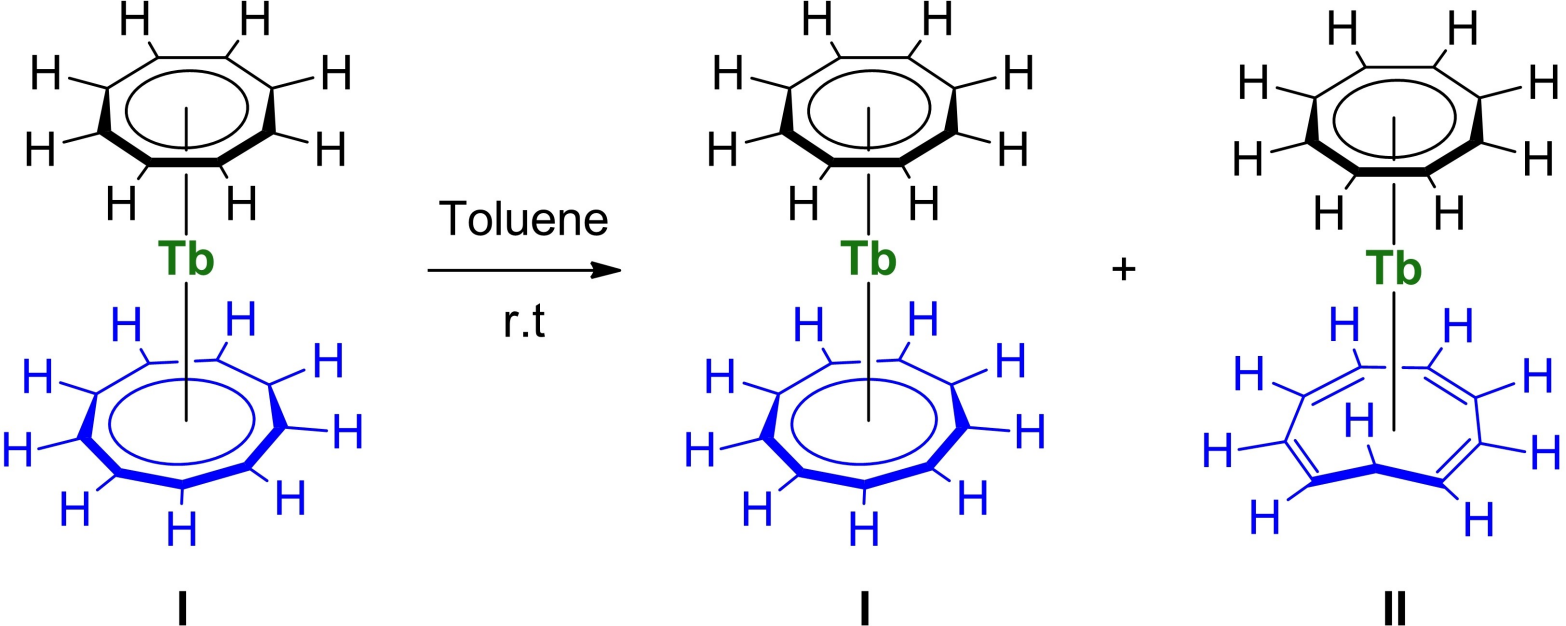
Isomerization of I to II in solution.

The crystallization of different terbium complexes, **1**, **1′**, and **1′′** with a different configuration for the Cnt ligand was intriguing to us. The free Cnt^−^ ligand indeed possesses two different isomers, easily quantified by ^1^H NMR spectroscopy: the *cis‐cis‐cis‐cis* ligand gives rise to one singlet in agreement with D_9h_ symmetry while the *cis‐cis‐cis‐trans* one features 5 signals in agreement with C_2v_ symmetry.^[21]^ In a previous work, the use of a soluble mixture of these two isomers leads to multiple Ln(Cnt)_2_ sandwich isomers, which were also easily identified by ^1^H NMR spectroscopy.^[9a]^ Additionally, Boche et al. gathered, in a comprehensive series of three articles, useful information on the Cnt ligand synthesis, as well as its topomerization and isomerization.[^19^, ^22^] These studies unveil several questions on the real nature of the isomerization process when assisted by metal complexes. An interesting connection to this is that **1′′** in which one ligand is partially isomerized in the *cis‐cis‐cis‐trans* form, is obtained in a relatively high‐temperature synthesis (150 °C for 3 h) from the *cis‐cis‐cis‐cis* ligand, supposedly thermodynamically more stable. In contrast, **1** only contains the *cis‐cis‐cis‐cis* ligand and is prepared under milder conditions (room temperature synthesis). Thus, in order to probe a possible temperature effect on the isomerization of the ligand in the Tb complex, a solution of **1** in toluene‐d_8_ was let stand for several days at room temperature. The resulting ^1^H NMR spectra of this experiment are shown in Figure S14. A new set of 6 signals increases with time at δ 404.0, 230.1, 203.6, 196.2, 165.1 and −169.5 ppm while the two initial signals of **1** (Scheme 3, **I**) were still present. The estimated ratio based on integration, 2 : 8 : 2 : 2 : 1 : 2, needs to be considered cautiously because of the highly paramagnetic nature of the complex. Yet, it is in full agreement with the formation of a [Tb(Cot)(Cnt)] isomer in which the Cnt ligand is in the *cis‐cis‐cis‐trans* configuration (Scheme 3, **II**). Indeed, the isomerized Cnt ligand in **1′** and **1′′** gives rise to 5 distinct proton resonances, as also observed in the free cis‐cis‐cis‐trans Cnt ligand,^[9a]^ while the Cot signal remains a singlet integrating for 8 protons. The ratio between the isomers **I** and **II** is ca. 66 : 34 after three days and stabilizes over time to approximately 60 : 40 after 20 days, remarkably close to the ratio found in the solid state structure of **1′′** (55 : 45), while **1′**, which was obtained from a slow evolution of **1** at room temperature features a higher **I**:**II** ratio of 79 : 21.

This unexpected situation again connects to Boche′s findings and shows that the nature of the coordinated metal greatly affects the inter‐conversion between topologic isomers of the Cnt ligand. Although this isomerization has only been observed so far in case of the Tb complex **1**, a thorough investigation of the Cnt isomerization within the [Ln(Cot)(Cnt)] series (which is outside the scope of this article) would still be necessary to understand whether it is related to size effects or other physical properties.

### X‐ray solid‐state crystal structure

**Figure 2:**
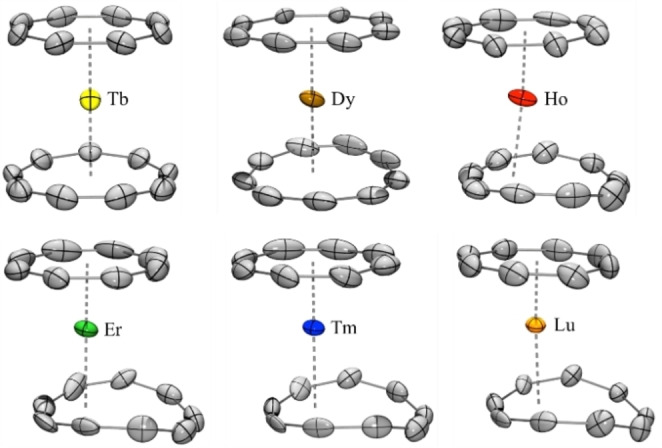
ORTEP of **1**–**6**. Thermal ellipsoids are depicted at 50 % probability level. Hydrogen atoms and the disorder are omitted for clarity (see main text for the discussion of the coordination mode).

The crystal structures are isomorphous from Dy to Lu (**2**–**6**) and were solved in *P*2_*1*_ */n* irrespective of the data collection temperatures (Figure 2). There is a positional disorder in both ligands, the first one featuring eight carbon atoms (Cot), the second one nine carbon atoms (Cnt). Within this space group, the presence of these specificities creates a situation in which the final solution has two rings embedded in each other with occupancy of 0.5 on each atom (See Figure S25a). Additionally, for the complexes **2**–**6**, the lanthanide ion is not placed at a special position (i. e. sitting on a symmetry element) but lies close to an inversion center, also with occupancy of 0.5. As a result, the lanthanide ion is not centered with the ligands but is slightly moved away, which seems to point out a possible lower coordination mode (hapticity) of one of the ligands. This situation also drastically complicates the resolution when one wants to separate the ligands from one another since the symmetry generation by the *P*2_*1*_ */n* space group creates two more rings embedded with each other along with an additional lanthanide atom close to the inversion center (See Figure S25b). However, because of the symmetry rules, only two variants exist and it is not possible to mix the two different configurations.

Thus, from a crystallographic point of view, two solutions are possible; *viz*. a lower hapticity either for the Cot or for the Cnt ligand (See Figure S25d, e). However, it seems that the deviation from planarity observed in the 9‐membered ring is in better agreement with a perturbed coordination mode for the Cnt ligand rather than for the Cot ligand. Additionally, the Ln−Ctr (Ctr, centroid) distance with both rings must respect the increased negative charge of Cot^2−^ with respect to Cnt^−^ and longer Ln−Cnt distances are therefore expected.^[13]^ If the η^9^‐Cnt coordination mode was preferred, both the Ln−Ctr distances and the coordination mode of Cot are unrealistic from a chemical point of view for the lutetium compound (**6**) (See Supporting Information). Lowering the symmetry to the *P*1 symmetry group (See Figure S26) increases the size of the asymmetric unit but does not allow to better define the disorder; it is still necessary to assume either longer Ln−Ctr distances for the Cnt‐ or for the Cot‐ ligand. When solving the data, it is also necessary to allow positional freedom to the carbon atoms except for the aromaticity restraint on the C−C distances on both rings in order to avoid scrambling of the carbon atoms between the two ligands moieties upon refinement. If the ring having eight carbon atoms (Cot) ends up relatively planar, in agreement with most Cot ligands coordinated to lanthanide ions in the literature, the one with nine carbon atoms (Cnt) shows a strong deviation from planarity with a curvature at the extremity, which tends to be indicative of a different coordination mode. In **2**–**6**, a closer look to the Ln−C set of distances within the eight values of the eight‐membered ring (See Table 1) shows a relatively close set of distances in the range 2.44(2)‐2.50(2) Å in **6**, 2.43(3)‐2.54(3) Å in **5**, 2.45(2)‐2.55(3) Å in **4**, 2.46(2)‐2.65(2) Å in **3**, and 2.52(2)‐2.64(2) Å in **2**. In contrast, the variation in the nine membered Cnt ring is much more pronounced (Table 1) with η(Ln−C(Cnt)_max_‐LnC(Cnt)_min_ values of 1.426 Å in Lu (**6**) (2.50(2) to 3.930(9) Å), 1.228 Å in Tm (**5**) (2.54(3) to 3.769(8) Å), 1.213 Å in Er (**4**) (2.55(3) to 3.763(15) Å), 0.906 Å in Ho (**3**) (2.56(2) to3.47(2) Å), and 0.284 Å in Dy (**2**) (2.66(2) to 2.96(2) Å).

**Table 1:** Main metric parameters for **1**–**6** at 150 K. [a] Only the carbon atoms formally η‐coordinated are considered in the average calculation.

	Tb (**1**, η^9^)	Dy (**2**, η^8^)	Ho (**3**, η^6^)	Er (**4**, η^6^)	Tm (**5**, η^6^)	Lu (**6**, η^6^)
Ln−C(Cnt)	2.775(7)	2.68(2)	2.56(2)	2.55(3)	2.54(3)	2.50(2)
	2.775(7)	2.72(2)	2.61(2)	2.59(3)	2.57(2)	2.54(2)
	2.79(1)	2.74(2)	2.62(2)	2.61(3)	2.64(2)	2.6(2)
	2.82(3)	2.74(2)	2.72(2)	2.69(3)	2.73(2)	2.68(2)
	2.83(1)	2.79(2)	2.82(2)	2.88()	2.875(13)	2.943(12)
	2.84(2)	2.86(2)	3.02(3)	3.10(2)	3.11(2)	3.14(2)
	2.85(3)	2.86(2)	3.212(14)	3.443(13)	3.449(9)	3.59(10)
	2.85(1)	2.95(2)	3.37(2)	3.60()	3.594(12)	3.719(13)
	2.86(2)	2.96(2)	3.47(2)	3.76 (2)	3.769(8)	3.930(9)
η(Ln−C(Cnt)_max_‐LnC(Cnt)_min_)	0.094	0.284	0.906	1.213	1.228	1.426
Ln−C(η‐Cnt)^[a]^ ave	2.82(3)	2.79(9)	2.73(17)	2.74(21)	2.74(21)	2.73(26)
C(η‐Cnt)‐Ln−C(η‐Cnt)^[a]^	**177.4**	**172.0**	**169.6**	**174.7**	**173.8**	**174.2**
Ln−C(Cnt) ave	**2.82(3)**	**2.81(10)**	**2.93(33)**	**3.02(44)**	**3.03(44)**	**3.07(55)**
Ln−C(Cot) range	2.57(3)‐2.63(2)	2.52(2)‐2.64(2)	2.46(2)‐2.65(2)	2.45(2)‐2.55(3)	2.43(3)‐2.54(3)	2.44(2)‐2.50(2)
Ln−C(Cot) ave	**2.58(2)**	**2.58(4)**	**2.55(7)**	**2.50(4)**	**2.48(3)**	**2.46(1)**
Ln−C(η‐all)^[a]^ ave	**2.71**	**2.69**	**2.63**	**2.60**	**2.59**	**2.58**

The space group of the Tb complex (**1**) is modified from *P*2_*1*_ */n* to *Pnma* with the removal of the inversion center on the lanthanide atom, which was confirmed by the analysis of the precession images. An additional symmetry plane that contains the centroids of both the Cot and Cnt ligands and the Tb metal center is present. The latter thus separates both ligands in two symmetrical sections. The differentiation in the space group between the late lanthanide ions (**2**–**6**) and the Tb one (**1**) could be explained by a hapticity switching of the Cnt ligand in the case of larger lanthanide ions, imposing a different symmetry than that observed in **1**. Consequently, the structure visually appears to be in better agreement with a formal η^9^‐coordination mode. A lower variation in the Ln−C(Cnt) distance range (η(Ln−C(Cnt)_max_‐LnC(Cnt)_min_=0.094 Å) (See Table 1) is observed. The deformation of the Cnt ligand is minimal.

**Figure 3:**
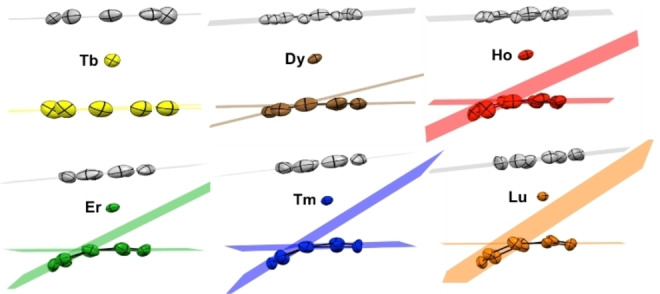
Plane constructions in **1**–**6**. The grey plane is constructed with the eight carbon atoms of the Cot ligand. The colored planes are constructed with the six closest carbon atoms and the three remaining carbons of the Cnt ligand, respectively (see main text for limits of this construction)

Several metric parameters help to visualize and quantify the structural modifications (curved vs. planar) and the hapticity modulation of the Cnt ligand in **1**–**6**. First, three main planes were constructed; the plane defined by the Cot ligand (in grey, Figure 3), the mean plane made by the six Cnt carbon atoms that are the closest to the lanthanide ion (in color) and the one set up by three remaining Cnt carbon atoms (in color), which are located further away from the lanthanide ion. The angle between the two Cnt planes decreases from the Lu to the Tb complexes in agreement with a stronger deviation from planarity with smaller lanthanide ions. At low‐temperature, the angle is decreasing gradually from 34.2° in Lu (**6**) to 4.80° in Tb (**1**), with intermediate values of 31.3°, 29.7° 23.7°, and 12.4° in Tm (**5**), Er (**4**), Ho (**3**) and Dy (**2**), respectively (Table S8). At room temperature, the decrease is weaker from 25.3° to 14.8° from **6** to **2**. Complex **5** has also been recorded at 100 K, 150 K, 200 K, and 250 K with no clear break in the metric parameters.

The plane angle difference between the low‐ and room‐temperature X‐ray structures is also significant. This set of angular parameters intends to show how the curvature of the Cnt ligand gradually evolves from Tb to Lu in agreement with the lanthanide contraction of the ionic radius.^[23]^

The plane angle difference between the low‐ and room‐temperature X‐ray structures is also significant. This set of angular parameters intends to show how the curvature of the Cnt ligand gradually evolves from Tb to Lu in agreement with the lanthanide contraction of the ionic radius.^[23]^

A second set of useful metric parameters corresponds to the distances and angles from the metal ion to the constructed centroids of both ligands (See Table S8). While the centroid of the Cot ligand is always defined by its eight carbons atoms, three different centroids were constructed for the Cnt ligand: the first with the eight carbon atoms of the Cot ligand and the three others with i) the six atoms closest to the lanthanide ion (Ctr6), ii) the eight atoms closest (Ctr8) and iii) the nine atoms of the Cnt ligand (Ctr9). The Ln−Cot(Ctr) distances (at 150 K) vary from 1.804 Å (**1**), 1.772 Å (**2**) 1.735 Å (**3**), 1.701 Å (**4**), 1.681 Å (5), to 1.653 Å (**6**). The overall trend in the distances follows the lanthanide contraction. It is rather informative to compare these distances with those in the mono‐Cot iodide or borohydride analogues [Ln(Cot)I(S)_2_] (S=thf, pyridine or CH_3_CN) of 1.814 (Tb), 1.80(1) (Dy), 1.78 (Ho), 1.763(12) (Er),^[11c]^ 1.750(5) Å (Tm)^[18]^ and [Lu(Cot)(BH_4_)(thf)]_2_ of 1.724 Å. Within this series, for Ln=Dy, Ho, Er, Tm, and Lu, the Ln−Cot(Ctr) distances are shorter than those reported for **2**–**6** while they are similar in **1**.

**Figure 4:**
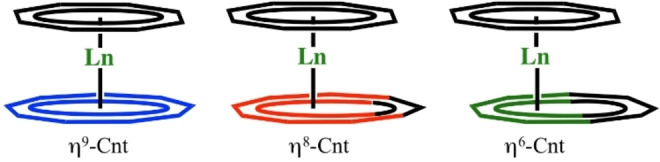
Representation of the different limit coordination modes of the Cnt ligand.

The Ln−Cnt distances are somewhat informative to distinguish three potential coordination modes (See Figure 4): at low‐temperature, for the late lanthanides (Ho to Lu), the shortest Ln−Cnt distances are that with Ctr6. In contrast, for Dy and Tb, the shortest Ln−Cnt distances are obtained with Ctr8 and Ctr9, respectively. Similarly, within all the series, the Cot(Ctr)‐Ln−Cnt(Ctr) angles are also the largest using the same aforementioned Cnt centroids (See Table S8). This set of data would best describe the hapticity of the Cnt ligand as formal η^6^ for Ho (**3**) to Lu (**6**), while the hapticity would be best described as formal η^8^ for Dy (**2**) and η^9^ for Tb (**1**). Interestingly, at higher temperature, the picture is slightly different and the coordination mode of the Cnt in Ho (**3**) would be best described as formal η^8^. The hapticity of the Cnt in the Er (**4**), Tm (**5**) and Lu (**6**) complexes would remain best described as η^6^ at room temperature. Thus, the average Ln−C distances on all formally η‐coordinated carbon atoms were calculated for **1**–**6** and are reported in Table 1. A smooth variation with the lanthanide ion size is reported from 2.71 Å for [Tb(Cot)(Cnt)] (**1**) complex to 2.58 Å for [Lu(Cot)(Cnt)] (**6**).

Additionally, it is possible to consider the sum of the van der Waals radii as additional information on the nature of the interaction, i. e. distance metrics that are larger than the sum of the van der Waals radii are not expected to size any interaction.^[24]^ The range of these van der Waals radii for Tb−Lu is 1.80 to 1.72 Å and that of aromatic C atom is 1.70 Å. Thus the maximum distance is 3.42 Å for **6** (Lu) and 3.47 Å for **1** (Tb). In these considerations **4**–**6** are η^6^, **3** is η^8^ and **1,2** are η^9^. However, these analyses should be taken cautiously because these metrics present the limit forms of coordination (η^6^ and η^9^) but the real hapticity is probably best found in between these limit forms, as found in solution (See ^1^H NMR). Moreover, from a strict crystallographic point of view, the opposite construction, i. e. η^9^‐coordination of the Cnt and lower coordination mode of the Cot ligand, remains valid.

### Magnetic measurements

**Figure 5:**
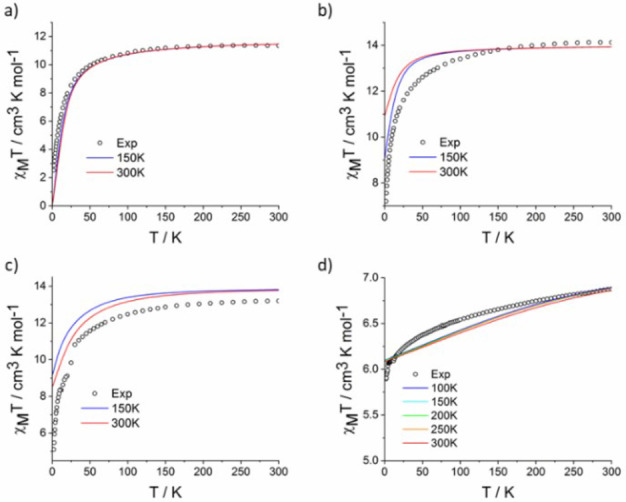
Temperature dependent χ_M_T values for compounds **1** (a), **2** (b), **3** (c) and **5** (d) in dots with the calculated curves from structures at various temperatures.

The solid‐state magnetic data for compounds **1**–**3** and **5** are reported in Figure 5. The room temperature χ_M_T values are equal to 11.34, 14.12, 13.2 and 6.8 cm^3^ K mol^−1^ for compounds **1**–**3** and **5** respectively. These values are in fairly good agreement with the calculated Curie constants for the ground state multiplets ^7^F_6_ (Tb(III), **1**, 11.82 cm^3^ K mol^−1^, g_J_=3/2), ^6^H_15/2_ (Dy(III), **2**, 14.17 cm^3^ K mol^−1^, g_J_=4/3), ^5^I_8_ (Ho(III), **3**, 14.07 cm^3^ K mol^−1^, g_J_=5/4) and ^3^H_6_ (Tm(III), **5**, 7.15 cm^3^ K mol^−1^, g_J_=7/6). On cooling, χ_M_T′s decrease monotonically down to 2 K: 2.52 cm^3^ K mol^−1^ for **1**, 7.2 cm^3^ K mol^−1^ for **2**, 5.1 cm^3^ K mol^−1^ for **3** and 5.9 cm^3^ K mol^−1^ for **5**. These values reflect the signature of the effect of the crystal field splitting that lifts the degeneracy of the ground state multiplets. The magnetization curves at 2 K are given in the Supporting Information (Figure S15). None of these compounds show slowing down of the relaxation of the magnetic moment in the absence of external dc field, which means that they do not behave as Single‐Molecule Magnets (SMM) in zero field as opposed to the reported Er complex, **4**.^[13]^

### Computational studies

Theoretical computations at the SA‐CASSCF/RASSI‐SO level have been performed for **1**–**5** based on the X‐ray crystal structures at the different temperatures at which the X‐ray data have been obtained (See Supporting Information for computational details). This series of computations can be compared to those done on the basis of the DFT optimized structure of [Er(Cot)(Cnt)] that lead to a perfectly linear structure.^[13]^ Ground and excited state wave functions for **1**–**5** along with the corresponding energy gaps are shown in Tables S30–S34.

Terbium is next to the isotropic gadolinium and is oblate. Tb is a non‐Kramers ion, and thus, the crystal field allows the formation of non‐degenerated m_J_ states. In the geometry imposed by the two large aromatic ligands, the linear geometry does not favor axial anisotropy, which shall allow mixing the m_J_ states and/or accounting for m_*J*_=0 ground state. The theoretical treatment needs to take into account this singularity and is then rendered more difficult. Thus, the ground calculated state is then the m_*J*_=0 state and just above the m_*J*_=±1 state, which is not in good agreement with the low temperature data for the above‐mentioned considerations.

However, the 2 *J*+1 states split on 873 cm^−1^ and give a reasonable agreement with the experimental data (Figure 5) at higher temperatures.

**Figure 6:**
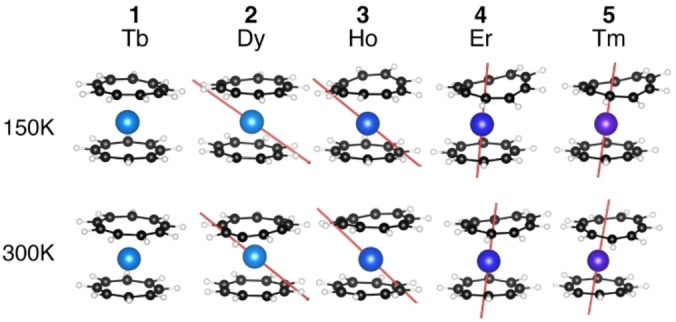
Computed structures of **1**–**5** at 150 and 300 K and ground‐state anisotropy axis (not represented for Tb due to planar anisotropy).

Following the trend described above, the oblate Dy and Ho ions have similar mixed configurations with principally m_*J*_=±15/2 (65 %) and ±11/2 (13 %) for **2** and principally m_*J*_=±8 (65) and ±6 (14 %) for **3**, both at 150 K. The m_*J*_ composition evolves at 300 K to be m_*J*_=±15/2 (83 %) and ±11/2 (12 %) for **2** and m_*J*_=±8 (56 %), ±6 (13 %), and ±7 (12 %) for **3**, explaining the different calculated low temperature magnetic susceptibility data observed (Figure S26 and S27). In **2**, six energy states are found within 109 cm^−1^, while in **3**, the overall splitting is only 397 cm^−1^ with 5 states within 136 cm^−1^. The modification of the energy state composition and splitting when different structures are used (X‐ray, 150 K, 300 K) is not greatly modulated, and thus the overall calculated magnetic temperature dependent curves is comparable with that of the experiments (Figure 5). These computations rationalize very well the magnetic behavior of **2** and **3**, showing no SMM behavior, as expected from the electrostatic model. Additionally, as expected for oblate ions with large aromatic ligands, the anisotropy orientation is not following the axial symmetry (Figure 6).

For the prolate ions, erbium and thulium, large aromatic ligands are usually well adapted for maximizing the anisotropy compared to the three ions discussed above; they disfavor the mixing of the m_*J*_ states and maximize anisotropy. Accordingly, the ground state is pure m_*J*_=±15/2 for the erbium complex (**4**), while it is pure m_*J*_=±6 for the thulium complex (**5**). For **4**, the first excited state of m_*J*_=±13/2 (98 %) and the second excited state, which is principally a mixed configuration of m_*J*_=±1/2 (68.2 %) and m_*J*_=±3/2 (19.9 %) are located at 170 and 251 cm^−1^, respectively. In **4**, the relative energy of the m_*J*_ crystal field states varies very little depending upon the structure (160 and 255 cm^−1^) chosen for the computations (X‐ray, 150 K, 300 K). However, as expected from the higher symmetry of the structure obtained from DFT optimization, the nature of the energy states differs in the fact that they are pure in symmetric structure but mixed with more realistic solid‐states structures. However, despite this, the computed variable temperature magnetic susceptibility curve is very similar to that reported experimentally in previous work.^[13]^ In **4**, the anisotropy is typically perpendicular to the aromatic sandwich ligands in agreement with the high symmetry of the complexes. In the case of the X‐ray structures, the anisotropy is not perfectly perpendicular to the Cot plane but crosses the two ligands in the approximate position of the η^8^‐centroid of the Cot ligands and the η^6^‐centroid of the Cnt ligand (Figure 3). In turn, this does not impact the magnetic properties and the magnetic barrier of 251 cm^−1^ measured in previous work. According to the computations, the barrier is therefore of good agreement with a thermally accessed QTM via the second excited state.

In **5**, the computations indicate a ground state with pure m_*J*_=±6, in agreement with the experimental low temperature χ_M_T value. The first excited state is found 431 cm^−1^ above and is pure m_*J*_=±5. The large gap between the ground and first excited state contrasts with the experimental curve shape that increases relatively fast between 2 and 50 K, which would agree with a Boltzmann population of lower excited energy states. The overall splitting is 770 cm^−1^.

## Conclusion

In conclusion, we report the synthesis and characterization of a series of heteroleptic late trivalent lanthanide complexes from Tb to Lu with the dianionic Cot and the monoanionic Cnt ligand. The terbium complex adopts a linear structure in which both ligands are aligned and their hapticity is η^8^ for the Cot and η^9^ for the Cnt ligand. However, the latter was shown to partially isomerize over time. This yields a structure, in which one of the carbon atoms of the Cnt moves inside the ring, giving a *cis*‐*cis*‐*cis*‐*trans* motif and modifying the overall hapticity of the ligand from nine to eight. The isomerization process has been tracked by ^1^H NMR spectroscopy and X‐ray crystallography. From Dy to Lu, the complexes are highly disordered, which makes the analysis more difficult. The solid‐state bond metrics show that the Cnt ligand curves with several carbon atoms being moved away from the metal center. The data follow well the ionic contraction of the lanthanide with the Lu complex being the most distorted one. In solution, in the 183–273 K temperature range, the ligand signal is single and remains flexible as observed by ^1^H NMR spectroscopy. The temperature‐dependent magnetic data show that the presence of large aromatic ligand suits the prolate ions Er and Tm particularly well. However, only the Er analogue was shown to exhibit SMM behavior.^32^ The *ab initio* wavefunction‐based computations insist on the role of the ligand geometry on the nature of the ground state and indicate that the deviation of the linearity of the Cnt ligand does not affect the anisotropy in the prolate ions significantly, while this modulates the composition and the ratio of the mixed m_*J*_ states of the non‐adapted oblate ions. Together, the data strongly indicate that the coordination versatility of the Cnt ligand might be a great tool for the modulation of the physical properties of lanthanide complexes.

## Experimental Section

**General considerations**: All reactions were performed using standard Schlenk‐line techniques or in an argon filled glovebox (MBraun). All glassware was dried at 140 °C for at least 12 h prior to use. THF, DME, toluene and toluene‐d_8_ were dried over sodium, degassed and transferred under reduced pressure in a cold flask. TmI_3,_ ErI_3_, HoI_3_, DyI_3_ and TbI_3_ were purchased from Sigma Aldrich and used without further purification. NMR spectra were recorded in 5 mm tubes adapted with a J. Young valve on Bruker 300 MHz Avance III spectrometers. Chemical shifts are expressed relative to TMS in ppm. Infrared (IR) spectra were recorded at room temperature under argon on a Thermo Scientific Nicolet iS5 spectrometer equipped with the iD7 ATR‐Diamond unit. Magnetic measurements were obtained in a Quantum Design MPMS‐XL SQUID magnetometer. An applied magnetic field of 2 kOe is used in the temperature range 2–20 K, and 10 kOe for temperatures above 20 K, for the χ_M_T vs. T curves. To avoid reorientation and sample degradation the microcrystals are trapped and slightly pressed between quartz wool and sealed in quartz tubes or were fixated with dried and degassed eicosane in flame sealed quartz tubes.

**Crystal‐structure analysis**: Deposition Numbers 2073511 (for [Tb(Cot)I(thf)_2_]), 2073512 (for [Ho(Cot)I(thf)_2_], 2073513 (for [Lu(Cot)(BH_4_)(THF)]_2_), 2073514 (for **1** at 150 K), 2073515 (for **1** at 300 K), 2073516 (for **2** at 150 K), 2073517 (for **2** at 300 K), 2073518 (for **3** at 150 K), 2073519 (for **3** at 300 K), 2073520 (for **4** at 150 K), 2073521 (for **4** at 300 K), 2073522 (for **5** at 150 K), 2073523 (for **5** at 300 K), 2073524 (for **6** at 150 K), 20735325 (for **6** at 300 K), 2073526 (for **1′** at 150 K), 2073527 (for **1′′** at 150 K), 2073528 (for **5** at 100 K), 2073529 (for **5** at 200 K), 2073530 (for **5** at 250 K), 2073531 (for **2 b** at 150 K) contain the supplementary crystallographic data for this paper. These data are provided free of charge by the joint Cambridge Crystallographic Data Centre and Fachinformationszentrum Karlsruhe Access Structures service.

### Syntheses

**[Er(Cot)I(thf)]**: The complex was synthesized as previously described.^[11c]^ A similar procedure was used for the other late lanthanides. [Lu(BH_4_)_3_(thf)_3_] was synthesized using the procedure reported for the Nd analogue.^[17]^

**[Tb(Cot)I(thf)]**: A cold thf solution (‐40 °C) of K_2_Cot (4 mL, 117 mg, 0.64 mmol, 1 equiv) was added to a stirred cold (−40 °C) thf suspension of TbI_3_ (3 mL, 346 mg, 0.64 mmol, 1 equiv). The mixture was allowed to warm to room temperature (r.t.*)* and was stirred for 16 h. The resultant light yellow suspension was filtered through a frit and the filtrate dried under reduced pressure. The solids were suspended in 5 mL thf and heated to 60 °C forming a deep yellow solution. Diethyl ether was layered on the top and the solution was stored at −40 °C. Yellow needles of the desired compound formed slowly (181 mg, 53 %). ^1^H NMR (300 MHz, thf‐d_8_, 293 K): δ (ppm), 194.92 (s br, 8H), *coordinated thf molecules are not visible*. IR (ATR): ṽ=2970 (br m), 2889 (m), 1859 (w), 1753 (w), 1616 (w), 1556 (w), 1444 (br m), 1343 (w), 1309 (w), 1245 (w), 1180 (w), 1011 (vs), 895 (s), 860 (vs), 775 (m), 749 (m), 708 (vs), 667 (s), 573 (m) cm^−1^. Anal. Calcd. for C_16_H_24_IO_2_Tb (534.19): C, 35.97; H, 4.53; Found: C, 35.77; H, 4.56.

**[Dy(Cot)I(thf)]**: A cold thf solution (‐40 °C) of K_2_Cot (5 mL, 138 mg, 0.76 mmol, 1 equiv) was added to a stirred cold (−40 °C) thf suspension of DyI_3_ (10 mL, 411 mg, 0.76 mmol, 1 equiv). The mixture was allowed to warm to r.t. and was stirred for 16 h. The resultant light yellow suspension was filtered through a frit and the filtrate dried under reduced pressure. The solids were suspended in 8 mL thf and heated to 60 °C forming a deep yellow solution. Diethyl ether was layered on the top and the solution was stored at −40 °C. Yellow needles of desired compound formed slowly (265 mg, 63 %). ^**1**^H NMR (300 MHz, thf‐d_8_, 293 K): δ (ppm), 88.66 (s br, 8H), *coordinated thf molecules are not visible*. IR (ATR): ṽ=2951 (br m), 2889 (m), 1858 (w), 1752 (w), 1614 (w), 1554 (w), 1453 (br m), 1343 (w); 1309 (w), 1256 (w), 1178 (w), 1010 (vs), 895 (s), 860 (vs), 750 (m), 705 (vs), 670 (s), 575 (m) cm^−1^. Anal. Calcd. for C_15.4_H_19.2_IO_2_Dy (537.77): C, 33.03; H, 3.91; Found: C, 32.94; H, 4.07. The number of thf was decreased upon drying.

**[Ho(Cot)I(thf)]**: A cold thf solution (‐40 °C) of K_2_Cot (5 mL, 223 mg, 1.22 mmol, 1 equiv) was added to a stirred cold (−40 °C) thf suspension of HoI_3_ (668 mg, 1.22 mmol, 1 equiv). The mixture was allowed to warm to r.t. and was stirred for 16 h. The resultant light yellow suspension was filtered through a frit and the filtrate dried under reduced pressure. The solids were suspended in 7 mL thf and heated to 60 °C forming a deep yellow solution. Diethyl ether was layered on the top and the solution was stored at −40 °C. Yellow needles of desired compound formed slowly (583 mg, 88 %). ^1^H NMR (300 MHz, thf‐d_8_, 293 K): δ (ppm), 70.88 (s br, 8H), *coordinated thf molecules are not visible*. IR (ATR): ṽ=2969 (br m), 2888 (m), 1852 (w), 1747 (w), 1601 (w), 1441 (br m), 1343 (w), 1212 (br w), 1008 (vs), 856 (vs), 750 (m), 704 (vs), 645 (s) cm^−1^. Anal. Calcd. for C_16_H_24_IO_2_Ho (540.20): C, 35.57; H, 4.48; Found: C, 35.63; H, 4.53.

**[Tm(Cot)I(thf)]**: A cold solution (−40 °C) of K_2_Cot (3 mL, 32 mg, 0.17 mmol, 1 equiv) was added to a stirred cold (−40 °C) thf suspension of TmI_3_ (5 mL, 95 mg, 0.17 mmol, 1 equiv). The mixture was allowed to warm to r.t. and was stirred for 16 h. The resultant light yellow suspension was filtered through a frit and the filtrate dried under reduced pressure. The solids were suspended in 7 mL thf and heated to 60 °C forming a deep yellow solution. Diethyl ether was layered on the top and the solution was stored at −40 °C. Yellow needles of desired compound formed slowly (54 mg, 57 %). The ^1^H NMR (300 MHz, thf‐d_8_, 293 K) remained silent. The compound was previously published by Fedushkin et al.^[18]^

**[Lu(Cot)(BH_4_)(thf)]** : A cold solution (‐40 °C) of K_2_Cot (61 mg, 0.33 mmol, 1 equiv) was added to a stirred cold (−40 °C) thf solution of [Lu(BH_4_)_3_(thf)_3_] (145 mg, 0.33 mmol, 1 equiv). The mixture was allowed to warm to r.t. and was stirred for 16 h. The resultant light yellow suspension was filtered through a teflon syringe filter. The filtrate was concentrated under reduced pressure until incipient crystallization and stored at −40 °C to afford the title compound as light yellow crystals (117 mg, 0.27 mmol, 81 %). Recrystallization from toluene led to the decoordination of one thf molecule and formation of [(Cot)Lu(BH_4_)(thf)]_2_ as colorless crystals suitable for X‐ray diffraction studies. ^1^H NMR (300 MHz, thf‐d_8_, 293 K): δ (ppm), 6.31 (s, 8H, Cot), 3.68‐3.59 (m, ca. 4H, OC*H*_2_ coordinated thf), 1.84‐1.74 (m, ca. 4H, OCH_2_C*H*_2_ coordinated thf), 0.04 (1 : 1 : 1 : 1 quartet ^1^ *J*_BH_=83.0 Hz, 4H, B*H*_4_). ^13^C^[25]^ NMR (75 MHz, thf‐d_8_, 293 K): δ (ppm), 92.6 (Cot), 68.0 (O*C*H_2_ coordinated thf), 26.1 (OCH_2_ *C*H_2_ coordinated thf). IR (ATR): ṽ=3021 (w), 2989 (w), 2930 (br m), 2889 (m), 2426 (m), 2272 (s), 2239 (s), 2164 (m), 2040 (w), 1855 (w), 1746 (w), 1610 (w), 1494 (w), 1453 (m), 1316 (br m), 1245 (m), 1213 (w), 1092 (s), 1012 (vs), 898 (s), 877 (vs), 752 (m), 706 (vs) cm‐1. No satisfactory EA was obtained.

**[Tb(Cot)(Cnt)]** (**1‘**): A brown acetonitrile solution of KCnt (2 mL, 42 mg, 0.27 mmol, 1.1 equiv) was added at r.t. to a toluene suspension of [Tb(Cot)I(thf)_2_] (10 mL, 149 mg, 0.25 mmol, 1 equiv). The resulting suspension was left to stir at r.t. for 12 h and was then dried under reduced pressure. The pale‐yellow residue was suspended in toluene (5 mL). After 1 h of stirring, the volatiles were removed under reduced pressure and the residue was further dried for 5 h at r.t. and extracted in several crops with large amounts of toluene. The pale‐yellow solution was filtered and cooled at −40 °C yielding X‐ray suitable pale yellow needles of **1‘** (32 mg, 29 %). ^1^H NMR (300 MHz, toluene‐d_8_, 293 K): δ (ppm), 246.3 (s, 8H, Cot), 101.6 (s, 9H, Cnt). The ^1^H NMR evolves with time and crystals of 1′ can be obtained after few days. IR (ATR): ṽ=3006 (m) 2920 (s), 2851 (s), 1935 (w), 1853 (w), 1746 (w), 1602 (w), 1551 (w), 1493 (w), 1457 (m), 1375 (w), 1313 (w), 1018 (w), 892 (vs), 846 (w), 772 (m), 747 (s), 704 (vs), 654 (vs) cm^−1^. Anal. Calcd. for C_17_H_17_Tb.0.2 Toluene (398.67): C, 55.43; H, 4.70; Found: C, 55.35; H, 5.03.

**[Tb(Cot)(Cnt)] (1′′)**: Toluene (20 mL) was condensed to a mixture of KCnt (30.0 mg, 0.192 mmol, 1 equiv), and [Tb(Cot)I(thf)_2_] (103 mg, 0.193 mmol; 1.01 eq) at −78 °C. The resulting suspension was heated to 150 °C for three hours and immediately passed through a syringe PTFE‐filter, while still hot. Upon slowly cooling the orange filtrate to r.t., single crystals of **1′′** suitable for X‐ray diffraction were obtained. Subsequent decantation of the mother liquor and drying *in vacuo* yielded **1′′** as orange crystalline solid (30.0 mg, 34 %). Raman (solid state, sealed ampule): ṽ [cm^−1^]=3044 (vw), 3008 (vw), 1515 (vw), 1495 (vw), 750 (s), 660(s), 366 (vw), 280 (w), 239 (s).

**[Dy(Cot)(Cnt)]** (**2**): A brown acetonitrile solution of KCnt (2 mL, 47 mg, 0.30 mmol, 1.1 equiv) was added at r.t. to a toluene suspension of [Dy(Cot)I(thf)_2_] (10 mL, 146 mg, 0.27 mmol, 1 equiv). The resulting suspension was left to stir at r.t. for 12 h and was then dried under reduced pressure. The yellow residue was suspended in toluene (5 mL). After 1 h of stirring, the volatiles were removed under reduced pressure and the residue was further dried for 5 h at r.t. and extracted in several crops with large amounts of toluene. The yellow solution was filtered and cooled at −40 °C yielding X‐ray suitable yellow needles of **2** (62.5 mg, 60 %). ^1^H NMR (300 MHz, toluene‐d_8_, 293 K): δ (ppm), 118.7 (s, 8H, Cot), 72.90 (s, 9H, Cnt). IR (ATR): ṽ=3005 (br m), 2917 (m), 1970 (w), 1851 (w), 1744 (w), 1601 (w), 1456 (m), 1373 (w), 1313 (w), 1018 (w), 892 (vs), 848 (w), 773 (m), 748 (s), 706 (vs), 654 (vs), 507 (w) cm^−1^. Anal. Calcd. for C_17_H_17_Dy (383.82): C, 53.20; H, 4.46; Found: C, 53.14; H, 4.48.

**[Ho(Cot)(Cnt)]** (**3**): A brown acetonitrile solution of KCnt (2 mL, 60 mg, 0.39 mmol, 1.05 equiv) was added at r.t. to a toluene suspension of [Ho(Cot)I(thf)_2_] (15 mL, 189 mg, 0.35 mmol, 1 equiv). The resulting suspension was left to stir at r.t. for 12 h and was then dried under reduced pressure. The pale‐orange residue was suspended in toluene (5 mL). After 1 h of stirring, the volatiles were removed under reduced pressure and the residue was further dried for 5 h at r.t. and extracted in several crops with large amounts of toluene. The pale‐orange solution was filtered and cooled at −40 °C yielding X‐ray suitable pale‐orange needles of **3** (77.8 mg, 67 %). ^1^H NMR (300 MHz, toluene‐d_8_, 293 K): δ (ppm), 90.45 (s, 8H, Cot), 59.17 (s, 9H, Cnt). IR (ATR): ṽ=3003 (br m), 2920 (m), 1969 (w), 1852 (w), 1745 (w), 1602 (w), 1455 (w), 1374 (w), 1313 (w), 1016 (m), 892 (s), 850 (w), 776 (m), 748 (m), 704 (vs), 656 (vs), 506 (m) cm^−1^. Anal. Calcd. for C_17_H_17_Ho (386.25): C, 52.86; H, 4.44; Found: C, 52.34; H, 4.53.

**[Er(Cot)(Cnt)]** (**4**): A brown acetonitrile solution of KCnt (2 mL, 65 mg, 0.42 mmol, 1.05 equiv) was added at r.t. to a toluene suspension of [Er(Cot)I(thf)_2_]_2_ (15 mL, 215 mg, 0.40 mmol, 1 equiv). The resulting suspension was left to stir at r.t. for 12 h and was then dried under reduced pressure. The orange residue was suspended in toluene (5 mL). After 1 h of stirring, the volatiles were removed under reduced pressure and the residue was further dried for 5 h at r.t. and extracted in several crops with large amounts of toluene. The orange solution was filtered and cooled at −40 °C yielding X‐ray suitable orange needles of **4** (73.6 mg, 63 %). ^1^H NMR (300 MHz, toluene‐d_8_, 293 K): δ (ppm),–5.01 (br s), −128.7 (br s). IR (ATR): ṽ=3030 (br m), 2959 (s), 2921 (m), 2851 (m), 1966 (w), 1854 (w), 1748 (w), 1604 (br w), 1451 (w), 1376 (w), 1313 (w), 1259 (s), 1087 (s), 1014 (s), 892 (s), 797 (s), 748 (m), 702 (vs), 657 (vs), 505 (m) cm^−1^. Anal. Calcd. for C_17_H_17_Er (388.58): C, 52.55; H, 4.41; Found: C, 52.80; H, 5.04.

**[Tm(Cot)(Cnt)]** (**5**): A brown acetonitrile solution of KCnt (2 mL, 65 mg, 0.42 mmol, 1.05 equiv) was added at r.t. to a toluene suspension of [Tm(Cot)I(thf)_2_] (15 mL, 217 mg, 0.40 mmol, 1 equiv). The resulting suspension was left to stir at r.t. for 12 h and was then dried under reduced pressure. The salmon‐orange residue was suspended in toluene (5 mL). After 1 h of stirring, the volatiles were removed under reduced pressure and the residue was further dried for 5 h at r.t. and extracted in several crops with large amounts of toluene. The salmon‐orange solution was filtered and cooled at −40 °C yielding X‐ray suitable salmon‐orange needles of **5** (74.6 mg, 48 %). ^1^H NMR (300 MHz, toluene‐d_8_, 293 K): δ (ppm), −23.21 (br s), −235.8 (br s). IR (ATR): ṽ=2998 (br m), 2920 (m), 2852 (m), 1965 (w), 1856 (w), 1753 (w), 1451 (w), 1314 (w), 1259 (w), 1016 (m), 893 (s), 781 (s), 749 (m), 703 (vs), 658 (vs), 505 (m) cm^−1^. Anal. Calcd. for C_17_H_17_Tm (390.25): C, 52.32; H, 4.39; Found: C, 52.80; H, 4.64.

**[Lu(Cot)(Cnt)]** (**6**): A mixture of [Lu(Cot)(BH_4_)(thf)]_2_ (146 mg, 0.33 mmol) and KCnt (52 mg, 0.33 mmol) in toluene (15 mL) was heated at 110 °C for 16 h and was then dried under reduced pressure for 2 h. The light yellow residue was extracted with hot toluene. The yellow filtrate was stored at −40 °C yielding yellow needles of **6** suitable for X‐ray diffraction studies (44 mg, 0.11 mmol, 34 %). ^1^H NMR (300 MHz, toluene‐d_8_, 293 K): δ (ppm), 6.54 (s, 9H, Cnt), 5.83 (s, 8H, Cot). ^13^C^[25]^ NMR (75 MHz, toluene‐d_8_, 293 K): δ (ppm), 107.8 (Cnt), 93.5 (Cot). IR (ATR): ṽ=2992 (br m), 1963 (w), 1857 (w), 1748 (w), 1604 (w), 1490 (w), 1447 (w), 1375 (w), 1313 (w), 1158 (w), 1018 (w), 892 (vs), 780 (m), 750 (m), 704 (vs), 662 (vs), 502 (w) cm^−1^. Anal. Calcd. for C_17_H_17_Lu (396.29): C, 51.52; H, 4.32; Found: C, 51.16; H, 4.34.

## Conflict of interest

The authors declare no conflict of interest.

## Supporting information

As a service to our authors and readers, this journal provides supporting information supplied by the authors. Such materials are peer reviewed and may be re‐organized for online delivery, but are not copy‐edited or typeset. Technical support issues arising from supporting information (other than missing files) should be addressed to the authors.

Supporting Information
