# Supplementary material for: Size‐Controlled Hapticity Switching in [Ln(C9H9)(C8H8)] Sandwiches
Source: Chemistry. 2021 Aug 21;27(54):13558–67. doi: 10.1002/chem.202101599 (PMC8518963; doi:10.1002/chem.202101599)
Supplement: Supplementary file 1 — Supporting Information [file CHEM-27-13558-s001.pdf]

# Chemistry–A European Journal

Supporting Information


## Size-Controlled Hapticity Switching in [$Ln(C_9H_9)(C_8H_8)$] Sandwiches


Maxime Tricoire, Luca Münzfeld, Jules Moutet, Nolwenn Mahieu, Léo La Droitte, Eufemio Moreno-Pineda, Frédéric Gendron, Jeremy D. Hilgar, Jeffrey D. Rinehart, Mario Ruben, Boris Le Guennic, Olivier Cador, Peter W. Roesky,* and Grégory Nocton*

**Outline.**

1. $^{1}$H NMR Spectroscopy........................................................ p S3

2. Magnetism.......................................................................... p S10

3. X-ray crystallography........................................................ p S11

4. Theoretical computations..................................................... p S116

5. References.......................................................................... p S126

### 3. $^1$H NMR Spectroscopy

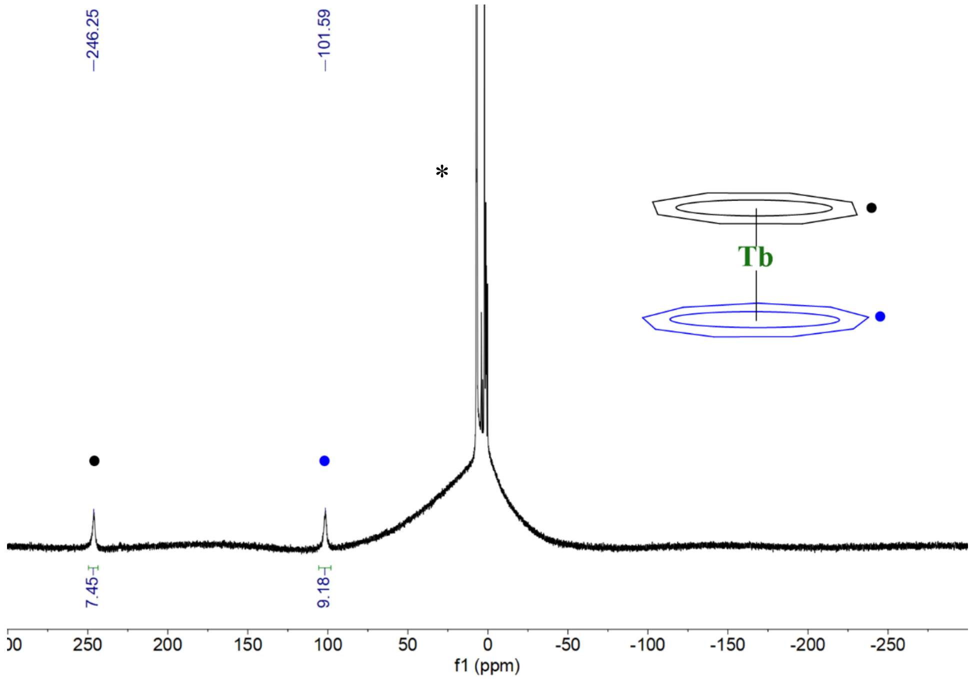


**Figure S1.** $^1$H NMR Spectrum of **1** at 293 K in toluene-$d_8$ (* residual protio signal of the solvent).

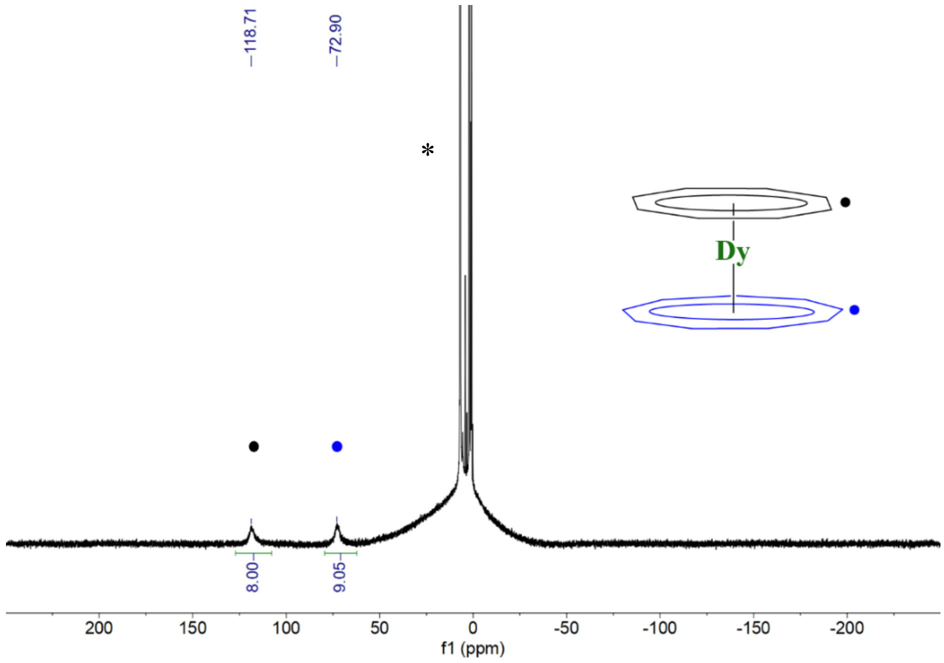


**Figure S2.** $^1$H NMR Spectrum of **2** at 293 K in toluene-$d_8$ (* residual protio signal of the solvent).

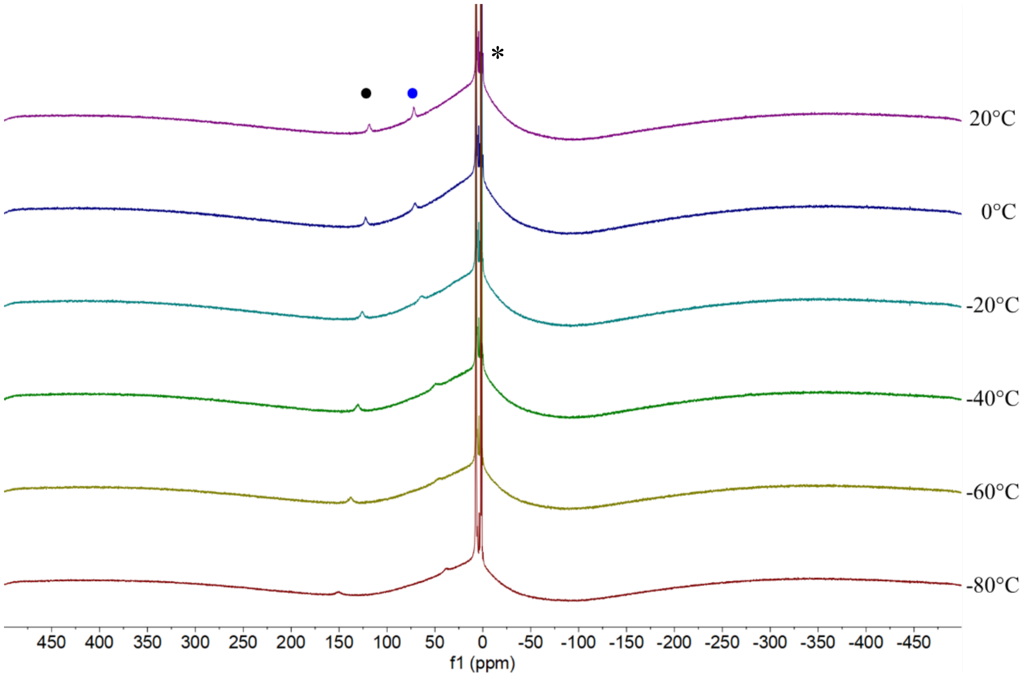


**Figure S3.** $^{1}$H NMR Spectrum of **2** at variable temperatures (from 193 K to 293 K) in toluene-$d_8$ (* residual protio signal of the solvent).

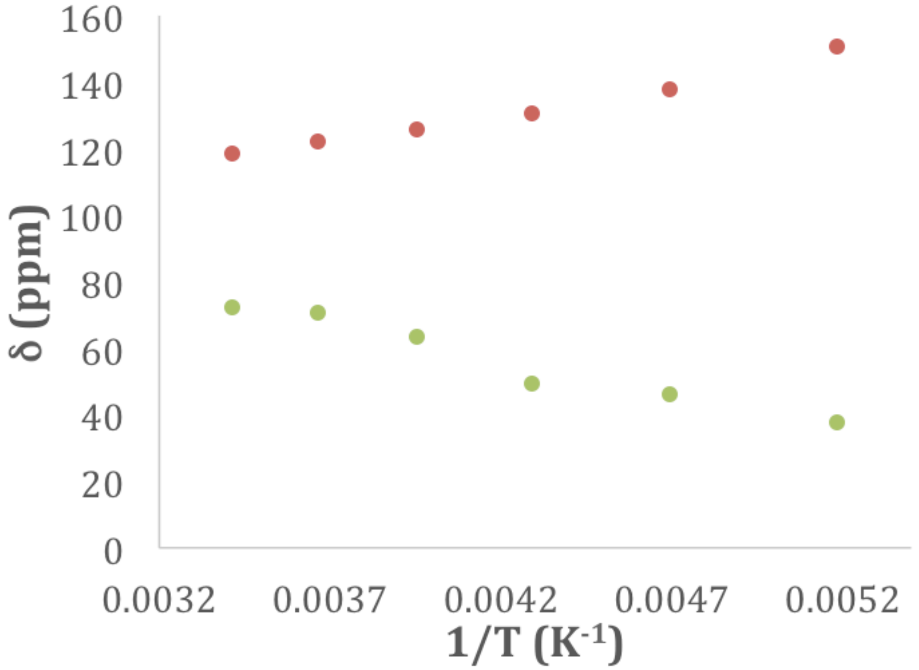


**Figure S4.** δ vs. 1/T plots of **2** at variable temperatures (from 193 K to 293 K) in toluene-$d_8$ (Cot in red, Cnt in green).

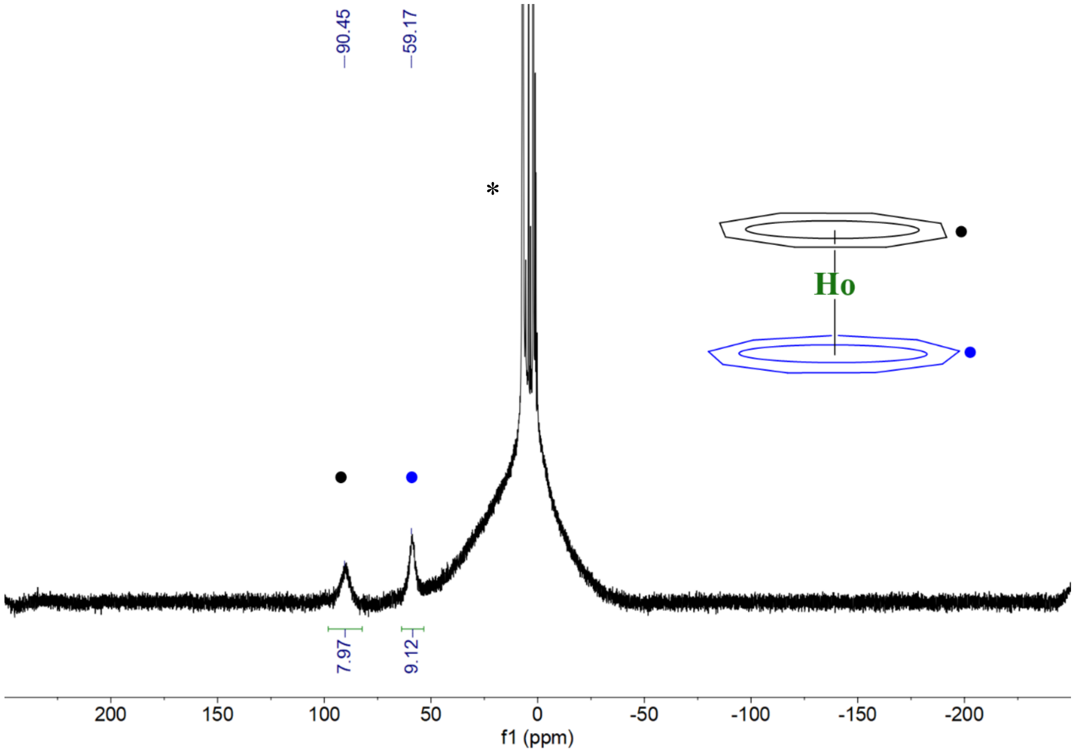


**Figure S5.** $^1$H NMR Spectrum of **3** at 293 K in toluene-$d_8$ (* residual protio signal of the solvent).

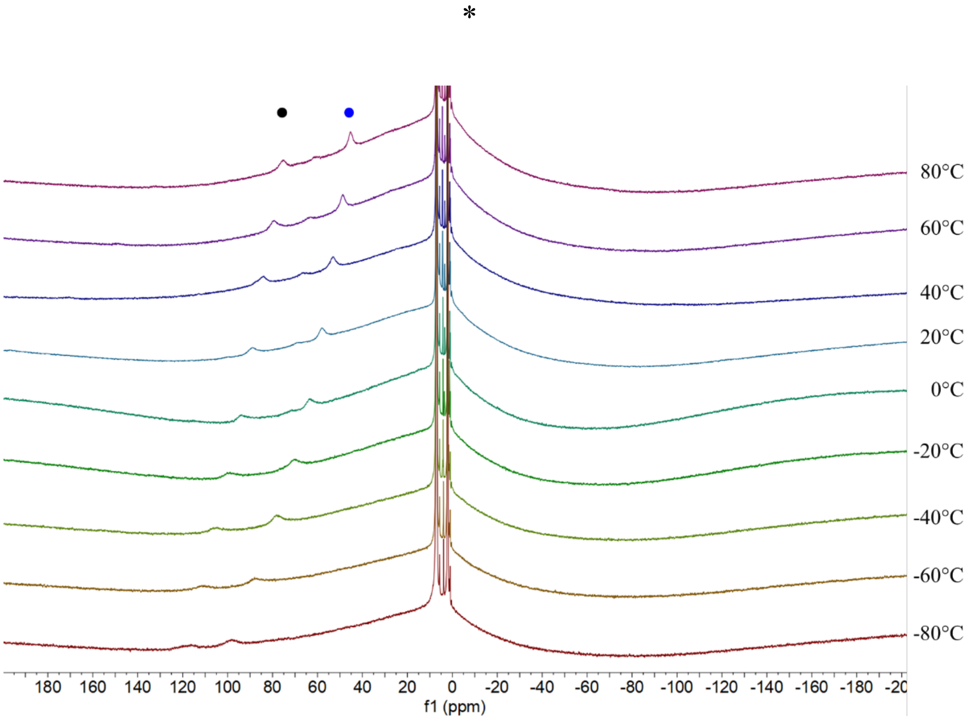


**Figure S6.** $^1$H NMR Spectrum of **3** at variable temperatures (from 193 K to 293 K) in toluene-$d_8$ (* for solvent).

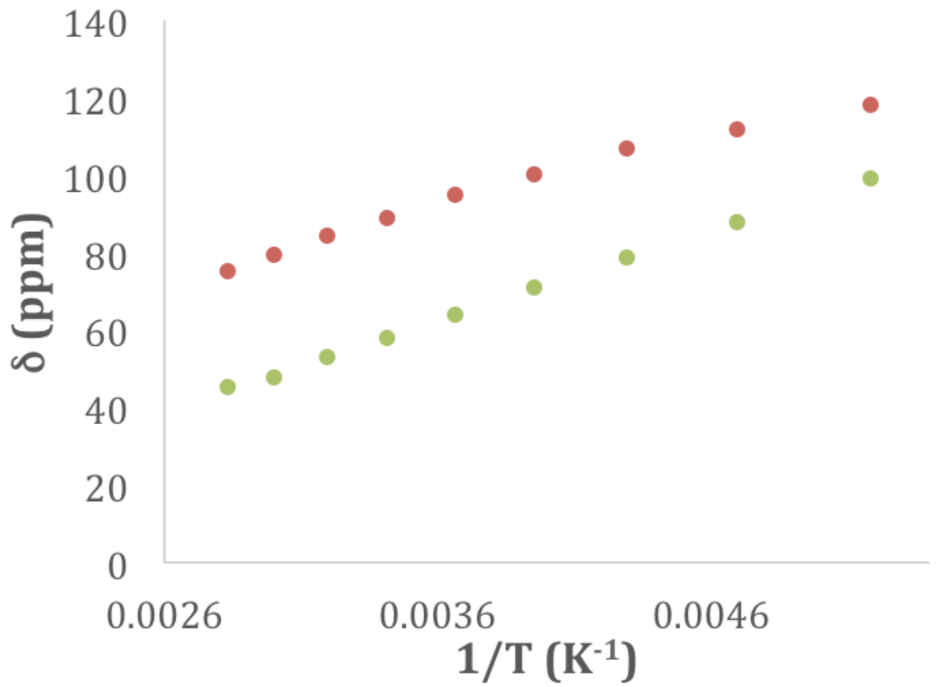


**Figure S7.** δ vs. 1/T plots of **3** at variable temperatures (from 193 K to 353 K) in toluene-$d_8$ (Cot in red, Cnt in green).

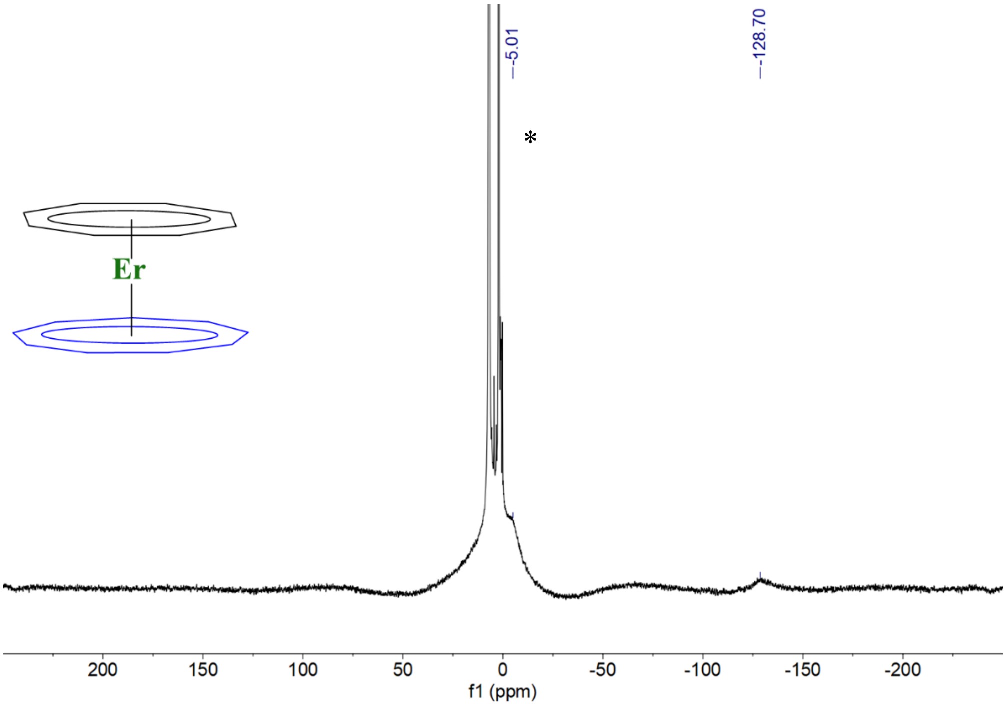


**Figure S8.** $^1$H NMR Spectrum of **4** at 293 K in toluene-$d_8$ (* residual protio signal of the solvent).

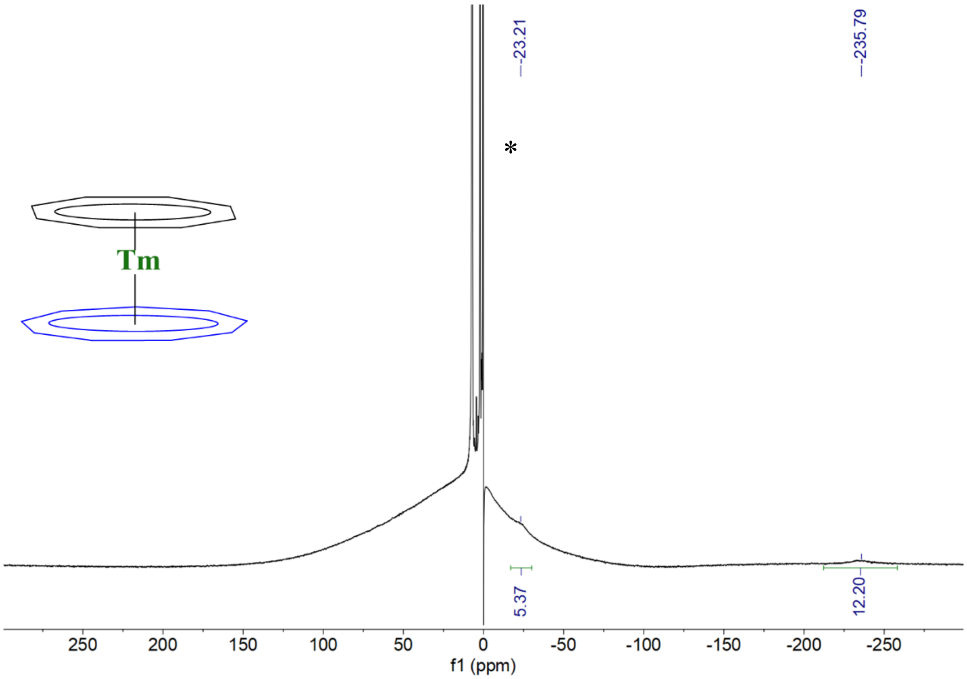


**Figure S9.** ${}^{1}$H NMR Spectrum of **5** at 293 K in toluene-$d_8$ (* residual protio signal of the solvent).

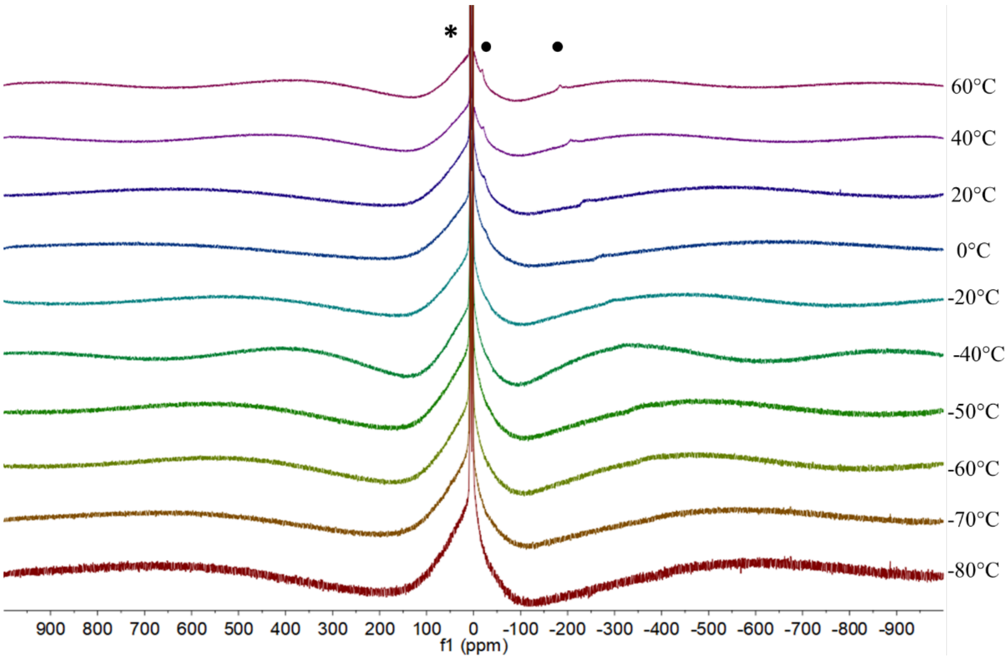


**Figure S10.** ${}^{1}$H NMR Spectrum of **5** at variable temperatures (from 193 K to 293 K) in toluene-$d_8$ (* residual protio signal of the solvent).

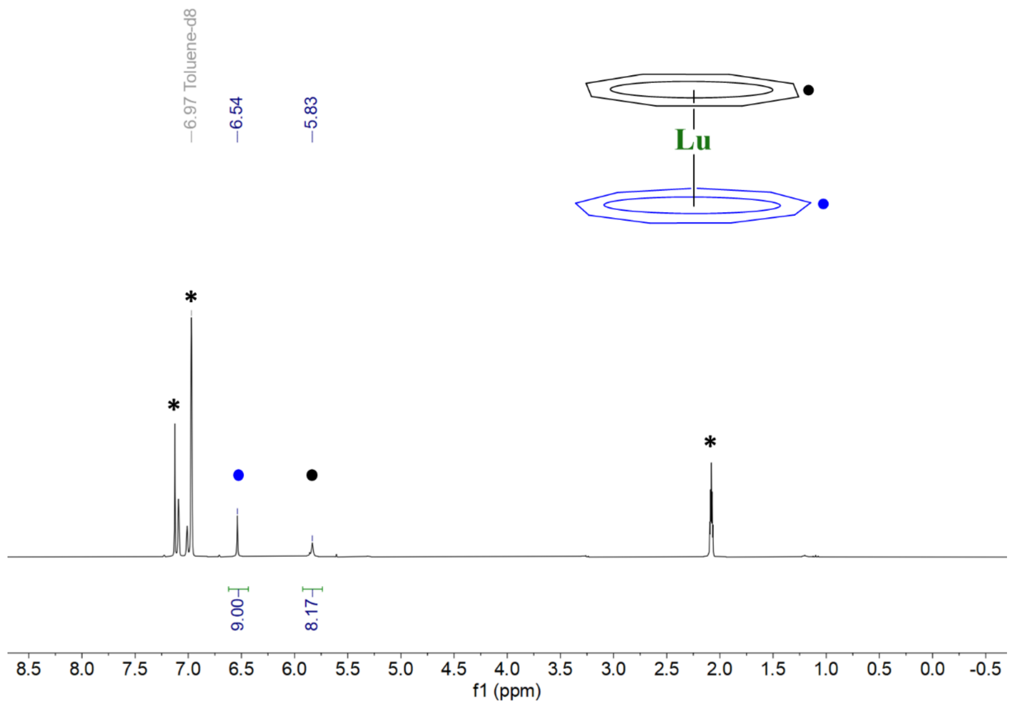


**Figure S11.** $^{1}H$ NMR Spectrum of **6** at 293 K in toluene-$d_8$ (* residual protio signal of the solvent).

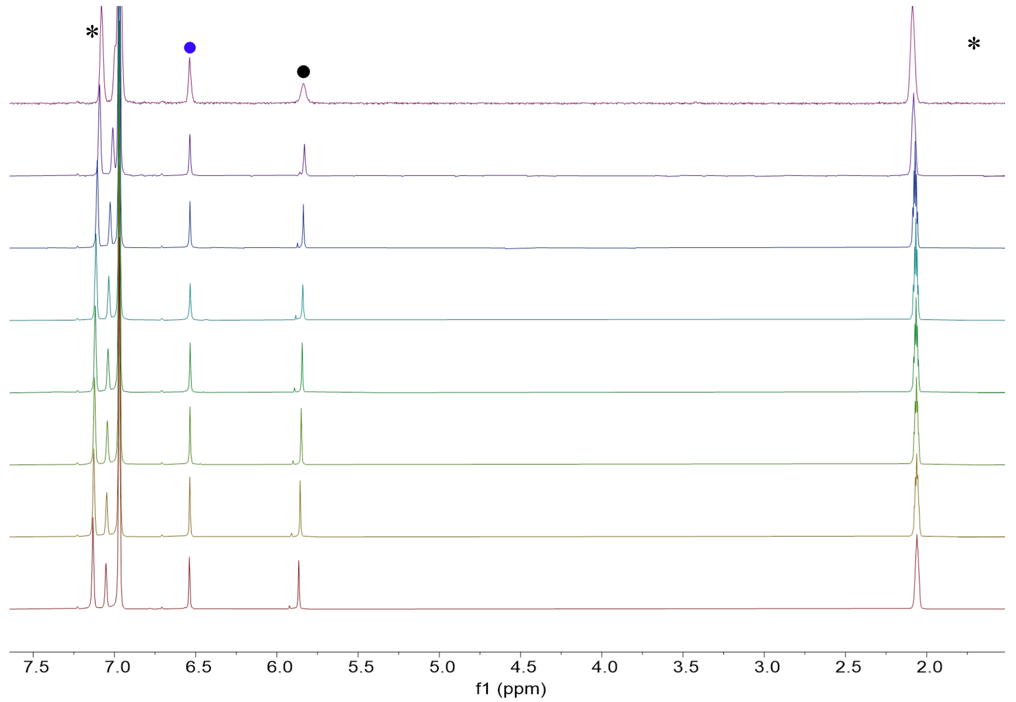


**Figure S12.** $^{1}H$ NMR Spectrum of **6** at variable temperatures (from 193 K to 293 K) in toluene-$d_8$ (* residual protio signal of the solvent).

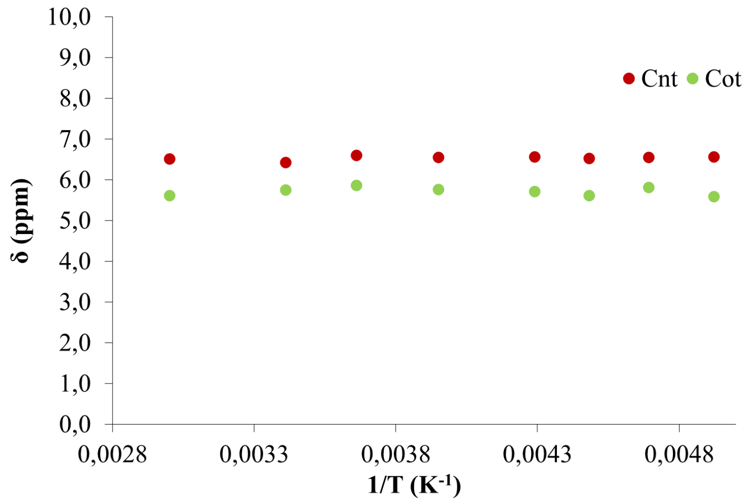


**Figure S13.** δ vs. 1/T plots of **6** at variable temperatures (from 193 K to 293 K) in toluene-$d_8$.

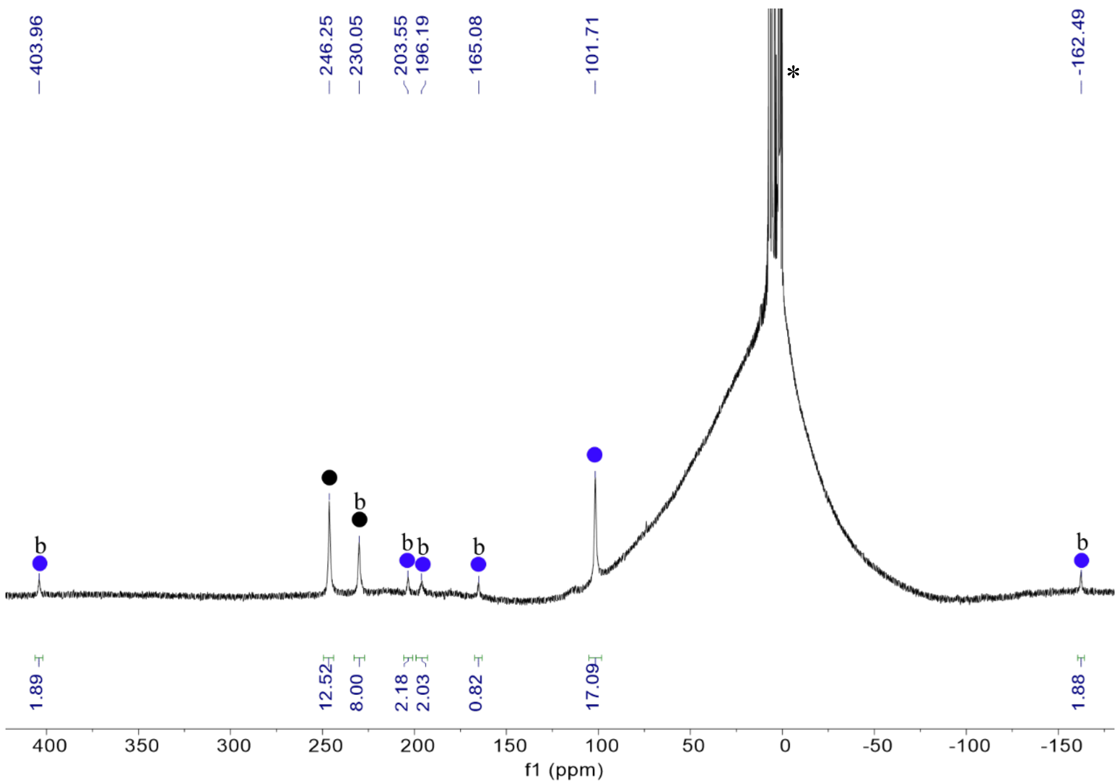


**Figure S14.** Evolution of the $^{1}$H NMR of **1** at room temperature for 45 days. **1b** Cot and Cnt peaks are labelled by a "b" note (* residual protio signal of the solvent; Cot in black, Cnt in blue)..

## 4. Magnetic measurements.

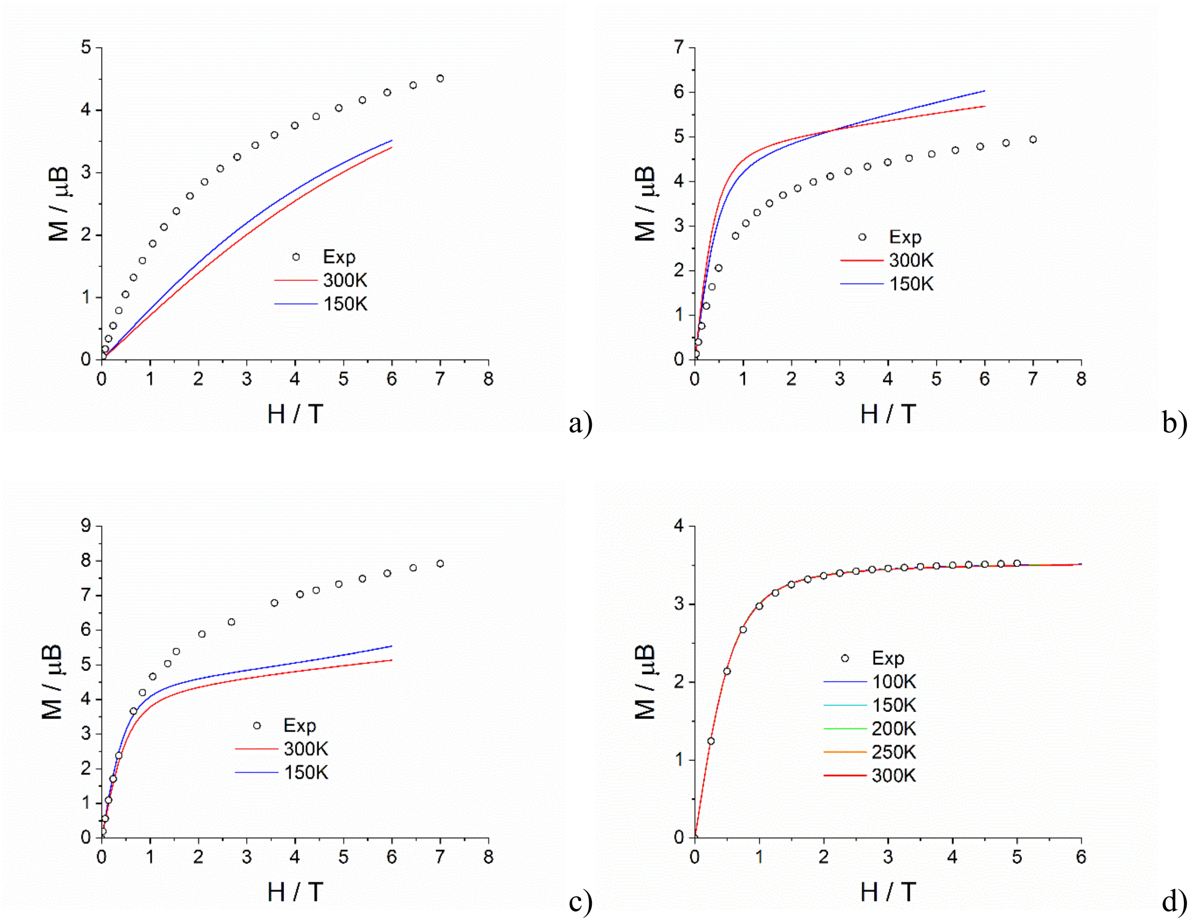


**Figure S15.** Field dependent magnetization values at 2K for compounds **1** (a), **2** (b), **3** (c) and **5** (d) in dots with the calculated curves from structures at various temperatures (all curves are superimposed for compound **5**).

## 5. X-ray crystallography

**General details.** The structure resolution was accomplished using the SHELXS-97 [5] and SHELXT [6] programs and the refinement was done with the SHELXL [7] program. The structure solution and the refinement were achieved with the PLATON and OLEX2 softwares.[8],[9] Finally, pictures of the compound structure were obtained using the MERCURY[10] and ORTEP3[11] softwares. During the refinement steps, all atoms - except hydrogen atoms - were refined anisotropically. The position of the hydrogen atoms was determined using residual electronic densities, which are calculated by a Fourier difference. Finally, in order to obtain a complete refinement, a weighting step followed by multiples loops of refinement was done. The crystal structures of [Tb(Cot)I(THF)$_2$], [Ho(Cot)I(THF)$_2$], [Lu(Cot)(BH$_4$)(THF)]$_2$ and **1-6** at each temperature measured have been deposited in the CCDC with #2073511-2073531.

For the data of **1'** and **1''**, which are very similar species, the same phenomenon occurs with residual density observed close to the Tb atom. The three B alerts for residual density (**1** in **1'**, **2** in **1''**) results from this. This could be explained by the very high angle data obtained with these two acquisitions. The residual density map represents the valence electron cloud that starts to be measurable at high angle. The absorption correction model used, is then not able to accuratly take this contribution into account  This was then considered as a measurement artifact, an atom at this position having no chemical sense. For the data of 5 at 100 K a similar residual density is also present resulting in a B alert. We came to the same conclusions as for compounds **1'** and **1''**.

**Table S1.** Crystallographic parameters for [Tb(Cot)I(THF)$_2$], [Ho(Cot)I(THF)$_2$] and [Lu(Cot)($BH_4$)(THF)]$_2$.

| **Compound** | [Tb(Cot)I(THF)$_2$] | [Ho(Cot)I(THF)$_2$] | [Lu(Cot)($BH_4$)(THF)]$_2$ |
|---|---|---|---|
| Formula | $C_{16}H_{24}TbIO_2$ | $C_{16}H_{24}HoIO_2$ | $C_{24}H_{40}B_2Lu_2O_2$ |
| Crystal size (mm) | 0.3 × 0.3 × 0.26 | 0.22 × 0.08 × 0.08 | 0.12 x 0.1 x 0.04 |
| Crystal system | monoclinic | monoclinic | monoclinic |
| Space group | $P2_1/n$ | $P2_1/n$ | $P2_1/c$ |
| Volume (Å$^3$) | 1766.38(13) | 1750.3(2) | 1240.90(18) |
| a (Å) | 8.5442(3) | 8.4864(6) | 9.5563(9) |
| b (Å) | 9.3864(4) | 9.3831(7) | 11.2696(9) |
| c (Å) | 22.2233(10) | 22.1744(16) | 12.4093(10) |
| α (deg) | 90 | 90 | 90 |
| β (deg) | 97.6620(14) | 97.579(3) | 111.795(3) |
| γ (deg) | 90 | 90 | 90 |
| Z | 4 | 4 | 2 |
| Formula weight (g/mol) | 534.17 | 540.18 | 732.12 |
| Density (calcd) (g/cm$^3$) | 2.009 | 2.050 | 1.959 |
| Absorption coefficient (mm$^{-1}$) | 5.751 | 6.283 | 7.926 |
| F(000) | 1016.0 | 1024.0 | 704.0 |
| Temp (K) | 219.99 | 219.99 | 150 |
| diffractometer | Kappa APEX II CCD | Kappa APEX II CCD | Kappa APEX II CCD |
| Radiation | MoKα (λ = 0.71073) | MoKα (λ = 0.71073) | MoKα (λ = 0.71073) |
| 2θ range for data collection (deg) | 5.38 to 60.066 | 3.706 to 60.064 | 4.59 to 60.212 |
| Absorption correction | Multi-scan | Multi-scan | Multi-scan |
| Total no. reflections | 33135 | 84933 | 27886 |
| Unique reflections [$R_{int}$] | 5155 [$R_{int}$ = 0.0477] | 5112 [$R_{int}$ = 0.0511] | 3654 [$R_{int}$ = 0.0766] |
| Final R indices [I>2σ(I)] | R = 0.0417, $R_w$ = 0.0841 | R = 0.0436, $R_w$ = 0.0924 | R = 0.0330, $R_w$ = 0.0654 |
| R indices (all data) | R = 0.0529, $R_w$ = 0.0884 | R = 0.0484, $R_w$ = 0.0946 | R = 0.0523, $R_w$ = 0.0717 |
| Largest diff. peak and hole (e.A$^{-3}$) | 0.85/-1.82 | 1.44/-1.52 | 1.14/-1.17 |
| GooF | 1.271 | 1.346 | 1.030 |

**Table S2.** Crystallographic parameters for **1** at 150 K and 300 K and **2** at 150 K.

| **Compound** | [Tb(Cot)(Cnt)] **(1)** at 150 K | [Tb(Cot)(Cnt)] **(1)** at 300 K | [Dy(Cot)(Cnt)] **(2)** at 150 K |
|---|---|---|---|
| Formula | $C_{17}H_{17}Tb$ | $C_{17}H_{17}Tb$ | $C_{17}H_{17}Dy$ |
| Crystal size (mm) | 0.16 × 0.04 × 0.04 | 0.18 × 0.02 × 0.02 | 0.11 × 0.04 × 0.04 |
| Crystal system | orthorhombic | orthorhombic | monoclinic |
| Space group | *Pnma* | *Pnma* | $P2_1/n$ |
| Volume ($Å^3$) | 1345.4(5) | 1366.9(13) | 671.97(11) |
| a (Å) | 11.939(2) | 12.002(7) | 7.1626(7) |
| b (Å) | 12.952(3) | 13.032(7) | 8.7103(8) |
| c (Å) | 8.7005(17) | 8.739(5) | 10.9560(11) |
| α (deg) | 90 | 90 | 90 |
| β (deg) | 90 | 90 | 100.551(4) |
| γ (deg) | 90 | 90 | 90 |
| Z | 4 | 4 | 2 |
| Formula weight (g/mol) | 380.22 | 380.22 | 383.80 |
| Density (calcd) ($g/cm^3$) | 1.877 | 1.848 | 1.897 |
| Absorption coefficient ($mm^{-1}$) | 5.234 | 5.152 | 5.538 |
| F(000) | 736.0 | 736.0 | 370.0 |
| Temp (K) | 149.99 | 300.15 | 149.99 |
| diffractometer | Kappa APEX II CCD | Kappa APEX II CCD | Kappa APEX II CCD |
| Radiation | MoKα (λ = 0.71073) | MoKα (λ = 0.71073) | MoKα (λ = 0.71073) |
| 2θ range for data collection (deg) | 5.64 to 58.256 | 5.612 to 52.044 | 7.442 to 60.632 |
| Absorption correction | Multi-scan | Multi-scan | Multi-scan |
| Total no. reflections | 27069 | 23335 | 10251 |
| Unique reflections [$R_{int}$] | 1881 [$R_{int}$ = 0.1360] | 1410 [$R_{int}$ = 0.2142] | 1977 [$R_{int}$ = 0.0528] |
| Final R indices [I>2σ(I)] | R = 0.0422, $R_w$ = 0.0986 | R = 0.0622, $R_w$ = 0.1285 | R = 0.0383, $R_w$ = 0.0783 |
| R indices (all data) | R = 0.0762, $R_w$ = 0.1156 | R = 0.1247, $R_w$ = 0.1675 | R = 0.0609, $R_w$ = 0.0856 |
| Largest diff. peak and hole ($e.A^{-3}$) | 2.00/-0.94 | 2.62/-1.77 | 1.26/-0.73 |
| GooF | 1.025 | 1.041 | 1.076 |

**Table S3.** Crystallographic parameters for **2** at 150 K, **3** at 150 K and 300 K.

| **Compound** | [Dy(Cot)(Cnt)] **(2)** at 300 K | [Ho(Cot)(Cnt)] **(3)** at 150 K | [Ho(Cot)(Cnt)] **(3)** at 300 K |
|---|---|---|---|
| Formula | $C_{17}H_{17}Dy$ | $C_{17}H_{17}Ho$ | $C_{17}H_{17}Ho$ |
| Crystal size (mm) | 0.3 × 0.08 × 0.07 | 0.16 × 0.07 × 0.04 | 0.16 × 0.07 × 0.04 |
| Crystal system | monoclinic | monoclinic | monoclinic |
| Space group | $P2_1/n$ | $P2_1/n$ | $P2_1/n$ |
| Volume (Å$^3$) | 695.1(5) | 669.36(13) | 689.3(2) |
| *a* (Å) | 7.253(3) | 7.0595(8) | 7.1615(13) |
| *b* (Å) | 8.808(4) | 8.7192(9) | 8.7931(16) |
| *c* (Å) | 11.070(5) | 11.0938(12) | 11.163(2) |
| *α* (deg) | 90 | 90 | 90 |
| *β* (deg) | 100.628(13) | 101.409(4) | 101.321(7) |
| *γ* (deg) | 90 | 90 | 90 |
| *Z* | 2 | 2 | 2 |
| Formula weight (g/mol) | 383.80 | 386.23 | 386.23 |
| Density (calcd) (g/cm$^3$) | 1.834 | 1.916 | 1.861 |
| Absorption coefficient (mm$^{-1}$) | 5.353 | 5.888 | 5.718 |
| F(000) | 370.0 | 372.0 | 372.0 |
| Temp (K) | 300.0 | 150.0 | 300.0 |
| diffractometer | Kappa APEX II CCD | Kappa APEX II CCD | Kappa APEX II CCD |
| Radiation | MoKα ($\lambda$ = 0.71073) | MoKα ($\lambda$ = 0.71073) | MoKα ($\lambda$ = 0.71073) |
| 2θ range for data collection (deg) | 5.95 to 65.148 | 7.494 to 60.042 | 6.248 to 54.938 |
| Absorption correction | Multi-scan | Multi-scan | Multi-scan |
| Total no. reflections | 17194 | 13553 | 10319 |
| Unique reflections [$R_{int}$] | 2531 [$R_{int}$ = 0.0382] | 1959 [$R_{int}$ = 0.0517] | 1581 [$R_{int}$ = 0.0452] |
| Final *R* indices [I>2σ(I)] | $R$ = 0.0349, $R_w$ = 0.0835 | R = 0.0514, $R_w$ = 0.1005 | $R$ = 0.0308, $R_w$ = 0.0637 |
| *R* indices (all data) | $R$ = 0.0461, $R_w$ = 0.0905 | $R$ = 0.0649, $\mathrm{R_w}$ = 0.1042 | $R$ = 0.0494, $R_w$ = 0.0707 |
| Largest diff. peak and hole (e.A$^{-3}$) | 1.57/-0.54 | 1.17/-1.48 | 0.66/-0.39 |
| GooF | 1.074 | 1.287 | 1.039 |

**Table S4.** Crystallographic parameters for **4** at 150 K and 300 K and **5** at 150 K.

| **Compound** | [Er(Cot)(Cnt)] **(4)** at 150 K | [Er(Cot)(Cnt)] **(4)** at 300 K | [Tm(Cot)(Cnt)] **(5)** at 150 K |
|---|---|---|---|
| Formula | $C_{17}H_{17}Er$ | $C_{17}H_{17}Er$ | $C_{17}H_{17}Tm$ |
| Crystal size (mm) | 0.24 × 0.06 × 0.04 | 0.27 × 0.1 × 0.05 | 0.15 × 0.04 × 0.04 |
| Crystal system | monoclinic | monoclinic | monoclinic |
| Space group | $P2_1/n$ | $P2_1/n$ | $P2_1/n$ |
| Volume (Å$^3$) | 666.72(13) | 687.88(19) | 666.12(11) |
| a (Å) | 6.9928(8) | 7.0902(12) | 6.9926(7) |
| b (Å) | 8.7303(9) | 8.8108(13) | 8.7084(8) |
| c (Å) | 11.1760(12) | 11.2593(17) | 11.2040(11) |
| α (deg) | 90 | 90 | 90 |
| β (deg) | 102.264(4) | 102.047(6) | 102.489(4) |
| γ (deg) | 90 | 90 | 90 |
| Z | 2 | 2 | 2 |
| Formula weight (g/mol) | 388.56 | 388.56 | 390.23 |
| Density (calcd) (g/cm$^3$) | 1.936 | 1.876 | 1.946 |
| Absorption coefficient (mm$^{-1}$) | 6.271 | 6.078 | 6.637 |
| F(000) | 374.0 | 374.0 | 376.0 |
| Temp (K) | 150.15 | 300.0 | 150.0 |
| diffractometer | Kappa APEX II CCD | Kappa APEX II CCD | Kappa APEX II CCD |
| Radiation | MoKα (λ = 0.71073) | MoKα (λ = 0.71073) | MoKα (λ = 0.71073) |
| 2θ range for data collection (deg) | 5.974 to 66.238 | 7.402 to 58.248 | 5.98 to 64.054 |
| Absorption correction | Multi-scan | Multi-scan | Multi-scan |
| Total no. reflections | 27235 | 3781 | 15568 |
| Unique reflections [$R_{int}$] | 2540 [$R_{int}$ = 0.1099] | 1839 [$R_{int}$ = 0.0363] | 2318 [$R_{int}$ = 0.0616] |
| Final R indices [I>2σ(I)] | R = 0.0462, $R_w$ = 0.1088 | R = 0.0549, $R_w$ = 0.1217 | R = 0.0311, $R_w$ = 0.0627 |
| R indices (all data) | R = 0.0620, $R_w$ = 0.1127 | R = 0.0625, $R_w$ = 0.1253 | R = 0.0461, $R_w$ = 0.0662 |
| Largest diff. peak and hole (e.A$^{-3}$) | 1.20/-2.01 | 1.05/-1.00 | 0.54/-1.00 |
| GooF | 1.307 | 1.226 | 1.122 |

**Table S5.** Crystallographic parameters for **5** at 300 K, **6** at 150 K and 300 K.

| **Compound** | [Tm(Cot)(Cnt)] **(5)** at 300 K | [Lu(Cot)(Cnt)] **(6)** at 150 K | [Lu(Cot)(Cnt)] **(6)** at 300 K |
|---|---|---|---|
| Formula | $C_{17}H_{17}Tm$ | $C_{17}H_{17}Lu$ | $C_{17}H_{17}Lu$ |
| Crystal size (mm) | 0.2 × 0.04 × 0.02 | 0.1 × 0.06 × 0.06 | 0.1 × 0.06 × 0.06 |
| Crystal system | monoclinic | monoclinic | monoclinic |
| Space group | $P2_1/n$ | $P2_1/n$ | $P2_1/n$ |
| Volume (Å$^3$) | 688.4(3) | 665.3(2) | 689.6(9) |
| a (Å) | 7.091(2) | 6.9721(12) | 7.067(5) |
| b (Å) | 8.805(3) | 8.7073(14) | 8.796(6) |
| c (Å) | 11.287(3) | 11.264(2) | 11.414(9) |
| α (deg) | 90 | 90 | 90 |
| β (deg) | 102.343(10) | 103.361(7) | 103.60(3) |
| γ (deg) | 90 | 90 | 90 |
| Z | 2 | 2 | 2 |
| Formula weight –(g/mol) | 390.23 | 396.27 | 396.27 |
| Density (calcd) (g/cm$^3$) | 1.883 | 1.978 | 1.908 |
| Absorption coefficient (mm$^{-1}$) | 6.422 | 7.397 | 7.136 |
| F(000) | 376.0 | 380.0 | 380.0 |
| Temp (K) | 300.0 | 150.0 | 300.0 |
| diffractometer | Kappa APEX II CCD | Kappa APEX II CCD | Kappa APEX II CCD |
| Radiation | MoKα (λ = 0.71073) | MoKα (λ = 0.71073) | MoKα (λ = 0.71073) |
| 2θ range for data collection (deg) | 5.922 to 60.334 | 5.976 to 65.154 | 6.198 to 60.06 |
| Absorption correction | Multi-scan | Multi-scan | Multi-scan |
| Total no. reflections | 14306 | 13809 | 12454 |
| Unique reflections [$R_{int}$] | 2020 [$R_{int}$ = 0.1199] | 2416 [$R_{int}$ = 0.0964] | 2015 [$R_{int}$ = 0.0609] |
| Final R indices [I>2σ(I)] | R = 0.0447, $R_w$ = 0.0947 | R = 0.0392, $R_w$ = 0.0757 | R = 0.0283, $R_w$ = 0.0581 |
| R indices (all data) | R = 0.0768, $R_w$ = 0.0993 | R = 0.0685, $R_w$ = 0.0881 | R = 0.0461, $R_w$ = 0.0636 |
| Largest diff. peak and hole (e.A$^{-3}$) | 0.49/-1.17 | 0.64/-1.14 | 0.43/-0.51 |
| GooF | 0.976 | 1.035 | 1.024 |

**Table S6.** Crystallographic parameters for **1b** at 150 K, **5** at 100 K and 200 K.

| **Compound** | [Tb(*cis*-Cnt)$_x$(*trans*-Cnt)$_{1-x}$(Cot)Tb] x=0.79 **(1')** at 150 K | [Tb(*cis*-Cnt)$_x$(*trans*-Cnt)$_{1-x}$(Cot)Tb] x=0.55 **(1'')** at 100 K | [Tm(Cot)(Cnt)] **(5)** at 100 K |
|---|---|---|---|
| Formula | $C_{17}H_{17}Tb$ | $C_{17}H_{17}Tb$ | $C_{17}H_{17}Tm$ |
| Crystal size (mm) | 0.14 × 0.04 × 0.02 | 0.325 × 0.148 × 0.052 | 0.21 × 0.04 × 0.02 |
| Crystal system | orthorhombic | monoclinic | monoclinic |
| Space group | *Pnma* | $P2_1/n$ | $P2_1/n$ |
| Volume (Å$^3$) | 1347.71(16) | 1327.4 | 661.00(15) |
| a (Å) | 11.9205(8) | 8.680 | 6.9750(9) |
| b (Å) | 12.9752(9) | 11.876 | 8.6935(12) |
| c (Å) | 8.7134(6) | 12.876 | 11.1619(14) |
| α (deg) | 90 | 90 | 90 |
| β (deg) | 90 | 90.01 | 102.413(3) |
| $\gamma$ (deg) | 90 | 90 | 90 |
| Z | 4 | 4 | 2 |
| Formula weight (g/mol) | 380.22 | 380.22 | 390.23 |
| Density (calcd) (g/cm$^3$) | 1.874 | 1.903 | 1.961 |
| Absorption coefficient (mm$^{-1}$) | 5.225 | 5.305 | 6.689 |
| F(000) | 736.0 | 736.0 | 376.0 |
| Temp (K) | 150.01 | 100.0 | 100.15 |
| diffractometer | Kappa APEX II CCD | STOE STADIVARI | Kappa APEX II CCD |
| Radiation | MoKα (λ = 0.71073) | MoKα (λ = 0.71073) | MoKα (λ = 0.71073) |
| 2θ range for data collection (deg) | 5.632 to 77.358 | 4.666 to 76.27 | 5.994 to 60.036 |
| Absorption correction | Multi-scan | Multi-scan | Multi-scan |
| Total no. reflections | 48996 | 24164 | 13322 |
| Unique reflections [$R_{int}$] | 3967 [$R_{int}$ = 0.0619] | 6827 [$R_{int}$ = 0.0348] | 1932 [$R_{int}$ = 0.0565] |
| Final R indices [I>2σ(I)] | R = 0.0269, $R_w$ = 0.0527 | R = 0.0346, $R_w$ = 0.0770 | R = 0.0484, $R_w$ = 0.1179 |
| R indices (all data) | R = 0.0392, $R_w$ = 0.0575 | R = 0.0571, $R_w$ = 0.0875 | R = 0.0545, $R_w$ = 0.1198 |
| Largest diff. peak and hole (e.A$^{-3}$) | 2.79/-1.37 | 3.64/-2.50 | 1.87/-2.46 |
| GooF | 1.021 | 1.028 | 1.416 |

**Table S7.** Crystallographic parameters for **5** at 250 K and 1/2 [$(C_9H_9)(C_8H_8)Dy$] ; [$(C_9H_9)(C_8H_8)Dy(NCMe)$] at 150 K .

| **Compound** | [Tm(Cot)(Cnt)] **(5)** at 200 K | [Tm(Cot)(Cnt)] **(5)** at 250 K | 1/2[Dy(Cot)(Cnt)]; [Dy(Cot)(Cnt)(NCMe)] at 150 K (**2b**) |
|---|---|---|---|
| Formula | $C_{17}H_{17}Tm$ | $C_{17}H_{17}Tm$ | $C_{28.5}H_{30.5}Cl_2Dy_{1.5}N$ |
| Crystal size (mm) | 0.21 × 0.04 × 0.02 | 0.21 × 0.04 × 0.02 | 0.3 × 0.1 × 0.03 |
| Crystal system | monoclinic | monoclinic | monoclinic |
| Space group | $P2_1/n$ | $P2_1/n$ | $P2_1/n$ |
| Volume (Å$^3$) | 669.4(3) | 678.78(10) | 2573.5(8) |
| a (Å) | 7.0155(17) | 7.0571(6) | 8.4461(15) |
| b (Å) | 8.727(2) | 8.7567(7) | 26.988(5) |
| c (Å) | 11.196(3) | 11.2488(10) | 11.290(2) |
| α (deg) | 90 | 90 | 90 |
| β (deg) | 102.440(10) | 102.456(4) | 90.128(6) |
| γ (deg) | 90 | 90 | 90 |
| Z | 2 | 2 | 4 |
| Formula weight (g/mol) | 390.23 | 390.23 | 701.69 |
| Density (calcd) (g/cm$^3$) | 1.936 | 1.909 | 1.811 |
| Absorption coefficient (mm$^{-1}$) | 6.605 | 6.513 | 4.558 |
| F(000) | 376.0 | 376.0 | 1366.0 |
| Temp (K) | 200.15 | 250.15 | 150 |
| diffractometer | Kappa APEX II CCD | Kappa APEX II CCD | Kappa APEX II CCD |
| Radiation | MoKα (λ = 0.71073) | MoKα (λ = 0.71073) | MoKα (λ = 0.71069) |
| 2θ range for data collection (deg) | 5.974 to 62.376 | 5.95 to 61.03 | 3.018 to 60.058 |
| Absorption correction | Multi-scan | Multi-scan | Multi-scan |
| Total no. reflections | 11543 | 12016 | 7506 |
| Unique reflections [$R_{int}$] | 2161 [$R_{int}$ = 0.0518] | 2062 [$R_{int}$ = 0.0447] | 7506 |
| Final R indices [I>2σ(I)] | R = 0.0509, $R_w$ = 0.1149 | R = 0.0463, $R_w$ = 0.1064 | R = 0.0391, $R_w$ = 0.0836 |
| R indices (all data) | R = 0.0645, $R_w$ = 0.1180 | R = 0.0549, $R_w$ = 0.1087 | R = 0.0447, $R_w$ = 0.0866 |
| Largest diff. peak and hole (e.A$^{-3}$) | 1.21/-1.98 | 1.03/-1.38 | 1.60/-2.85 |
| GooF | 1.317 | 1.325 | 1.133 |

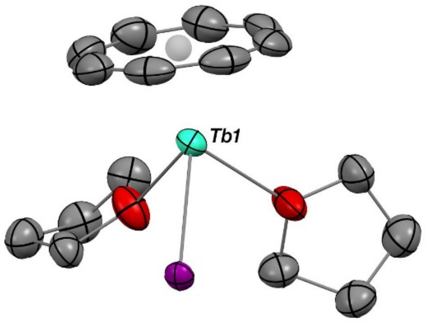


**Figure S16.** ORTEP of [Tb(Cot)I(THF)$_2$]. Thermal ellipsoids are depicted at 50 % probability level. Carbon atoms are in grey (centroid in light grey), iodide atom in purple, oxygen atoms in red and terbium atom in light green. Disordered positions and hydrogen atoms have been omitted for clarity.

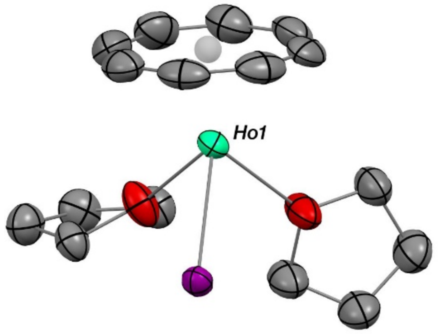


**Figure S17.** ORTEP of [Ho(Cot)I(THF)$_2$]. Thermal ellipsoids are depicted at 50 % probability level Carbon atoms are in grey (centroid in light grey), iodide atom in purple, oxygen atoms in red and holmium atom in light green. Disordered positions and hydrogen atoms have been removed for clarity.

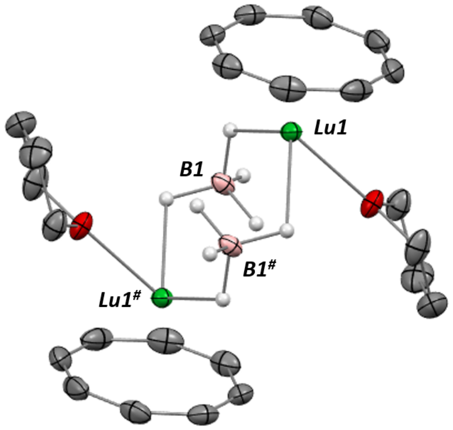


**Figure S18.** ORTEP of [Lu(Cot)(BH$_4$)(THF)]$_2$. Thermal ellipsoids are depicted at 50 % probability level Carbon atoms are in grey, boron atoms in pink, oxygen atoms in red and lutetium atom in light green. Hydrogen atoms except on the boron atom have been removed for clarity.

**Discussion of the crystal structure** of $Tb(Cot)I(THF)_2$, $Ho(Cot)I(THF)_2$.

The $[Tb(Cot)I(THF)_2]$ and $[Ho(Cot)I(THF)_2]$ structures were measured at 220 K to avoid a possible phase transition at 150 K temperature at which the data were not good. In the crystal structure of $[Lu(Cot)(BH_4)(THF)]_2$, the H atoms on the boron have been localized in the difference Fourier map and the B-H bond lengths restrained to 1.12 Å.

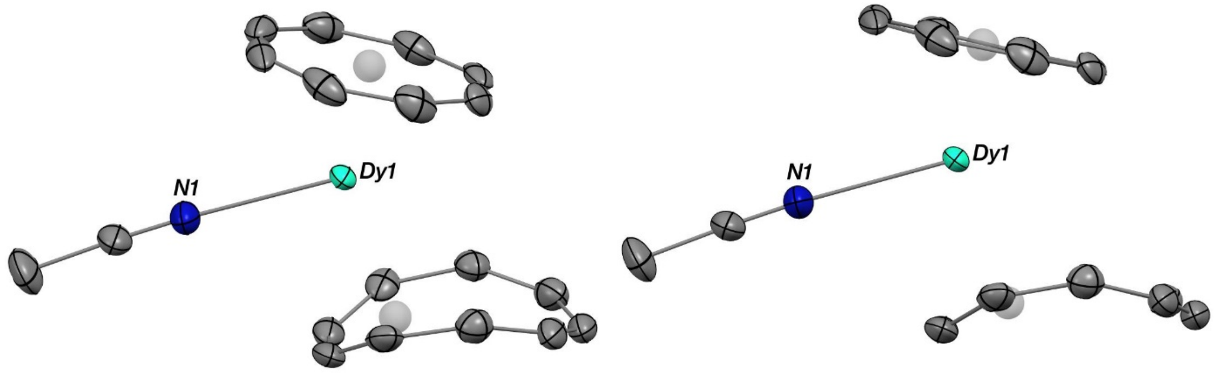


**Figure S19.** ORTEP of 1/2[Dy(Cot)(Cnt)];[Dy(Cot)(Cnt)(NCMe)] (**2b**) at 150 K. Thermal ellipsoids are depicted at 50 % probability level. Carbon atoms are in grey, nitrogen atom in blue, and dysprosium atom in light green. The 1/2[Dy(Cot)(Cnt)] part of the asymmetric unit, the cocrystallized DCM molecule and hydrogen atoms have been removed for clarity. The light grey centroids are for Ctr8 of the Cot and Ctr6 centroid of the Cnt.

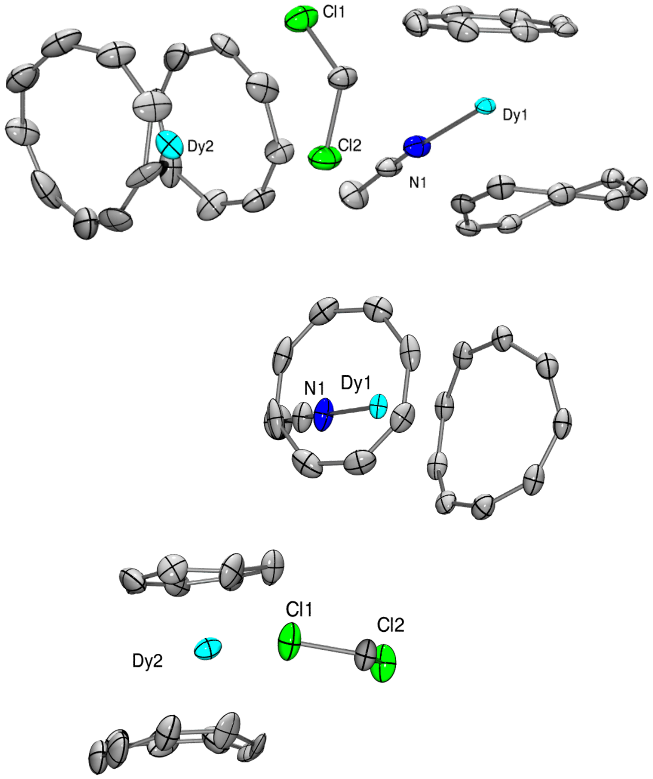


**Figure S20.** ORTEP of 1/2[Dy(Cot)(Cnt)];[Dy(Cot)(Cnt)(NCMe)] (**2b**) at 150 K. Thermal ellipsoids are depicted at 50 % probability level. Carbon atoms are in grey, nitrogen atom in blue, chloride atoms in green and dysprosium in light green. Full asymmetric unit is shown, hydrogen atoms have been removed for clarity.

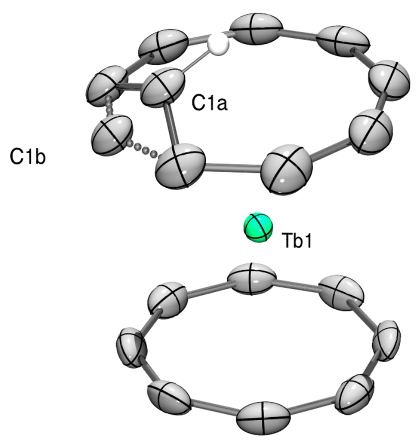


**Figure S21.** ORTEP of [Tb(*cis*-Cnt)(*trans*-Cnt)(Cot)] **(1')** at 150 K. Thermal ellipsoids are depicted at 50 % probability level. Carbon atoms are in grey, hydrogen atom in white, and terbium atom in light green. Part of the disordered carbons and non-essential hydrogen atoms have been removed for clarity.

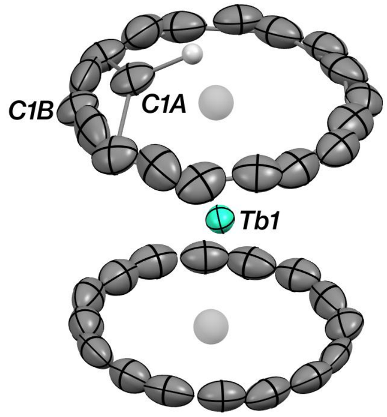


**Figure S22.** ORTEP of [Tb(Cot)(*cis*-Cnt)(*trans*-Cnt)] **(1'')** at 150 K. Thermal ellipsoids are depicted at 50 % probability level. Carbon atoms are in grey (centroid in light grey), hydrogen atom in white, and terbium atom in light green. Full disorder is shown, non-essential hydrogen atoms have been removed for clarity.

**Discussion of the crystal structure of 2b and 1'.**

During the synthesis of **2**, it is clearly important to further dry the compound cautiously. If this is not done properly, an MeCN adduct of **2** can be formed and crystallized from DCM. The important point to observe here is the way Cot and Cnt ligands behave. Without any disorder on the adduct part, it is easily identifiable that the Cnt ligand geometry is largely impacted by the MeCN adduct while the Cot ligands remains planar. The Ln-C distances range for the eight membered ring is 2.545(7)–2.596(5) Å, similar to that found in the base-free **2** (2.52(2)-2.639 (16) Å). For the nine membered ring the range is 2.694(6)-4.353(5) Å with a Ln-C(max)-Ln-C(min) difference of 1.659 Å which is of course larger than in **2** but also larger than the one observed in **6** (1.426 Å). As stated in an earlier publication, the Cnt ligand is strongly impacted by the coordination of coordinating solvents, such as acetonitrile.[12] On the other hand, the Cot remains with a similar coordination without any disorder. This indicates that the Cnt is more labile than the Cot ligand and is more likely to switch hapticity.

The structure of **1'** shows another type of disorder. As discussed in previous articles,[12-15] complexes of lanthanides with the Cnt ligand can be isolated as a mixture of *cis,cis,cis*,*cis* and *cis,cis,cis*,*trans* forms, which can be further isomerized in the *cis,cis,cis*,*cis* form by the use of MeCN as co-solvent of toluene in the reaction. However, in different reaction conditions (hot toluene synthesis), it is also possible to isolate a mixture of both *cis,cis,cis*,*cis* and *cis,cis,cis*,*trans* isomers. The crystals are then more deeply colored (red instead of pale yellow). In this structure, a position disorder is used to discriminate between both forms, leading to C1A, being moved inside the ring (*cis,cis,cis*,*trans*) and C1B, closing the aromatic ring for the *cis,cis,cis*,*cis* form. **1** and **1'** have different $^{1}H$ NMR spectra.

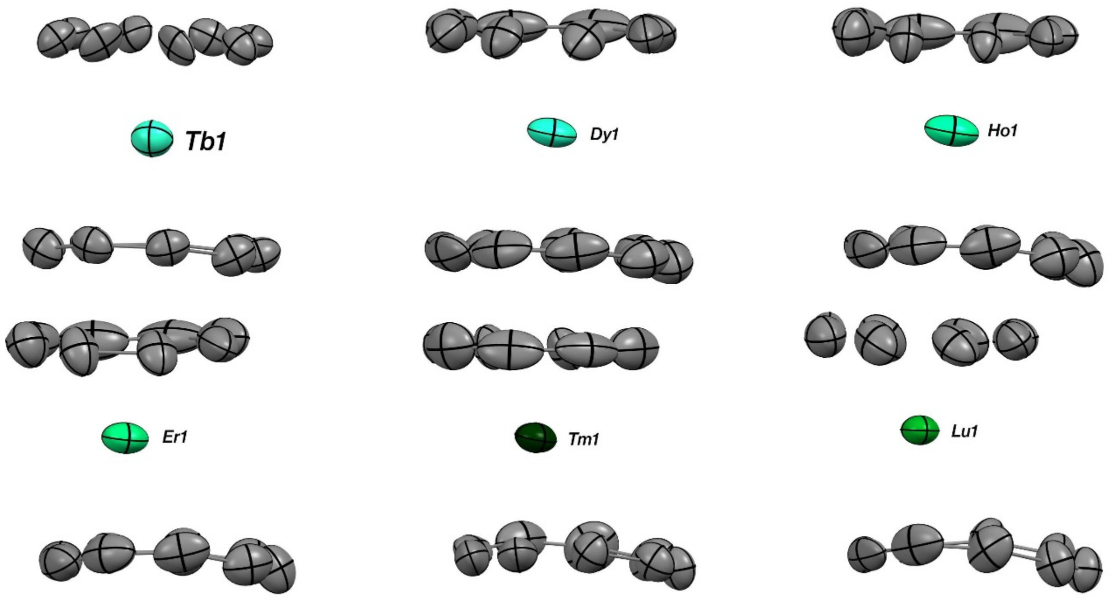


**Figure S23.** ORTEP of **1-6** at 300 K. Thermal ellipsoids are depicted at 50 % probability level. Carbon atoms are in grey, lanthanide atoms in several shades of green. Disordered positions and hydrogen atoms have been removed for clarity.

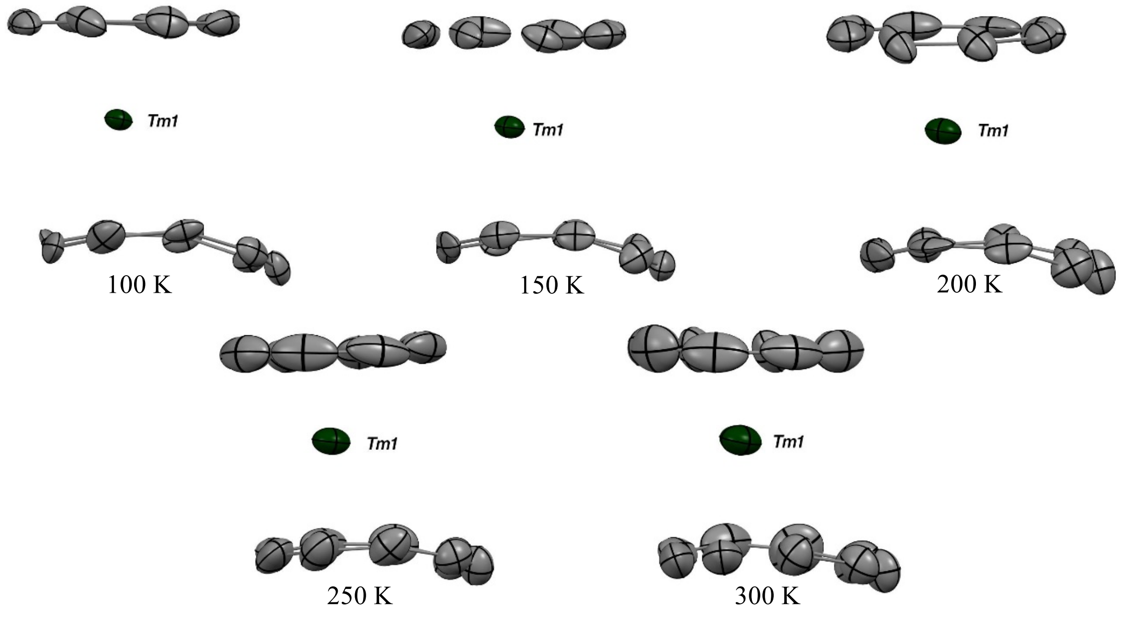


**Figure S24.** ORTEP of **5** from 100 K to 300 K. Thermal ellipsoids are depicted at 50 % probability level. Carbon atoms are in grey, thulium atom in dark green. Disordered positions and hydrogen atoms have been removed for clarity.

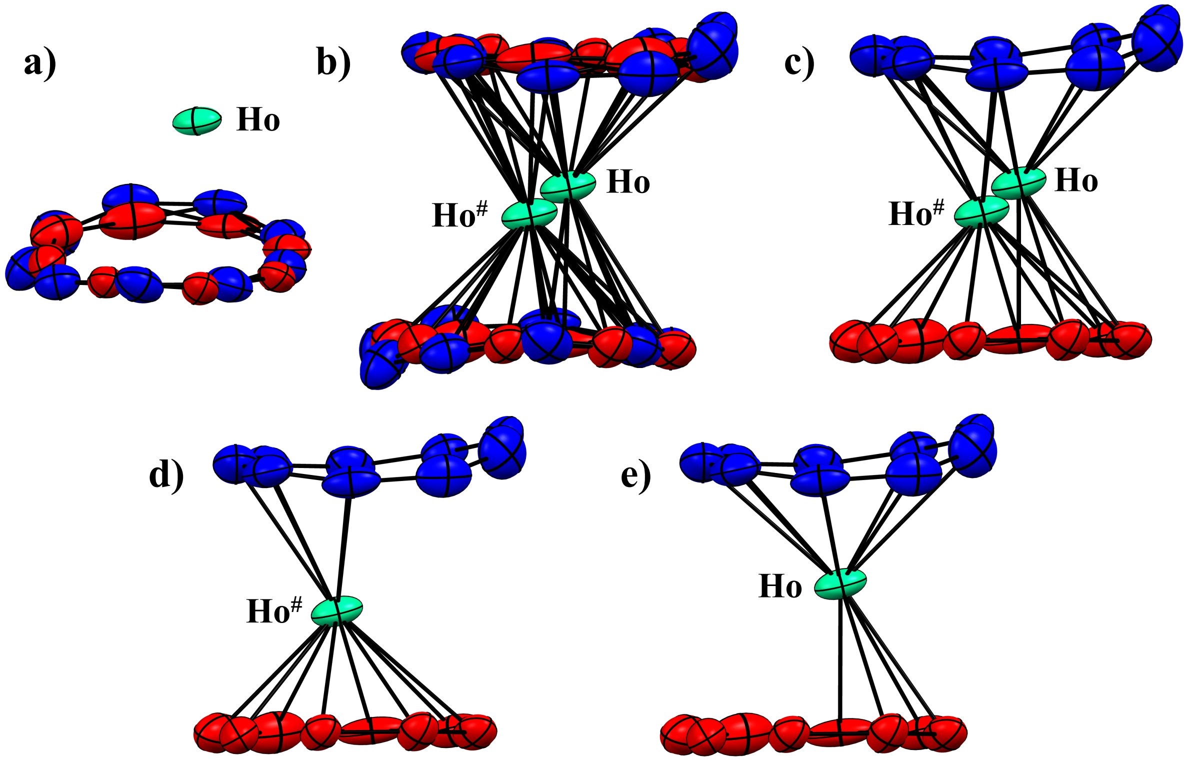


**Figure S25.** Crystallographic resolution of [Ho(Cnt)(Cot)]. a) Asymmetric unit with the Cnt (9-membered ring) in blue and the Cot (8-membered ring) in red. b) construction of the overall molecule by inversion symmetry. Ho# represents the symmetry equivalent of Ho; both blue and red rings are duplicated by symmetry. c) removal of the disorder: one blue ring (bottom) and one red ring (bottom). d) solution in which the Cnt is in lower coordination mode. e) solution in which the Cot is in lower coordination mode.

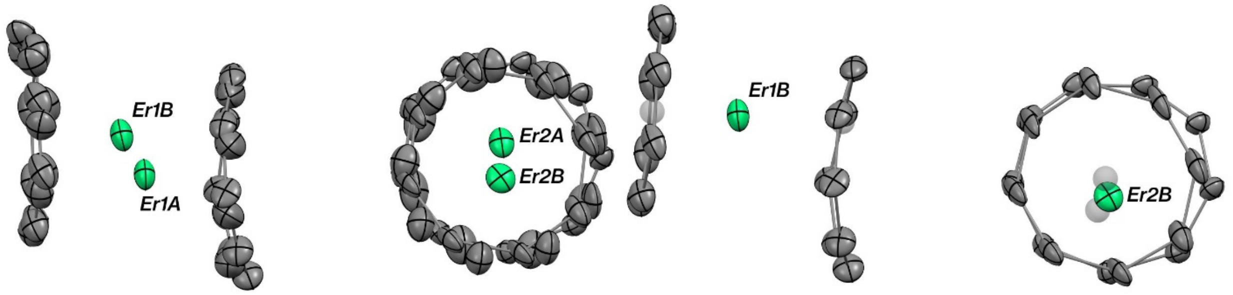


**Figure S26.** Structure of **4** solved in *P*1 at 150 K. Thermal ellipsoids are depicted at 50 % probability level. Carbon atoms are in grey, erbium atoms in green, hydrogen atoms have been removed for clarity. On the left full disorder is shown, on the right only half of the disorder is shown for clarity. The light grey centroid are for Ctr8 of the Cot and Ctr6 centroid of the Cnt.

**Table S8.** Main metric parameters for **1-6** at 150 and 300 K. The smallest distances and angles for each complex are given in red. a) The Tb parameters are the average distances and angles between part A and B.

| | **Tb (1)**[a)] | **Dy (2)** | **Ho (3)** | **Er (4)** | **Tm (5)** | **Lu (6)** |
|---|---|---|---|---|---|---|
| Plane angles | 4.80 | 12.5 | 23.7 | 29.7 | 31.3 | 34.2 |
| Ln-Ctr(6-C/Cnt) | 2.130 | 2.082 | 2.054 | 2.065 | 2.070 | 2.066 |
| Ln-Ctr(8-C/Cnt) | 1.979 | 1.997 | 2.102 | 2.193 | 2.197 | 2.239 |
| Ln-Ctr(9-C/Cnt) | 1.970 | 2.016 | 2.184 | 2.309 | 2.314 | 2.375 |
| Ln-Cot(Ctr) | 1.804 | 1.772 | 1.735 | 1.701 | 1.681 | 1.653 |
| Ln-Cot-(6-C/Cnt) | 157.5 | 161.0 | 169.6 | 174.7 | 173.8 | 174.2 |
| Ln-Cot-(8-C/Cnt) | 173.6 | 172.0 | 166.7 | 163.1 | 163.5 | 161.3 |
| Ln-Cot-(9-C/Cnt) | 177.4 | 170.5 | 161.4 | 157.6 | 158.1 | 156.0 |
| Ln-Ln[#] | 0 | 0.248 | 0.635 | 0.912 | 0.896 | 1.095 |
| | **1, 300 K** | **2, 300 K** | **3, 300 K** | **4, 300 K** | **5, 300 K** | **6, 300 K** |
| Plane angles | 3.2 | 14.8 | 15.0 | 17.7 | 19.8 | 25.3 |
| Ln-Ctr(6-C/Cnt) | 2.142 | 2.158 | 2.137 | 2.105 | 2.121 | 2.135 |
| Ln-Ctr(8-C/Cnt) | 1.985 | 2.072 | 2.117 | 2.182 | 2.203 | 2.278 |
| Ln-Ctr(9-C/Cnt) | 1.967 | 2.092 | 2.167 | 2.269 | 2.293 | 2.398 |
| Ln-Cot(Ctr) | 1.802 | 1.778 | 1.705 | 1.687 | 1.667 | 1.627 |
| Ln-Cot-(6-C/Cnt) | 154.3 | 160.3 | 165.0 | 174.0 | 168.6 | 171.9 |
| Ln-Cot-(8-C/Cnt) | 170.2 | 170.2 | 173.3 | 167.0 | 163.2 | 161.9 |
| Ln-Cot-(9-C/Cnt) | 177.4 | 169.4 | 169.2 | 161.3 | 158.5 | 156.8 |
| Ln-Ln[#] | 0 | 0.366 | 0.602 | 0.882 | 0.876 | 1.137 |

**Table S9.** Bond lengths (Å) and angles (deg) for [Tb(Cot)I(THF)$_2$]. (Continuing over several pages)

| Atom A | Atom B | Distance(esd) |
|---|---|---|
| Tb1 | I1 | 3.138(5) |
| Tb1 | C8 | 2.563(7) |
| Tb1 | C4 | 2.571(6) |
| Tb1 | C7 | 2.573(7) |
| Tb1 | C1 | 2.574(7) |
| Tb1 | C6 | 2.560(7) |
| Tb1 | C5 | 2.566(6) |
| Tb1 | C2 | 2.575(8) |
| Tb1 | O1 | 2.413(5) |
| Tb1 | O2 | 2.391(4) |
| Tb1 | I1A | 3.014(5) |
| Tb1 | C3 | 2.610(8) |
| C16 | O2 | 1.447(7) |
| C16 | C15 | 1.494(9) |
| C8 | C7 | 1.400(7) |
| C8 | C1 | 1.371(11) |
| C9 | O1 | 1.419(8) |
| C9 | C10 | 1.517(10) |
| C4 | C5 | 1.418(11) |
| C4 | C3 | 1.400(7) |
| C7 | C6 | 1.425(11) |
| C1 | C2 | 1.394(7) |
| C6 | C5 | 1.409(7) |
| C2 | C3 | 1.367(11) |
| C14 | C15 | 1.519(11) |
| C14 | C13 | 1.41(2) |
| C14 | C19 | 1.513(16) |
| C12 | O1 | 1.475(19) |
| C12 | C11 | 1.56(3) |
| O1 | C12A | 1.528(12) |
| O2 | C13 | 1.58(2) |
| O2 | C19 | 1.435(13) |
| C10 | C11A | 1.505(16) |
| C10 | C11 | 1.49(2) |
| C11A | C12A | 1.477(19) |

| Atom A | Atom B | Atom C | Angle(esd) |
|---|---|---|---|
| C8 | Tb1 | I1 | 96.1(2) |
| C8 | Tb1 | C4 | 90.6(2) |
| C8 | Tb1 | C7 | 31.64(17) |
| C8 | Tb1 | C1 | 31.0(3) |
| C8 | Tb1 | C5 | 82.9(2) |
| C8 | Tb1 | C2 | 59.6(2) |
| C8 | Tb1 | I1A | 98.8(2) |

| | | | |
|---|---|---|---|
| C8 | Tb1 | C3 | 81.1(3) |
| C4 | Tb1 | I1 | 144.2(2) |
| C4 | Tb1 | C7 | 82.8(2) |
| C4 | Tb1 | C1 | 81.5(2) |
| C4 | Tb1 | C2 | 59.3(2) |
| C4 | Tb1 | I1A | 145.60(19) |
| C4 | Tb1 | C3 | 31.35(16) |
| C7 | Tb1 | I1 | 84.80(18) |
| C7 | Tb1 | C1 | 59.8(2) |
| C7 | Tb1 | C2 | 81.3(3) |
| C7 | Tb1 | I1A | 88.15(18) |
| C7 | Tb1 | C3 | 90.2(2) |
| C1 | Tb1 | I1 | 120.0(2) |
| C1 | Tb1 | C2 | 31.41(17) |
| C1 | Tb1 | I1A | 121.8(2) |
| C1 | Tb1 | C3 | 59.5(2) |
| C6 | Tb1 | I1 | 92.07(19) |
| C6 | Tb1 | C8 | 61.0(2) |
| C6 | Tb1 | C4 | 60.9(2) |
| C6 | Tb1 | C7 | 32.2(2) |
| C6 | Tb1 | C1 | 82.0(3) |
| C6 | Tb1 | C5 | 31.91(16) |
| C6 | Tb1 | C2 | 90.0(2) |
| C6 | Tb1 | I1A | 95.15(19) |
| C6 | Tb1 | C3 | 82.1(2) |
| C5 | Tb1 | I1 | 114.1(2) |
| C5 | Tb1 | C4 | 32.1(2) |
| C5 | Tb1 | C7 | 61.3(2) |
| C5 | Tb1 | C1 | 90.7(2) |
| C5 | Tb1 | C2 | 81.6(2) |
| C5 | Tb1 | I1A | 116.3(2) |
| C5 | Tb1 | C3 | 60.6(2) |
| C2 | Tb1 | I1 | 150.4(2) |
| C2 | Tb1 | I1A | 151.5(2) |
| C2 | Tb1 | C3 | 30.6(3) |
| O1 | Tb1 | I1 | 87.20(16) |
| O1 | Tb1 | C8 | 175.60(19) |
| O1 | Tb1 | C4 | 85.0(2) |
| O1 | Tb1 | C7 | 146.6(2) |
| O1 | Tb1 | C1 | 147.9(2) |
| O1 | Tb1 | C6 | 116.2(2) |
| O1 | Tb1 | C5 | 93.1(2) |
| O1 | Tb1 | C2 | 118.1(2) |
| O1 | Tb1 | I1A | 84.60(16) |
| O1 | Tb1 | C3 | 95.3(2) |
| O2 | Tb1 | I1 | 89.35(16) |
| O2 | Tb1 | C8 | 104.5(2) |
| O2 | Tb1 | C4 | 122.9(2) |
| O2 | Tb1 | C7 | 133.8(2) |
| O2 | Tb1 | C1 | 84.7(2) |
| O2 | Tb1 | C6 | 165.4(2) |
| O2 | Tb1 | C5 | 154.9(2) |
| O2 | Tb1 | C2 | 81.7(2) |
| O2 | Tb1 | O1 | 78.36(17) |

| | | | |
|---|---|---|---|
| O2 | Tb1 | I1A | 86.72(16) |
| O2 | Tb1 | C3 | 96.3(2) |
| C3 | Tb1 | I1 | 174.1(2) |
| C3 | Tb1 | I1A | 176.84(19) |
| O2 | C16 | C15 | 105.2(6) |
| C7 | C8 | Tb1 | 74.5(4) |
| C1 | C8 | Tb1 | 75.0(5) |
| C1 | C8 | C7 | 135.7(8) |
| O1 | C9 | C10 | 105.9(6) |
| C5 | C4 | Tb1 | 73.8(4) |
| C3 | C4 | Tb1 | 75.8(4) |
| C3 | C4 | C5 | 135.6(8) |
| C8 | C7 | Tb1 | 73.8(4) |
| C8 | C7 | C6 | 133.9(7) |
| C6 | C7 | Tb1 | 73.4(4) |
| C8 | C1 | Tb1 | 74.1(4) |
| C8 | C1 | C2 | 134.8(8) |
| C2 | C1 | Tb1 | 74.3(4) |
| C7 | C6 | Tb1 | 74.4(4) |
| C5 | C6 | Tb1 | 74.3(4) |
| C5 | C6 | C7 | 135.2(7) |
| C4 | C5 | Tb1 | 74.2(4) |
| C6 | C5 | Tb1 | 73.8(4) |
| C6 | C5 | C4 | 133.8(7) |
| C1 | C2 | Tb1 | 74.3(4) |
| C3 | C2 | Tb1 | 76.1(5) |
| C3 | C2 | C1 | 137.3(8) |
| C13 | C14 | C15 | 103.1(11) |
| C19 | C14 | C15 | 105.7(7) |
| O1 | C12 | C11 | 94.6(15) |
| C9 | O1 | Tb1 | 126.9(4) |
| C9 | O1 | C12 | 108.3(9) |
| C9 | O1 | C12A | 106.9(6) |
| C12 | O1 | Tb1 | 122.0(8) |
| C12A | O1 | Tb1 | 124.4(5) |
| C16 | O2 | Tb1 | 125.9(4) |
| C16 | O2 | C13 | 106.7(9) |
| C13 | O2 | Tb1 | 119.5(9) |
| C19 | O2 | Tb1 | 126.2(6) |
| C19 | O2 | C16 | 107.8(7) |
| C16 | C15 | C14 | 104.7(6) |
| C11A | C10 | C9 | 105.6(7) |
| C11 | C10 | C9 | 102.4(10) |
| C12A | C11A | C10 | 100.1(10) |
| C11A | C12A | O1 | 103.4(10) |
| C10 | C11 | C12 | 102.8(17) |
| C14 | C13 | O2 | 104.2(14) |
| O2 | C19 | C14 | 106.3(9) |
| C4 | C3 | Tb1 | 72.8(4) |
| C2 | C3 | Tb1 | 73.3(5) |
| C2 | C3 | C4 | 133.7(8) |

**Table S10.** Bond lengths (Å) and angles (deg) for [Ho(Cot)I(THF)$_2$]. (Continuing over several pages).

| Atom A | Atom B | Distance(esd) |
|---|---|---|
| Ho(1) | O(2) | 2.381(5) |
| Ho(1) | O(4) | 2.362(5) |
| Ho(1) | C(1) | 2.558(8) |
| Ho(1) | C(2) | 2.545(8) |
| Ho(1) | C(3) | 2.546(9) |
| Ho(1) | C(4) | 2.556(9) |
| Ho(1) | C(5) | 2.579(10) |
| Ho(1) | C(6) | 2.556(8) |
| Ho(1) | C(7) | 2.540(7) |
| Ho(1) | C(8) | 2.538(7) |
| Ho(1) | I(2) | 3.107(6) |
| Ho(1) | I(1) | 2.990(5) |
| O(2) | C(16) | 1.422(9) |
| O(2) | C(13) | 1.47(2) |
| O(2) | C(13B) | 1.529(14) |
| O(4) | C(9) | 1.443(8) |
| O(4) | C(12) | 1.39(2) |
| O(4) | C(12B) | 1.522(17) |
| C(1) | C(2) | 1.401(10) |
| C(1) | C(8) | 1.424(13) |
| C(2) | C(3) | 1.357(13) |
| C(3) | C(4) | 1.389(11) |
| C(4) | C(5) | 1.358(13) |
| C(5) | C(6) | 1.408(11) |
| C(6) | C(7) | 1.417(13) |
| C(7) | C(8) | 1.423(11) |
| C(9) | C(10) | 1.499(11) |
| C(10) | C(11) | 1.507(12) |
| C(11) | C(12) | 1.58(2) |
| C(11) | C(12B) | 1.385(17) |
| C(15) | C(16) | 1.506(11) |
| C(15) | C(14) | 1.47(3) |
| C(15) | C(14B) | 1.524(18) |
| C(13) | C(14) | 1.53(4) |
| C(13B) | C(14B) | 1.47(2) |

| Atom A | Atom B | Atom C | Angle/˚ |
|---|---|---|---|
| O(2) | Ho(1) | C(1) | 146.1(3) |
| O(2) | Ho(1) | C(2) | 176.3(2) |
| O(2) | Ho(1) | C(3) | 148.8(3) |
| O(2) | Ho(1) | C(4) | 118.9(3) |
| O(2) | Ho(1) | C(5) | 95.7(3) |
| O(2) | Ho(1) | C(6) | 85.1(2) |
| O(2) | Ho(1) | C(7) | 92.6(2) |
| O(2) | Ho(1) | C(8) | 115.9(3) |
| O(2) | Ho(1) | I(2) | 86.99(17) |
| O(2) | Ho(1) | I(1) | 84.30(18) |
| O(4) | Ho(1) | O(2) | 78.50(19) |
| O(4) | Ho(1) | C(1) | 133.5(3) |
| O(4) | Ho(1) | C(2) | 104.4(2) |

| | | | |
|---|---|---|---|
| O(4) | Ho(1) | C(3) | 84.7(3) |
| O(4) | Ho(1) | C(4) | 81.9(3) |
| O(4) | Ho(1) | C(5) | 96.1(3) |
| O(4) | Ho(1) | C(6) | 123.0(3) |
| O(4) | Ho(1) | C(7) | 155.2(3) |
| O(4) | Ho(1) | C(8) | 165.6(3) |
| O(4) | Ho(1) | I(2) | 88.60(18) |
| O(4) | Ho(1) | I(1) | 86.25(18) |
| C(1) | Ho(1) | C(5) | 91.6(3) |
| C(1) | Ho(1) | I(2) | 83.9(2) |
| C(1) | Ho(1) | I(1) | 87.1(2) |
| C(2) | Ho(1) | C(1) | 31.9(2) |
| C(2) | Ho(1) | C(3) | 30.9(3) |
| C(2) | Ho(1) | C(4) | 59.7(3) |
| C(2) | Ho(1) | C(5) | 81.8(3) |
| C(2) | Ho(1) | C(6) | 91.3(3) |
| C(2) | Ho(1) | I(2) | 95.4(2) |
| C(2) | Ho(1) | I(1) | 98.1(2) |
| C(3) | Ho(1) | C(1) | 60.2(3) |
| C(3) | Ho(1) | C(4) | 31.6(3) |
| C(3) | Ho(1) | C(5) | 59.9(3) |
| C(3) | Ho(1) | C(6) | 82.2(3) |
| C(3) | Ho(1) | I(2) | 119.0(2) |
| C(3) | Ho(1) | I(1) | 120.9(2) |
| C(4) | Ho(1) | C(1) | 82.0(3) |
| C(4) | Ho(1) | C(5) | 30.7(3) |
| C(4) | Ho(1) | I(2) | 149.5(2) |
| C(4) | Ho(1) | I(1) | 150.8(2) |
| C(5) | Ho(1) | I(2) | 175.0(2) |
| C(5) | Ho(1) | I(1) | 177.6(2) |
| C(6) | Ho(1) | C(1) | 83.9(3) |
| C(6) | Ho(1) | C(4) | 59.7(3) |
| C(6) | Ho(1) | C(5) | 31.8(2) |
| C(6) | Ho(1) | I(2) | 144.8(2) |
| C(6) | Ho(1) | I(1) | 145.9(2) |
| C(7) | Ho(1) | C(1) | 62.3(3) |
| C(7) | Ho(1) | C(2) | 83.8(3) |
| C(7) | Ho(1) | C(3) | 91.7(3) |
| C(7) | Ho(1) | C(4) | 82.4(3) |
| C(7) | Ho(1) | C(5) | 61.4(3) |
| C(7) | Ho(1) | C(6) | 32.3(3) |
| C(7) | Ho(1) | I(2) | 114.3(2) |
| C(7) | Ho(1) | I(1) | 116.2(2) |
| C(8) | Ho(1) | C(1) | 32.4(3) |
| C(8) | Ho(1) | C(2) | 61.3(3) |
| C(8) | Ho(1) | C(3) | 82.5(3) |
| C(8) | Ho(1) | C(4) | 90.6(3) |
| C(8) | Ho(1) | C(5) | 83.2(3) |
| C(8) | Ho(1) | C(6) | 61.6(3) |
| C(8) | Ho(1) | C(7) | 32.6(2) |
| C(8) | Ho(1) | I(2) | 91.8(2) |
| C(8) | Ho(1) | I(1) | 94.7(2) |
| C(16) | O(2) | Ho(1) | 127.3(4) |
| C(16) | O(2) | C(13) | 107.2(10) |

| | | | |
|---|---|---|---|
| C(16) | O(2) | C(13B) | 106.3(7) |
| C(13) | O(2) | Ho(1) | 123.0(9) |
| C(13B) | O(2) | Ho(1) | 124.5(6) |
| C(9) | O(4) | Ho(1) | 126.3(4) |
| C(9) | O(4) | C(12B) | 106.9(8) |
| C(12) | O(4) | Ho(1) | 125.9(10) |
| C(12) | O(4) | C(9) | 107.8(11) |
| C(12B) | O(4) | Ho(1) | 121.6(7) |
| C(2) | C(1) | Ho(1) | 73.5(5) |
| C(2) | C(1) | C(8) | 132.9(8) |
| C(8) | C(1) | Ho(1) | 73.0(4) |
| C(1) | C(2) | Ho(1) | 74.6(5) |
| C(3) | C(2) | Ho(1) | 74.6(5) |
| C(3) | C(2) | C(1) | 136.2(9) |
| C(2) | C(3) | Ho(1) | 74.5(5) |
| C(2) | C(3) | C(4) | 135.3(9) |
| C(4) | C(3) | Ho(1) | 74.6(5) |
| C(3) | C(4) | Ho(1) | 73.8(5) |
| C(5) | C(4) | Ho(1) | 75.6(6) |
| C(5) | C(4) | C(3) | 137.3(9) |
| C(4) | C(5) | Ho(1) | 73.8(6) |
| C(4) | C(5) | C(6) | 133.7(10) |
| C(6) | C(5) | Ho(1) | 73.2(5) |
| C(5) | C(6) | Ho(1) | 75.0(5) |
| C(5) | C(6) | C(7) | 135.4(9) |
| C(7) | C(6) | Ho(1) | 73.2(4) |
| C(6) | C(7) | Ho(1) | 74.5(4) |
| C(6) | C(7) | C(8) | 133.4(8) |
| C(8) | C(7) | Ho(1) | 73.7(4) |
| C(1) | C(8) | Ho(1) | 74.5(4) |
| C(7) | C(8) | Ho(1) | 73.8(4) |
| C(7) | C(8) | C(1) | 135.8(8) |
| O(4) | C(9) | C(10) | 105.4(6) |
| C(9) | C(10) | C(11) | 104.3(7) |
| C(10) | C(11) | C(12) | 104.9(10) |
| C(12B) | C(11) | C(10) | 105.5(9) |
| C(16) | C(15) | C(14B) | 104.9(8) |
| C(14) | C(15) | C(16) | 101.2(11) |
| O(2) | C(16) | C(15) | 106.9(7) |
| O(4) | C(12) | C(11) | 104.3(15) |
| O(2) | C(13) | C(14) | 94.9(17) |
| C(15) | C(14) | C(13) | 105(2) |
| C(11) | C(12B) | O(4) | 107.7(11) |
| C(14B) | C(13B) | O(2) | 103.4(12) |
| C(13B) | C(14B) | C(15) | 100.2(11) |

**Table S11.** Bond lengths (Å) and angles (deg) for $[Lu(Cot)(BH_4)(THF)]_2$. (Continuing over several pages).

The 1 after atom name refers to the atom generated by the symmetry operation 1 applied on asymmetric unit atomic coordinates:
1-X,1-Y,1-Z

| Atom A | Atom B | Distance(esd) |
|---|---|---|
| Lu1 | O1 | 2.309(4) |
| Lu1 | C1 | 2.517(6) |
| Lu1 | C2 | 2.506(6) |
| Lu1 | C3 | 2.513(6) |
| Lu1 | C4 | 2.508(6) |
| Lu1 | C5 | 2.499(6) |
| Lu1 | C6 | 2.525(6) |
| Lu1 | C7 | 2.531(6) |
| Lu1 | C8 | 2.528(6) |
| Lu1 | B1 | 2.786(6) |
| Lu1 | $B1^1$ | 2.882(6) |
| O1 | C9 | 1.454(7) |
| O1 | C12 | 1.461(7) |
| C1 | C2 | 1.415(9) |
| C1 | C8 | 1.391(9) |
| C2 | C3 | 1.410(10) |
| C3 | C4 | 1.396(10) |
| C4 | C5 | 1.384(10) |
| C5 | C6 | 1.418(9) |
| C6 | C7 | 1.396(9) |
| C7 | C8 | 1.409(8) |
| C9 | C10 | 1.511(9) |
| C10 | C11 | 1.529(9) |
| C11 | C12 | 1.499(9) |

| Atom A | Atom B | Atom C | Angle(esd) |
|---|---|---|---|
| O1 | Lu1 | C1 | 157.20(18) |
| O1 | Lu1 | C2 | 124.85(19) |
| O1 | Lu1 | C3 | 97.51(19) |
| O1 | Lu1 | C4 | 82.04(18) |
| O1 | Lu1 | C5 | 85.23(17) |
| O1 | Lu1 | C6 | 105.55(18) |
| O1 | Lu1 | C7 | 134.87(18) |
| O1 | Lu1 | C8 | 167.18(18) |
| O1 | Lu1 | $B1^1$ | 79.20(17) |
| O1 | Lu1 | B1 | 86.75(18) |
| C1 | Lu1 | C6 | 84.7(2) |
| C1 | Lu1 | C7 | 61.7(2) |
| C1 | Lu1 | C8 | 32.0(2) |
| C1 | Lu1 | $B1^1$ | 120.6(2) |
| C1 | Lu1 | B1 | 86.1(2) |
| C2 | Lu1 | C1 | 32.7(2) |
| C2 | Lu1 | C3 | 32.6(2) |
| C2 | Lu1 | C4 | 62.2(2) |
| C2 | Lu1 | C6 | 93.7(2) |
| C2 | Lu1 | C7 | 84.22(19) |

| | | | |
|---|---|---|---|
| C2 | Lu1 | C8 | 61.8(2) |
| C2 | Lu1 | B1 | 84.1(2) |
| C2 | Lu1 | $B1^{1}$ | 150.2(2) |
| C3 | Lu1 | C1 | 62.6(2) |
| C3 | Lu1 | C6 | 84.7(2) |
| C3 | Lu1 | C7 | 93.05(19) |
| C3 | Lu1 | C8 | 84.5(2) |
| C3 | Lu1 | $B1^{1}$ | 176.7(2) |
| C3 | Lu1 | B1 | 100.2(2) |
| C4 | Lu1 | C1 | 85.1(2) |
| C4 | Lu1 | C3 | 32.3(2) |
| C4 | Lu1 | C6 | 62.2(2) |
| C4 | Lu1 | C7 | 84.2(2) |
| C4 | Lu1 | C8 | 93.4(2) |
| C4 | Lu1 | B1 | 127.3(2) |
| C4 | Lu1 | $B1^{1}$ | 146.0(2) |
| C5 | Lu1 | C1 | 93.8(2) |
| C5 | Lu1 | C2 | 84.5(2) |
| C5 | Lu1 | C3 | 61.7(2) |
| C5 | Lu1 | C4 | 32.1(2) |
| C5 | Lu1 | C6 | 32.8(2) |
| C5 | Lu1 | C7 | 61.9(2) |
| C5 | Lu1 | C8 | 84.64(19) |
| C5 | Lu1 | B1 | 158.9(2) |
| C5 | Lu1 | $B1^{1}$ | 117.7(2) |
| C6 | Lu1 | C7 | 32.1(2) |
| C6 | Lu1 | C8 | 61.9(2) |
| C6 | Lu1 | $B1^{1}$ | 96.11(19) |
| C6 | Lu1 | B1 | 166.2(2) |
| C7 | Lu1 | $B1^{1}$ | 89.29(18) |
| C7 | Lu1 | B1 | 134.2(2) |
| C8 | Lu1 | C7 | 32.34(19) |
| C8 | Lu1 | $B1^{1}$ | 98.7(2) |
| C8 | Lu1 | B1 | 105.4(2) |
| B1 | Lu1 | $B1^{1}$ | 79.70(18) |
| C9 | O1 | Lu1 | 128.1(3) |
| C9 | O1 | C12 | 104.2(4) |
| C12 | O1 | Lu1 | 126.6(3) |
| C2 | C1 | Lu1 | 73.2(3) |
| C8 | C1 | Lu1 | 74.4(3) |
| C8 | C1 | C2 | 134.2(6) |
| C1 | C2 | Lu1 | 74.1(3) |
| C3 | C2 | Lu1 | 74.0(4) |
| C3 | C2 | C1 | 135.4(6) |
| C2 | C3 | Lu1 | 73.4(3) |
| C4 | C3 | Lu1 | 73.7(4) |
| C4 | C3 | C2 | 134.7(6) |
| C3 | C4 | Lu1 | 74.0(4) |
| C5 | C4 | Lu1 | 73.6(4) |
| C5 | C4 | C3 | 135.0(6) |
| C4 | C5 | Lu1 | 74.3(4) |
| C4 | C5 | C6 | 136.1(6) |
| C6 | C5 | Lu1 | 74.6(3) |
| C5 | C6 | Lu1 | 72.6(3) |

| | | | |
|---|---|---|---|
| C7 | C6 | Lu1 | 74.2(3) |
| C7 | C6 | C5 | 133.5(6) |
| C6 | C7 | Lu1 | 73.8(3) |
| C6 | C7 | C8 | 135.9(6) |
| C8 | C7 | Lu1 | 73.7(3) |
| C1 | C8 | Lu1 | 73.6(3) |
| C1 | C8 | C7 | 135.1(6) |
| C7 | C8 | Lu1 | 73.9(3) |
| O1 | C9 | C10 | 104.7(5) |
| C9 | C10 | C11 | 104.7(5) |
| C12 | C11 | C10 | 104.9(5) |
| O1 | C12 | C11 | 104.3(5) |
| Lu1 | B1 | $Lu1^{1}$ | 100.29(18) |

**Table S12.** Bond lengths (Å) and angles (deg) for [Tb(Cnt)(Cot)] **(1)** at 150 K. (Continuing over several pages).

The 1 after atom name refers to the atom generated by the symmetry operation 1 applied on asymmetric unit atomic coordinates:
+X,-3/2-Y,+Z

| Atom A | Atom B | Distance(esd) |
|---|---|---|
| Tb(1) | C(1A) | 2.78(2) |
| Tb(1) | C(2A)1 | 2.839(16) |
| Tb(1) | C(2A) | 2.839(16) |
| Tb(1) | C(3A)1 | 2.874(15) |
| Tb(1) | C(3A) | 2.874(15) |
| Tb(1) | C(4A)1 | 2.852(16) |
| Tb(1) | C(4A) | 2.852(16) |
| Tb(1) | C(5A)1 | 2.780(14) |
| Tb(1) | C(5A) | 2.780(14) |
| Tb(1) | C(1B) | 2.82(4) |
| Tb(1) | C(2B)1 | 2.84(2) |
| Tb(1) | C(2B) | 2.84(2) |
| Tb(1) | C(3B) | 2.83(3) |
| Tb(1) | C(3B)1 | 2.83(3) |
| Tb(1) | C(4B)1 | 2.80(3) |
| Tb(1) | C(4B) | 2.80(3) |
| Tb(1) | C(5B)1 | 2.77(3) |
| Tb(1) | C(5B) | 2.77(3) |
| Tb(1) | C(6A) | 2.573(14) |
| Tb(1) | C(6A)1 | 2.573(14) |
| Tb(1) | C(7A)1 | 2.591(16) |
| Tb(1) | C(7A) | 2.591(16) |
| Tb(1) | C(8A)1 | 2.586(14) |
| Tb(1) | C(8A) | 2.586(14) |
| Tb(1) | C(9A) | 2.573(13) |
| Tb(1) | C(9A)1 | 2.573(13) |
| Tb(1) | C(6B) | 2.57(3) |
| Tb(1) | C(7B)1 | 2.63(2) |
| Tb(1) | C(7B) | 2.63(2) |
| Tb(1) | C(8B)1 | 2.56(2) |
| Tb(1) | C(8B) | 2.56(2) |
| Tb(1) | C(9B)1 | 2.58(2) |
| Tb(1) | C(9B) | 2.58(2) |
| Tb(1) | C(10B) | 2.60(3) |
| C(1A) | C(2A)1 | 1.397(12) |
| C(1A) | C(2A) | 1.398(12) |
| C(2A) | C(3A) | 1.386(12) |
| C(3A) | C(4A) | 1.384(12) |
| C(4A) | C(5A) | 1.387(12) |

| | | |
|---|---|---|
| C(5A) | C(5A)1 | 1.387(14) |
| C(1B) | C(2B) | 1.373(14) |
| C(1B) | C(2B)1 | 1.373(14) |
| C(2B) | C(3B) | 1.373(14) |
| C(3B) | C(4B) | 1.366(15) |
| C(4B) | C(5B) | 1.376(15) |
| C(5B) | C(5B)1 | 1.379(17) |
| C(6A) | C(6A)1 | 1.419(15) |
| C(6A) | C(7A) | 1.414(12) |
| C(7A) | C(8A) | 1.407(12) |
| C(8A) | C(9A) | 1.410(11) |
| C(9A) | C(9A)1 | 1.408(15) |
| C(6B) | C(7B) | 1.424(14) |
| C(6B) | C(7B)1 | 1.424(14) |
| C(7B) | C(8B) | 1.433(14) |
| C(8B) | C(9B) | 1.412(14) |
| C(9B) | C(10B) | 1.418(14) |

| Atom A | Atom B | Atom C | Angle(esd) |
|---|---|---|---|
| C(1A) | Tb(1) | C(2A)1 | 28.8(2) |
| C(1A) | Tb(1) | C(2A) | 28.8(2) |
| C(1A) | Tb(1) | C(3A)1 | 54.9(4) |
| C(1A) | Tb(1) | C(3A) | 54.9(4) |
| C(1A) | Tb(1) | C(4A) | 76.9(5) |
| C(1A) | Tb(1) | C(4A)1 | 76.9(5) |
| C(1A) | Tb(1) | C(2B)1 | 86.4(7) |
| C(1A) | Tb(1) | C(3B)1 | 68.1(6) |
| C(1A) | Tb(1) | C(4B)1 | 43.4(5) |
| C(2A)1 | Tb(1) | C(2A) | 55.1(5) |
| C(2A) | Tb(1) | C(3A) | 28.1(2) |
| C(2A)1 | Tb(1) | C(3A) | 76.2(4) |
| C(2A) | Tb(1) | C(3A)1 | 76.2(4) |
| C(2A)1 | Tb(1) | C(3A)1 | 28.1(2) |
| C(2A) | Tb(1) | C(4A)1 | 89.3(5) |
| C(2A)1 | Tb(1) | C(4A)1 | 54.4(3) |
| C(2A) | Tb(1) | C(4A) | 54.4(3) |
| C(2A)1 | Tb(1) | C(4A) | 89.3(5) |
| C(3A) | Tb(1) | C(3A)1 | 88.1(6) |
| C(4A)1 | Tb(1) | C(3A) | 88.4(4) |
| C(4A)1 | Tb(1) | C(3A)1 | 28.0(2) |
| C(4A) | Tb(1) | C(3A)1 | 88.4(4) |
| C(4A) | Tb(1) | C(3A) | 28.0(2) |
| C(4A)1 | Tb(1) | C(4A) | 75.8(6) |
| C(5A)1 | Tb(1) | C(1A) | 91.5(6) |
| C(5A) | Tb(1) | C(1A) | 91.5(6) |
| C(5A) | Tb(1) | C(2A)1 | 90.6(5) |
| C(5A)1 | Tb(1) | C(2A)1 | 77.3(4) |
| C(5A)1 | Tb(1) | C(2A) | 90.6(5) |
| C(5A) | Tb(1) | C(2A) | 77.3(4) |
| C(5A) | Tb(1) | C(3A)1 | 76.8(4) |
| C(5A) | Tb(1) | C(3A) | 54.8(3) |

| | | | |
|---|---|---|---|
| C(5A)1 | Tb(1) | C(3A)1 | 54.8(3) |
| C(5A)1 | Tb(1) | C(3A) | 76.8(4) |
| C(5A)1 | Tb(1) | C(4A) | 55.1(4) |
| C(5A)1 | Tb(1) | C(4A)1 | 28.5(2) |
| C(5A) | Tb(1) | C(4A) | 28.5(2) |
| C(5A) | Tb(1) | C(4A)1 | 55.1(4) |
| C(5A) | Tb(1) | C(5A)1 | 28.9(3) |
| C(5A) | Tb(1) | C(2B)1 | 42.1(4) |
| C(5A)1 | Tb(1) | C(2B)1 | 13.9(3) |
| C(5A) | Tb(1) | C(3B)1 | 66.4(5) |
| C(5A)1 | Tb(1) | C(3B)1 | 41.3(4) |
| C(5A)1 | Tb(1) | C(4B)1 | 65.8(5) |
| C(5A) | Tb(1) | C(4B)1 | 84.4(6) |
| C(1B) | Tb(1) | C(2B) | 28.1(3) |
| C(1B) | Tb(1) | C(3B) | 54.0(6) |
| C(3B) | Tb(1) | C(2B) | 28.0(3) |
| C(3B)1 | Tb(1) | C(2B) | 76.3(6) |
| C(3B)1 | Tb(1) | C(3B) | 89.4(12) |
| C(4B)1 | Tb(1) | C(1B) | 75.5(9) |
| C(4B) | Tb(1) | C(1B) | 75.5(9) |
| C(4B)1 | Tb(1) | C(2B) | 89.5(8) |
| C(4B) | Tb(1) | C(2B) | 54.3(5) |
| C(4B)1 | Tb(1) | C(3B) | 89.9(8) |
| C(4B) | Tb(1) | C(3B) | 28.1(3) |
| C(4B)1 | Tb(1) | C(4B) | 77.5(10) |
| C(5B)1 | Tb(1) | C(1B) | 88.4(11) |
| C(5B) | Tb(1) | C(1B) | 88.4(11) |
| C(5B)1 | Tb(1) | C(2B) | 89.5(8) |
| C(5B) | Tb(1) | C(2B) | 76.2(7) |
| C(5B) | Tb(1) | C(3B) | 54.7(6) |
| C(5B)1 | Tb(1) | C(3B) | 76.8(7) |
| C(5B)1 | Tb(1) | C(4B) | 55.5(5) |
| C(5B) | Tb(1) | C(4B) | 28.6(3) |
| C(5B)1 | Tb(1) | C(5B) | 28.9(4) |
| C(6A)1 | Tb(1) | C(6A) | 32.0(4) |
| C(6A) | Tb(1) | C(7A) | 31.8(3) |
| C(6A)1 | Tb(1) | C(7A) | 60.9(4) |
| C(6A)1 | Tb(1) | C(8A) | 82.8(4) |
| C(6A) | Tb(1) | C(8A) | 60.7(4) |
| C(6A)1 | Tb(1) | C(9B)1 | 71.6(5) |
| C(6A) | Tb(1) | C(9B)1 | 87.6(5) |
| C(7A)1 | Tb(1) | C(7A) | 82.4(7) |
| C(8A)1 | Tb(1) | C(7A) | 90.9(5) |
| C(8A) | Tb(1) | C(7A) | 31.5(3) |
| C(8A)1 | Tb(1) | C(8A) | 82.5(6) |
| C(9A) | Tb(1) | C(6A) | 82.8(4) |
| C(9A)1 | Tb(1) | C(6A) | 91.5(4) |
| C(9A)1 | Tb(1) | C(7A) | 82.4(4) |
| C(9A) | Tb(1) | C(7A) | 60.5(4) |
| C(9A)1 | Tb(1) | C(8A) | 60.7(4) |
| C(9A) | Tb(1) | C(8A) | 31.7(2) |
| C(9A) | Tb(1) | C(9B)1 | 46.6(4) |
| C(9A)1 | Tb(1) | C(9B)1 | 16.4(3) |
| C(6B) | Tb(1) | C(7B) | 31.8(3) |

| | | | |
|---|---|---|---|
| C(6B) | Tb(1) | C(9B) | 83.8(8) |
| C(6B) | Tb(1) | C(9B)1 | 83.8(8) |
| C(6B) | Tb(1) | C(10B) | 94.3(11) |
| C(8B) | Tb(1) | C(6B) | 61.1(6) |
| C(8B)1 | Tb(1) | C(6B) | 61.1(6) |
| C(8B)1 | Tb(1) | C(7B) | 82.0(6) |
| C(8B) | Tb(1) | C(7B) | 32.0(3) |
| C(8B)1 | Tb(1) | C(8B) | 89.9(9) |
| C(8B)1 | Tb(1) | C(9B) | 81.6(6) |
| C(8B)1 | Tb(1) | C(9B)1 | 31.9(3) |
| C(8B) | Tb(1) | C(9B) | 31.9(3) |
| C(8B) | Tb(1) | C(9B)1 | 81.6(6) |
| C(8B) | Tb(1) | C(10B) | 61.3(6) |
| C(8B)1 | Tb(1) | C(10B) | 61.3(6) |
| C(9B) | Tb(1) | C(7B) | 61.3(5) |
| C(9B)1 | Tb(1) | C(7B) | 91.1(7) |
| C(9B)1 | Tb(1) | C(9B) | 59.7(7) |
| C(9B)1 | Tb(1) | C(10B) | 31.8(3) |
| C(9B) | Tb(1) | C(10B) | 31.8(3) |
| C(10B) | Tb(1) | C(7B) | 84.5(8) |
| C(2A) | C(1A) | Tb(1) | 77.8(12) |
| C(2A)1 | C(1A) | Tb(1) | 77.8(12) |
| C(2A)1 | C(1A) | C(2A) | 140(3) |
| C(1A) | C(2A) | Tb(1) | 73.4(12) |
| C(3A) | C(2A) | Tb(1) | 77.4(9) |
| C(3A) | C(2A) | C(1A) | 139(2) |
| C(2A) | C(3A) | Tb(1) | 74.5(9) |
| C(4A) | C(3A) | Tb(1) | 75.1(9) |
| C(4A) | C(3A) | C(2A) | 140.0(16) |
| C(3A) | C(4A) | Tb(1) | 76.9(9) |
| C(3A) | C(4A) | C(5A) | 140.2(15) |
| C(5A) | C(4A) | Tb(1) | 72.9(9) |
| C(4A) | C(5A) | Tb(1) | 78.6(9) |
| C(5A)1 | C(5A) | Tb(1) | 75.56(16) |
| C(5A)1 | C(5A) | C(4A) | 139.8(8) |
| C(2B)1 | C(1B) | Tb(1) | 77(2) |
| C(2B) | C(1B) | Tb(1) | 77(2) |
| C(1B) | C(2B) | Tb(1) | 75(2) |
| C(3B) | C(2B) | Tb(1) | 75.6(15) |
| C(3B) | C(2B) | C(1B) | 138(3) |
| C(2B) | C(3B) | Tb(1) | 76.3(15) |
| C(4B) | C(3B) | Tb(1) | 74.8(15) |
| C(4B) | C(3B) | C(2B) | 140(3) |
| C(3B) | C(4B) | Tb(1) | 77.1(15) |
| C(3B) | C(4B) | C(5B) | 139(3) |
| C(5B) | C(4B) | Tb(1) | 74.2(16) |
| C(4B) | C(5B) | Tb(1) | 77.2(16) |
| C(5B)1 | C(5B) | Tb(1) | 75.6(2) |
| C(6A)1 | C(6A) | Tb(1) | 74.00(18) |
| C(7A) | C(6A) | Tb(1) | 74.8(9) |
| C(6A) | C(7A) | Tb(1) | 73.4(9) |
| C(8A) | C(7A) | Tb(1) | 74.0(9) |
| C(8A) | C(7A) | C(6A) | 135.1(15) |
| C(7A) | C(8A) | Tb(1) | 74.4(9) |

| | | | |
|---|---|---|---|
| C(7A) | C(8A) | C(9A) | 134.9(16) |
| C(9A) | C(8A) | Tb(1) | 73.6(8) |
| C(8A) | C(9A) | Tb(1) | 74.7(8) |
| C(9A)1 | C(9A) | Tb(1) | 74.11(17) |
| C(9A)1 | C(9A) | C(8A) | 135.2(8) |
| C(7B)1 | C(6B) | Tb(1) | 76.5(19) |
| C(7B) | C(6B) | Tb(1) | 76.5(19) |
| C(6B) | C(7B) | Tb(1) | 71.7(19) |
| C(6B) | C(7B) | C(8B) | 132(3) |
| C(8B) | C(7B) | Tb(1) | 71.4(13) |
| C(7B) | C(8B) | Tb(1) | 76.6(13) |
| C(9B) | C(8B) | Tb(1) | 74.7(12) |
| C(9B) | C(8B) | C(7B) | 138(2) |
| C(8B) | C(9B) | Tb(1) | 73.4(12) |
| C(8B) | C(9B) | C(10B) | 137(2) |
| C(10B) | C(9B) | Tb(1) | 74.8(14) |
| C(9B) | C(10B) | Tb(1) | 73.4(14) |
| C(9B)1 | C(10B) | Tb(1) | 73.4(14) |
| C(9B)1 | C(10B) | C(9B) | 130(3) |

**Table S13.** Bond lengths (Å) and angles (deg) for [Tb(Cnt)(Cot)] **(1)** at 300 K. (Continuing over several pages).

The 1 after atom name refers to the atom generated by the symmetry operation 1 applied on asymmetric unit atomic coordinates:
+X,-3/2-Y,+Z

| Atom A | Atom B | Distance(esd) |
|---|---|---|
| Tb(1) | C(1A) | 2.78(5) |
| Tb(1) | C(2A)1 | 2.90(3) |
| Tb(1) | C(2A) | 2.90(3) |
| Tb(1) | C(3A) | 2.88(3) |
| Tb(1) | C(3A)1 | 2.88(3) |
| Tb(1) | C(4A) | 2.90(3) |
| Tb(1) | C(4A)1 | 2.90(3) |
| Tb(1) | C(5A) | 2.80(3) |
| Tb(1) | C(5A)1 | 2.80(3) |
| Tb(1) | C(1B) | 2.77(5) |
| Tb(1) | C(2B)1 | 2.82(3) |
| Tb(1) | C(2B) | 2.82(3) |
| Tb(1) | C(3B) | 2.77(3) |
| Tb(1) | C(3B)1 | 2.77(3) |
| Tb(1) | C(4B) | 2.80(3) |
| Tb(1) | C(4B)1 | 2.80(3) |
| Tb(1) | C(5B) | 2.81(4) |
| Tb(1) | C(5B)1 | 2.81(4) |
| Tb(1) | C(6A) | 2.577(19) |
| Tb(1) | C(6A)1 | 2.577(19) |
| Tb(1) | C(7A)1 | 2.58(2) |
| Tb(1) | C(7A) | 2.58(2) |
| Tb(1) | C(8A)1 | 2.57(2) |
| Tb(1) | C(8A) | 2.57(2) |
| Tb(1) | C(9A) | 2.58(2) |
| Tb(1) | C(9A)1 | 2.58(2) |
| Tb(1) | C(6B) | 2.64(4) |
| Tb(1) | C(7B)1 | 2.56(3) |
| Tb(1) | C(7B) | 2.56(3) |
| Tb(1) | C(8B) | 2.61(3) |
| Tb(1) | C(8B)1 | 2.61(3) |
| Tb(1) | C(9B)1 | 2.61(3) |
| Tb(1) | C(9B) | 2.61(3) |
| Tb(1) | C(10B) | 2.62(4) |
| C(1A) | C(2A) | 1.399(19) |
| C(1A) | C(2A)1 | 1.399(19) |
| C(2A) | C(3A) | 1.393(19) |
| C(3A) | C(4A) | 1.395(18) |
| C(4A) | C(5A) | 1.394(19) |
| C(5A) | C(5A)1 | 1.39(2) |
| C(1B) | C(2B) | 1.357(19) |
| C(1B) | C(2B)1 | 1.357(19) |
| C(2B) | C(3B) | 1.364(19) |
| C(3B) | C(4B) | 1.360(19) |
| C(4B) | C(5B) | 1.37(2) |
| C(5B) | C(5B)1 | 1.36(2) |

| | | |
|---|---|---|
| C(6A) | C(6A)1 | 1.412(18) |
| C(6A) | C(7A) | 1.416(15) |
| C(7A) | C(8A) | 1.412(15) |
| C(8A) | C(9A) | 1.402(15) |
| C(9A) | C(9A)1 | 1.410(17) |
| C(6B) | C(7B) | 1.440(17) |
| C(6B) | C(7B)1 | 1.440(17) |
| C(7B) | C(8B) | 1.431(17) |
| C(8B) | C(9B) | 1.424(17) |
| C(9B) | C(10B) | 1.425(18) |

| Atom A | Atom B | Atom C | Angle(esd) |
|---|---|---|---|
| C(1A) | Tb(1) | C(2A) | 28.4(3) |
| C(1A) | Tb(1) | C(2A)1 | 28.4(3) |
| C(1A) | Tb(1) | C(3A) | 54.0(7) |
| C(1A) | Tb(1) | C(3A)1 | 54.0(7) |
| C(1A) | Tb(1) | C(4A)1 | 75.8(11) |
| C(1A) | Tb(1) | C(4A) | 75.8(11) |
| C(1A) | Tb(1) | C(5A)1 | 90.0(14) |
| C(1A) | Tb(1) | C(5A) | 90.0(14) |
| C(1A) | Tb(1) | C(2B)1 | 85.4(12) |
| C(1A) | Tb(1) | C(4B)1 | 41.6(7) |
| C(1A) | Tb(1) | C(5B)1 | 14.2(3) |
| C(2A)1 | Tb(1) | C(2A) | 55.1(8) |
| C(3A) | Tb(1) | C(2A) | 27.9(3) |
| C(3A)1 | Tb(1) | C(2A)1 | 27.9(3) |
| C(3A) | Tb(1) | C(2A)1 | 75.9(8) |
| C(3A)1 | Tb(1) | C(2A) | 75.9(8) |
| C(3A) | Tb(1) | C(3A)1 | 87.6(12) |
| C(3A)1 | Tb(1) | C(4A) | 87.9(9) |
| C(3A) | Tb(1) | C(4A) | 27.9(3) |
| C(3A) | Tb(1) | C(4A)1 | 87.9(9) |
| C(3A)1 | Tb(1) | C(4A)1 | 27.9(3) |
| C(4A) | Tb(1) | C(2A) | 54.3(5) |
| C(4A) | Tb(1) | C(2A)1 | 88.9(9) |
| C(4A)1 | Tb(1) | C(2A) | 88.9(9) |
| C(4A)1 | Tb(1) | C(2A)1 | 54.3(5) |
| C(4A) | Tb(1) | C(4A)1 | 75.4(10) |
| C(5A)1 | Tb(1) | C(2A)1 | 77.0(9) |
| C(5A) | Tb(1) | C(2A) | 77.0(9) |
| C(5A)1 | Tb(1) | C(2A) | 90.3(10) |
| C(5A) | Tb(1) | C(2A)1 | 90.3(10) |
| C(5A)1 | Tb(1) | C(3A)1 | 54.6(6) |
| C(5A) | Tb(1) | C(3A)1 | 76.4(8) |
| C(5A) | Tb(1) | C(3A) | 54.6(6) |
| C(5A)1 | Tb(1) | C(3A) | 76.4(8) |
| C(5A)1 | Tb(1) | C(4A)1 | 28.3(3) |
| C(5A)1 | Tb(1) | C(4A) | 54.8(6) |
| C(5A) | Tb(1) | C(4A)1 | 54.8(6) |
| C(5A) | Tb(1) | C(4A) | 28.3(3) |
| C(5A) | Tb(1) | C(5A)1 | 28.9(5) |
| C(5A)1 | Tb(1) | C(2B)1 | 13.9(4) |
| C(5A) | Tb(1) | C(2B)1 | 42.2(4) |
| C(5A) | Tb(1) | C(4B)1 | 83.8(9) |

| | | | |
|---|---|---|---|
| C(5A)1 | Tb(1) | C(4B)1 | 65.7(7) |
| C(5A) | Tb(1) | C(5B)1 | 91.7(10) |
| C(5A)1 | Tb(1) | C(5B)1 | 84.8(9) |
| C(1B) | Tb(1) | C(2B) | 28.0(4) |
| C(1B) | Tb(1) | C(4B) | 74.8(13) |
| C(1B) | Tb(1) | C(5B) | 88.7(16) |
| C(3B)1 | Tb(1) | C(1B) | 53.5(8) |
| C(3B) | Tb(1) | C(1B) | 53.5(8) |
| C(3B) | Tb(1) | C(2B) | 28.2(4) |
| C(3B)1 | Tb(1) | C(2B) | 76.2(10) |
| C(3B)1 | Tb(1) | C(3B) | 88.1(15) |
| C(3B)1 | Tb(1) | C(4B) | 88.0(12) |
| C(3B) | Tb(1) | C(4B) | 28.3(4) |
| C(3B)1 | Tb(1) | C(5B) | 76.3(10) |
| C(3B) | Tb(1) | C(5B) | 54.9(8) |
| C(4B) | Tb(1) | C(2B) | 54.8(7) |
| C(4B)1 | Tb(1) | C(2B) | 89.1(11) |
| C(4B)1 | Tb(1) | C(4B) | 74.9(14) |
| C(4B)1 | Tb(1) | C(5B) | 54.2(7) |
| C(4B) | Tb(1) | C(5B) | 28.3(4) |
| C(5B)1 | Tb(1) | C(2B) | 90.4(12) |
| C(5B) | Tb(1) | C(2B) | 77.5(11) |
| C(5B)1 | Tb(1) | C(5B) | 28.2(5) |
| C(6A)1 | Tb(1) | C(6A) | 31.8(4) |
| C(6A) | Tb(1) | C(7A) | 31.9(3) |
| C(6A)1 | Tb(1) | C(7A) | 60.7(5) |
| C(6A)1 | Tb(1) | C(9A) | 91.3(6) |
| C(6A) | Tb(1) | C(9A) | 82.7(6) |
| C(6A) | Tb(1) | C(9B)1 | 87.8(7) |
| C(6A)1 | Tb(1) | C(9B)1 | 72.1(6) |
| C(7A)1 | Tb(1) | C(7A) | 82.3(9) |
| C(7A)1 | Tb(1) | C(9A) | 82.2(6) |
| C(7A) | Tb(1) | C(9A) | 60.4(5) |
| C(7A)1 | Tb(1) | C(9B)1 | 46.0(5) |
| C(7A) | Tb(1) | C(9B)1 | 87.4(8) |
| C(8A)1 | Tb(1) | C(6A) | 83.1(6) |
| C(8A) | Tb(1) | C(6A) | 61.2(5) |
| C(8A)1 | Tb(1) | C(7A) | 91.2(7) |
| C(8A) | Tb(1) | C(7A) | 31.8(3) |
| C(8A)1 | Tb(1) | C(9A) | 60.6(5) |
| C(8A) | Tb(1) | C(9A) | 31.6(3) |
| C(8A) | Tb(1) | C(9B)1 | 71.9(6) |
| C(8A)1 | Tb(1) | C(9B)1 | 15.8(4) |
| C(9A)1 | Tb(1) | C(9B)1 | 15.8(4) |
| C(9A) | Tb(1) | C(9B)1 | 46.1(5) |
| C(7B)1 | Tb(1) | C(6B) | 32.1(4) |
| C(7B) | Tb(1) | C(6B) | 32.1(4) |
| C(7B)1 | Tb(1) | C(7B) | 60.2(10) |
| C(7B) | Tb(1) | C(8B) | 32.2(4) |
| C(7B)1 | Tb(1) | C(8B) | 82.1(9) |
| C(7B) | Tb(1) | C(9B)1 | 90.6(9) |
| C(7B) | Tb(1) | C(9B) | 61.1(6) |
| C(7B)1 | Tb(1) | C(9B)1 | 61.1(6) |
| C(7B)1 | Tb(1) | C(9B) | 90.6(9) |

| | | | |
|---|---|---|---|
| C(7B) | Tb(1) | C(10B) | 84.4(11) |
| C(7B)1 | Tb(1) | C(10B) | 84.4(11) |
| C(8B)1 | Tb(1) | C(6B) | 61.9(7) |
| C(8B) | Tb(1) | C(6B) | 61.9(7) |
| C(8B)1 | Tb(1) | C(8B) | 90.1(13) |
| C(8B)1 | Tb(1) | C(9B) | 81.2(8) |
| C(8B) | Tb(1) | C(9B) | 31.7(4) |
| C(8B) | Tb(1) | C(9B)1 | 81.2(8) |
| C(8B)1 | Tb(1) | C(9B)1 | 31.7(4) |
| C(8B) | Tb(1) | C(10B) | 61.2(7) |
| C(8B)1 | Tb(1) | C(10B) | 61.2(7) |
| C(9B) | Tb(1) | C(6B) | 84.4(10) |
| C(9B)1 | Tb(1) | C(6B) | 84.4(10) |
| C(9B)1 | Tb(1) | C(9B) | 59.1(9) |
| C(9B) | Tb(1) | C(10B) | 31.6(4) |
| C(9B)1 | Tb(1) | C(10B) | 31.6(4) |
| C(10B) | Tb(1) | C(6B) | 95.3(14) |
| C(2A) | C(1A) | Tb(1) | 81(3) |
| C(2A)1 | C(1A) | Tb(1) | 81(3) |
| C(2A) | C(1A) | C(2A)1 | 147(6) |
| C(1A) | C(2A) | Tb(1) | 71(3) |
| C(3A) | C(2A) | Tb(1) | 75.2(16) |
| C(3A) | C(2A) | C(1A) | 134(4) |
| C(2A) | C(3A) | Tb(1) | 76.9(16) |
| C(2A) | C(3A) | C(4A) | 143(3) |
| C(4A) | C(3A) | Tb(1) | 76.8(16) |
| C(3A) | C(4A) | Tb(1) | 75.2(16) |
| C(5A) | C(4A) | Tb(1) | 71.9(17) |
| C(5A) | C(4A) | C(3A) | 138(3) |
| C(4A) | C(5A) | Tb(1) | 79.9(17) |
| C(4A) | C(5A) | C(5A)1 | 140.5(15) |
| C(5A)1 | C(5A) | Tb(1) | 75.6(2) |
| C(2B)1 | C(1B) | Tb(1) | 78(2) |
| C(2B) | C(1B) | Tb(1) | 78(2) |
| C(1B) | C(2B) | Tb(1) | 74(3) |
| C(1B) | C(2B) | C(3B) | 133(4) |
| C(3B) | C(2B) | Tb(1) | 73.6(17) |
| C(2B) | C(3B) | Tb(1) | 78.2(17) |
| C(4B) | C(3B) | Tb(1) | 77.4(17) |
| C(4B) | C(3B) | C(2B) | 144(3) |
| C(3B) | C(4B) | Tb(1) | 74.4(17) |
| C(3B) | C(4B) | C(5B) | 141(3) |
| C(5B) | C(4B) | Tb(1) | 76(2) |
| C(4B) | C(5B) | Tb(1) | 75.8(19) |
| C(5B)1 | C(5B) | Tb(1) | 75.9(2) |
| C(5B)1 | C(5B) | C(4B) | 138.3(18) |
| C(6A)1 | C(6A) | Tb(1) | 74.1(2) |
| C(6A)1 | C(6A) | C(7A) | 134.5(10) |
| C(7A) | C(6A) | Tb(1) | 74.2(11) |
| C(6A) | C(7A) | Tb(1) | 73.9(11) |
| C(8A) | C(7A) | Tb(1) | 73.7(11) |
| C(8A) | C(7A) | C(6A) | 135.6(19) |
| C(7A) | C(8A) | Tb(1) | 74.5(11) |
| C(9A) | C(8A) | Tb(1) | 74.7(11) |

| | | | |
|---|---|---|---|
| C(9A) | C(8A) | C(7A) | 134.5(19) |
| C(8A) | C(9A) | Tb(1) | 73.7(11) |
| C(9A)1 | C(9A) | Tb(1) | 74.2(2) |
| C(7B)1 | C(6B) | Tb(1) | 71(2) |
| C(7B) | C(6B) | Tb(1) | 71(2) |
| C(7B)1 | C(6B) | C(7B) | 126(4) |
| C(6B) | C(7B) | Tb(1) | 77(2) |
| C(8B) | C(7B) | Tb(1) | 75.7(16) |
| C(8B) | C(7B) | C(6B) | 140(3) |
| C(7B) | C(8B) | Tb(1) | 72.1(16) |
| C(9B) | C(8B) | Tb(1) | 74.4(17) |
| C(9B) | C(8B) | C(7B) | 134(3) |
| C(8B) | C(9B) | Tb(1) | 74.0(17) |
| C(8B) | C(9B) | C(10B) | 138(3) |
| C(10B) | C(9B) | Tb(1) | 75(2) |
| C(9B)1 | C(10B) | Tb(1) | 74(2) |
| C(9B) | C(10B) | Tb(1) | 74(2) |
| C(9B)1 | C(10B) | C(9B) | 129(4) |

**Table S14.** Bond lengths (Å) and angles (deg) for [Dy(Cnt)(Cot)] **(2)** at 150 K. (Continuing over several pages).

| Atom A | Atom B | Distance(esd) |
|---|---|---|
| Dy(1) | C(1) | 2.675(17) |
| Dy(1) | C(2) | 2.723(16) |
| Dy(1) | C(3) | 2.738(16) |
| Dy(1) | C(4) | 2.787(15) |
| Dy(1) | C(5) | 2.860(18) |
| Dy(1) | C(6) | 2.953(18) |
| Dy(1) | C(7) | 2.959(17) |
| Dy(1) | C(8) | 2.857(16) |
| Dy(1) | C(9) | 2.736(16) |
| Dy(1) | C(10) | 2.52(2) |
| Dy(1) | C(11) | 2.560(16) |
| Dy(1) | C(12) | 2.611(16) |
| Dy(1) | C(13) | 2.639(16) |
| Dy(1) | C(14) | 2.600(16) |
| Dy(1) | C(15) | 2.615(15) |
| Dy(1) | C(16) | 2.590(15) |
| Dy(1) | C(17) | 2.536(17) |
| C(1) | C(2) | 1.366(13) |
| C(1) | C(9) | 1.371(13) |
| C(2) | C(3) | 1.381(12) |
| C(3) | C(4) | 1.365(12) |
| C(4) | C(5) | 1.294(13) |
| C(5) | C(6) | 1.258(12) |
| C(6) | C(7) | 1.333(12) |
| C(7) | C(8) | 1.349(12) |
| C(8) | C(9) | 1.364(12) |
| C(10) | C(11) | 1.484(13) |
| C(10) | C(17) | 1.479(12) |
| C(11) | C(12) | 1.438(12) |
| C(12) | C(13) | 1.417(11) |
| C(13) | C(14) | 1.429(11) |
| C(14) | C(15) | 1.429(12) |
| C(15) | C(16) | 1.423(12) |
| C(16) | C(17) | 1.425(11) |

| Atom A | Atom B | Atom C | Angle(esd) |
|---|---|---|---|
| C(1) | Dy(1) | C(2) | 29.3(3) |
| C(1) | Dy(1) | C(3) | 56.4(4) |
| C(1) | Dy(1) | C(4) | 78.0(5) |
| C(1) | Dy(1) | C(5) | 89.3(5) |
| C(1) | Dy(1) | C(6) | 86.2(5) |
| C(1) | Dy(1) | C(7) | 73.5(5) |
| C(1) | Dy(1) | C(8) | 54.3(4) |
| C(1) | Dy(1) | C(9) | 29.3(3) |
| C(2) | Dy(1) | C(3) | 29.3(3) |
| C(2) | Dy(1) | C(4) | 56.0(4) |
| C(2) | Dy(1) | C(5) | 76.1(4) |
| C(2) | Dy(1) | C(6) | 85.1(4) |
| C(2) | Dy(1) | C(7) | 84.9(4) |
| C(2) | Dy(1) | C(8) | 75.6(5) |

| | | | |
|---|---|---|---|
| C(2) | Dy(1) | C(9) | 56.6(4) |
| C(3) | Dy(1) | C(4) | 28.6(3) |
| C(3) | Dy(1) | C(5) | 53.2(4) |
| C(3) | Dy(1) | C(6) | 70.8(4) |
| C(3) | Dy(1) | C(7) | 82.9(4) |
| C(3) | Dy(1) | C(8) | 87.0(5) |
| C(4) | Dy(1) | C(5) | 26.5(3) |
| C(4) | Dy(1) | C(6) | 49.0(4) |
| C(4) | Dy(1) | C(7) | 69.6(4) |
| C(4) | Dy(1) | C(8) | 85.4(5) |
| C(5) | Dy(1) | C(6) | 24.9(3) |
| C(5) | Dy(1) | C(7) | 49.8(4) |
| C(6) | Dy(1) | C(7) | 26.1(3) |
| C(8) | Dy(1) | C(5) | 72.6(4) |
| C(8) | Dy(1) | C(6) | 51.7(4) |
| C(8) | Dy(1) | C(7) | 26.7(2) |
| C(9) | Dy(1) | C(3) | 78.4(5) |
| C(9) | Dy(1) | C(4) | 90.4(5) |
| C(9) | Dy(1) | C(5) | 88.9(5) |
| C(9) | Dy(1) | C(6) | 74.4(4) |
| C(9) | Dy(1) | C(7) | 53.0(4) |
| C(9) | Dy(1) | C(8) | 28.1(3) |
| C(10) | Dy(1) | C(1) | 95.8(6) |
| C(10) | Dy(1) | C(2) | 109.2(6) |
| C(10) | Dy(1) | C(3) | 131.4(6) |
| C(10) | Dy(1) | C(4) | 156.9(7) |
| C(10) | Dy(1) | C(5) | 174.7(6) |
| C(10) | Dy(1) | C(6) | 153.7(7) |
| C(10) | Dy(1) | C(7) | 130.4(6) |
| C(10) | Dy(1) | C(8) | 109.2(5) |
| C(10) | Dy(1) | C(9) | 94.7(6) |
| C(10) | Dy(1) | C(11) | 33.9(3) |
| C(10) | Dy(1) | C(12) | 62.8(4) |
| C(10) | Dy(1) | C(13) | 83.7(4) |
| C(10) | Dy(1) | C(14) | 92.3(5) |
| C(10) | Dy(1) | C(15) | 84.1(5) |
| C(10) | Dy(1) | C(16) | 62.9(4) |
| C(10) | Dy(1) | C(17) | 34.0(3) |
| C(11) | Dy(1) | C(1) | 109.2(6) |
| C(11) | Dy(1) | C(2) | 133.9(5) |
| C(11) | Dy(1) | C(3) | 162.5(6) |
| C(11) | Dy(1) | C(4) | 168.4(6) |
| C(11) | Dy(1) | C(5) | 142.2(6) |
| C(11) | Dy(1) | C(6) | 121.0(6) |
| C(11) | Dy(1) | C(7) | 103.1(6) |
| C(11) | Dy(1) | C(8) | 91.4(5) |
| C(11) | Dy(1) | C(9) | 92.1(6) |
| C(11) | Dy(1) | C(12) | 32.3(3) |
| C(11) | Dy(1) | C(13) | 60.9(4) |
| C(11) | Dy(1) | C(14) | 83.6(4) |
| C(11) | Dy(1) | C(15) | 93.7(5) |
| C(11) | Dy(1) | C(16) | 86.9(5) |
| C(12) | Dy(1) | C(1) | 133.1(6) |
| C(12) | Dy(1) | C(2) | 162.4(5) |

| | | | |
|---|---|---|---|
| C(12) | Dy(1) | C(3) | 165.1(5) |
| C(12) | Dy(1) | C(4) | 136.5(5) |
| C(12) | Dy(1) | C(5) | 112.3(6) |
| C(12) | Dy(1) | C(6) | 96.9(6) |
| C(12) | Dy(1) | C(7) | 89.3(5) |
| C(12) | Dy(1) | C(8) | 91.8(5) |
| C(12) | Dy(1) | C(9) | 107.0(6) |
| C(12) | Dy(1) | C(13) | 31.3(3) |
| C(12) | Dy(1) | C(15) | 83.6(5) |
| C(13) | Dy(1) | C(1) | 160.7(6) |
| C(13) | Dy(1) | C(2) | 165.2(5) |
| C(13) | Dy(1) | C(3) | 135.9(5) |
| C(13) | Dy(1) | C(4) | 109.4(5) |
| C(13) | Dy(1) | C(5) | 90.9(5) |
| C(13) | Dy(1) | C(6) | 85.9(5) |
| C(13) | Dy(1) | C(7) | 92.1(5) |
| C(13) | Dy(1) | C(8) | 107.5(6) |
| C(13) | Dy(1) | C(9) | 131.4(6) |
| C(14) | Dy(1) | C(1) | 166.2(6) |
| C(14) | Dy(1) | C(2) | 137.0(6) |
| C(14) | Dy(1) | C(3) | 110.1(5) |
| C(14) | Dy(1) | C(4) | 90.3(5) |
| C(14) | Dy(1) | C(5) | 83.0(5) |
| C(14) | Dy(1) | C(6) | 91.4(5) |
| C(14) | Dy(1) | C(7) | 109.3(5) |
| C(14) | Dy(1) | C(8) | 132.5(6) |
| C(14) | Dy(1) | C(9) | 160.4(6) |
| C(14) | Dy(1) | C(12) | 60.6(4) |
| C(14) | Dy(1) | C(13) | 31.6(3) |
| C(14) | Dy(1) | C(15) | 31.8(3) |
| C(15) | Dy(1) | C(1) | 138.4(6) |
| C(15) | Dy(1) | C(2) | 112.1(6) |
| C(15) | Dy(1) | C(3) | 93.1(5) |
| C(15) | Dy(1) | C(4) | 86.2(5) |
| C(15) | Dy(1) | C(5) | 93.2(5) |
| C(15) | Dy(1) | C(6) | 111.6(5) |
| C(15) | Dy(1) | C(7) | 135.6(5) |
| C(15) | Dy(1) | C(8) | 162.1(5) |
| C(15) | Dy(1) | C(9) | 167.6(6) |
| C(15) | Dy(1) | C(13) | 60.9(4) |
| C(16) | Dy(1) | C(1) | 113.3(6) |
| C(16) | Dy(1) | C(2) | 95.6(6) |
| C(16) | Dy(1) | C(3) | 90.5(5) |
| C(16) | Dy(1) | C(4) | 98.8(6) |
| C(16) | Dy(1) | C(5) | 116.4(5) |
| C(16) | Dy(1) | C(6) | 139.6(5) |
| C(16) | Dy(1) | C(7) | 165.7(5) |
| C(16) | Dy(1) | C(8) | 166.0(5) |
| C(16) | Dy(1) | C(9) | 138.0(6) |
| C(16) | Dy(1) | C(12) | 94.1(4) |
| C(16) | Dy(1) | C(13) | 83.7(4) |
| C(16) | Dy(1) | C(14) | 61.1(4) |
| C(16) | Dy(1) | C(15) | 31.7(3) |
| C(17) | Dy(1) | C(1) | 96.7(6) |

| | | | |
|---|---|---|---|
| C(17) | Dy(1) | C(2) | 93.6(6) |
| C(17) | Dy(1) | C(3) | 104.3(6) |
| C(17) | Dy(1) | C(4) | 123.9(6) |
| C(17) | Dy(1) | C(5) | 146.9(6) |
| C(17) | Dy(1) | C(6) | 171.7(6) |
| C(17) | Dy(1) | C(7) | 162.1(5) |
| C(17) | Dy(1) | C(8) | 135.9(5) |
| C(17) | Dy(1) | C(9) | 111.7(6) |
| C(17) | Dy(1) | C(11) | 65.4(4) |
| C(17) | Dy(1) | C(12) | 86.8(5) |
| C(17) | Dy(1) | C(13) | 93.6(5) |
| C(17) | Dy(1) | C(14) | 83.9(5) |
| C(17) | Dy(1) | C(15) | 61.3(4) |
| C(17) | Dy(1) | C(16) | 32.3(3) |
| C(2) | C(1) | Dy(1) | 77.3(9) |
| C(2) | C(1) | C(9) | 141.9(16) |
| C(9) | C(1) | Dy(1) | 77.8(9) |
| C(1) | C(2) | Dy(1) | 73.4(9) |
| C(1) | C(2) | C(3) | 137.1(14) |
| C(3) | C(2) | Dy(1) | 75.9(8) |
| C(2) | C(3) | Dy(1) | 74.8(8) |
| C(4) | C(3) | Dy(1) | 77.7(8) |
| C(4) | C(3) | C(2) | 140.6(14) |
| C(3) | C(4) | Dy(1) | 73.7(8) |
| C(5) | C(4) | Dy(1) | 79.9(9) |
| C(5) | C(4) | C(3) | 141.2(14) |
| C(4) | C(5) | Dy(1) | 73.6(8) |
| C(6) | C(5) | Dy(1) | 81.7(10) |
| C(6) | C(5) | C(4) | 138.3(14) |
| C(5) | C(6) | Dy(1) | 73.4(10) |
| C(5) | C(6) | C(7) | 142.0(14) |
| C(7) | C(6) | Dy(1) | 77.2(9) |
| C(6) | C(7) | Dy(1) | 76.7(9) |
| C(6) | C(7) | C(8) | 141.7(15) |
| C(8) | C(7) | Dy(1) | 72.4(8) |
| C(7) | C(8) | Dy(1) | 80.9(9) |
| C(7) | C(8) | C(9) | 140.0(15) |
| C(9) | C(8) | Dy(1) | 71.0(8) |
| C(1) | C(9) | Dy(1) | 72.9(9) |
| C(8) | C(9) | Dy(1) | 80.9(8) |
| C(8) | C(9) | C(1) | 135.5(16) |
| C(11) | C(10) | Dy(1) | 74.3(8) |
| C(17) | C(10) | Dy(1) | 73.4(9) |
| C(17) | C(10) | C(11) | 136.5(12) |
| C(10) | C(11) | Dy(1) | 71.8(8) |
| C(12) | C(11) | Dy(1) | 75.8(8) |
| C(12) | C(11) | C(10) | 132.9(12) |
| C(11) | C(12) | Dy(1) | 71.9(8) |
| C(13) | C(12) | Dy(1) | 75.4(7) |
| C(13) | C(12) | C(11) | 134.8(13) |
| C(12) | C(13) | Dy(1) | 73.2(7) |
| C(12) | C(13) | C(14) | 135.0(13) |
| C(14) | C(13) | Dy(1) | 72.7(8) |
| C(13) | C(14) | Dy(1) | 75.7(8) |

| | | | |
|---|---|---|---|
| C(13) | C(14) | C(15) | 137.3(13) |
| C(15) | C(14) | Dy(1) | 74.7(9) |
| C(14) | C(15) | Dy(1) | 73.5(8) |
| C(16) | C(15) | Dy(1) | 73.1(7) |
| C(16) | C(15) | C(14) | 135.2(13) |
| C(15) | C(16) | Dy(1) | 75.1(7) |
| C(15) | C(16) | C(17) | 134.3(12) |
| C(17) | C(16) | Dy(1) | 71.8(8) |
| C(10) | C(17) | Dy(1) | 72.6(9) |
| C(16) | C(17) | Dy(1) | 76.0(7) |
| C(16) | C(17) | C(10) | 133.7(12) |

**Table S15.** Bond lengths (Å) and angles (deg) for [Dy(Cnt)(Cot)] **(2)** at 300 K. (Continuing over several pages).

| Atom A | Atom B | Distance(esd) |
|---|---|---|
| Dy(1) | C(1) | 2.806(11) |
| Dy(1) | C(2) | 2.799(9) |
| Dy(1) | C(3) | 2.828(10) |
| Dy(1) | C(4) | 2.938(11) |
| Dy(1) | C(5) | 3.017(12) |
| Dy(1) | C(6) | 2.953(11) |
| Dy(1) | C(7) | 2.847(11) |
| Dy(1) | C(8) | 2.797(16) |
| Dy(1) | C(9) | 2.844(11) |
| Dy(1) | C(10) | 2.607(9) |
| Dy(1) | C(11) | 2.605(9) |
| Dy(1) | C(12) | 2.564(8) |
| Dy(1) | C(13) | 2.553(10) |
| Dy(1) | C(14) | 2.457(13) |
| Dy(1) | C(15) | 2.480(17) |
| Dy(1) | C(16) | 2.534(11) |
| Dy(1) | C(17) | 2.585(10) |
| C(1) | C(2) | 1.364(11) |
| C(1) | C(9) | 1.329(11) |
| C(2) | C(3) | 1.385(12) |
| C(3) | C(4) | 1.383(12) |
| C(4) | C(5) | 1.363(11) |
| C(5) | C(6) | 1.361(10) |
| C(6) | C(7) | 1.351(10) |
| C(7) | C(8) | 1.315(11) |
| C(8) | C(9) | 1.299(11) |
| C(10) | C(11) | 1.415(10) |
| C(10) | C(17) | 1.403(10) |
| C(11) | C(12) | 1.413(10) |
| C(12) | C(13) | 1.422(11) |
| C(13) | C(14) | 1.425(11) |
| C(14) | C(15) | 1.461(12) |
| C(15) | C(16) | 1.469(13) |
| C(16) | C(17) | 1.417(11) |

| Atom A | Atom B | Atom C | Angle(esd) |
|---|---|---|---|
| C(1) | Dy(1) | C(3) | 54.5(3) |
| C(1) | Dy(1) | C(4) | 74.5(3) |
| C(1) | Dy(1) | C(5) | 84.5(3) |
| C(1) | Dy(1) | C(6) | 84.0(3) |
| C(1) | Dy(1) | C(7) | 72.8(3) |
| C(1) | Dy(1) | C(9) | 27.2(2) |
| C(2) | Dy(1) | C(1) | 28.2(2) |
| C(2) | Dy(1) | C(3) | 28.5(2) |
| C(2) | Dy(1) | C(4) | 53.8(3) |
| C(2) | Dy(1) | C(5) | 73.0(3) |
| C(2) | Dy(1) | C(6) | 84.9(3) |
| C(2) | Dy(1) | C(7) | 86.1(3) |
| C(2) | Dy(1) | C(9) | 53.3(3) |
| C(3) | Dy(1) | C(4) | 27.7(2) |

| | | | |
|---|---|---|---|
| C(3) | Dy(1) | C(5) | 52.0(3) |
| C(3) | Dy(1) | C(6) | 72.6(3) |
| C(3) | Dy(1) | C(7) | 86.2(3) |
| C(3) | Dy(1) | C(9) | 74.5(4) |
| C(4) | Dy(1) | C(5) | 26.4(2) |
| C(4) | Dy(1) | C(6) | 51.3(3) |
| C(6) | Dy(1) | C(5) | 26.3(2) |
| C(7) | Dy(1) | C(4) | 72.8(3) |
| C(7) | Dy(1) | C(5) | 51.6(3) |
| C(7) | Dy(1) | C(6) | 26.9(2) |
| C(8) | Dy(1) | C(1) | 52.2(3) |
| C(8) | Dy(1) | C(2) | 74.0(3) |
| C(8) | Dy(1) | C(3) | 86.7(3) |
| C(8) | Dy(1) | C(4) | 85.3(3) |
| C(8) | Dy(1) | C(5) | 71.8(3) |
| C(8) | Dy(1) | C(6) | 51.5(3) |
| C(8) | Dy(1) | C(7) | 26.9(2) |
| C(8) | Dy(1) | C(9) | 26.6(2) |
| C(9) | Dy(1) | C(4) | 85.3(4) |
| C(9) | Dy(1) | C(5) | 83.2(3) |
| C(9) | Dy(1) | C(6) | 71.1(3) |
| C(9) | Dy(1) | C(7) | 51.5(3) |
| C(10) | Dy(1) | C(1) | 94.4(3) |
| C(10) | Dy(1) | C(2) | 112.7(3) |
| C(10) | Dy(1) | C(3) | 138.4(3) |
| C(10) | Dy(1) | C(4) | 166.1(2) |
| C(10) | Dy(1) | C(5) | 163.6(2) |
| C(10) | Dy(1) | C(6) | 137.3(3) |
| C(10) | Dy(1) | C(7) | 112.4(3) |
| C(10) | Dy(1) | C(8) | 94.5(3) |
| C(10) | Dy(1) | C(9) | 88.3(4) |
| C(11) | Dy(1) | C(1) | 111.7(3) |
| C(11) | Dy(1) | C(2) | 137.5(3) |
| C(11) | Dy(1) | C(3) | 165.9(3) |
| C(11) | Dy(1) | C(4) | 161.3(3) |
| C(11) | Dy(1) | C(5) | 134.9(3) |
| C(11) | Dy(1) | C(6) | 110.7(3) |
| C(11) | Dy(1) | C(7) | 91.8(3) |
| C(11) | Dy(1) | C(8) | 85.2(3) |
| C(11) | Dy(1) | C(9) | 93.3(3) |
| C(11) | Dy(1) | C(10) | 31.5(2) |
| C(12) | Dy(1) | C(1) | 137.2(3) |
| C(12) | Dy(1) | C(2) | 165.3(3) |
| C(12) | Dy(1) | C(3) | 160.8(3) |
| C(12) | Dy(1) | C(4) | 133.1(3) |
| C(12) | Dy(1) | C(5) | 109.7(3) |
| C(12) | Dy(1) | C(6) | 92.1(3) |
| C(12) | Dy(1) | C(7) | 84.8(3) |
| C(12) | Dy(1) | C(8) | 92.9(3) |
| C(12) | Dy(1) | C(9) | 112.1(3) |
| C(12) | Dy(1) | C(10) | 60.8(3) |
| C(12) | Dy(1) | C(11) | 31.7(2) |
| C(12) | Dy(1) | C(17) | 83.5(3) |
| C(13) | Dy(1) | C(1) | 167.4(3) |

| | | | |
|---|---|---|---|
| C(13) | Dy(1) | C(2) | 161.3(2) |
| C(13) | Dy(1) | C(3) | 132.9(3) |
| C(13) | Dy(1) | C(4) | 109.2(3) |
| C(13) | Dy(1) | C(5) | 94.0(3) |
| C(13) | Dy(1) | C(6) | 89.0(3) |
| C(13) | Dy(1) | C(7) | 96.4(4) |
| C(13) | Dy(1) | C(8) | 115.4(4) |
| C(13) | Dy(1) | C(9) | 140.2(4) |
| C(13) | Dy(1) | C(10) | 83.5(3) |
| C(13) | Dy(1) | C(11) | 61.1(3) |
| C(13) | Dy(1) | C(12) | 32.3(3) |
| C(13) | Dy(1) | C(17) | 93.5(3) |
| C(14) | Dy(1) | C(1) | 159.4(4) |
| C(14) | Dy(1) | C(2) | 131.9(4) |
| C(14) | Dy(1) | C(3) | 108.1(4) |
| C(14) | Dy(1) | C(4) | 93.8(4) |
| C(14) | Dy(1) | C(5) | 92.4(4) |
| C(14) | Dy(1) | C(6) | 102.0(4) |
| C(14) | Dy(1) | C(7) | 120.5(5) |
| C(14) | Dy(1) | C(8) | 145.2(5) |
| C(14) | Dy(1) | C(9) | 171.8(5) |
| C(14) | Dy(1) | C(10) | 94.2(4) |
| C(14) | Dy(1) | C(11) | 84.9(4) |
| C(14) | Dy(1) | C(12) | 62.8(4) |
| C(14) | Dy(1) | C(13) | 33.0(3) |
| C(14) | Dy(1) | C(15) | 34.4(3) |
| C(14) | Dy(1) | C(16) | 65.8(4) |
| C(14) | Dy(1) | C(17) | 86.6(4) |
| C(15) | Dy(1) | C(1) | 128.4(4) |
| C(15) | Dy(1) | C(2) | 107.0(4) |
| C(15) | Dy(1) | C(3) | 94.6(3) |
| C(15) | Dy(1) | C(4) | 95.9(4) |
| C(15) | Dy(1) | C(5) | 109.1(3) |
| C(15) | Dy(1) | C(6) | 129.1(4) |
| C(15) | Dy(1) | C(7) | 153.3(4) |
| C(15) | Dy(1) | C(8) | 178.8(4) |
| C(15) | Dy(1) | C(9) | 153.8(5) |
| C(15) | Dy(1) | C(10) | 84.4(4) |
| C(15) | Dy(1) | C(11) | 93.5(3) |
| C(15) | Dy(1) | C(12) | 86.0(4) |
| C(15) | Dy(1) | C(13) | 63.9(4) |
| C(15) | Dy(1) | C(16) | 34.0(3) |
| C(15) | Dy(1) | C(17) | 62.6(4) |
| C(16) | Dy(1) | C(1) | 102.5(4) |
| C(16) | Dy(1) | C(2) | 92.4(3) |
| C(16) | Dy(1) | C(3) | 96.1(3) |
| C(16) | Dy(1) | C(4) | 112.3(3) |
| C(16) | Dy(1) | C(5) | 135.0(3) |
| C(16) | Dy(1) | C(6) | 160.5(3) |
| C(16) | Dy(1) | C(7) | 172.3(4) |
| C(16) | Dy(1) | C(8) | 145.6(5) |
| C(16) | Dy(1) | C(9) | 122.0(4) |
| C(16) | Dy(1) | C(10) | 61.2(3) |
| C(16) | Dy(1) | C(11) | 84.2(3) |

| | | | |
|---|---|---|---|
| C(16) | Dy(1) | C(12) | 95.1(3) |
| C(16) | Dy(1) | C(13) | 87.5(4) |
| C(16) | Dy(1) | C(17) | 32.1(3) |
| C(17) | Dy(1) | C(1) | 91.1(3) |
| C(17) | Dy(1) | C(2) | 96.3(3) |
| C(17) | Dy(1) | C(3) | 113.8(3) |
| C(17) | Dy(1) | C(4) | 138.3(3) |
| C(17) | Dy(1) | C(5) | 164.6(2) |
| C(17) | Dy(1) | C(6) | 167.4(3) |
| C(17) | Dy(1) | C(7) | 140.5(3) |
| C(17) | Dy(1) | C(8) | 116.7(4) |
| C(17) | Dy(1) | C(9) | 99.5(4) |
| C(17) | Dy(1) | C(10) | 31.3(2) |
| C(17) | Dy(1) | C(11) | 60.4(3) |
| C(2) | C(1) | Dy(1) | 75.7(6) |
| C(9) | C(1) | Dy(1) | 78.0(6) |
| C(9) | C(1) | C(2) | 140.1(10) |
| C(1) | C(2) | Dy(1) | 76.2(6) |
| C(1) | C(2) | C(3) | 139.3(10) |
| C(3) | C(2) | Dy(1) | 76.9(6) |
| C(2) | C(3) | Dy(1) | 74.6(6) |
| C(4) | C(3) | Dy(1) | 80.6(6) |
| C(4) | C(3) | C(2) | 139.8(9) |
| C(3) | C(4) | Dy(1) | 71.7(6) |
| C(5) | C(4) | Dy(1) | 80.0(6) |
| C(5) | C(4) | C(3) | 138.6(10) |
| C(4) | C(5) | Dy(1) | 73.6(7) |
| C(6) | C(5) | Dy(1) | 74.2(6) |
| C(6) | C(5) | C(4) | 138.6(11) |
| C(5) | C(6) | Dy(1) | 79.4(7) |
| C(7) | C(6) | Dy(1) | 72.2(6) |
| C(7) | C(6) | C(5) | 140.9(10) |
| C(6) | C(7) | Dy(1) | 81.0(7) |
| C(8) | C(7) | Dy(1) | 74.4(8) |
| C(8) | C(7) | C(6) | 139.9(9) |
| C(7) | C(8) | Dy(1) | 78.6(8) |
| C(9) | C(8) | Dy(1) | 78.7(8) |
| C(9) | C(8) | C(7) | 142.1(10) |
| C(1) | C(9) | Dy(1) | 74.8(6) |
| C(8) | C(9) | Dy(1) | 74.7(8) |
| C(8) | C(9) | C(1) | 139.7(10) |
| C(11) | C(10) | Dy(1) | 74.2(5) |
| C(17) | C(10) | Dy(1) | 73.5(5) |
| C(17) | C(10) | C(11) | 135.8(9) |
| C(10) | C(11) | Dy(1) | 74.3(5) |
| C(12) | C(11) | Dy(1) | 72.6(5) |
| C(12) | C(11) | C(10) | 135.6(9) |
| C(11) | C(12) | Dy(1) | 75.7(5) |
| C(11) | C(12) | C(13) | 135.4(9) |
| C(13) | C(12) | Dy(1) | 73.4(5) |
| C(12) | C(13) | Dy(1) | 74.3(5) |
| C(12) | C(13) | C(14) | 133.8(9) |
| C(14) | C(13) | Dy(1) | 69.8(6) |
| C(13) | C(14) | Dy(1) | 77.2(6) |

| | | | |
|---|---|---|---|
| C(13) | C(14) | C(15) | 134.7(10) |
| C(15) | C(14) | Dy(1) | 73.7(9) |
| C(14) | C(15) | Dy(1) | 71.9(8) |
| C(14) | C(15) | C(16) | 135.6(10) |
| C(16) | C(15) | Dy(1) | 75.0(7) |
| C(15) | C(16) | Dy(1) | 71.0(8) |
| C(17) | C(16) | Dy(1) | 75.9(5) |
| C(17) | C(16) | C(15) | 131.8(9) |
| C(10) | C(17) | Dy(1) | 75.2(5) |
| C(10) | C(17) | C(16) | 136.6(9) |
| C(16) | C(17) | Dy(1) | 72.0(6) |

**Table S16.** Bond lengths (Å) and angles (deg) for [Ho(Cnt)(Cot)] **(3)** at 150 K. (Continuing over several pages).

| Atom A | Atom B | Distance(esd) |
|---|---|---|
| Ho(1) | C(1) | 2.56(2) |
| Ho(1) | C(2) | 2.61(2) |
| Ho(1) | C(3) | 2.821(16) |
| Ho(1) | C(4) | 3.212(14) |
| Ho(1) | C(5) | 3.466(17) |
| Ho(1) | C(6) | 3.37(2) |
| Ho(1) | C(7) | 3.02(3) |
| Ho(1) | C(8) | 2.72(3) |
| Ho(1) | C(9) | 2.62(2) |
| Ho(1) | C(10) | 2.493(15) |
| Ho(1) | C(11) | 2.519(14) |
| Ho(1) | C(12) | 2.580(16) |
| Ho(1) | C(13) | 2.652(18) |
| Ho(1) | C(14) | 2.61(2) |
| Ho(1) | C(15) | 2.55(3) |
| Ho(1) | C(16) | 2.47(4) |
| Ho(1) | C(17) | 2.464(19) |
| C(1) | C(2) | 1.370(13) |
| C(1) | C(9) | 1.369(13) |
| C(2) | C(3) | 1.378(13) |
| C(3) | C(4) | 1.379(12) |
| C(4) | C(5) | 1.376(12) |
| C(5) | C(6) | 1.361(12) |
| C(6) | C(7) | 1.338(13) |
| C(7) | C(8) | 1.344(13) |
| C(8) | C(9) | 1.355(13) |
| C(10) | C(11) | 1.395(12) |
| C(10) | C(17) | 1.420(13) |
| C(11) | C(12) | 1.421(12) |
| C(12) | C(13) | 1.418(12) |
| C(13) | C(14) | 1.419(12) |
| C(14) | C(15) | 1.434(13) |
| C(15) | C(16) | 1.443(13) |
| C(16) | C(17) | 1.429(14) |

| Atom A | Atom B | Atom C | Angle(esd) |
|---|---|---|---|
| C(1) | Ho(1) | C(2) | 30.7(3) |
| C(1) | Ho(1) | C(3) | 57.2(5) |
| C(1) | Ho(1) | C(4) | 73.0(5) |
| C(1) | Ho(1) | C(5) | 79.2(5) |
| C(1) | Ho(1) | C(6) | 80.6(6) |
| C(1) | Ho(1) | C(7) | 75.0(7) |
| C(1) | Ho(1) | C(8) | 57.4(5) |
| C(1) | Ho(1) | C(9) | 30.6(3) |
| C(1) | Ho(1) | C(12) | 141.6(5) |
| C(1) | Ho(1) | C(13) | 162.7(8) |
| C(1) | Ho(1) | C(14) | 156.8(5) |
| C(2) | Ho(1) | C(3) | 29.1(3) |
| C(2) | Ho(1) | C(4) | 51.3(4) |
| C(2) | Ho(1) | C(5) | 67.4(5) |

| | | | |
|---|---|---|---|
| C(2) | Ho(1) | C(6) | 80.3(6) |
| C(2) | Ho(1) | C(7) | 86.9(7) |
| C(2) | Ho(1) | C(8) | 79.8(6) |
| C(2) | Ho(1) | C(9) | 59.1(5) |
| C(2) | Ho(1) | C(13) | 150.6(5) |
| C(3) | Ho(1) | C(4) | 25.4(3) |
| C(3) | Ho(1) | C(5) | 47.0(3) |
| C(3) | Ho(1) | C(6) | 66.9(4) |
| C(3) | Ho(1) | C(7) | 83.4(5) |
| C(4) | Ho(1) | C(5) | 23.4(2) |
| C(4) | Ho(1) | C(6) | 45.8(3) |
| C(6) | Ho(1) | C(5) | 22.9(2) |
| C(7) | Ho(1) | C(4) | 66.7(4) |
| C(7) | Ho(1) | C(5) | 45.4(3) |
| C(7) | Ho(1) | C(6) | 23.4(3) |
| C(8) | Ho(1) | C(3) | 89.4(6) |
| C(8) | Ho(1) | C(4) | 82.1(5) |
| C(8) | Ho(1) | C(5) | 65.9(5) |
| C(8) | Ho(1) | C(6) | 47.4(5) |
| C(8) | Ho(1) | C(7) | 26.4(4) |
| C(9) | Ho(1) | C(3) | 79.5(6) |
| C(9) | Ho(1) | C(4) | 84.9(5) |
| C(9) | Ho(1) | C(5) | 78.8(6) |
| C(9) | Ho(1) | C(6) | 68.3(6) |
| C(9) | Ho(1) | C(7) | 53.0(6) |
| C(9) | Ho(1) | C(8) | 29.3(3) |
| C(9) | Ho(1) | C(13) | 135.0(6) |
| C(10) | Ho(1) | C(1) | 104.5(5) |
| C(10) | Ho(1) | C(2) | 123.4(5) |
| C(10) | Ho(1) | C(3) | 147.8(4) |
| C(10) | Ho(1) | C(4) | 172.7(3) |
| C(10) | Ho(1) | C(5) | 163.8(3) |
| C(10) | Ho(1) | C(6) | 141.2(4) |
| C(10) | Ho(1) | C(7) | 119.6(5) |
| C(10) | Ho(1) | C(8) | 102.5(6) |
| C(10) | Ho(1) | C(9) | 96.3(6) |
| C(10) | Ho(1) | C(11) | 32.3(3) |
| C(10) | Ho(1) | C(12) | 61.9(4) |
| C(10) | Ho(1) | C(13) | 84.0(5) |
| C(10) | Ho(1) | C(14) | 93.7(6) |
| C(10) | Ho(1) | C(15) | 86.5(6) |
| C(11) | Ho(1) | C(1) | 119.3(5) |
| C(11) | Ho(1) | C(2) | 148.1(5) |
| C(11) | Ho(1) | C(3) | 176.1(7) |
| C(11) | Ho(1) | C(4) | 154.8(3) |
| C(11) | Ho(1) | C(5) | 132.3(4) |
| C(11) | Ho(1) | C(6) | 111.5(4) |
| C(11) | Ho(1) | C(7) | 94.1(5) |
| C(11) | Ho(1) | C(8) | 87.0(5) |
| C(11) | Ho(1) | C(9) | 96.6(5) |
| C(11) | Ho(1) | C(12) | 32.3(3) |
| C(11) | Ho(1) | C(13) | 61.2(4) |
| C(11) | Ho(1) | C(14) | 83.7(5) |
| C(11) | Ho(1) | C(15) | 94.2(6) |

| | | | |
|---|---|---|---|
| C(12) | Ho(1) | C(2) | 168.4(8) |
| C(12) | Ho(1) | C(3) | 149.3(4) |
| C(12) | Ho(1) | C(4) | 124.4(4) |
| C(12) | Ho(1) | C(5) | 105.3(4) |
| C(12) | Ho(1) | C(6) | 89.9(5) |
| C(12) | Ho(1) | C(7) | 81.7(6) |
| C(12) | Ho(1) | C(8) | 89.1(5) |
| C(12) | Ho(1) | C(9) | 111.6(5) |
| C(12) | Ho(1) | C(13) | 31.4(3) |
| C(12) | Ho(1) | C(14) | 60.3(4) |
| C(13) | Ho(1) | C(3) | 121.5(4) |
| C(13) | Ho(1) | C(4) | 100.3(4) |
| C(13) | Ho(1) | C(5) | 88.7(5) |
| C(13) | Ho(1) | C(6) | 83.5(6) |
| C(13) | Ho(1) | C(7) | 87.8(7) |
| C(13) | Ho(1) | C(8) | 106.5(5) |
| C(14) | Ho(1) | C(2) | 126.2(5) |
| C(14) | Ho(1) | C(3) | 99.9(5) |
| C(14) | Ho(1) | C(4) | 87.2(5) |
| C(14) | Ho(1) | C(5) | 87.4(6) |
| C(14) | Ho(1) | C(6) | 94.3(7) |
| C(14) | Ho(1) | C(7) | 108.6(7) |
| C(14) | Ho(1) | C(8) | 132.9(7) |
| C(14) | Ho(1) | C(9) | 161.6(9) |
| C(14) | Ho(1) | C(13) | 31.3(3) |
| C(15) | Ho(1) | C(1) | 133.3(6) |
| C(15) | Ho(1) | C(2) | 106.3(6) |
| C(15) | Ho(1) | C(3) | 89.7(6) |
| C(15) | Ho(1) | C(4) | 90.5(5) |
| C(15) | Ho(1) | C(5) | 102.6(6) |
| C(15) | Ho(1) | C(6) | 118.4(7) |
| C(15) | Ho(1) | C(7) | 138.0(8) |
| C(15) | Ho(1) | C(8) | 164.3(8) |
| C(15) | Ho(1) | C(9) | 163.9(6) |
| C(15) | Ho(1) | C(12) | 83.8(6) |
| C(15) | Ho(1) | C(13) | 61.1(5) |
| C(15) | Ho(1) | C(14) | 32.3(3) |
| C(16) | Ho(1) | C(1) | 113.0(7) |
| C(16) | Ho(1) | C(2) | 98.1(7) |
| C(16) | Ho(1) | C(3) | 97.4(6) |
| C(16) | Ho(1) | C(4) | 110.8(5) |
| C(16) | Ho(1) | C(5) | 130.1(5) |
| C(16) | Ho(1) | C(6) | 150.3(6) |
| C(16) | Ho(1) | C(7) | 171.1(9) |
| C(16) | Ho(1) | C(8) | 161.9(8) |
| C(16) | Ho(1) | C(9) | 135.9(8) |
| C(16) | Ho(1) | C(10) | 63.5(5) |
| C(16) | Ho(1) | C(11) | 85.6(6) |
| C(16) | Ho(1) | C(12) | 93.4(7) |
| C(16) | Ho(1) | C(13) | 84.2(7) |
| C(16) | Ho(1) | C(14) | 62.5(6) |
| C(16) | Ho(1) | C(15) | 33.4(4) |
| C(17) | Ho(1) | C(1) | 101.0(7) |
| C(17) | Ho(1) | C(2) | 103.8(6) |

| | | | |
|---|---|---|---|
| C(17) | Ho(1) | C(3) | 118.4(5) |
| C(17) | Ho(1) | C(4) | 139.8(4) |
| C(17) | Ho(1) | C(5) | 162.5(4) |
| C(17) | Ho(1) | C(6) | 174.4(5) |
| C(17) | Ho(1) | C(7) | 151.8(6) |
| C(17) | Ho(1) | C(8) | 129.0(7) |
| C(17) | Ho(1) | C(9) | 110.3(7) |
| C(17) | Ho(1) | C(10) | 33.3(3) |
| C(17) | Ho(1) | C(11) | 63.0(4) |
| C(17) | Ho(1) | C(12) | 85.6(6) |
| C(17) | Ho(1) | C(13) | 94.3(6) |
| C(17) | Ho(1) | C(14) | 86.3(7) |
| C(17) | Ho(1) | C(15) | 64.4(6) |
| C(17) | Ho(1) | C(16) | 33.7(4) |
| C(2) | C(1) | Ho(1) | 76.7(11) |
| C(9) | C(1) | Ho(1) | 77.2(11) |
| C(9) | C(1) | C(2) | 140.3(18) |
| C(1) | C(2) | Ho(1) | 72.6(10) |
| C(1) | C(2) | C(3) | 140.1(18) |
| C(3) | C(2) | Ho(1) | 84.1(10) |
| C(2) | C(3) | Ho(1) | 66.8(10) |
| C(2) | C(3) | C(4) | 138.6(17) |
| C(4) | C(3) | Ho(1) | 93.4(10) |
| C(3) | C(4) | Ho(1) | 61.2(9) |
| C(5) | C(4) | Ho(1) | 88.7(9) |
| C(5) | C(4) | C(3) | 138.2(15) |
| C(4) | C(5) | Ho(1) | 67.9(8) |
| C(6) | C(5) | Ho(1) | 74.4(11) |
| C(6) | C(5) | C(4) | 139.0(15) |
| C(5) | C(6) | Ho(1) | 82.6(11) |
| C(7) | C(6) | Ho(1) | 63.6(16) |
| C(7) | C(6) | C(5) | 140.2(18) |
| C(6) | C(7) | Ho(1) | 93.0(17) |
| C(6) | C(7) | C(8) | 139.9(19) |
| C(8) | C(7) | Ho(1) | 64.4(16) |
| C(7) | C(8) | Ho(1) | 89.2(18) |
| C(7) | C(8) | C(9) | 140.8(19) |
| C(9) | C(8) | Ho(1) | 71.1(13) |
| C(1) | C(9) | Ho(1) | 72.1(11) |
| C(8) | C(9) | Ho(1) | 79.6(14) |
| C(8) | C(9) | C(1) | 137.4(19) |
| C(11) | C(10) | Ho(1) | 74.9(8) |
| C(11) | C(10) | C(17) | 135.6(14) |
| C(17) | C(10) | Ho(1) | 72.2(10) |
| C(10) | C(11) | Ho(1) | 72.8(8) |
| C(10) | C(11) | C(12) | 135.7(14) |
| C(12) | C(11) | Ho(1) | 76.2(8) |
| C(11) | C(12) | Ho(1) | 71.5(8) |
| C(13) | C(12) | Ho(1) | 77.1(8) |
| C(13) | C(12) | C(11) | 136.3(14) |
| C(12) | C(13) | Ho(1) | 71.5(8) |
| C(12) | C(13) | C(14) | 133.4(15) |
| C(14) | C(13) | Ho(1) | 72.6(9) |
| C(13) | C(14) | Ho(1) | 76.1(9) |

| | | | |
|---|---|---|---|
| C(13) | C(14) | C(15) | 136.1(16) |
| C(15) | C(14) | Ho(1) | 71.8(13) |
| C(14) | C(15) | Ho(1) | 75.9(13) |
| C(14) | C(15) | C(16) | 132.7(19) |
| C(16) | C(15) | Ho(1) | 70.1(17) |
| C(15) | C(16) | Ho(1) | 76.6(17) |
| C(17) | C(16) | Ho(1) | 73.0(16) |
| C(17) | C(16) | C(15) | 137(2) |
| C(10) | C(17) | Ho(1) | 74.5(9) |
| C(10) | C(17) | C(16) | 132.7(18) |
| C(16) | C(17) | Ho(1) | 73.3(17) |

**Table S17.** Bond lengths (Å) and angles (deg) for [Ho(Cnt)(Cot)] **(3)** at 300 K. (Continuing over several pages).

| Atom A | Atom B | Distance(esd) |
|---|---|---|
| Ho(1) | C(1) | 2.667(13) |
| Ho(1) | C(2) | 2.717(11) |
| Ho(1) | C(3) | 2.909(12) |
| Ho(1) | C(4) | 3.171(14) |
| Ho(1) | C(5) | 3.294(14) |
| Ho(1) | C(6) | 3.163(13) |
| Ho(1) | C(7) | 2.897(15) |
| Ho(1) | C(8) | 2.75(2) |
| Ho(1) | C(9) | 2.719(16) |
| Ho(1) | C(10) | 2.557(13) |
| Ho(1) | C(11) | 2.538(12) |
| Ho(1) | C(12) | 2.545(10) |
| Ho(1) | C(13) | 2.571(13) |
| Ho(1) | C(14) | 2.494(18) |
| Ho(1) | C(15) | 2.42(3) |
| Ho(1) | C(16) | 2.458(17) |
| Ho(1) | C(17) | 2.535(14) |
| C(1) | C(2) | 1.367(12) |
| C(1) | C(9) | 1.345(12) |
| C(2) | C(3) | 1.377(12) |
| C(3) | C(4) | 1.379(12) |
| C(4) | C(5) | 1.365(11) |
| C(5) | C(6) | 1.364(10) |
| C(6) | C(7) | 1.340(11) |
| C(7) | C(8) | 1.300(12) |
| C(8) | C(9) | 1.313(12) |
| C(10) | C(11) | 1.402(10) |
| C(10) | C(17) | 1.409(11) |
| C(11) | C(12) | 1.411(10) |
| C(12) | C(13) | 1.408(11) |
| C(13) | C(14) | 1.406(11) |
| C(14) | C(15) | 1.443(13) |
| C(15) | C(16) | 1.443(13) |
| C(16) | C(17) | 1.405(12) |

| Atom A | Atom B | Atom C | Angle(esd) |
|---|---|---|---|
| C(1) | Ho(1) | C(2) | 29.4(3) |
| C(1) | Ho(1) | C(3) | 54.8(3) |
| C(1) | Ho(1) | C(4) | 72.5(4) |
| C(1) | Ho(1) | C(5) | 81.1(4) |
| C(1) | Ho(1) | C(6) | 82.2(4) |
| C(1) | Ho(1) | C(7) | 73.7(4) |
| C(1) | Ho(1) | C(8) | 54.6(4) |
| C(1) | Ho(1) | C(9) | 28.9(3) |
| C(2) | Ho(1) | C(3) | 28.1(2) |
| C(2) | Ho(1) | C(4) | 51.5(3) |
| C(2) | Ho(1) | C(5) | 69.2(3) |
| C(2) | Ho(1) | C(6) | 82.0(3) |
| C(2) | Ho(1) | C(7) | 85.9(4) |
| C(2) | Ho(1) | C(8) | 76.3(5) |

| | | | |
|---|---|---|---|
| C(2) | Ho(1) | C(9) | 56.0(4) |
| C(3) | Ho(1) | C(4) | 25.8(2) |
| C(3) | Ho(1) | C(5) | 48.2(3) |
| C(3) | Ho(1) | C(6) | 68.3(3) |
| C(4) | Ho(1) | C(5) | 24.28(19) |
| C(6) | Ho(1) | C(4) | 47.6(3) |
| C(6) | Ho(1) | C(5) | 24.3(2) |
| C(7) | Ho(1) | C(3) | 83.1(4) |
| C(7) | Ho(1) | C(4) | 68.5(3) |
| C(7) | Ho(1) | C(5) | 47.9(3) |
| C(7) | Ho(1) | C(6) | 25.1(2) |
| C(8) | Ho(1) | C(3) | 86.1(5) |
| C(8) | Ho(1) | C(4) | 82.0(4) |
| C(8) | Ho(1) | C(5) | 67.9(4) |
| C(8) | Ho(1) | C(6) | 49.2(3) |
| C(8) | Ho(1) | C(7) | 26.5(3) |
| C(9) | Ho(1) | C(3) | 75.6(4) |
| C(9) | Ho(1) | C(4) | 83.6(4) |
| C(9) | Ho(1) | C(5) | 80.2(4) |
| C(9) | Ho(1) | C(6) | 69.6(4) |
| C(9) | Ho(1) | C(7) | 52.0(4) |
| C(9) | Ho(1) | C(8) | 27.8(3) |
| C(10) | Ho(1) | C(1) | 101.4(4) |
| C(10) | Ho(1) | C(2) | 120.1(3) |
| C(10) | Ho(1) | C(3) | 145.0(3) |
| C(10) | Ho(1) | C(4) | 170.7(2) |
| C(10) | Ho(1) | C(5) | 164.0(3) |
| C(10) | Ho(1) | C(6) | 139.8(3) |
| C(10) | Ho(1) | C(7) | 117.2(4) |
| C(10) | Ho(1) | C(8) | 100.4(5) |
| C(10) | Ho(1) | C(9) | 94.3(5) |
| C(10) | Ho(1) | C(13) | 83.7(4) |
| C(11) | Ho(1) | C(1) | 117.4(3) |
| C(11) | Ho(1) | C(2) | 144.8(3) |
| C(11) | Ho(1) | C(3) | 172.2(3) |
| C(11) | Ho(1) | C(4) | 157.3(3) |
| C(11) | Ho(1) | C(5) | 133.3(3) |
| C(11) | Ho(1) | C(6) | 111.2(3) |
| C(11) | Ho(1) | C(7) | 93.8(4) |
| C(11) | Ho(1) | C(8) | 87.9(5) |
| C(11) | Ho(1) | C(9) | 96.8(4) |
| C(11) | Ho(1) | C(10) | 31.9(2) |
| C(11) | Ho(1) | C(12) | 32.2(2) |
| C(11) | Ho(1) | C(13) | 61.3(3) |
| C(12) | Ho(1) | C(1) | 141.2(3) |
| C(12) | Ho(1) | C(2) | 168.7(4) |
| C(12) | Ho(1) | C(3) | 153.2(3) |
| C(12) | Ho(1) | C(4) | 127.6(3) |
| C(12) | Ho(1) | C(5) | 106.7(3) |
| C(12) | Ho(1) | C(6) | 90.5(3) |
| C(12) | Ho(1) | C(7) | 83.7(4) |
| C(12) | Ho(1) | C(8) | 92.3(5) |
| C(12) | Ho(1) | C(9) | 113.4(4) |
| C(12) | Ho(1) | C(10) | 61.5(3) |

| | | | |
|---|---|---|---|
| C(12) | Ho(1) | C(13) | 31.9(2) |
| C(13) | Ho(1) | C(1) | 166.1(5) |
| C(13) | Ho(1) | C(2) | 153.9(3) |
| C(13) | Ho(1) | C(3) | 125.9(3) |
| C(13) | Ho(1) | C(4) | 103.8(3) |
| C(13) | Ho(1) | C(5) | 90.5(3) |
| C(13) | Ho(1) | C(6) | 85.6(4) |
| C(13) | Ho(1) | C(7) | 92.4(5) |
| C(13) | Ho(1) | C(8) | 112.0(5) |
| C(13) | Ho(1) | C(9) | 138.8(4) |
| C(14) | Ho(1) | C(1) | 156.9(4) |
| C(14) | Ho(1) | C(2) | 127.5(4) |
| C(14) | Ho(1) | C(3) | 103.4(4) |
| C(14) | Ho(1) | C(4) | 90.2(5) |
| C(14) | Ho(1) | C(5) | 89.3(5) |
| C(14) | Ho(1) | C(6) | 97.5(5) |
| C(14) | Ho(1) | C(7) | 114.7(6) |
| C(14) | Ho(1) | C(8) | 139.6(6) |
| C(14) | Ho(1) | C(9) | 166.6(7) |
| C(14) | Ho(1) | C(10) | 93.7(5) |
| C(14) | Ho(1) | C(11) | 84.4(4) |
| C(14) | Ho(1) | C(12) | 61.8(4) |
| C(14) | Ho(1) | C(13) | 32.2(3) |
| C(14) | Ho(1) | C(17) | 86.5(5) |
| C(15) | Ho(1) | C(1) | 129.4(5) |
| C(15) | Ho(1) | C(2) | 105.1(5) |
| C(15) | Ho(1) | C(3) | 91.9(5) |
| C(15) | Ho(1) | C(4) | 93.1(5) |
| C(15) | Ho(1) | C(5) | 105.4(5) |
| C(15) | Ho(1) | C(6) | 123.3(5) |
| C(15) | Ho(1) | C(7) | 146.0(6) |
| C(15) | Ho(1) | C(8) | 172.4(7) |
| C(15) | Ho(1) | C(9) | 157.7(6) |
| C(15) | Ho(1) | C(10) | 85.4(6) |
| C(15) | Ho(1) | C(11) | 94.6(6) |
| C(15) | Ho(1) | C(12) | 86.1(5) |
| C(15) | Ho(1) | C(13) | 63.5(4) |
| C(15) | Ho(1) | C(14) | 34.1(3) |
| C(15) | Ho(1) | C(16) | 34.4(4) |
| C(15) | Ho(1) | C(17) | 63.4(5) |
| C(16) | Ho(1) | C(1) | 106.2(5) |
| C(16) | Ho(1) | C(2) | 94.2(5) |
| C(16) | Ho(1) | C(3) | 97.2(4) |
| C(16) | Ho(1) | C(4) | 111.9(4) |
| C(16) | Ho(1) | C(5) | 132.5(3) |
| C(16) | Ho(1) | C(6) | 155.4(4) |
| C(16) | Ho(1) | C(7) | 179.6(5) |
| C(16) | Ho(1) | C(8) | 153.2(6) |
| C(16) | Ho(1) | C(9) | 127.7(6) |
| C(16) | Ho(1) | C(10) | 62.4(4) |
| C(16) | Ho(1) | C(11) | 85.9(4) |
| C(16) | Ho(1) | C(12) | 96.1(5) |
| C(16) | Ho(1) | C(13) | 87.6(5) |
| C(16) | Ho(1) | C(14) | 65.6(5) |

| | | | |
|---|---|---|---|
| C(16) | Ho(1) | C(17) | 32.6(3) |
| C(17) | Ho(1) | C(1) | 97.0(4) |
| C(17) | Ho(1) | C(2) | 101.4(4) |
| C(17) | Ho(1) | C(3) | 118.0(4) |
| C(17) | Ho(1) | C(4) | 140.1(3) |
| C(17) | Ho(1) | C(5) | 163.9(3) |
| C(17) | Ho(1) | C(6) | 171.7(3) |
| C(17) | Ho(1) | C(7) | 146.9(4) |
| C(17) | Ho(1) | C(8) | 123.9(5) |
| C(17) | Ho(1) | C(9) | 105.9(5) |
| C(17) | Ho(1) | C(10) | 32.1(3) |
| C(17) | Ho(1) | C(11) | 61.7(3) |
| C(17) | Ho(1) | C(12) | 84.9(4) |
| C(17) | Ho(1) | C(13) | 94.1(4) |
| C(2) | C(1) | Ho(1) | 77.4(7) |
| C(9) | C(1) | Ho(1) | 77.7(8) |
| C(9) | C(1) | C(2) | 140.3(12) |
| C(1) | C(2) | Ho(1) | 73.2(7) |
| C(1) | C(2) | C(3) | 139.6(11) |
| C(3) | C(2) | Ho(1) | 83.7(7) |
| C(2) | C(3) | Ho(1) | 68.2(6) |
| C(2) | C(3) | C(4) | 140.1(10) |
| C(4) | C(3) | Ho(1) | 87.8(7) |
| C(3) | C(4) | Ho(1) | 66.4(7) |
| C(5) | C(4) | Ho(1) | 82.9(8) |
| C(5) | C(4) | C(3) | 137.7(11) |
| C(4) | C(5) | Ho(1) | 72.8(8) |
| C(6) | C(5) | Ho(1) | 72.5(7) |
| C(6) | C(5) | C(4) | 139.0(12) |
| C(5) | C(6) | Ho(1) | 83.2(8) |
| C(7) | C(6) | Ho(1) | 66.3(8) |
| C(7) | C(6) | C(5) | 140.0(12) |
| C(6) | C(7) | Ho(1) | 88.7(8) |
| C(8) | C(7) | Ho(1) | 70.3(11) |
| C(8) | C(7) | C(6) | 141.2(12) |
| C(7) | C(8) | Ho(1) | 83.2(11) |
| C(7) | C(8) | C(9) | 141.7(14) |
| C(9) | C(8) | Ho(1) | 74.9(11) |
| C(1) | C(9) | Ho(1) | 73.4(8) |
| C(8) | C(9) | Ho(1) | 77.3(12) |
| C(8) | C(9) | C(1) | 138.2(13) |
| C(11) | C(10) | Ho(1) | 73.3(6) |
| C(11) | C(10) | C(17) | 135.6(11) |
| C(17) | C(10) | Ho(1) | 73.1(7) |
| C(10) | C(11) | Ho(1) | 74.8(7) |
| C(10) | C(11) | C(12) | 136.3(10) |
| C(12) | C(11) | Ho(1) | 74.2(6) |
| C(11) | C(12) | Ho(1) | 73.6(6) |
| C(13) | C(12) | Ho(1) | 75.1(6) |
| C(13) | C(12) | C(11) | 135.2(10) |
| C(12) | C(13) | Ho(1) | 73.0(6) |
| C(14) | C(13) | Ho(1) | 70.9(8) |
| C(14) | C(13) | C(12) | 133.5(11) |
| C(13) | C(14) | Ho(1) | 76.9(8) |

| | | | |
|---|---|---|---|
| C(13) | C(14) | C(15) | 134.8(13) |
| C(15) | C(14) | Ho(1) | 70.3(12) |
| C(14) | C(15) | Ho(1) | 75.6(12) |
| C(14) | C(15) | C(16) | 136.7(14) |
| C(16) | C(15) | Ho(1) | 74.1(11) |
| C(15) | C(16) | Ho(1) | 71.5(12) |
| C(17) | C(16) | Ho(1) | 76.7(8) |
| C(17) | C(16) | C(15) | 132.6(13) |
| C(10) | C(17) | Ho(1) | 74.8(7) |
| C(16) | C(17) | Ho(1) | 70.7(8) |
| C(16) | C(17) | C(10) | 134.9(11) |

**Table S18.** Bond lengths (Å) and angles (deg) for [Er(Cnt)(Cot)] **(4)** at 150 K. (Continuing over several pages).

| Atom A | Atom B | Distance(esd) |
|---|---|---|
| Er(1) | C(1) | 2.55(3) |
| Er(1) | C(2) | 2.61(3) |
| Er(1) | C(3) | 2.881(17) |
| Er(1) | C(4) | 3.443(13) |
| Er(1) | C(5) | 3.763(13) |
| Er(1) | C(6) | 3.597(15) |
| Er(1) | C(7) | 3.10(2) |
| Er(1) | C(8) | 2.69(3) |
| Er(1) | C(9) | 2.59(3) |
| Er(1) | C(10) | 2.470(13) |
| Er(1) | C(11) | 2.499(15) |
| Er(1) | C(12) | 2.50(2) |
| Er(1) | C(13) | 2.55(3) |
| Er(1) | C(14) | 2.51(3) |
| Er(1) | C(15) | 2.52(3) |
| Er(1) | C(16) | 2.45(3) |
| Er(1) | C(17) | 2.448(16) |
| C(1) | C(2) | 1.377(14) |
| C(1) | C(9) | 1.382(14) |
| C(2) | C(3) | 1.396(13) |
| C(3) | C(4) | 1.379(11) |
| C(4) | C(5) | 1.382(11) |
| C(5) | C(6) | 1.381(11) |
| C(6) | C(7) | 1.371(11) |
| C(7) | C(8) | 1.380(12) |
| C(8) | C(9) | 1.382(13) |
| C(10) | C(11) | 1.393(12) |
| C(10) | C(17) | 1.390(13) |
| C(11) | C(12) | 1.410(13) |
| C(12) | C(13) | 1.397(14) |
| C(13) | C(14) | 1.399(14) |
| C(14) | C(15) | 1.401(14) |
| C(15) | C(16) | 1.394(14) |
| C(16) | C(17) | 1.381(14) |

| Atom A | Atom B | Atom C | Angle(esd) |
|---|---|---|---|
| C(1) | Er(1) | C(2) | 30.9(4) |
| C(1) | Er(1) | C(3) | 56.6(6) |
| C(1) | Er(1) | C(4) | 69.9(8) |
| C(1) | Er(1) | C(5) | 75.4(9) |
| C(1) | Er(1) | C(6) | 77.9(9) |
| C(1) | Er(1) | C(7) | 75.4(8) |
| C(1) | Er(1) | C(8) | 58.5(7) |
| C(1) | Er(1) | C(9) | 31.2(4) |
| C(2) | Er(1) | C(3) | 28.9(3) |
| C(2) | Er(1) | C(4) | 48.9(4) |
| C(2) | Er(1) | C(5) | 63.8(5) |
| C(2) | Er(1) | C(6) | 77.1(6) |
| C(2) | Er(1) | C(7) | 86.2(6) |
| C(2) | Er(1) | C(8) | 80.3(7) |

| | | | |
|---|---|---|---|
| C(3) | Er(1) | C(4) | 23.1(3) |
| C(3) | Er(1) | C(5) | 43.2(3) |
| C(3) | Er(1) | C(6) | 62.6(3) |
| C(3) | Er(1) | C(7) | 80.0(4) |
| C(4) | Er(1) | C(5) | 21.53(18) |
| C(4) | Er(1) | C(6) | 42.7(2) |
| C(6) | Er(1) | C(5) | 21.48(18) |
| C(7) | Er(1) | C(4) | 63.1(3) |
| C(7) | Er(1) | C(5) | 43.0(3) |
| C(7) | Er(1) | C(6) | 22.1(2) |
| C(8) | Er(1) | C(3) | 87.2(6) |
| C(8) | Er(1) | C(4) | 78.4(5) |
| C(8) | Er(1) | C(5) | 62.7(4) |
| C(8) | Er(1) | C(6) | 45.4(4) |
| C(8) | Er(1) | C(7) | 26.4(3) |
| C(9) | Er(1) | C(2) | 59.7(7) |
| C(9) | Er(1) | C(3) | 78.6(9) |
| C(9) | Er(1) | C(4) | 81.7(10) |
| C(9) | Er(1) | C(5) | 75.6(10) |
| C(9) | Er(1) | C(6) | 66.4(9) |
| C(9) | Er(1) | C(7) | 53.8(6) |
| C(9) | Er(1) | C(8) | 30.3(4) |
| C(10) | Er(1) | C(11) | 32.5(3) |
| C(10) | Er(1) | C(12) | 62.6(5) |
| C(10) | Er(1) | C(13) | 85.4(8) |
| C(10) | Er(1) | C(14) | 94.5(10) |
| C(10) | Er(1) | C(15) | 85.6(7) |
| C(11) | Er(1) | C(12) | 32.8(3) |
| C(11) | Er(1) | C(13) | 62.3(6) |
| C(11) | Er(1) | C(14) | 84.8(9) |
| C(11) | Er(1) | C(15) | 93.8(7) |
| C(12) | Er(1) | C(13) | 32.1(4) |
| C(12) | Er(1) | C(14) | 61.5(7) |
| C(12) | Er(1) | C(15) | 84.1(8) |
| C(14) | Er(1) | C(13) | 32.1(4) |
| C(14) | Er(1) | C(15) | 32.3(4) |
| C(15) | Er(1) | C(13) | 61.7(8) |
| C(16) | Er(1) | C(10) | 62.4(5) |
| C(16) | Er(1) | C(11) | 84.4(5) |
| C(16) | Er(1) | C(12) | 92.8(6) |
| C(16) | Er(1) | C(13) | 84.2(9) |
| C(16) | Er(1) | C(14) | 62.0(7) |
| C(16) | Er(1) | C(15) | 32.5(4) |
| C(16) | Er(1) | C(17) | 32.8(4) |
| C(17) | Er(1) | C(10) | 32.8(3) |
| C(17) | Er(1) | C(11) | 62.6(4) |
| C(17) | Er(1) | C(12) | 85.4(6) |
| C(17) | Er(1) | C(13) | 94.7(10) |
| C(17) | Er(1) | C(14) | 85.8(10) |
| C(17) | Er(1) | C(15) | 63.1(6) |
| C(2) | C(1) | Er(1) | 76.7(18) |
| C(2) | C(1) | C(9) | 139(3) |
| C(9) | C(1) | Er(1) | 75.8(17) |
| C(1) | C(2) | Er(1) | 72.4(18) |

| | | | |
|---|---|---|---|
| C(1) | C(2) | C(3) | 138(2) |
| C(3) | C(2) | Er(1) | 86.5(14) |
| C(2) | C(3) | Er(1) | 64.6(13) |
| C(4) | C(3) | Er(1) | 102.0(11) |
| C(4) | C(3) | C(2) | 141.0(19) |
| C(3) | C(4) | Er(1) | 55.0(10) |
| C(3) | C(4) | C(5) | 138.2(14) |
| C(5) | C(4) | Er(1) | 92.4(7) |
| Er(1)1 | C(5) | Er(1) | 4.29(5) |
| C(4) | C(5) | Er(1) | 66.1(6) |
| C(6) | C(5) | Er(1) | 72.5(7) |
| C(6) | C(5) | C(4) | 136.4(11) |
| C(5) | C(6) | Er(1) | 86.0(8) |
| C(7) | C(6) | Er(1) | 58.2(12) |
| C(7) | C(6) | C(5) | 140.3(15) |
| C(6) | C(7) | Er(1) | 99.8(13) |
| C(6) | C(7) | C(8) | 137.6(16) |
| C(8) | C(7) | Er(1) | 59.8(14) |
| C(7) | C(8) | Er(1) | 93.9(16) |
| C(7) | C(8) | C(9) | 142(2) |
| C(9) | C(8) | Er(1) | 70.9(16) |
| C(1) | C(9) | Er(1) | 73.1(17) |
| C(1) | C(9) | C(8) | 136(3) |
| C(8) | C(9) | Er(1) | 78.8(16) |
| C(11) | C(10) | Er(1) | 74.9(8) |
| C(17) | C(10) | Er(1) | 72.7(8) |
| C(17) | C(10) | C(11) | 135.0(14) |
| C(10) | C(11) | Er(1) | 72.6(8) |
| C(10) | C(11) | C(12) | 134.1(15) |
| C(12) | C(11) | Er(1) | 73.6(11) |
| C(11) | C(12) | Er(1) | 73.6(11) |
| C(13) | C(12) | Er(1) | 75.9(15) |
| C(13) | C(12) | C(11) | 137.1(19) |
| C(12) | C(13) | Er(1) | 72.0(15) |
| C(12) | C(13) | C(14) | 133(3) |
| C(14) | C(13) | Er(1) | 72.3(16) |
| C(13) | C(14) | Er(1) | 75.6(17) |
| C(13) | C(14) | C(15) | 137(3) |
| C(15) | C(14) | Er(1) | 74.4(18) |
| C(14) | C(15) | Er(1) | 73.3(18) |
| C(16) | C(15) | Er(1) | 70.7(18) |
| C(16) | C(15) | C(14) | 132(3) |
| C(15) | C(16) | Er(1) | 76.8(18) |
| C(17) | C(16) | Er(1) | 73.7(13) |
| C(17) | C(16) | C(15) | 139(2) |
| C(10) | C(17) | Er(1) | 74.5(8) |
| C(16) | C(17) | Er(1) | 73.5(14) |
| C(16) | C(17) | C(10) | 133.4(17) |

**Table S19.** Bond lengths (Å) and angles (deg) for [Er(Cnt)(Cot)] **(4)** at 300 K. (Continuing over several pages).

| Atom A | Atom B | Distance(esd) |
|---|---|---|
| Er(1) | C(1) | 2.56(2) |
| Er(1) | C(2) | 2.689(17) |
| Er(1) | C(3) | 3.025(18) |
| Er(1) | C(4) | 3.414(17) |
| Er(1) | C(5) | 3.567(17) |
| Er(1) | C(6) | 3.389(15) |
| Er(1) | C(7) | 2.933(17) |
| Er(1) | C(8) | 2.65(3) |
| Er(1) | C(9) | 2.59(2) |
| Er(1) | C(10) | 2.512(18) |
| Er(1) | C(11) | 2.484(18) |
| Er(1) | C(12) | 2.515(17) |
| Er(1) | C(13) | 2.58(2) |
| Er(1) | C(14) | 2.51(3) |
| Er(1) | C(15) | 2.46(3) |
| Er(1) | C(16) | 2.43(3) |
| Er(1) | C(17) | 2.488(16) |
| C(1) | C(2) | 1.364(13) |
| C(1) | C(9) | 1.334(13) |
| C(2) | C(3) | 1.361(13) |
| C(3) | C(4) | 1.372(12) |
| C(4) | C(5) | 1.377(11) |
| C(5) | C(6) | 1.364(12) |
| C(6) | C(7) | 1.343(12) |
| C(7) | C(8) | 1.318(13) |
| C(8) | C(9) | 1.316(13) |
| C(10) | C(11) | 1.398(13) |
| C(10) | C(17) | 1.410(13) |
| C(11) | C(12) | 1.403(13) |
| C(12) | C(13) | 1.402(12) |
| C(13) | C(14) | 1.404(14) |
| C(14) | C(15) | 1.424(14) |
| C(15) | C(16) | 1.425(14) |
| C(16) | C(17) | 1.411(14) |

| Atom A | Atom B | Atom C | Angle(esd) |
|---|---|---|---|
| C(1) | Er(1) | C(2) | 30.0(3) |
| C(1) | Er(1) | C(3) | 53.8(4) |
| C(1) | Er(1) | C(4) | 69.1(5) |
| C(1) | Er(1) | C(5) | 77.0(5) |
| C(1) | Er(1) | C(6) | 79.5(5) |
| C(1) | Er(1) | C(7) | 74.1(6) |
| C(1) | Er(1) | C(8) | 56.6(5) |
| C(1) | Er(1) | C(9) | 30.0(4) |
| C(1) | Er(1) | C(13) | 162.1(9) |
| C(2) | Er(1) | C(3) | 26.7(3) |
| C(2) | Er(1) | C(4) | 48.0(4) |
| C(2) | Er(1) | C(5) | 64.8(4) |
| C(2) | Er(1) | C(6) | 78.2(4) |
| C(2) | Er(1) | C(7) | 84.7(5) |

| | | | |
|---|---|---|---|
| C(3) | Er(1) | C(4) | 23.6(2) |
| C(3) | Er(1) | C(5) | 44.8(3) |
| C(3) | Er(1) | C(6) | 64.2(4) |
| C(4) | Er(1) | C(5) | 22.61(19) |
| C(6) | Er(1) | C(4) | 44.3(3) |
| C(6) | Er(1) | C(5) | 22.4(2) |
| C(7) | Er(1) | C(3) | 79.7(4) |
| C(7) | Er(1) | C(4) | 64.3(4) |
| C(7) | Er(1) | C(5) | 44.4(3) |
| C(7) | Er(1) | C(6) | 23.1(3) |
| C(8) | Er(1) | C(2) | 77.9(6) |
| C(8) | Er(1) | C(3) | 85.2(6) |
| C(8) | Er(1) | C(4) | 78.9(5) |
| C(8) | Er(1) | C(5) | 64.7(4) |
| C(8) | Er(1) | C(6) | 47.4(4) |
| C(8) | Er(1) | C(7) | 26.7(3) |
| C(9) | Er(1) | C(2) | 57.4(5) |
| C(9) | Er(1) | C(3) | 75.0(6) |
| C(9) | Er(1) | C(4) | 80.5(6) |
| C(9) | Er(1) | C(5) | 76.7(5) |
| C(9) | Er(1) | C(6) | 67.7(5) |
| C(9) | Er(1) | C(7) | 52.8(5) |
| C(9) | Er(1) | C(8) | 29.0(4) |
| C(10) | Er(1) | C(1) | 109.2(6) |
| C(10) | Er(1) | C(2) | 127.9(5) |
| C(10) | Er(1) | C(3) | 150.8(4) |
| C(10) | Er(1) | C(4) | 173.7(4) |
| C(10) | Er(1) | C(5) | 163.7(3) |
| C(10) | Er(1) | C(6) | 141.9(4) |
| C(10) | Er(1) | C(7) | 121.5(5) |
| C(10) | Er(1) | C(8) | 105.4(6) |
| C(10) | Er(1) | C(9) | 101.0(6) |
| C(10) | Er(1) | C(12) | 62.6(5) |
| C(10) | Er(1) | C(13) | 84.0(6) |
| C(11) | Er(1) | C(1) | 124.6(5) |
| C(11) | Er(1) | C(2) | 153.1(5) |
| C(11) | Er(1) | C(3) | 175.5(7) |
| C(11) | Er(1) | C(4) | 153.4(4) |
| C(11) | Er(1) | C(5) | 131.6(4) |
| C(11) | Er(1) | C(6) | 111.6(5) |
| C(11) | Er(1) | C(7) | 95.8(6) |
| C(11) | Er(1) | C(8) | 90.5(6) |
| C(11) | Er(1) | C(9) | 101.9(6) |
| C(11) | Er(1) | C(10) | 32.5(3) |
| C(11) | Er(1) | C(12) | 32.6(3) |
| C(11) | Er(1) | C(13) | 61.2(5) |
| C(11) | Er(1) | C(14) | 83.9(7) |
| C(11) | Er(1) | C(17) | 63.0(5) |
| C(12) | Er(1) | C(1) | 144.9(5) |
| C(12) | Er(1) | C(2) | 166.5(8) |
| C(12) | Er(1) | C(3) | 145.7(4) |
| C(12) | Er(1) | C(4) | 122.4(4) |
| C(12) | Er(1) | C(5) | 103.4(5) |
| C(12) | Er(1) | C(6) | 88.5(5) |

| | | | |
|---|---|---|---|
| C(12) | Er(1) | C(7) | 82.0(6) |
| C(12) | Er(1) | C(8) | 91.2(6) |
| C(12) | Er(1) | C(9) | 115.1(6) |
| C(12) | Er(1) | C(13) | 31.9(3) |
| C(13) | Er(1) | C(2) | 145.5(5) |
| C(13) | Er(1) | C(3) | 118.8(5) |
| C(13) | Er(1) | C(4) | 99.1(5) |
| C(13) | Er(1) | C(5) | 87.2(5) |
| C(13) | Er(1) | C(6) | 82.6(6) |
| C(13) | Er(1) | C(7) | 88.7(6) |
| C(13) | Er(1) | C(8) | 109.1(7) |
| C(13) | Er(1) | C(9) | 137.8(7) |
| C(14) | Er(1) | C(1) | 151.3(6) |
| C(14) | Er(1) | C(2) | 121.3(6) |
| C(14) | Er(1) | C(3) | 98.0(6) |
| C(14) | Er(1) | C(4) | 86.6(7) |
| C(14) | Er(1) | C(5) | 86.4(7) |
| C(14) | Er(1) | C(6) | 94.0(7) |
| C(14) | Er(1) | C(7) | 109.7(7) |
| C(14) | Er(1) | C(8) | 135.2(8) |
| C(14) | Er(1) | C(9) | 161.7(10) |
| C(14) | Er(1) | C(10) | 93.2(7) |
| C(14) | Er(1) | C(12) | 61.7(6) |
| C(14) | Er(1) | C(13) | 32.0(4) |
| C(15) | Er(1) | C(1) | 128.6(7) |
| C(15) | Er(1) | C(2) | 102.5(6) |
| C(15) | Er(1) | C(3) | 89.4(6) |
| C(15) | Er(1) | C(4) | 91.1(6) |
| C(15) | Er(1) | C(5) | 102.7(5) |
| C(15) | Er(1) | C(6) | 118.9(6) |
| C(15) | Er(1) | C(7) | 139.6(7) |
| C(15) | Er(1) | C(8) | 166.2(8) |
| C(15) | Er(1) | C(9) | 158.5(7) |
| C(15) | Er(1) | C(10) | 85.4(6) |
| C(15) | Er(1) | C(11) | 94.4(7) |
| C(15) | Er(1) | C(12) | 86.1(6) |
| C(15) | Er(1) | C(13) | 62.8(6) |
| C(15) | Er(1) | C(14) | 33.3(4) |
| C(15) | Er(1) | C(17) | 63.6(6) |
| C(16) | Er(1) | C(1) | 109.3(7) |
| C(16) | Er(1) | C(2) | 95.7(6) |
| C(16) | Er(1) | C(3) | 97.7(6) |
| C(16) | Er(1) | C(4) | 111.2(5) |
| C(16) | Er(1) | C(5) | 130.0(4) |
| C(16) | Er(1) | C(6) | 150.6(5) |
| C(16) | Er(1) | C(7) | 173.4(7) |
| C(16) | Er(1) | C(8) | 159.7(8) |
| C(16) | Er(1) | C(9) | 132.6(8) |
| C(16) | Er(1) | C(10) | 63.2(5) |
| C(16) | Er(1) | C(11) | 86.8(6) |
| C(16) | Er(1) | C(12) | 97.2(7) |
| C(16) | Er(1) | C(13) | 87.2(7) |
| C(16) | Er(1) | C(14) | 64.5(7) |
| C(16) | Er(1) | C(15) | 33.8(4) |

| | | | |
|---|---|---|---|
| C(16) | Er(1) | C(17) | 33.3(3) |
| C(17) | Er(1) | C(1) | 102.7(6) |
| C(17) | Er(1) | C(2) | 106.3(5) |
| C(17) | Er(1) | C(3) | 121.1(5) |
| C(17) | Er(1) | C(4) | 141.0(4) |
| C(17) | Er(1) | C(5) | 162.7(4) |
| C(17) | Er(1) | C(6) | 174.6(4) |
| C(17) | Er(1) | C(7) | 152.6(5) |
| C(17) | Er(1) | C(8) | 129.8(6) |
| C(17) | Er(1) | C(9) | 111.8(6) |
| C(17) | Er(1) | C(10) | 32.8(3) |
| C(17) | Er(1) | C(12) | 86.9(6) |
| C(17) | Er(1) | C(13) | 94.9(6) |
| C(17) | Er(1) | C(14) | 86.3(7) |
| C(2) | C(1) | Er(1) | 80.4(10) |
| C(9) | C(1) | Er(1) | 76.6(12) |
| C(9) | C(1) | C(2) | 140.4(17) |
| C(1) | C(2) | Er(1) | 69.6(10) |
| C(3) | C(2) | Er(1) | 90.5(10) |
| C(3) | C(2) | C(1) | 140.1(15) |
| C(2) | C(3) | Er(1) | 62.7(9) |
| C(2) | C(3) | C(4) | 139.9(15) |
| C(4) | C(3) | Er(1) | 94.3(10) |
| C(3) | C(4) | Er(1) | 62.1(9) |
| C(3) | C(4) | C(5) | 137.6(15) |
| C(5) | C(4) | Er(1) | 85.0(10) |
| C(4) | C(5) | Er(1) | 72.4(10) |
| C(6) | C(5) | Er(1) | 71.4(9) |
| C(6) | C(5) | C(4) | 139.1(15) |
| C(5) | C(6) | Er(1) | 86.2(9) |
| C(7) | C(6) | Er(1) | 59.0(9) |
| C(7) | C(6) | C(5) | 137.9(14) |
| C(6) | C(7) | Er(1) | 97.9(10) |
| C(8) | C(7) | Er(1) | 64.7(15) |
| C(8) | C(7) | C(6) | 142.8(16) |
| C(7) | C(8) | Er(1) | 88.6(16) |
| C(9) | C(8) | Er(1) | 73.0(16) |
| C(9) | C(8) | C(7) | 140.1(19) |
| C(1) | C(9) | Er(1) | 73.4(12) |
| C(8) | C(9) | Er(1) | 78.0(17) |
| C(8) | C(9) | C(1) | 137.6(19) |
| C(11) | C(10) | Er(1) | 72.7(10) |
| C(11) | C(10) | C(17) | 135.5(15) |
| C(17) | C(10) | Er(1) | 72.7(9) |
| C(10) | C(11) | Er(1) | 74.8(10) |
| C(10) | C(11) | C(12) | 137.6(15) |
| C(12) | C(11) | Er(1) | 74.9(9) |
| C(11) | C(12) | Er(1) | 72.5(9) |
| C(13) | C(12) | Er(1) | 76.6(9) |
| C(13) | C(12) | C(11) | 133.8(15) |
| C(12) | C(13) | Er(1) | 71.5(9) |
| C(12) | C(13) | C(14) | 133.1(16) |
| C(14) | C(13) | Er(1) | 71.1(12) |
| C(13) | C(14) | Er(1) | 76.9(12) |

| | | | |
|---|---|---|---|
| C(13) | C(14) | C(15) | 137(2) |
| C(15) | C(14) | Er(1) | 71.6(18) |
| C(14) | C(15) | Er(1) | 75.1(18) |
| C(14) | C(15) | C(16) | 135(2) |
| C(16) | C(15) | Er(1) | 71.8(15) |
| C(15) | C(16) | Er(1) | 74.3(16) |
| C(17) | C(16) | Er(1) | 75.6(12) |
| C(17) | C(16) | C(15) | 134.0(19) |
| C(10) | C(17) | Er(1) | 74.5(9) |
| C(10) | C(17) | C(16) | 133.5(16) |
| C(16) | C(17) | Er(1) | 71.1(12) |

**Table S20.** Bond lengths (Å) and angles (deg) for [Tm(Cnt)(Cot)] **(5)** at 150 K. (Continuing over several pages).

| Atom A | Atom B | Distance(esd) |
|---|---|---|
| Tm(1) | C(1) | 2.54(3) |
| Tm(1) | C(2) | 2.64(2) |
| Tm(1) | C(3) | 2.875(13) |
| Tm(1) | C(4) | 3.449(9) |
| Tm(1) | C(5) | 3.769(8) |
| Tm(1) | C(6) | 3.594(12) |
| Tm(1) | C(7) | 3.106(18) |
| Tm(1) | C(8) | 2.73(2) |
| Tm(1) | C(9) | 2.57(2) |
| Tm(1) | C(10) | 2.467(8) |
| Tm(1) | C(11) | 2.479(12) |
| Tm(1) | C(12) | 2.46(2) |
| Tm(1) | C(13) | 2.54(3) |
| Tm(1) | C(14) | 2.50(3) |
| Tm(1) | C(15) | 2.43(3) |
| Tm(1) | C(16) | 2.45(2) |
| Tm(1) | C(17) | 2.456(12) |
| C(1) | C(2) | 1.385(12) |
| C(1) | C(9) | 1.392(12) |
| C(2) | C(3) | 1.396(12) |
| C(3) | C(4) | 1.386(9) |
| C(4) | C(5) | 1.383(9) |
| C(5) | C(6) | 1.372(9) |
| C(6) | C(7) | 1.363(10) |
| C(7) | C(8) | 1.395(11) |
| C(8) | C(9) | 1.384(11) |
| C(10) | C(11) | 1.377(11) |
| C(10) | C(17) | 1.386(11) |
| C(11) | C(12) | 1.398(12) |
| C(12) | C(13) | 1.383(13) |
| C(13) | C(14) | 1.395(13) |
| C(14) | C(15) | 1.390(13) |
| C(15) | C(16) | 1.394(13) |
| C(16) | C(17) | 1.384(12) |

| Atom A | Atom B | Atom C | Angle(esd) |
|---|---|---|---|
| C(1) | Tm(1) | C(2) | 31.0(3) |
| C(1) | Tm(1) | C(3) | 56.7(5) |
| C(1) | Tm(1) | C(4) | 70.0(6) |
| C(1) | Tm(1) | C(5) | 75.0(6) |
| C(1) | Tm(1) | C(6) | 77.5(7) |
| C(1) | Tm(1) | C(7) | 75.0(6) |
| C(1) | Tm(1) | C(8) | 58.5(5) |
| C(1) | Tm(1) | C(9) | 31.6(3) |
| C(2) | Tm(1) | C(3) | 28.9(3) |
| C(2) | Tm(1) | C(4) | 48.9(4) |
| C(2) | Tm(1) | C(5) | 63.4(4) |
| C(2) | Tm(1) | C(6) | 76.6(5) |
| C(2) | Tm(1) | C(7) | 85.8(5) |
| C(2) | Tm(1) | C(8) | 80.5(6) |

| | | | |
|---|---|---|---|
| C(3) | Tm(1) | C(4) | 23.1(2) |
| C(3) | Tm(1) | C(5) | 43.1(2) |
| C(3) | Tm(1) | C(6) | 62.4(2) |
| C(3) | Tm(1) | C(7) | 79.8(3) |
| C(4) | Tm(1) | C(5) | 21.50(14) |
| C(4) | Tm(1) | C(6) | 42.54(19) |
| C(6) | Tm(1) | C(5) | 21.31(16) |
| C(7) | Tm(1) | C(4) | 63.0(2) |
| C(7) | Tm(1) | C(5) | 42.7(2) |
| C(7) | Tm(1) | C(6) | 21.96(19) |
| C(8) | Tm(1) | C(3) | 87.8(5) |
| C(8) | Tm(1) | C(4) | 79.0(4) |
| C(8) | Tm(1) | C(5) | 63.0(4) |
| C(8) | Tm(1) | C(6) | 45.7(3) |
| C(8) | Tm(1) | C(7) | 26.7(3) |
| C(9) | Tm(1) | C(2) | 60.2(5) |
| C(9) | Tm(1) | C(3) | 79.3(6) |
| C(9) | Tm(1) | C(4) | 82.3(6) |
| C(9) | Tm(1) | C(5) | 75.6(6) |
| C(9) | Tm(1) | C(6) | 66.4(5) |
| C(9) | Tm(1) | C(7) | 53.7(4) |
| C(9) | Tm(1) | C(8) | 30.0(3) |
| C(10) | Tm(1) | C(11) | 32.3(3) |
| C(10) | Tm(1) | C(13) | 85.4(7) |
| C(10) | Tm(1) | C(14) | 94.0(8) |
| C(11) | Tm(1) | C(13) | 62.1(6) |
| C(11) | Tm(1) | C(14) | 84.1(7) |
| C(12) | Tm(1) | C(10) | 62.8(4) |
| C(12) | Tm(1) | C(11) | 32.9(3) |
| C(12) | Tm(1) | C(13) | 32.1(3) |
| C(12) | Tm(1) | C(14) | 61.5(6) |
| C(14) | Tm(1) | C(13) | 32.2(3) |
| C(15) | Tm(1) | C(10) | 85.6(5) |
| C(15) | Tm(1) | C(11) | 93.7(5) |
| C(15) | Tm(1) | C(12) | 85.0(6) |
| C(15) | Tm(1) | C(13) | 62.5(6) |
| C(15) | Tm(1) | C(14) | 32.7(4) |
| C(15) | Tm(1) | C(16) | 33.2(4) |
| C(15) | Tm(1) | C(17) | 63.6(5) |
| C(16) | Tm(1) | C(10) | 62.1(4) |
| C(16) | Tm(1) | C(11) | 84.2(4) |
| C(16) | Tm(1) | C(12) | 94.0(5) |
| C(16) | Tm(1) | C(13) | 85.4(8) |
| C(16) | Tm(1) | C(14) | 62.8(6) |
| C(16) | Tm(1) | C(17) | 32.8(3) |
| C(17) | Tm(1) | C(10) | 32.7(3) |
| C(17) | Tm(1) | C(11) | 62.4(4) |
| C(17) | Tm(1) | C(12) | 86.4(5) |
| C(17) | Tm(1) | C(13) | 95.6(9) |
| C(17) | Tm(1) | C(14) | 86.3(7) |
| C(2) | C(1) | Tm(1) | 78.5(13) |
| C(2) | C(1) | C(9) | 141(2) |
| C(9) | C(1) | Tm(1) | 75.6(14) |
| C(1) | C(2) | Tm(1) | 70.6(14) |

| | | | |
|---|---|---|---|
| C(1) | C(2) | C(3) | 137.0(19) |
| C(3) | C(2) | Tm(1) | 85.0(11) |
| C(2) | C(3) | Tm(1) | 66.1(10) |
| C(4) | C(3) | Tm(1) | 102.4(8) |
| C(4) | C(3) | C(2) | 141.2(15) |
| C(3) | C(4) | Tm(1) | 54.5(7) |
| C(5) | C(4) | Tm(1) | 92.4(5) |
| C(5) | C(4) | C(3) | 137.1(9) |
| C(4) | C(5) | Tm(1) | 66.1(4) |
| C(6) | C(5) | Tm(1) | 72.1(5) |
| C(6) | C(5) | C(4) | 136.5(8) |
| C(5) | C(6) | Tm(1) | 86.5(5) |
| C(7) | C(6) | Tm(1) | 58.5(9) |
| C(7) | C(6) | C(5) | 141.0(12) |
| C(6) | C(7) | Tm(1) | 99.6(10) |
| C(6) | C(7) | C(8) | 139.0(13) |
| C(8) | C(7) | Tm(1) | 61.5(11) |
| C(7) | C(8) | Tm(1) | 91.8(13) |
| C(9) | C(8) | Tm(1) | 68.7(12) |
| C(9) | C(8) | C(7) | 139.5(17) |
| C(1) | C(9) | Tm(1) | 72.8(14) |
| C(8) | C(9) | Tm(1) | 81.3(12) |
| C(8) | C(9) | C(1) | 136.9(19) |
| C(11) | C(10) | Tm(1) | 74.3(6) |
| C(11) | C(10) | C(17) | 135.5(10) |
| C(17) | C(10) | Tm(1) | 73.2(6) |
| C(10) | C(11) | Tm(1) | 73.4(6) |
| C(10) | C(11) | C(12) | 135.6(12) |
| C(12) | C(11) | Tm(1) | 72.9(10) |
| C(11) | C(12) | Tm(1) | 74.2(9) |
| C(13) | C(12) | Tm(1) | 76.9(14) |
| C(13) | C(12) | C(11) | 136.8(18) |
| C(12) | C(13) | Tm(1) | 71.0(13) |
| C(12) | C(13) | C(14) | 132(2) |
| C(14) | C(13) | Tm(1) | 72.4(15) |
| C(13) | C(14) | Tm(1) | 75.5(15) |
| C(15) | C(14) | Tm(1) | 71.1(16) |
| C(15) | C(14) | C(13) | 136(2) |
| C(14) | C(15) | Tm(1) | 76.2(16) |
| C(14) | C(15) | C(16) | 136(2) |
| C(16) | C(15) | Tm(1) | 74.2(15) |
| C(15) | C(16) | Tm(1) | 72.6(16) |
| C(17) | C(16) | Tm(1) | 73.7(10) |
| C(17) | C(16) | C(15) | 136.1(19) |
| C(10) | C(17) | Tm(1) | 74.1(5) |
| C(16) | C(17) | Tm(1) | 73.5(11) |
| C(16) | C(17) | C(10) | 132.5(13) |

**Table S21.** Bond lengths (Å) and angles (deg) for [Tm(Cnt)(Cot)] **(5)** at 300 K. (Continuing over several pages).

| Atom A | Atom B | Distance(esd) |
|---|---|---|
| Tm(1) | C(1) | 2.533(16) |
| Tm(1) | C(2) | 2.613(16) |
| Tm(1) | C(3) | 2.80(3) |
| Tm(1) | C(4) | 3.16(3) |
| Tm(1) | C(5) | 3.496(15) |
| Tm(1) | C(6) | 3.587(13) |
| Tm(1) | C(7) | 3.348(13) |
| Tm(1) | C(8) | 2.888(15) |
| Tm(1) | C(9) | 2.573(15) |
| Tm(1) | C(10) | 2.40(2) |
| Tm(1) | C(11) | 2.42(3) |
| Tm(1) | C(12) | 2.49(2) |
| Tm(1) | C(13) | 2.562(16) |
| Tm(1) | C(14) | 2.521(14) |
| Tm(1) | C(15) | 2.473(14) |
| Tm(1) | C(16) | 2.509(13) |
| Tm(1) | C(17) | 2.452(18) |
| C(1) | C(2) | 1.326(12) |
| C(1) | C(9) | 1.370(11) |
| C(2) | C(3) | 1.333(13) |
| C(3) | C(4) | 1.322(13) |
| C(4) | C(5) | 1.333(12) |
| C(5) | C(6) | 1.360(11) |
| C(6) | C(7) | 1.374(10) |
| C(7) | C(8) | 1.375(10) |
| C(8) | C(9) | 1.392(11) |
| C(10) | C(11) | 1.430(13) |
| C(10) | C(17) | 1.404(13) |
| C(11) | C(12) | 1.428(13) |
| C(12) | C(13) | 1.370(11) |
| C(13) | C(14) | 1.395(11) |
| C(14) | C(15) | 1.408(12) |
| C(15) | C(16) | 1.401(11) |
| C(16) | C(17) | 1.396(11) |

| Atom A | Atom B | Atom C | Angle(esd) |
|---|---|---|---|
| C(1) | Tm(1) | C(2) | 29.8(3) |
| C(1) | Tm(1) | C(3) | 55.3(5) |
| C(1) | Tm(1) | C(4) | 70.3(6) |
| C(1) | Tm(1) | C(5) | 76.7(5) |
| C(1) | Tm(1) | C(6) | 76.0(4) |
| C(1) | Tm(1) | C(7) | 70.9(4) |
| C(1) | Tm(1) | C(8) | 57.2(4) |
| C(1) | Tm(1) | C(9) | 31.1(3) |
| C(1) | Tm(1) | C(13) | 157.1(6) |
| C(2) | Tm(1) | C(3) | 28.2(4) |
| C(2) | Tm(1) | C(4) | 49.8(5) |
| C(2) | Tm(1) | C(5) | 65.5(5) |
| C(2) | Tm(1) | C(6) | 76.0(4) |
| C(2) | Tm(1) | C(7) | 82.5(4) |

| | | | |
|---|---|---|---|
| C(2) | Tm(1) | C(8) | 78.7(5) |
| C(3) | Tm(1) | C(4) | 24.7(4) |
| C(3) | Tm(1) | C(5) | 45.6(4) |
| C(3) | Tm(1) | C(6) | 63.6(5) |
| C(3) | Tm(1) | C(7) | 79.4(5) |
| C(3) | Tm(1) | C(8) | 87.3(6) |
| C(4) | Tm(1) | C(5) | 22.4(2) |
| C(4) | Tm(1) | C(6) | 43.4(3) |
| C(4) | Tm(1) | C(7) | 63.6(4) |
| C(5) | Tm(1) | C(6) | 22.08(18) |
| C(7) | Tm(1) | C(5) | 44.1(2) |
| C(7) | Tm(1) | C(6) | 22.51(17) |
| C(8) | Tm(1) | C(4) | 79.5(4) |
| C(8) | Tm(1) | C(5) | 64.2(3) |
| C(8) | Tm(1) | C(6) | 45.0(2) |
| C(8) | Tm(1) | C(7) | 24.0(2) |
| C(9) | Tm(1) | C(2) | 58.5(4) |
| C(9) | Tm(1) | C(3) | 77.8(6) |
| C(9) | Tm(1) | C(4) | 82.2(6) |
| C(9) | Tm(1) | C(5) | 76.6(4) |
| C(9) | Tm(1) | C(6) | 64.4(4) |
| C(9) | Tm(1) | C(7) | 49.3(3) |
| C(9) | Tm(1) | C(8) | 28.8(3) |
| C(10) | Tm(1) | C(1) | 115.3(7) |
| C(10) | Tm(1) | C(2) | 137.5(7) |
| C(10) | Tm(1) | C(3) | 163.0(8) |
| C(10) | Tm(1) | C(4) | 172.2(8) |
| C(10) | Tm(1) | C(5) | 150.8(5) |
| C(10) | Tm(1) | C(6) | 131.0(4) |
| C(10) | Tm(1) | C(7) | 112.2(5) |
| C(10) | Tm(1) | C(8) | 98.8(6) |
| C(10) | Tm(1) | C(9) | 100.0(7) |
| C(10) | Tm(1) | C(11) | 34.5(4) |
| C(10) | Tm(1) | C(12) | 65.4(6) |
| C(10) | Tm(1) | C(13) | 87.3(6) |
| C(10) | Tm(1) | C(14) | 96.7(7) |
| C(10) | Tm(1) | C(15) | 86.7(6) |
| C(10) | Tm(1) | C(16) | 63.7(5) |
| C(10) | Tm(1) | C(17) | 33.6(3) |
| C(11) | Tm(1) | C(1) | 134.9(6) |
| C(11) | Tm(1) | C(2) | 164.7(7) |
| C(11) | Tm(1) | C(3) | 162.3(7) |
| C(11) | Tm(1) | C(4) | 137.7(7) |
| C(11) | Tm(1) | C(5) | 117.9(6) |
| C(11) | Tm(1) | C(6) | 102.4(6) |
| C(11) | Tm(1) | C(7) | 90.6(6) |
| C(11) | Tm(1) | C(8) | 89.4(6) |
| C(11) | Tm(1) | C(9) | 106.8(6) |
| C(11) | Tm(1) | C(12) | 33.8(4) |
| C(11) | Tm(1) | C(13) | 62.7(5) |
| C(11) | Tm(1) | C(14) | 85.9(6) |
| C(11) | Tm(1) | C(15) | 95.0(7) |
| C(11) | Tm(1) | C(16) | 87.1(6) |
| C(11) | Tm(1) | C(17) | 64.8(6) |

| | | | |
|---|---|---|---|
| C(12) | Tm(1) | C(1) | 155.1(5) |
| C(12) | Tm(1) | C(2) | 157.0(7) |
| C(12) | Tm(1) | C(3) | 129.7(6) |
| C(12) | Tm(1) | C(4) | 107.2(7) |
| C(12) | Tm(1) | C(5) | 92.4(6) |
| C(12) | Tm(1) | C(6) | 85.5(5) |
| C(12) | Tm(1) | C(7) | 85.7(5) |
| C(12) | Tm(1) | C(8) | 97.9(5) |
| C(12) | Tm(1) | C(9) | 124.9(4) |
| C(12) | Tm(1) | C(13) | 31.4(3) |
| C(12) | Tm(1) | C(14) | 61.1(4) |
| C(12) | Tm(1) | C(16) | 94.9(5) |
| C(13) | Tm(1) | C(2) | 131.7(5) |
| C(13) | Tm(1) | C(3) | 103.9(5) |
| C(13) | Tm(1) | C(4) | 86.9(6) |
| C(13) | Tm(1) | C(5) | 81.4(5) |
| C(13) | Tm(1) | C(6) | 86.3(4) |
| C(13) | Tm(1) | C(7) | 97.9(4) |
| C(13) | Tm(1) | C(8) | 118.3(4) |
| C(13) | Tm(1) | C(9) | 146.8(4) |
| C(14) | Tm(1) | C(1) | 139.0(4) |
| C(14) | Tm(1) | C(2) | 109.3(4) |
| C(14) | Tm(1) | C(3) | 86.8(6) |
| C(14) | Tm(1) | C(4) | 80.9(6) |
| C(14) | Tm(1) | C(5) | 87.7(4) |
| C(14) | Tm(1) | C(6) | 102.6(4) |
| C(14) | Tm(1) | C(7) | 121.3(3) |
| C(14) | Tm(1) | C(8) | 145.1(3) |
| C(14) | Tm(1) | C(9) | 163.1(6) |
| C(14) | Tm(1) | C(13) | 31.8(3) |
| C(15) | Tm(1) | C(1) | 120.6(5) |
| C(15) | Tm(1) | C(2) | 97.4(5) |
| C(15) | Tm(1) | C(3) | 87.5(6) |
| C(15) | Tm(1) | C(4) | 95.1(5) |
| C(15) | Tm(1) | C(5) | 110.8(4) |
| C(15) | Tm(1) | C(6) | 130.6(3) |
| C(15) | Tm(1) | C(7) | 152.3(3) |
| C(15) | Tm(1) | C(8) | 174.6(5) |
| C(15) | Tm(1) | C(9) | 150.4(5) |
| C(15) | Tm(1) | C(12) | 84.2(5) |
| C(15) | Tm(1) | C(13) | 61.6(4) |
| C(15) | Tm(1) | C(14) | 32.7(3) |
| C(15) | Tm(1) | C(16) | 32.7(3) |
| C(16) | Tm(1) | C(1) | 107.7(4) |
| C(16) | Tm(1) | C(2) | 98.7(5) |
| C(16) | Tm(1) | C(3) | 104.0(6) |
| C(16) | Tm(1) | C(4) | 120.9(5) |
| C(16) | Tm(1) | C(5) | 140.9(3) |
| C(16) | Tm(1) | C(6) | 162.6(3) |
| C(16) | Tm(1) | C(7) | 174.9(3) |
| C(16) | Tm(1) | C(8) | 151.2(3) |
| C(16) | Tm(1) | C(9) | 127.2(4) |
| C(16) | Tm(1) | C(13) | 85.1(4) |
| C(16) | Tm(1) | C(14) | 63.1(4) |

| | | | |
|---|---|---|---|
| C(17) | Tm(1) | C(1) | 105.8(5) |
| C(17) | Tm(1) | C(2) | 113.7(6) |
| C(17) | Tm(1) | C(3) | 130.8(6) |
| C(17) | Tm(1) | C(4) | 152.4(5) |
| C(17) | Tm(1) | C(5) | 173.4(4) |
| C(17) | Tm(1) | C(6) | 164.1(3) |
| C(17) | Tm(1) | C(7) | 142.5(3) |
| C(17) | Tm(1) | C(8) | 122.4(4) |
| C(17) | Tm(1) | C(9) | 108.8(5) |
| C(17) | Tm(1) | C(12) | 87.5(6) |
| C(17) | Tm(1) | C(13) | 95.2(5) |
| C(17) | Tm(1) | C(14) | 86.4(5) |
| C(17) | Tm(1) | C(15) | 62.6(4) |
| C(17) | Tm(1) | C(16) | 32.7(3) |
| C(2) | C(1) | Tm(1) | 78.4(9) |
| C(2) | C(1) | C(9) | 140.4(13) |
| C(9) | C(1) | Tm(1) | 76.0(8) |
| C(1) | C(2) | Tm(1) | 71.8(8) |
| C(1) | C(2) | C(3) | 138.3(15) |
| C(3) | C(2) | Tm(1) | 83.6(15) |
| C(2) | C(3) | Tm(1) | 68.2(12) |
| C(4) | C(3) | Tm(1) | 93(2) |
| C(4) | C(3) | C(2) | 138.1(19) |
| C(3) | C(4) | Tm(1) | 62.3(18) |
| C(3) | C(4) | C(5) | 143.4(19) |
| C(5) | C(4) | Tm(1) | 93.3(13) |
| C(4) | C(5) | Tm(1) | 64.3(13) |
| C(4) | C(5) | C(6) | 139.5(14) |
| C(6) | C(5) | Tm(1) | 82.7(8) |
| C(5) | C(6) | Tm(1) | 75.2(7) |
| C(5) | C(6) | C(7) | 140.3(11) |
| C(7) | C(6) | Tm(1) | 68.9(7) |
| C(6) | C(7) | Tm(1) | 88.6(8) |
| C(6) | C(7) | C(8) | 137.1(12) |
| C(8) | C(7) | Tm(1) | 58.9(7) |
| C(7) | C(8) | Tm(1) | 97.1(8) |
| C(7) | C(8) | C(9) | 136.2(13) |
| C(9) | C(8) | Tm(1) | 62.9(7) |
| C(1) | C(9) | Tm(1) | 72.9(8) |
| C(1) | C(9) | C(8) | 142.3(13) |
| C(8) | C(9) | Tm(1) | 88.3(8) |
| C(11) | C(10) | Tm(1) | 73.4(17) |
| C(17) | C(10) | Tm(1) | 75.2(11) |
| C(17) | C(10) | C(11) | 133.9(18) |
| C(10) | C(11) | Tm(1) | 72.0(16) |
| C(12) | C(11) | Tm(1) | 75.9(13) |
| C(12) | C(11) | C(10) | 135.2(19) |
| C(11) | C(12) | Tm(1) | 70.3(14) |
| C(13) | C(12) | Tm(1) | 77.2(9) |
| C(13) | C(12) | C(11) | 135.8(15) |
| C(12) | C(13) | Tm(1) | 71.3(9) |
| C(12) | C(13) | C(14) | 134.1(12) |
| C(14) | C(13) | Tm(1) | 72.4(7) |
| C(13) | C(14) | Tm(1) | 75.7(7) |

| | | | |
|---|---|---|---|
| C(13) | C(14) | C(15) | 133.8(11) |
| C(15) | C(14) | Tm(1) | 71.7(7) |
| C(14) | C(15) | Tm(1) | 75.5(7) |
| C(16) | C(15) | Tm(1) | 75.1(8) |
| C(16) | C(15) | C(14) | 138.9(12) |
| C(15) | C(16) | Tm(1) | 72.2(7) |
| C(17) | C(16) | Tm(1) | 71.4(8) |
| C(17) | C(16) | C(15) | 132.4(12) |
| C(10) | C(17) | Tm(1) | 71.1(11) |
| C(16) | C(17) | Tm(1) | 75.9(8) |
| C(16) | C(17) | C(10) | 135.6(14) |

**Table S22.** Bond lengths (Å) and angles (deg) for [Lu(Cnt)(Cot)] **(6)** at 150 K. (Continuing over several pages).

| Atom A | Atom B | Distance(esd) |
|---|---|---|
| Lu(1) | C(1) | 2.504(19) |
| Lu(1) | C(2) | 2.580(15) |
| Lu(1) | C(3) | 2.943(12) |
| Lu(1) | C(4) | 3.590(10) |
| Lu(1) | C(5) | 3.930(9) |
| Lu(1) | C(6) | 3.719(13) |
| Lu(1) | C(7) | 3.14(2) |
| Lu(1) | C(8) | 2.68(2) |
| Lu(1) | C(9) | 2.541(19) |
| Lu(1) | C(10) | 2.461(10) |
| Lu(1) | C(11) | 2.457(12) |
| Lu(1) | C(12) | 2.465(17) |
| Lu(1) | C(13) | 2.495(19) |
| Lu(1) | C(14) | 2.45(2) |
| Lu(1) | C(15) | 2.44(2) |
| Lu(1) | C(16) | 2.47(2) |
| Lu(1) | C(17) | 2.468(13) |
| C(1) | C(2) | 1.392(11) |
| C(1) | C(9) | 1.378(12) |
| C(2) | C(3) | 1.411(11) |
| C(3) | C(4) | 1.402(9) |
| C(4) | C(5) | 1.411(9) |
| C(5) | C(6) | 1.389(10) |
| C(6) | C(7) | 1.393(11) |
| C(7) | C(8) | 1.377(12) |
| C(8) | C(9) | 1.367(12) |
| C(10) | C(11) | 1.393(11) |
| C(10) | C(17) | 1.393(12) |
| C(11) | C(12) | 1.400(12) |
| C(12) | C(13) | 1.388(11) |
| C(13) | C(14) | 1.408(12) |
| C(14) | C(15) | 1.411(13) |
| C(15) | C(16) | 1.405(12) |
| C(16) | C(17) | 1.384(12) |

| Atom A | Atom B | Atom C | Angle(esd) |
|---|---|---|---|
| C(1) | Lu(1) | C(2) | 31.7(3) |
| C(1) | Lu(1) | C(3) | 57.4(4) |
| C(1) | Lu(1) | C(4) | 69.0(4) |
| C(1) | Lu(1) | C(5) | 73.3(5) |
| C(1) | Lu(1) | C(6) | 76.2(5) |
| C(1) | Lu(1) | C(7) | 74.1(6) |
| C(1) | Lu(1) | C(8) | 58.6(5) |
| C(1) | Lu(1) | C(9) | 31.7(3) |
| C(2) | Lu(1) | C(3) | 28.6(2) |
| C(2) | Lu(1) | C(4) | 47.0(3) |
| C(2) | Lu(1) | C(5) | 61.1(3) |
| C(2) | Lu(1) | C(6) | 74.5(4) |
| C(2) | Lu(1) | C(7) | 84.0(5) |
| C(2) | Lu(1) | C(8) | 80.3(5) |

| | | | |
|---|---|---|---|
| C(3) | Lu(1) | C(4) | 22.1(2) |
| C(3) | Lu(1) | C(5) | 41.8(2) |
| C(3) | Lu(1) | C(6) | 61.0(2) |
| C(3) | Lu(1) | C(7) | 78.3(3) |
| C(4) | Lu(1) | C(5) | 21.00(15) |
| C(4) | Lu(1) | C(6) | 41.51(19) |
| C(6) | Lu(1) | C(5) | 20.68(15) |
| C(7) | Lu(1) | C(4) | 61.4(3) |
| C(7) | Lu(1) | C(5) | 41.4(2) |
| C(7) | Lu(1) | C(6) | 21.3(2) |
| C(8) | Lu(1) | C(3) | 87.4(4) |
| C(8) | Lu(1) | C(4) | 77.5(4) |
| C(8) | Lu(1) | C(5) | 61.3(3) |
| C(8) | Lu(1) | C(6) | 44.6(3) |
| C(8) | Lu(1) | C(7) | 25.9(3) |
| C(9) | Lu(1) | C(2) | 60.6(5) |
| C(9) | Lu(1) | C(3) | 79.7(5) |
| C(9) | Lu(1) | C(4) | 81.0(4) |
| C(9) | Lu(1) | C(5) | 73.8(4) |
| C(9) | Lu(1) | C(6) | 65.1(4) |
| C(9) | Lu(1) | C(7) | 52.8(4) |
| C(9) | Lu(1) | C(8) | 30.2(3) |
| C(10) | Lu(1) | C(12) | 63.7(4) |
| C(10) | Lu(1) | C(13) | 86.0(5) |
| C(10) | Lu(1) | C(16) | 62.7(4) |
| C(10) | Lu(1) | C(17) | 32.8(3) |
| C(11) | Lu(1) | C(10) | 32.9(3) |
| C(11) | Lu(1) | C(12) | 33.0(3) |
| C(11) | Lu(1) | C(13) | 62.3(5) |
| C(11) | Lu(1) | C(16) | 86.2(4) |
| C(11) | Lu(1) | C(17) | 63.2(4) |
| C(12) | Lu(1) | C(13) | 32.5(3) |
| C(12) | Lu(1) | C(16) | 97.1(6) |
| C(12) | Lu(1) | C(17) | 87.5(5) |
| C(14) | Lu(1) | C(10) | 94.4(6) |
| C(14) | Lu(1) | C(11) | 84.9(6) |
| C(14) | Lu(1) | C(12) | 62.8(5) |
| C(14) | Lu(1) | C(13) | 33.1(3) |
| C(14) | Lu(1) | C(16) | 63.9(5) |
| C(14) | Lu(1) | C(17) | 86.1(6) |
| C(15) | Lu(1) | C(10) | 85.9(4) |
| C(15) | Lu(1) | C(11) | 95.1(5) |
| C(15) | Lu(1) | C(12) | 87.4(6) |
| C(15) | Lu(1) | C(13) | 64.3(5) |
| C(15) | Lu(1) | C(14) | 33.6(3) |
| C(15) | Lu(1) | C(16) | 33.3(3) |
| C(15) | Lu(1) | C(17) | 63.0(4) |
| C(16) | Lu(1) | C(13) | 87.8(6) |
| C(16) | Lu(1) | C(17) | 32.6(3) |
| C(17) | Lu(1) | C(13) | 96.2(6) |
| C(2) | C(1) | Lu(1) | 77.1(8) |
| C(9) | C(1) | Lu(1) | 75.6(10) |
| C(9) | C(1) | C(2) | 137.9(14) |
| C(1) | C(2) | Lu(1) | 71.1(9) |

| | | | |
|---|---|---|---|
| C(1) | C(2) | C(3) | 141.5(14) |
| C(3) | C(2) | Lu(1) | 90.1(8) |
| C(2) | C(3) | Lu(1) | 61.2(7) |
| C(4) | C(3) | Lu(1) | 105.9(8) |
| C(4) | C(3) | C(2) | 138.2(12) |
| C(3) | C(4) | Lu(1) | 52.1(7) |
| C(3) | C(4) | C(5) | 137.1(11) |
| C(5) | C(4) | Lu(1) | 93.2(6) |
| C(4) | C(5) | Lu(1) | 65.8(5) |
| C(6) | C(5) | Lu(1) | 71.1(6) |
| C(6) | C(5) | C(4) | 135.7(9) |
| C(5) | C(6) | Lu(1) | 88.3(6) |
| C(5) | C(6) | C(7) | 139.1(13) |
| C(7) | C(6) | Lu(1) | 55.0(10) |
| C(6) | C(7) | Lu(1) | 103.7(12) |
| C(8) | C(7) | Lu(1) | 58.4(11) |
| C(8) | C(7) | C(6) | 141.0(15) |
| C(7) | C(8) | Lu(1) | 95.6(12) |
| C(9) | C(8) | Lu(1) | 69.1(11) |
| C(9) | C(8) | C(7) | 140.3(14) |
| C(1) | C(9) | Lu(1) | 72.7(10) |
| C(8) | C(9) | Lu(1) | 80.7(12) |
| C(8) | C(9) | C(1) | 136.1(13) |
| C(11) | C(10) | Lu(1) | 73.4(6) |
| C(17) | C(10) | Lu(1) | 73.8(7) |
| C(17) | C(10) | C(11) | 135.5(11) |
| C(10) | C(11) | Lu(1) | 73.7(6) |
| C(10) | C(11) | C(12) | 137.0(12) |
| C(12) | C(11) | Lu(1) | 73.8(8) |
| C(11) | C(12) | Lu(1) | 73.2(8) |
| C(13) | C(12) | Lu(1) | 74.9(10) |
| C(13) | C(12) | C(11) | 133.7(15) |
| C(12) | C(13) | Lu(1) | 72.6(9) |
| C(12) | C(13) | C(14) | 132.7(15) |
| C(14) | C(13) | Lu(1) | 71.7(10) |
| C(13) | C(14) | Lu(1) | 75.3(10) |
| C(13) | C(14) | C(15) | 137.5(14) |
| C(15) | C(14) | Lu(1) | 72.9(12) |
| C(14) | C(15) | Lu(1) | 73.6(12) |
| C(16) | C(15) | Lu(1) | 74.4(11) |
| C(16) | C(15) | C(14) | 134.8(15) |
| C(15) | C(16) | Lu(1) | 72.3(11) |
| C(17) | C(16) | Lu(1) | 73.8(10) |
| C(17) | C(16) | C(15) | 133.8(15) |
| C(10) | C(17) | Lu(1) | 73.3(7) |
| C(16) | C(17) | Lu(1) | 73.7(10) |
| C(16) | C(17) | C(10) | 134.9(13) |

**Table S23.** Bond lengths (Å) and angles (deg) for [Lu(Cnt)(Cot)] **(6)** at 300 K. (Continuing over several pages).

| Atom A | Atom B | Distance(esd) |
|---|---|---|
| Lu(1) | C(1) | 2.494(13) |
| Lu(1) | C(2) | 2.579(11) |
| Lu(1) | C(3) | 2.962(12) |
| Lu(1) | C(4) | 3.552(10) |
| Lu(1) | C(5) | 3.880(10) |
| Lu(1) | C(6) | 3.679(12) |
| Lu(1) | C(7) | 3.184(17) |
| Lu(1) | C(8) | 2.768(19) |
| Lu(1) | C(9) | 2.585(13) |
| Lu(1) | C(10) | 2.493(10) |
| Lu(1) | C(11) | 2.456(11) |
| Lu(1) | C(12) | 2.485(13) |
| Lu(1) | C(13) | 2.519(13) |
| Lu(1) | C(14) | 2.423(15) |
| Lu(1) | C(15) | 2.35(2) |
| Lu(1) | C(16) | 2.437(16) |
| Lu(1) | C(17) | 2.506(11) |
| C(1) | C(2) | 1.378(9) |
| C(1) | C(9) | 1.326(11) |
| C(2) | C(3) | 1.405(10) |
| C(3) | C(4) | 1.404(8) |
| C(4) | C(5) | 1.401(8) |
| C(5) | C(6) | 1.389(8) |
| C(6) | C(7) | 1.365(10) |
| C(7) | C(8) | 1.323(12) |
| C(8) | C(9) | 1.318(12) |
| C(10) | C(11) | 1.413(10) |
| C(10) | C(17) | 1.391(10) |
| C(11) | C(12) | 1.428(11) |
| C(12) | C(13) | 1.392(9) |
| C(13) | C(14) | 1.409(11) |
| C(14) | C(15) | 1.455(12) |
| C(15) | C(16) | 1.436(12) |
| C(16) | C(17) | 1.370(10) |

| Atom A | Atom B | Atom C | Angle(esd) |
|---|---|---|---|
| C(1) | Lu(1) | C(2) | 31.5(2) |
| C(1) | Lu(1) | C(3) | 57.0(3) |
| C(1) | Lu(1) | C(4) | 69.7(3) |
| C(1) | Lu(1) | C(5) | 74.1(3) |
| C(1) | Lu(1) | C(6) | 75.1(4) |
| C(1) | Lu(1) | C(7) | 70.3(4) |
| C(1) | Lu(1) | C(8) | 55.8(4) |
| C(1) | Lu(1) | C(9) | 30.2(3) |
| C(2) | Lu(1) | C(3) | 28.3(2) |
| C(2) | Lu(1) | C(4) | 47.6(2) |
| C(2) | Lu(1) | C(5) | 61.6(3) |
| C(2) | Lu(1) | C(6) | 73.7(3) |
| C(2) | Lu(1) | C(7) | 80.7(4) |
| C(2) | Lu(1) | C(8) | 77.3(5) |

| | | | |
|---|---|---|---|
| C(2) | Lu(1) | C(9) | 58.6(4) |
| C(3) | Lu(1) | C(4) | 22.65(17) |
| C(3) | Lu(1) | C(5) | 42.23(19) |
| C(3) | Lu(1) | C(6) | 60.8(2) |
| C(3) | Lu(1) | C(7) | 76.6(3) |
| C(4) | Lu(1) | C(5) | 21.14(12) |
| C(4) | Lu(1) | C(6) | 41.69(16) |
| C(6) | Lu(1) | C(5) | 20.96(12) |
| C(7) | Lu(1) | C(4) | 61.0(2) |
| C(7) | Lu(1) | C(5) | 41.6(2) |
| C(7) | Lu(1) | C(6) | 21.43(17) |
| C(8) | Lu(1) | C(3) | 85.2(4) |
| C(8) | Lu(1) | C(4) | 76.8(3) |
| C(8) | Lu(1) | C(5) | 61.3(3) |
| C(8) | Lu(1) | C(6) | 44.1(3) |
| C(8) | Lu(1) | C(7) | 24.4(3) |
| C(9) | Lu(1) | C(3) | 77.4(4) |
| C(9) | Lu(1) | C(4) | 80.1(4) |
| C(9) | Lu(1) | C(5) | 73.3(4) |
| C(9) | Lu(1) | C(6) | 63.3(4) |
| C(9) | Lu(1) | C(7) | 49.3(4) |
| C(9) | Lu(1) | C(8) | 28.2(3) |
| C(10) | Lu(1) | C(13) | 86.1(4) |
| C(10) | Lu(1) | C(17) | 32.3(2) |
| C(11) | Lu(1) | C(10) | 33.2(2) |
| C(11) | Lu(1) | C(12) | 33.6(3) |
| C(11) | Lu(1) | C(13) | 62.8(3) |
| C(11) | Lu(1) | C(17) | 63.4(3) |
| C(12) | Lu(1) | C(10) | 64.1(3) |
| C(12) | Lu(1) | C(13) | 32.3(2) |
| C(12) | Lu(1) | C(17) | 88.1(4) |
| C(14) | Lu(1) | C(10) | 94.7(5) |
| C(14) | Lu(1) | C(11) | 85.8(5) |
| C(14) | Lu(1) | C(12) | 62.8(4) |
| C(14) | Lu(1) | C(13) | 33.1(3) |
| C(14) | Lu(1) | C(16) | 67.0(4) |
| C(14) | Lu(1) | C(17) | 87.6(5) |
| C(15) | Lu(1) | C(10) | 86.3(5) |
| C(15) | Lu(1) | C(11) | 97.3(5) |
| C(15) | Lu(1) | C(12) | 89.4(5) |
| C(15) | Lu(1) | C(13) | 66.3(4) |
| C(15) | Lu(1) | C(14) | 35.5(3) |
| C(15) | Lu(1) | C(16) | 34.9(3) |
| C(15) | Lu(1) | C(17) | 63.8(4) |
| C(16) | Lu(1) | C(10) | 62.2(3) |
| C(16) | Lu(1) | C(11) | 87.5(4) |
| C(16) | Lu(1) | C(12) | 99.4(5) |
| C(16) | Lu(1) | C(13) | 90.6(4) |
| C(16) | Lu(1) | C(17) | 32.2(3) |
| C(17) | Lu(1) | C(13) | 97.0(4) |
| C(2) | C(1) | Lu(1) | 77.7(6) |
| C(9) | C(1) | Lu(1) | 78.7(7) |
| C(9) | C(1) | C(2) | 138.1(11) |
| C(1) | C(2) | Lu(1) | 70.9(6) |

| | | | |
|---|---|---|---|
| C(1) | C(2) | C(3) | 142.3(11) |
| C(3) | C(2) | Lu(1) | 91.2(7) |
| C(2) | C(3) | Lu(1) | 60.5(6) |
| C(4) | C(3) | Lu(1) | 103.0(8) |
| C(4) | C(3) | C(2) | 138.8(11) |
| C(3) | C(4) | Lu(1) | 54.3(7) |
| C(5) | C(4) | Lu(1) | 92.7(6) |
| C(5) | C(4) | C(3) | 137.0(11) |
| C(4) | C(5) | Lu(1) | 66.1(5) |
| C(6) | C(5) | Lu(1) | 71.3(6) |
| C(6) | C(5) | C(4) | 134.8(10) |
| C(5) | C(6) | Lu(1) | 87.7(6) |
| C(7) | C(6) | Lu(1) | 58.4(8) |
| C(7) | C(6) | C(5) | 140.4(12) |
| C(6) | C(7) | Lu(1) | 100.2(9) |
| C(8) | C(7) | Lu(1) | 59.9(9) |
| C(8) | C(7) | C(6) | 144.6(14) |
| C(7) | C(8) | Lu(1) | 95.7(10) |
| C(9) | C(8) | Lu(1) | 68.2(9) |
| C(9) | C(8) | C(7) | 138.1(13) |
| C(1) | C(9) | Lu(1) | 71.1(7) |
| C(8) | C(9) | Lu(1) | 83.6(10) |
| C(8) | C(9) | C(1) | 138.8(10) |
| C(11) | C(10) | Lu(1) | 72.0(6) |
| C(17) | C(10) | Lu(1) | 74.4(6) |
| C(17) | C(10) | C(11) | 137.0(10) |
| C(10) | C(11) | Lu(1) | 74.9(6) |
| C(10) | C(11) | C(12) | 136.8(10) |
| C(12) | C(11) | Lu(1) | 74.3(6) |
| C(11) | C(12) | Lu(1) | 72.1(6) |
| C(13) | C(12) | Lu(1) | 75.2(7) |
| C(13) | C(12) | C(11) | 133.7(12) |
| C(12) | C(13) | Lu(1) | 72.5(7) |
| C(12) | C(13) | C(14) | 131.9(11) |
| C(14) | C(13) | Lu(1) | 69.7(7) |
| C(13) | C(14) | Lu(1) | 77.2(6) |
| C(13) | C(14) | C(15) | 136.9(11) |
| C(15) | C(14) | Lu(1) | 69.5(9) |
| C(14) | C(15) | Lu(1) | 75.0(9) |
| C(16) | C(15) | Lu(1) | 75.9(9) |
| C(16) | C(15) | C(14) | 136.2(11) |
| C(15) | C(16) | Lu(1) | 69.2(9) |
| C(17) | C(16) | Lu(1) | 76.7(8) |
| C(17) | C(16) | C(15) | 132.4(11) |
| C(10) | C(17) | Lu(1) | 73.3(6) |
| C(16) | C(17) | Lu(1) | 71.2(8) |
| C(16) | C(17) | C(10) | 134.5(10) |

**Table S24.** Bond lengths (Å) and angles (deg) for [Tb(*cis*-Cnt)(*trans*-Cnt)(Cot)] **(1')** at 150 K. (Continuing over several pages).

The 1 after atom name refers to the atom generated by the symmetry operation 1 applied on asymmetric unit atomic coordinates:
+X,1/2-Y,+Z

| Atom A | Atom B | Distance(esd) |
|---|---|---|
| Tb(1) | C(1A) | 2.704(15) |
| Tb(1) | C(2A) | 2.838(6) |
| Tb(1) | C(2A)1 | 2.838(6) |
| Tb(1) | C(3A)1 | 2.846(6) |
| Tb(1) | C(3A) | 2.846(6) |
| Tb(1) | C(4A)1 | 2.812(6) |
| Tb(1) | C(4A) | 2.812(6) |
| Tb(1) | C(5A)1 | 2.767(6) |
| Tb(1) | C(5A) | 2.767(6) |
| Tb(1) | C(1B) | 2.804(16) |
| Tb(1) | C(2B) | 2.838(6) |
| Tb(1) | C(2B)1 | 2.838(6) |
| Tb(1) | C(3B) | 2.846(6) |
| Tb(1) | C(4B) | 2.812(6) |
| Tb(1) | C(5B) | 2.767(6) |
| Tb(1) | C(5B)1 | 2.767(6) |
| Tb(1) | C(1C) | 2.791(6) |
| Tb(1) | C(2C)1 | 2.850(5) |
| Tb(1) | C(2C) | 2.850(5) |
| Tb(1) | C(3C) | 2.898(5) |
| Tb(1) | C(3C)1 | 2.898(5) |
| Tb(1) | C(4C) | 2.881(5) |
| Tb(1) | C(4C)1 | 2.881(5) |
| Tb(1) | C(5C) | 2.826(5) |
| Tb(1) | C(5C)1 | 2.826(5) |
| Tb(1) | C(6A)1 | 2.573(3) |
| Tb(1) | C(6A) | 2.573(3) |
| Tb(1) | C(7A) | 2.581(4) |
| Tb(1) | C(7A)1 | 2.581(4) |
| Tb(1) | C(8A) | 2.582(4) |
| Tb(1) | C(8A)1 | 2.582(4) |
| Tb(1) | C(9A) | 2.577(3) |
| Tb(1) | C(9A)1 | 2.577(3) |
| Tb(1) | C(6B) | 2.548(11) |
| Tb(1) | C(7B) | 2.559(8) |
| Tb(1) | C(7B)1 | 2.559(8) |
| Tb(1) | C(8B) | 2.559(8) |
| Tb(1) | C(8B)1 | 2.559(8) |
| Tb(1) | C(9B)1 | 2.576(7) |
| Tb(1) | C(9B) | 2.576(7) |
| Tb(1) | C(10B) | 2.561(10) |
| C(1A) | C(2A) | 1.408(8) |
| C(1A) | C(2A)1 | 1.408(8) |
| C(2A) | C(3A) | 1.386(7) |
| C(3A) | C(4A) | 1.384(7) |
| C(4A) | C(5A) | 1.390(7) |

| | | |
|---|---|---|
| C(5A) | C(5A)1 | 1.390(9) |
| C(1B) | C(2B)1 | 1.370(8) |
| C(1B) | C(2B) | 1.370(8) |
| C(2B) | C(3B) | 1.386(7) |
| C(3B) | C(4B) | 1.384(7) |
| C(4B) | C(5B) | 1.390(7) |
| C(5B) | C(5B)1 | 1.390(9) |
| C(1C) | C(2C) | 1.398(6) |
| C(1C) | C(2C)1 | 1.398(6) |
| C(2C) | C(3C) | 1.382(7) |
| C(3C) | C(4C) | 1.396(7) |
| C(4C) | C(5C) | 1.393(8) |
| C(5C) | C(5C)1 | 1.391(11) |
| C(6A) | C(6A)1 | 1.401(10) |
| C(6A) | C(7A) | 1.396(7) |
| C(7A) | C(8A) | 1.398(7) |
| C(8A) | C(9A) | 1.419(7) |
| C(9A) | C(9A)1 | 1.455(12) |
| C(6B) | C(7B)1 | 1.401(12) |
| C(6B) | C(7B) | 1.401(12) |
| C(7B) | C(8B) | 1.366(13) |
| C(8B) | C(9B) | 1.380(13) |
| C(9B) | C(10B) | 1.456(12) |

| Atom A | Atom B | Atom C | Angle(esd) |
|---|---|---|---|
| C(1A) | Tb(1) | C(2A)1 | 29.31(17) |
| C(1A) | Tb(1) | C(2A) | 29.31(17) |
| C(1A) | Tb(1) | C(3A)1 | 47.93(17) |
| C(1A) | Tb(1) | C(3A) | 47.93(17) |
| C(1A) | Tb(1) | C(4A)1 | 62.4(3) |
| C(1A) | Tb(1) | C(4A) | 62.4(3) |
| C(1A) | Tb(1) | C(5A) | 70.3(4) |
| C(1A) | Tb(1) | C(5A)1 | 70.3(4) |
| C(1A) | Tb(1) | C(2B)1 | 29.31(17) |
| C(1A) | Tb(1) | C(5B)1 | 70.3(4) |
| C(1A) | Tb(1) | C(2C)1 | 69.0(4) |
| C(1A) | Tb(1) | C(3C)1 | 55.7(2) |
| C(1A) | Tb(1) | C(4C)1 | 38.09(15) |
| C(1A) | Tb(1) | C(5C)1 | 20.9(3) |
| C(2A)1 | Tb(1) | C(2A) | 54.4(2) |
| C(2A) | Tb(1) | C(3A) | 28.23(14) |
| C(2A)1 | Tb(1) | C(3A) | 77.1(2) |
| C(2A) | Tb(1) | C(3A)1 | 77.1(2) |
| C(2A)1 | Tb(1) | C(3A)1 | 28.23(14) |
| C(2A)1 | Tb(1) | C(2B)1 | 0.0(4) |
| C(2A)1 | Tb(1) | C(2C)1 | 67.53(18) |
| C(2A) | Tb(1) | C(2C)1 | 92.35(18) |
| C(2A) | Tb(1) | C(3C)1 | 84.54(18) |
| C(2A)1 | Tb(1) | C(3C)1 | 42.96(17) |
| C(2A)1 | Tb(1) | C(4C)1 | 15.43(14) |
| C(2A) | Tb(1) | C(4C)1 | 66.4(2) |
| C(3A) | Tb(1) | C(3A)1 | 92.0(3) |
| C(3A)1 | Tb(1) | C(2C)1 | 43.81(17) |

| | | | |
|---|---|---|---|
| C(3A) | Tb(1) | C(2C)1 | 86.8(2) |
| C(3A) | Tb(1) | C(3C)1 | 92.44(17) |
| C(3A)1 | Tb(1) | C(3C)1 | 16.27(14) |
| C(3A)1 | Tb(1) | C(4C)1 | 12.81(14) |
| C(3A) | Tb(1) | C(4C)1 | 84.95(19) |
| C(4A)1 | Tb(1) | C(2A)1 | 54.15(19) |
| C(4A) | Tb(1) | C(2A) | 54.15(18) |
| C(4A) | Tb(1) | C(2A)1 | 89.7(2) |
| C(4A)1 | Tb(1) | C(2A) | 89.7(2) |
| C(4A) | Tb(1) | C(3A) | 28.31(15) |
| C(4A)1 | Tb(1) | C(3A)1 | 28.31(15) |
| C(4A) | Tb(1) | C(3A)1 | 92.0(2) |
| C(4A)1 | Tb(1) | C(3A) | 92.0(2) |
| C(4A) | Tb(1) | C(4A)1 | 79.0(3) |
| C(4A) | Tb(1) | C(2B)1 | 89.7(2) |
| C(4A)1 | Tb(1) | C(2B)1 | 54.15(19) |
| C(4A) | Tb(1) | C(2C)1 | 68.2(2) |
| C(4A)1 | Tb(1) | C(2C)1 | 16.04(15) |
| C(4A)1 | Tb(1) | C(3C)1 | 12.05(14) |
| C(4A) | Tb(1) | C(3C)1 | 84.72(16) |
| C(4A) | Tb(1) | C(4C)1 | 90.78(19) |
| C(4A)1 | Tb(1) | C(4C)1 | 39.61(17) |
| C(4A) | Tb(1) | C(5C)1 | 83.31(19) |
| C(4A)1 | Tb(1) | C(5C)1 | 64.57(18) |
| C(5A)1 | Tb(1) | C(2A)1 | 75.6(2) |
| C(5A) | Tb(1) | C(2A)1 | 88.9(2) |
| C(5A) | Tb(1) | C(2A) | 75.6(2) |
| C(5A)1 | Tb(1) | C(2A) | 88.9(2) |
| C(5A) | Tb(1) | C(3A)1 | 77.8(2) |
| C(5A) | Tb(1) | C(3A) | 55.11(19) |
| C(5A)1 | Tb(1) | C(3A)1 | 55.11(19) |
| C(5A)1 | Tb(1) | C(3A) | 77.8(2) |
| C(5A)1 | Tb(1) | C(4A) | 56.2(2) |
| C(5A) | Tb(1) | C(4A)1 | 56.2(2) |
| C(5A)1 | Tb(1) | C(4A)1 | 28.83(15) |
| C(5A) | Tb(1) | C(4A) | 28.83(15) |
| C(5A)1 | Tb(1) | C(5A) | 29.10(19) |
| C(5A) | Tb(1) | C(2B)1 | 88.9(2) |
| C(5A)1 | Tb(1) | C(2B)1 | 75.6(2) |
| C(5A)1 | Tb(1) | C(2C)1 | 13.44(13) |
| C(5A) | Tb(1) | C(2C)1 | 42.28(17) |
| C(5A) | Tb(1) | C(3C)1 | 65.33(16) |
| C(5A)1 | Tb(1) | C(3C)1 | 39.87(15) |
| C(5A)1 | Tb(1) | C(4C)1 | 63.86(17) |
| C(5A) | Tb(1) | C(4C)1 | 82.48(16) |
| C(5A) | Tb(1) | C(5C)1 | 88.94(18) |
| C(5A)1 | Tb(1) | C(5C)1 | 81.83(18) |
| C(1B) | Tb(1) | C(2B) | 28.09(15) |
| C(1B) | Tb(1) | C(3B) | 54.8(2) |
| C(1B) | Tb(1) | C(4B) | 76.1(3) |
| C(2B)1 | Tb(1) | C(2B) | 54.4(2) |
| C(2B)1 | Tb(1) | C(3B) | 77.1(2) |
| C(2B) | Tb(1) | C(3B) | 28.23(14) |
| C(4B) | Tb(1) | C(2B) | 54.15(18) |

| | | | |
|---|---|---|---|
| C(4B) | Tb(1) | C(3B) | 28.31(15) |
| C(5B) | Tb(1) | C(1B) | 88.4(4) |
| C(5B)1 | Tb(1) | C(1B) | 88.4(4) |
| C(5B) | Tb(1) | C(2B) | 75.6(2) |
| C(5B)1 | Tb(1) | C(2B) | 88.9(2) |
| C(5B)1 | Tb(1) | C(3B) | 77.8(2) |
| C(5B) | Tb(1) | C(3B) | 55.11(19) |
| C(5B) | Tb(1) | C(4B) | 28.83(15) |
| C(5B)1 | Tb(1) | C(4B) | 56.2(2) |
| C(5B)1 | Tb(1) | C(5B) | 29.10(19) |
| C(1C) | Tb(1) | C(2C) | 28.67(12) |
| C(1C) | Tb(1) | C(3C) | 54.54(14) |
| C(1C) | Tb(1) | C(4C) | 76.37(16) |
| C(1C) | Tb(1) | C(5C) | 89.70(18) |
| C(2C)1 | Tb(1) | C(2C) | 55.2(2) |
| C(2C)1 | Tb(1) | C(3C) | 76.15(17) |
| C(2C) | Tb(1) | C(3C) | 27.80(14) |
| C(2C) | Tb(1) | C(4C) | 54.09(15) |
| C(2C)1 | Tb(1) | C(4C) | 89.11(15) |
| C(4C)1 | Tb(1) | C(3C) | 88.61(18) |
| C(4C) | Tb(1) | C(3C) | 27.97(14) |
| C(4C)1 | Tb(1) | C(4C) | 76.1(3) |
| C(5C)1 | Tb(1) | C(2C) | 89.30(16) |
| C(5C) | Tb(1) | C(2C) | 76.11(15) |
| C(5C) | Tb(1) | C(3C) | 54.31(16) |
| C(5C)1 | Tb(1) | C(3C) | 76.08(17) |
| C(5C)1 | Tb(1) | C(4C) | 54.7(2) |
| C(5C) | Tb(1) | C(4C) | 28.23(15) |
| C(5C)1 | Tb(1) | C(5C) | 28.5(2) |
| C(6A)1 | Tb(1) | C(7A) | 60.2(2) |
| C(6A) | Tb(1) | C(7A) | 31.42(16) |
| C(6A) | Tb(1) | C(8A) | 60.22(16) |
| C(6A)1 | Tb(1) | C(8A) | 82.15(15) |
| C(6A)1 | Tb(1) | C(9A) | 91.69(13) |
| C(6A) | Tb(1) | C(9A) | 82.86(14) |
| C(6A)1 | Tb(1) | C(9B)1 | 73.6(3) |
| C(6A) | Tb(1) | C(9B)1 | 90.1(2) |
| C(7A) | Tb(1) | C(8A) | 31.43(16) |
| C(7A)1 | Tb(1) | C(8A) | 90.76(13) |
| C(8A)1 | Tb(1) | C(8A) | 82.8(2) |
| C(9A)1 | Tb(1) | C(7A) | 83.19(15) |
| C(9A) | Tb(1) | C(7A) | 60.76(17) |
| C(9A)1 | Tb(1) | C(8A) | 61.6(2) |
| C(9A) | Tb(1) | C(8A) | 31.94(17) |
| C(6B) | Tb(1) | C(7B) | 31.8(3) |
| C(6B) | Tb(1) | C(8B) | 60.2(3) |
| C(6B) | Tb(1) | C(9B)1 | 82.4(3) |
| C(6B) | Tb(1) | C(9B) | 82.4(3) |
| C(6B) | Tb(1) | C(10B) | 91.7(4) |
| C(7B) | Tb(1) | C(8B) | 31.0(3) |
| C(7B)1 | Tb(1) | C(8B) | 81.9(3) |
| C(7B) | Tb(1) | C(9B)1 | 91.2(3) |
| C(7B) | Tb(1) | C(9B) | 59.7(3) |
| C(7B)1 | Tb(1) | C(9B) | 91.2(3) |

| | | | |
|---|---|---|---|
| C(7B)1 | Tb(1) | C(9B)1 | 59.7(3) |
| C(7B)1 | Tb(1) | C(10B) | 82.6(3) |
| C(7B) | Tb(1) | C(10B) | 82.6(3) |
| C(8B) | Tb(1) | C(9B)1 | 83.3(3) |
| C(8B)1 | Tb(1) | C(9B)1 | 31.2(3) |
| C(8B)1 | Tb(1) | C(9B) | 83.3(3) |
| C(8B) | Tb(1) | C(9B) | 31.2(3) |
| C(8B)1 | Tb(1) | C(10B) | 61.1(3) |
| C(8B) | Tb(1) | C(10B) | 61.1(3) |
| C(9B) | Tb(1) | C(9B)1 | 62.7(5) |
| C(10B) | Tb(1) | C(9B) | 32.9(3) |
| C(10B) | Tb(1) | C(9B)1 | 32.9(3) |
| C(2A)1 | C(1A) | Tb(1) | 80.6(6) |
| C(2A) | C(1A) | Tb(1) | 80.6(6) |
| C(2A) | C(1A) | C(2A)1 | 134.2(14) |
| C(1A) | C(2A) | Tb(1) | 70.0(7) |
| C(3A) | C(2A) | Tb(1) | 76.2(4) |
| C(3A) | C(2A) | C(1A) | 107.8(9) |
| C(2A) | C(3A) | Tb(1) | 75.6(4) |
| C(4A) | C(3A) | Tb(1) | 74.5(4) |
| C(4A) | C(3A) | C(2A) | 136.3(7) |
| C(3A) | C(4A) | Tb(1) | 77.2(4) |
| C(3A) | C(4A) | C(5A) | 138.9(7) |
| C(5A) | C(4A) | Tb(1) | 73.8(4) |
| C(4A) | C(5A) | Tb(1) | 77.4(4) |
| C(4A) | C(5A) | C(5A)1 | 141.9(4) |
| C(5A)1 | C(5A) | Tb(1) | 75.45(10) |
| C(2B) | C(1B) | Tb(1) | 77.3(7) |
| C(2B)1 | C(1B) | Tb(1) | 77.3(7) |
| C(2B)1 | C(1B) | C(2B) | 142.5(16) |
| C(1B) | C(2B) | Tb(1) | 74.6(7) |
| C(1B) | C(2B) | C(3B) | 141.5(10) |
| C(3B) | C(2B) | Tb(1) | 76.2(4) |
| C(2B) | C(3B) | Tb(1) | 75.6(4) |
| C(4B) | C(3B) | Tb(1) | 74.5(4) |
| C(4B) | C(3B) | C(2B) | 136.3(7) |
| C(3B) | C(4B) | Tb(1) | 77.2(4) |
| C(3B) | C(4B) | C(5B) | 138.9(7) |
| C(5B) | C(4B) | Tb(1) | 73.8(4) |
| C(4B) | C(5B) | Tb(1) | 77.4(4) |
| C(5B)1 | C(5B) | Tb(1) | 75.45(10) |
| C(2C)1 | C(1C) | Tb(1) | 78.0(3) |
| C(2C) | C(1C) | Tb(1) | 78.0(3) |
| C(2C)1 | C(1C) | C(2C) | 141.5(7) |
| C(1C) | C(2C) | Tb(1) | 73.3(3) |
| C(3C) | C(2C) | Tb(1) | 78.0(3) |
| C(3C) | C(2C) | C(1C) | 139.5(6) |
| C(2C) | C(3C) | Tb(1) | 74.2(3) |
| C(2C) | C(3C) | C(4C) | 139.5(6) |
| C(4C) | C(3C) | Tb(1) | 75.3(3) |
| C(3C) | C(4C) | Tb(1) | 76.7(3) |
| C(5C) | C(4C) | Tb(1) | 73.7(3) |
| C(5C) | C(4C) | C(3C) | 139.1(5) |
| C(4C) | C(5C) | Tb(1) | 78.1(3) |

| | | | |
|---|---|---|---|
| C(5C)1 | C(5C) | Tb(1) | 75.76(11) |
| C(5C)1 | C(5C) | C(4C) | 140.9(3) |
| C(6A)1 | C(6A) | Tb(1) | 74.20(12) |
| C(7A) | C(6A) | Tb(1) | 74.6(2) |
| C(6A) | C(7A) | Tb(1) | 73.9(2) |
| C(6A) | C(7A) | C(8A) | 135.5(4) |
| C(8A) | C(7A) | Tb(1) | 74.3(2) |
| C(7A) | C(8A) | Tb(1) | 74.2(2) |
| C(7A) | C(8A) | C(9A) | 135.6(4) |
| C(9A) | C(8A) | Tb(1) | 73.8(2) |
| C(8A) | C(9A) | Tb(1) | 74.3(2) |
| C(9A)1 | C(9A) | Tb(1) | 73.60(14) |
| C(7B)1 | C(6B) | Tb(1) | 74.5(6) |
| C(7B) | C(6B) | Tb(1) | 74.5(6) |
| C(7B)1 | C(6B) | C(7B) | 134.3(12) |
| C(6B) | C(7B) | Tb(1) | 73.6(6) |
| C(8B) | C(7B) | Tb(1) | 74.5(5) |
| C(8B) | C(7B) | C(6B) | 135.5(9) |
| C(7B) | C(8B) | Tb(1) | 74.5(5) |
| C(7B) | C(8B) | C(9B) | 137.1(8) |
| C(9B) | C(8B) | Tb(1) | 75.1(5) |
| C(8B) | C(9B) | Tb(1) | 73.7(5) |
| C(8B) | C(9B) | C(10B) | 133.2(9) |
| C(10B) | C(9B) | Tb(1) | 72.9(5) |
| C(9B) | C(10B) | Tb(1) | 74.1(5) |
| C(9B)1 | C(10B) | Tb(1) | 74.1(5) |
| C(9B)1 | C(10B) | C(9B) | 134.1(12) |

**Table S25.** Bond lengths (Å) and angles (deg) for [Tb(*cis*-Cnt)(*trans*-Cnt)(Cot)] **(1'')** at XX K. (Continuing over several pages).

| Atom | Atom | Length/Å |
|---|---|---|
| C(10) | C(11) | 1.362(10) |
| C(10) | C(17) | 1.375(10) |
| C(11) | C(12) | 1.380(9) |
| C(12) | C(13) | 1.355(10) |
| C(13) | C(14) | 1.379(10) |
| C(14) | C(15) | 1.413(12) |
| C(15) | C(16) | 1.446(14) |
| C(16) | C(17) | 1.407(12) |
| C(1A) | C(2A) | 1.44(2) |
| C(1A) | C(9A) | 1.12(2) |
| C(2A) | C(3A) | 1.374(18) |
| C(3A) | C(4A) | 1.408(16) |
| C(4A) | C(5A) | 1.409(15) |
| C(5A) | C(6A) | 1.436(15) |
| C(6A) | C(7A) | 1.379(16) |
| C(7A) | C(8A) | 1.363(18) |
| C(8A) | C(9A) | 1.42(2) |
| C(1B) | C(2B) | 1.4377 |
| C(1B) | C(9B) | 1.4451 |
| C(2B) | C(3B) | 1.3739 |
| C(3B) | C(4B) | 1.4118 |
| C(4B) | C(5B) | 1.4125 |
| C(5B) | C(6B) | 1.4499 |
| C(6B) | C(7B) | 1.4061 |
| C(7B) | C(8B) | 1.3758 |
| C(8B) | C(9B) | 1.3851 |

| Atom | Atom | Atom | Angle/° |
|---|---|---|---|
| C(11) | C(10) | C(17) | 135.4(5) |
| C(10) | C(11) | C(12) | 135.5(5) |
| C(13) | C(12) | C(11) | 135.3(5) |
| C(12) | C(13) | C(14) | 135.8(5) |
| C(13) | C(14) | C(15) | 135.9(5) |
| C(14) | C(15) | C(16) | 132.7(5) |
| C(17) | C(16) | C(15) | 133.1(5) |
| C(10) | C(17) | C(16) | 136.2(5) |
| C(9A) | C(1A) | C(2A) | 142.3(10) |
| C(3A) | C(2A) | C(1A) | 138.8(9) |
| C(2A) | C(3A) | C(4A) | 140.0(11) |
| C(3A) | C(4A) | C(5A) | 138.0(10) |
| C(4A) | C(5A) | C(6A) | 139.7(8) |
| C(7A) | C(6A) | C(5A) | 137.2(10) |
| C(8A) | C(7A) | C(6A) | 141.3(11) |
| C(7A) | C(8A) | C(9A) | 138.8(9) |
| C(1A) | C(9A) | C(8A) | 143.4(10) |
| C(2B) | C(1B) | C(9B) | 124.8 |
| C(3B) | C(2B) | C(1B) | 104.5 |
| C(2B) | C(3B) | C(4B) | 130.9 |
| C(3B) | C(4B) | C(5B) | 139.0 |
| C(4B) | C(5B) | C(6B) | 142.2 |

| | | | |
|---|---|---|---|
| C(7B) | C(6B) | C(5B) | 141.9 |
| C(8B) | C(7B) | C(6B) | 140.1 |
| C(7B) | C(8B) | C(9B) | 130.4 |
| C(8B) | C(9B) | C(1B) | 104.9 |

**Table S26.** Bond lengths (Å) and angles (deg) for [Tm(Cnt)(Cot)] **(5)** at 100 K. (Continuing over several pages).

| Atom | Atom | Length/Å |
|---|---|---|
| Tm(1) | C(1) | 2.54(4) |
| Tm(1) | C(2) | 2.56(4) |
| Tm(1) | C(3) | 2.70(4) |
| Tm(1) | C(4) | 3.09(3) |
| Tm(1) | C(5) | 3.594(16) |
| Tm(1) | C(6) | 3.763(16) |
| Tm(1) | C(7) | 3.454(14) |
| Tm(1) | C(8) | 2.88(2) |
| Tm(1) | C(9) | 2.58(4) |
| Tm(1) | C(10) | 2.458(18) |
| Tm(1) | C(11) | 2.46(4) |
| Tm(1) | C(12) | 2.50(4) |
| Tm(1) | C(13) | 2.52(5) |
| Tm(1) | C(14) | 2.56(4) |
| Tm(1) | C(15) | 2.51(3) |
| Tm(1) | C(16) | 2.49(2) |
| Tm(1) | C(17) | 2.487(14) |
| C(1) | C(2) | 1.384(14) |
| C(1) | C(9) | 1.385(14) |
| C(2) | C(3) | 1.386(14) |
| C(3) | C(4) | 1.382(13) |
| C(4) | C(5) | 1.376(13) |
| C(5) | C(6) | 1.384(12) |
| C(6) | C(7) | 1.383(12) |
| C(7) | C(8) | 1.383(12) |
| C(8) | C(9) | 1.388(14) |
| C(10) | C(11) | 1.404(15) |
| C(10) | C(17) | 1.406(14) |
| C(11) | C(12) | 1.411(15) |
| C(12) | C(13) | 1.415(15) |
| C(13) | C(14) | 1.412(14) |
| C(14) | C(15) | 1.404(14) |
| C(15) | C(16) | 1.403(14) |
| C(16) | C(17) | 1.400(14) |

| Atom | Atom | Atom | Angle/˚ |
|---|---|---|---|
| C(1) | Tm(1) | C(2) | 31.5(5) |
| C(1) | Tm(1) | C(3) | 58.8(7) |
| C(1) | Tm(1) | C(4) | 75.3(8) |
| C(1) | Tm(1) | C(5) | 77.8(8) |
| C(1) | Tm(1) | C(6) | 75.1(7) |
| C(1) | Tm(1) | C(7) | 70.0(7) |
| C(1) | Tm(1) | C(8) | 57.2(7) |
| C(1) | Tm(1) | C(9) | 31.4(5) |
| C(1) | Tm(1) | C(14) | 160.3(15) |
| C(2) | Tm(1) | C(3) | 30.4(4) |
| C(2) | Tm(1) | C(4) | 54.0(7) |
| C(2) | Tm(1) | C(5) | 66.8(9) |
| C(2) | Tm(1) | C(6) | 75.9(10) |
| C(2) | Tm(1) | C(7) | 82.6(10) |

| | | | |
|---|---|---|---|
| C(2) | Tm(1) | C(8) | 79.9(10) |
| C(2) | Tm(1) | C(9) | 60.5(8) |
| C(3) | Tm(1) | C(4) | 26.6(4) |
| C(3) | Tm(1) | C(5) | 45.8(5) |
| C(3) | Tm(1) | C(6) | 63.1(6) |
| C(3) | Tm(1) | C(7) | 79.2(6) |
| C(3) | Tm(1) | C(8) | 88.5(7) |
| C(4) | Tm(1) | C(5) | 22.1(3) |
| C(4) | Tm(1) | C(6) | 43.0(3) |
| C(4) | Tm(1) | C(7) | 63.4(4) |
| C(5) | Tm(1) | C(6) | 21.52(19) |
| C(7) | Tm(1) | C(5) | 42.8(3) |
| C(7) | Tm(1) | C(6) | 21.6(2) |
| C(8) | Tm(1) | C(4) | 80.5(5) |
| C(8) | Tm(1) | C(5) | 62.9(4) |
| C(8) | Tm(1) | C(6) | 43.2(3) |
| C(8) | Tm(1) | C(7) | 23.0(3) |
| C(9) | Tm(1) | C(3) | 80.9(9) |
| C(9) | Tm(1) | C(4) | 85.9(9) |
| C(9) | Tm(1) | C(5) | 76.6(8) |
| C(9) | Tm(1) | C(6) | 63.0(7) |
| C(9) | Tm(1) | C(7) | 48.3(6) |
| C(9) | Tm(1) | C(8) | 28.7(3) |
| C(10) | Tm(1) | C(1) | 103.8(8) |
| C(10) | Tm(1) | C(2) | 112.5(10) |
| C(10) | Tm(1) | C(3) | 131.7(7) |
| C(10) | Tm(1) | C(4) | 154.2(6) |
| C(10) | Tm(1) | C(5) | 175.8(5) |
| C(10) | Tm(1) | C(6) | 162.5(4) |
| C(10) | Tm(1) | C(7) | 141.3(4) |
| C(10) | Tm(1) | C(8) | 121.2(5) |
| C(10) | Tm(1) | C(9) | 106.8(8) |
| C(10) | Tm(1) | C(12) | 63.7(7) |
| C(10) | Tm(1) | C(13) | 86.9(11) |
| C(10) | Tm(1) | C(14) | 95.8(10) |
| C(10) | Tm(1) | C(15) | 86.7(7) |
| C(10) | Tm(1) | C(16) | 63.1(5) |
| C(10) | Tm(1) | C(17) | 33.0(4) |
| C(11) | Tm(1) | C(1) | 112.1(10) |
| C(11) | Tm(1) | C(2) | 135.7(11) |
| C(11) | Tm(1) | C(3) | 163.5(10) |
| C(11) | Tm(1) | C(4) | 169.9(10) |
| C(11) | Tm(1) | C(5) | 149.8(6) |
| C(11) | Tm(1) | C(6) | 130.5(5) |
| C(11) | Tm(1) | C(7) | 111.8(5) |
| C(11) | Tm(1) | C(8) | 97.6(6) |
| C(11) | Tm(1) | C(9) | 97.1(10) |
| C(11) | Tm(1) | C(10) | 33.2(4) |
| C(11) | Tm(1) | C(12) | 33.1(5) |
| C(11) | Tm(1) | C(13) | 63.1(8) |
| C(11) | Tm(1) | C(14) | 85.6(10) |
| C(11) | Tm(1) | C(15) | 94.7(9) |
| C(11) | Tm(1) | C(16) | 85.2(7) |
| C(11) | Tm(1) | C(17) | 62.8(6) |

| | | | |
|---|---|---|---|
| C(12) | Tm(1) | C(1) | 129.3(10) |
| C(12) | Tm(1) | C(2) | 160.7(11) |
| C(12) | Tm(1) | C(3) | 163.5(11) |
| C(12) | Tm(1) | C(4) | 136.9(9) |
| C(12) | Tm(1) | C(5) | 118.4(8) |
| C(12) | Tm(1) | C(6) | 103.1(7) |
| C(12) | Tm(1) | C(7) | 90.2(7) |
| C(12) | Tm(1) | C(8) | 86.4(8) |
| C(12) | Tm(1) | C(9) | 101.6(10) |
| C(12) | Tm(1) | C(13) | 32.7(5) |
| C(12) | Tm(1) | C(14) | 62.2(8) |
| C(12) | Tm(1) | C(15) | 84.9(9) |
| C(13) | Tm(1) | C(1) | 149.8(13) |
| C(13) | Tm(1) | C(2) | 160.3(17) |
| C(13) | Tm(1) | C(3) | 132.2(11) |
| C(13) | Tm(1) | C(4) | 106.9(11) |
| C(13) | Tm(1) | C(5) | 93.6(10) |
| C(13) | Tm(1) | C(6) | 86.3(11) |
| C(13) | Tm(1) | C(7) | 83.9(11) |
| C(13) | Tm(1) | C(8) | 92.9(11) |
| C(13) | Tm(1) | C(9) | 118.5(12) |
| C(13) | Tm(1) | C(14) | 32.3(5) |
| C(14) | Tm(1) | C(2) | 136.1(11) |
| C(14) | Tm(1) | C(3) | 106.0(10) |
| C(14) | Tm(1) | C(4) | 86.1(10) |
| C(14) | Tm(1) | C(5) | 82.5(9) |
| C(14) | Tm(1) | C(6) | 87.0(9) |
| C(14) | Tm(1) | C(7) | 96.1(8) |
| C(14) | Tm(1) | C(8) | 114.0(9) |
| C(14) | Tm(1) | C(9) | 142.7(9) |
| C(15) | Tm(1) | C(1) | 145.5(10) |
| C(15) | Tm(1) | C(2) | 114.1(10) |
| C(15) | Tm(1) | C(3) | 89.6(9) |
| C(15) | Tm(1) | C(4) | 81.3(8) |
| C(15) | Tm(1) | C(5) | 89.9(7) |
| C(15) | Tm(1) | C(6) | 104.2(6) |
| C(15) | Tm(1) | C(7) | 120.7(5) |
| C(15) | Tm(1) | C(8) | 142.5(6) |
| C(15) | Tm(1) | C(9) | 166.5(13) |
| C(15) | Tm(1) | C(13) | 61.8(8) |
| C(15) | Tm(1) | C(14) | 32.1(4) |
| C(16) | Tm(1) | C(1) | 125.6(8) |
| C(16) | Tm(1) | C(2) | 101.0(10) |
| C(16) | Tm(1) | C(3) | 89.9(7) |
| C(16) | Tm(1) | C(4) | 96.0(5) |
| C(16) | Tm(1) | C(5) | 112.8(4) |
| C(16) | Tm(1) | C(6) | 132.1(4) |
| C(16) | Tm(1) | C(7) | 152.0(4) |
| C(16) | Tm(1) | C(8) | 174.9(5) |
| C(16) | Tm(1) | C(9) | 155.3(8) |
| C(16) | Tm(1) | C(12) | 93.8(8) |
| C(16) | Tm(1) | C(13) | 84.6(10) |
| C(16) | Tm(1) | C(14) | 61.9(7) |
| C(16) | Tm(1) | C(15) | 32.6(4) |

| | | | |
|---|---|---|---|
| C(17) | Tm(1) | C(1) | 109.8(7) |
| C(17) | Tm(1) | C(2) | 100.1(10) |
| C(17) | Tm(1) | C(3) | 105.6(7) |
| C(17) | Tm(1) | C(4) | 122.2(5) |
| C(17) | Tm(1) | C(5) | 142.8(4) |
| C(17) | Tm(1) | C(6) | 164.0(4) |
| C(17) | Tm(1) | C(7) | 174.3(3) |
| C(17) | Tm(1) | C(8) | 152.3(4) |
| C(17) | Tm(1) | C(9) | 128.9(7) |
| C(17) | Tm(1) | C(12) | 85.7(7) |
| C(17) | Tm(1) | C(13) | 94.7(11) |
| C(17) | Tm(1) | C(14) | 85.4(9) |
| C(17) | Tm(1) | C(15) | 62.9(6) |
| C(17) | Tm(1) | C(16) | 32.7(3) |
| C(2) | C(1) | Tm(1) | 75(2) |
| C(2) | C(1) | C(9) | 138(3) |
| C(9) | C(1) | Tm(1) | 75.9(18) |
| C(1) | C(2) | Tm(1) | 73(2) |
| C(1) | C(2) | C(3) | 136(3) |
| C(3) | C(2) | Tm(1) | 80(2) |
| C(2) | C(3) | Tm(1) | 69(2) |
| C(4) | C(3) | Tm(1) | 93(2) |
| C(4) | C(3) | C(2) | 141(3) |
| C(3) | C(4) | Tm(1) | 60.7(19) |
| C(5) | C(4) | Tm(1) | 100.1(17) |
| C(5) | C(4) | C(3) | 139(2) |
| C(4) | C(5) | Tm(1) | 57.8(16) |
| C(4) | C(5) | C(6) | 139.7(19) |
| C(6) | C(5) | Tm(1) | 86.2(9) |
| C(5) | C(6) | Tm(1) | 72.3(9) |
| C(7) | C(6) | Tm(1) | 66.6(8) |
| C(7) | C(6) | C(5) | 137.2(14) |
| C(6) | C(7) | Tm(1) | 91.9(8) |
| C(8) | C(7) | Tm(1) | 54.7(11) |
| C(8) | C(7) | C(6) | 137.6(16) |
| C(7) | C(8) | Tm(1) | 102.3(13) |
| C(7) | C(8) | C(9) | 139(2) |
| C(9) | C(8) | Tm(1) | 63.2(16) |
| C(1) | C(9) | Tm(1) | 72.7(18) |
| C(1) | C(9) | C(8) | 141(3) |
| C(8) | C(9) | Tm(1) | 88.0(18) |
| C(11) | C(10) | Tm(1) | 73.4(19) |
| C(11) | C(10) | C(17) | 133(2) |
| C(17) | C(10) | Tm(1) | 74.6(9) |
| C(10) | C(11) | Tm(1) | 73.4(17) |
| C(10) | C(11) | C(12) | 137(3) |
| C(12) | C(11) | Tm(1) | 75(2) |
| C(11) | C(12) | Tm(1) | 72(2) |
| C(11) | C(12) | C(13) | 135(4) |
| C(13) | C(12) | Tm(1) | 75(3) |
| C(12) | C(13) | Tm(1) | 73(3) |
| C(14) | C(13) | Tm(1) | 75(2) |
| C(14) | C(13) | C(12) | 135(4) |
| C(13) | C(14) | Tm(1) | 72(2) |

| | | | |
|---|---|---|---|
| C(15) | C(14) | Tm(1) | 71.9(16) |
| C(15) | C(14) | C(13) | 133(3) |
| C(14) | C(15) | Tm(1) | 76.0(17) |
| C(16) | C(15) | Tm(1) | 72.9(15) |
| C(16) | C(15) | C(14) | 135(3) |
| C(15) | C(16) | Tm(1) | 74.5(15) |
| C(17) | C(16) | Tm(1) | 73.6(11) |
| C(17) | C(16) | C(15) | 137(2) |
| C(10) | C(17) | Tm(1) | 72.4(9) |
| C(16) | C(17) | Tm(1) | 73.7(11) |
| C(16) | C(17) | C(10) | 134.6(17) |

**Table S27.** Bond lengths (Å) and angles (deg) for [Tm(Cnt)(Cot)] **(5)** at 200 K. (Continuing over several pages).

| Atom A | Atom B | Distance(esd) |
|---|---|---|
| Tm(1) | C(1) | 2.53(4) |
| Tm(1) | C(2) | 2.57(3) |
| Tm(1) | C(3) | 2.89(2) |
| Tm(1) | C(4) | 3.408(15) |
| Tm(1) | C(5) | 3.674(17) |
| Tm(1) | C(6) | 3.53(2) |
| Tm(1) | C(7) | 3.05(3) |
| Tm(1) | C(8) | 2.68(3) |
| Tm(1) | C(9) | 2.59(4) |
| Tm(1) | C(10) | 2.490(16) |
| Tm(1) | C(11) | 2.48(2) |
| Tm(1) | C(12) | 2.51(2) |
| Tm(1) | C(13) | 2.57(3) |
| Tm(1) | C(14) | 2.48(4) |
| Tm(1) | C(15) | 2.51(3) |
| Tm(1) | C(16) | 2.48(3) |
| Tm(1) | C(17) | 2.47(2) |
| C(1) | C(2) | 1.370(13) |
| C(1) | C(9) | 1.352(13) |
| C(2) | C(3) | 1.382(12) |
| C(3) | C(4) | 1.385(12) |
| C(4) | C(5) | 1.375(11) |
| C(5) | C(6) | 1.368(12) |
| C(6) | C(7) | 1.351(12) |
| C(7) | C(8) | 1.348(13) |
| C(8) | C(9) | 1.363(13) |
| C(10) | C(11) | 1.396(13) |
| C(10) | C(17) | 1.404(14) |
| C(11) | C(12) | 1.401(13) |
| C(12) | C(13) | 1.391(13) |
| C(13) | C(14) | 1.409(13) |
| C(14) | C(15) | 1.424(14) |
| C(15) | C(16) | 1.413(14) |
| C(16) | C(17) | 1.402(14) |

| Atom A | Atom B | Atom C | Angle(esd) |
|---|---|---|---|
| C(1) | Tm(1) | C(2) | 31.1(4) |
| C(1) | Tm(1) | C(3) | 56.7(6) |
| C(1) | Tm(1) | C(4) | 70.9(6) |
| C(1) | Tm(1) | C(5) | 76.1(7) |
| C(1) | Tm(1) | C(6) | 77.7(8) |
| C(1) | Tm(1) | C(7) | 74.2(7) |
| C(1) | Tm(1) | C(8) | 57.7(6) |
| C(1) | Tm(1) | C(9) | 30.6(4) |
| C(1) | Tm(1) | C(13) | 159.8(13) |
| C(2) | Tm(1) | C(3) | 28.5(3) |
| C(2) | Tm(1) | C(4) | 49.3(4) |
| C(2) | Tm(1) | C(5) | 64.1(5) |
| C(2) | Tm(1) | C(6) | 76.9(6) |
| C(2) | Tm(1) | C(7) | 85.1(7) |

| | | | |
|---|---|---|---|
| C(2) | Tm(1) | C(8) | 79.8(7) |
| C(2) | Tm(1) | C(9) | 59.3(7) |
| C(3) | Tm(1) | C(4) | 23.6(3) |
| C(3) | Tm(1) | C(5) | 43.8(3) |
| C(3) | Tm(1) | C(6) | 63.1(4) |
| C(3) | Tm(1) | C(7) | 80.0(5) |
| C(4) | Tm(1) | C(5) | 21.98(19) |
| C(4) | Tm(1) | C(6) | 43.4(3) |
| C(6) | Tm(1) | C(5) | 21.77(19) |
| C(7) | Tm(1) | C(4) | 63.8(3) |
| C(7) | Tm(1) | C(5) | 43.4(3) |
| C(7) | Tm(1) | C(6) | 22.2(3) |
| C(8) | Tm(1) | C(3) | 87.5(7) |
| C(8) | Tm(1) | C(4) | 79.6(5) |
| C(8) | Tm(1) | C(5) | 63.6(5) |
| C(8) | Tm(1) | C(6) | 45.8(5) |
| C(8) | Tm(1) | C(7) | 26.2(4) |
| C(9) | Tm(1) | C(3) | 78.5(8) |
| C(9) | Tm(1) | C(4) | 82.8(7) |
| C(9) | Tm(1) | C(5) | 76.3(7) |
| C(9) | Tm(1) | C(6) | 66.5(7) |
| C(9) | Tm(1) | C(7) | 53.0(6) |
| C(9) | Tm(1) | C(8) | 30.0(4) |
| C(10) | Tm(1) | C(1) | 108.9(7) |
| C(10) | Tm(1) | C(2) | 128.1(5) |
| C(10) | Tm(1) | C(3) | 152.0(4) |
| C(10) | Tm(1) | C(4) | 174.5(4) |
| C(10) | Tm(1) | C(5) | 163.4(4) |
| C(10) | Tm(1) | C(6) | 142.1(5) |
| C(10) | Tm(1) | C(7) | 121.6(5) |
| C(10) | Tm(1) | C(8) | 105.0(6) |
| C(10) | Tm(1) | C(9) | 99.8(8) |
| C(10) | Tm(1) | C(12) | 62.2(5) |
| C(10) | Tm(1) | C(13) | 84.5(7) |
| C(10) | Tm(1) | C(15) | 85.5(6) |
| C(11) | Tm(1) | C(1) | 122.4(7) |
| C(11) | Tm(1) | C(2) | 152.2(6) |
| C(11) | Tm(1) | C(3) | 174.6(7) |
| C(11) | Tm(1) | C(4) | 152.1(4) |
| C(11) | Tm(1) | C(5) | 131.2(4) |
| C(11) | Tm(1) | C(6) | 111.7(5) |
| C(11) | Tm(1) | C(7) | 94.7(6) |
| C(11) | Tm(1) | C(8) | 87.8(7) |
| C(11) | Tm(1) | C(9) | 98.7(8) |
| C(11) | Tm(1) | C(10) | 32.6(3) |
| C(11) | Tm(1) | C(12) | 32.6(4) |
| C(11) | Tm(1) | C(13) | 62.0(6) |
| C(11) | Tm(1) | C(14) | 85.0(9) |
| C(11) | Tm(1) | C(15) | 95.0(8) |
| C(11) | Tm(1) | C(16) | 85.7(6) |
| C(12) | Tm(1) | C(1) | 143.0(7) |
| C(12) | Tm(1) | C(2) | 166.2(11) |
| C(12) | Tm(1) | C(3) | 144.5(5) |
| C(12) | Tm(1) | C(4) | 121.4(5) |

| | | | |
|---|---|---|---|
| C(12) | Tm(1) | C(5) | 104.0(5) |
| C(12) | Tm(1) | C(6) | 89.7(7) |
| C(12) | Tm(1) | C(7) | 81.1(7) |
| C(12) | Tm(1) | C(8) | 88.6(8) |
| C(12) | Tm(1) | C(9) | 112.6(8) |
| C(12) | Tm(1) | C(13) | 31.8(4) |
| C(13) | Tm(1) | C(2) | 145.4(6) |
| C(13) | Tm(1) | C(3) | 116.9(6) |
| C(13) | Tm(1) | C(4) | 97.2(6) |
| C(13) | Tm(1) | C(5) | 87.0(7) |
| C(13) | Tm(1) | C(6) | 82.4(8) |
| C(13) | Tm(1) | C(7) | 86.0(8) |
| C(13) | Tm(1) | C(8) | 104.9(8) |
| C(13) | Tm(1) | C(9) | 134.5(8) |
| C(14) | Tm(1) | C(1) | 152.6(9) |
| C(14) | Tm(1) | C(2) | 121.7(8) |
| C(14) | Tm(1) | C(3) | 96.2(9) |
| C(14) | Tm(1) | C(4) | 85.0(9) |
| C(14) | Tm(1) | C(5) | 86.4(9) |
| C(14) | Tm(1) | C(6) | 93.8(9) |
| C(14) | Tm(1) | C(7) | 107.1(9) |
| C(14) | Tm(1) | C(8) | 131.6(9) |
| C(14) | Tm(1) | C(9) | 159.9(13) |
| C(14) | Tm(1) | C(10) | 93.9(10) |
| C(14) | Tm(1) | C(12) | 61.5(7) |
| C(14) | Tm(1) | C(13) | 32.3(4) |
| C(14) | Tm(1) | C(15) | 33.2(4) |
| C(15) | Tm(1) | C(1) | 131.4(8) |
| C(15) | Tm(1) | C(2) | 103.9(7) |
| C(15) | Tm(1) | C(3) | 88.7(7) |
| C(15) | Tm(1) | C(4) | 90.6(6) |
| C(15) | Tm(1) | C(5) | 103.2(6) |
| C(15) | Tm(1) | C(6) | 118.8(7) |
| C(15) | Tm(1) | C(7) | 137.4(8) |
| C(15) | Tm(1) | C(8) | 163.6(9) |
| C(15) | Tm(1) | C(9) | 161.9(9) |
| C(15) | Tm(1) | C(12) | 85.2(8) |
| C(15) | Tm(1) | C(13) | 62.9(6) |
| C(16) | Tm(1) | C(1) | 113.9(8) |
| C(16) | Tm(1) | C(2) | 99.1(7) |
| C(16) | Tm(1) | C(3) | 99.5(6) |
| C(16) | Tm(1) | C(4) | 112.5(5) |
| C(16) | Tm(1) | C(5) | 131.1(5) |
| C(16) | Tm(1) | C(6) | 150.5(6) |
| C(16) | Tm(1) | C(7) | 170.1(9) |
| C(16) | Tm(1) | C(8) | 163.3(9) |
| C(16) | Tm(1) | C(9) | 136.7(9) |
| C(16) | Tm(1) | C(10) | 62.4(5) |
| C(16) | Tm(1) | C(12) | 94.1(8) |
| C(16) | Tm(1) | C(13) | 85.5(8) |
| C(16) | Tm(1) | C(14) | 63.0(8) |
| C(16) | Tm(1) | C(15) | 32.9(4) |
| C(17) | Tm(1) | C(1) | 104.4(9) |
| C(17) | Tm(1) | C(2) | 107.4(7) |

| | | | |
|---|---|---|---|
| C(17) | Tm(1) | C(3) | 122.0(5) |
| C(17) | Tm(1) | C(4) | 141.7(4) |
| C(17) | Tm(1) | C(5) | 163.0(4) |
| C(17) | Tm(1) | C(6) | 174.9(6) |
| C(17) | Tm(1) | C(7) | 153.5(6) |
| C(17) | Tm(1) | C(8) | 131.4(7) |
| C(17) | Tm(1) | C(9) | 113.0(9) |
| C(17) | Tm(1) | C(10) | 32.9(4) |
| C(17) | Tm(1) | C(11) | 63.3(6) |
| C(17) | Tm(1) | C(12) | 85.9(8) |
| C(17) | Tm(1) | C(13) | 95.1(9) |
| C(17) | Tm(1) | C(14) | 86.3(10) |
| C(17) | Tm(1) | C(15) | 63.4(6) |
| C(17) | Tm(1) | C(16) | 32.9(4) |
| C(2) | C(1) | Tm(1) | 76.2(15) |
| C(9) | C(1) | Tm(1) | 77.2(19) |
| C(9) | C(1) | C(2) | 139(2) |
| C(1) | C(2) | Tm(1) | 72.6(15) |
| C(1) | C(2) | C(3) | 140.8(19) |
| C(3) | C(2) | Tm(1) | 88.7(14) |
| C(2) | C(3) | Tm(1) | 62.8(13) |
| C(2) | C(3) | C(4) | 140.6(18) |
| C(4) | C(3) | Tm(1) | 99.6(12) |
| C(3) | C(4) | Tm(1) | 56.8(11) |
| C(5) | C(4) | Tm(1) | 90.0(9) |
| C(5) | C(4) | C(3) | 135.7(15) |
| C(4) | C(5) | Tm(1) | 68.1(9) |
| C(6) | C(5) | Tm(1) | 73.1(10) |
| C(6) | C(5) | C(4) | 138.5(15) |
| C(5) | C(6) | Tm(1) | 85.1(10) |
| C(7) | C(6) | Tm(1) | 58.7(15) |
| C(7) | C(6) | C(5) | 139.6(18) |
| C(6) | C(7) | Tm(1) | 99.1(16) |
| C(8) | C(7) | Tm(1) | 61.0(18) |
| C(8) | C(7) | C(6) | 140(2) |
| C(7) | C(8) | Tm(1) | 93(2) |
| C(7) | C(8) | C(9) | 141(3) |
| C(9) | C(8) | Tm(1) | 71.5(19) |
| C(1) | C(9) | Tm(1) | 72.1(19) |
| C(1) | C(9) | C(8) | 136(2) |
| C(8) | C(9) | Tm(1) | 79(2) |
| C(11) | C(10) | Tm(1) | 73.1(11) |
| C(11) | C(10) | C(17) | 136.0(17) |
| C(17) | C(10) | Tm(1) | 72.9(10) |
| C(10) | C(11) | Tm(1) | 74.2(11) |
| C(10) | C(11) | C(12) | 134.8(17) |
| C(12) | C(11) | Tm(1) | 75.0(12) |
| C(11) | C(12) | Tm(1) | 72.4(11) |
| C(13) | C(12) | Tm(1) | 76.4(13) |
| C(13) | C(12) | C(11) | 137.0(17) |
| C(12) | C(13) | Tm(1) | 71.8(12) |
| C(12) | C(13) | C(14) | 131.4(19) |
| C(14) | C(13) | Tm(1) | 70.4(17) |
| C(13) | C(14) | Tm(1) | 77.2(17) |

| | | | |
|---|---|---|---|
| C(13) | C(14) | C(15) | 138(2) |
| C(15) | C(14) | Tm(1) | 74(2) |
| C(14) | C(15) | Tm(1) | 72(2) |
| C(16) | C(15) | Tm(1) | 72.6(19) |
| C(16) | C(15) | C(14) | 132(3) |
| C(15) | C(16) | Tm(1) | 74.5(19) |
| C(17) | C(16) | Tm(1) | 73.3(16) |
| C(17) | C(16) | C(15) | 137(3) |
| C(10) | C(17) | Tm(1) | 74.2(10) |
| C(16) | C(17) | Tm(1) | 73.8(17) |
| C(16) | C(17) | C(10) | 133(2) |

**Table S28.** Bond lengths (Å) and angles (deg) for [Tm(Cnt)(Cot)] **(5)** at 250 K. (Continuing over several pages).

| Atom A | Atom B | Distance(esd) |
|---|---|---|
| Tm(1) | C(1) | 2.46(3) |
| Tm(1) | C(2) | 2.56(3) |
| Tm(1) | C(3) | 2.76(4) |
| Tm(1) | C(4) | 3.13(3) |
| Tm(1) | C(5) | 3.531(19) |
| Tm(1) | C(6) | 3.641(17) |
| Tm(1) | C(7) | 3.382(16) |
| Tm(1) | C(8) | 2.89(2) |
| Tm(1) | C(9) | 2.55(3) |
| Tm(1) | C(10) | 2.44(2) |
| Tm(1) | C(11) | 2.40(3) |
| Tm(1) | C(12) | 2.45(5) |
| Tm(1) | C(13) | 2.55(4) |
| Tm(1) | C(14) | 2.62(2) |
| Tm(1) | C(15) | 2.516(19) |
| Tm(1) | C(16) | 2.46(2) |
| Tm(1) | C(17) | 2.482(18) |
| C(1) | C(2) | 1.352(14) |
| C(1) | C(9) | 1.363(13) |
| C(2) | C(3) | 1.361(13) |
| C(3) | C(4) | 1.358(13) |
| C(4) | C(5) | 1.341(13) |
| C(5) | C(6) | 1.365(12) |
| C(6) | C(7) | 1.369(12) |
| C(7) | C(8) | 1.382(12) |
| C(8) | C(9) | 1.386(13) |
| C(10) | C(11) | 1.390(14) |
| C(10) | C(17) | 1.395(14) |
| C(11) | C(12) | 1.402(14) |
| C(12) | C(13) | 1.406(14) |
| C(13) | C(14) | 1.400(13) |
| C(14) | C(15) | 1.382(13) |
| C(15) | C(16) | 1.390(13) |
| C(16) | C(17) | 1.388(13) |

| Atom A | Atom B | Atom C | Angle(esd) |
|---|---|---|---|
| C(1) | Tm(1) | C(2) | 31.1(4) |
| C(1) | Tm(1) | C(3) | 57.3(7) |
| C(1) | Tm(1) | C(4) | 73.7(7) |
| C(1) | Tm(1) | C(5) | 78.2(7) |
| C(1) | Tm(1) | C(6) | 76.5(6) |
| C(1) | Tm(1) | C(7) | 70.9(6) |
| C(1) | Tm(1) | C(8) | 57.3(5) |
| C(1) | Tm(1) | C(9) | 31.5(4) |
| C(1) | Tm(1) | C(13) | 153.9(7) |
| C(1) | Tm(1) | C(14) | 159.9(11) |
| C(1) | Tm(1) | C(15) | 142.8(8) |
| C(1) | Tm(1) | C(17) | 108.9(7) |
| C(2) | Tm(1) | C(3) | 29.4(4) |
| C(2) | Tm(1) | C(4) | 52.4(6) |

| | | | |
|---|---|---|---|
| C(2) | Tm(1) | C(5) | 66.7(7) |
| C(2) | Tm(1) | C(6) | 76.7(6) |
| C(2) | Tm(1) | C(7) | 83.0(6) |
| C(2) | Tm(1) | C(8) | 79.8(7) |
| C(2) | Tm(1) | C(14) | 133.6(9) |
| C(3) | Tm(1) | C(4) | 25.7(4) |
| C(3) | Tm(1) | C(5) | 45.5(5) |
| C(3) | Tm(1) | C(6) | 63.2(5) |
| C(3) | Tm(1) | C(7) | 78.9(6) |
| C(3) | Tm(1) | C(8) | 87.7(8) |
| C(4) | Tm(1) | C(5) | 22.2(3) |
| C(4) | Tm(1) | C(6) | 43.3(3) |
| C(4) | Tm(1) | C(7) | 63.6(4) |
| C(5) | Tm(1) | C(6) | 21.9(2) |
| C(7) | Tm(1) | C(5) | 43.5(3) |
| C(7) | Tm(1) | C(6) | 22.1(2) |
| C(8) | Tm(1) | C(4) | 80.4(5) |
| C(8) | Tm(1) | C(5) | 63.9(4) |
| C(8) | Tm(1) | C(6) | 44.5(3) |
| C(8) | Tm(1) | C(7) | 23.8(3) |
| C(9) | Tm(1) | C(2) | 60.2(6) |
| C(9) | Tm(1) | C(3) | 79.5(8) |
| C(9) | Tm(1) | C(4) | 84.9(7) |
| C(9) | Tm(1) | C(5) | 77.4(7) |
| C(9) | Tm(1) | C(6) | 64.4(6) |
| C(9) | Tm(1) | C(7) | 49.1(5) |
| C(9) | Tm(1) | C(8) | 28.7(3) |
| C(9) | Tm(1) | C(14) | 146.0(6) |
| C(10) | Tm(1) | C(1) | 105.3(8) |
| C(10) | Tm(1) | C(2) | 113.9(9) |
| C(10) | Tm(1) | C(3) | 132.5(9) |
| C(10) | Tm(1) | C(4) | 153.9(7) |
| C(10) | Tm(1) | C(5) | 174.3(7) |
| C(10) | Tm(1) | C(6) | 162.8(5) |
| C(10) | Tm(1) | C(7) | 141.7(5) |
| C(10) | Tm(1) | C(8) | 121.7(6) |
| C(10) | Tm(1) | C(9) | 108.0(8) |
| C(10) | Tm(1) | C(12) | 64.2(7) |
| C(10) | Tm(1) | C(13) | 85.9(9) |
| C(10) | Tm(1) | C(14) | 93.8(8) |
| C(10) | Tm(1) | C(15) | 85.3(7) |
| C(10) | Tm(1) | C(16) | 63.0(5) |
| C(10) | Tm(1) | C(17) | 32.9(4) |
| C(11) | Tm(1) | C(1) | 114.8(9) |
| C(11) | Tm(1) | C(2) | 138.1(10) |
| C(11) | Tm(1) | C(3) | 164.8(11) |
| C(11) | Tm(1) | C(4) | 169.2(10) |
| C(11) | Tm(1) | C(5) | 149.3(8) |
| C(11) | Tm(1) | C(6) | 130.1(6) |
| C(11) | Tm(1) | C(7) | 111.8(6) |
| C(11) | Tm(1) | C(8) | 98.5(7) |
| C(11) | Tm(1) | C(9) | 99.3(8) |
| C(11) | Tm(1) | C(10) | 33.3(4) |
| C(11) | Tm(1) | C(12) | 33.6(5) |

| | | | |
|---|---|---|---|
| C(11) | Tm(1) | C(13) | 62.9(8) |
| C(11) | Tm(1) | C(14) | 84.6(8) |
| C(11) | Tm(1) | C(15) | 93.8(8) |
| C(11) | Tm(1) | C(16) | 85.9(7) |
| C(11) | Tm(1) | C(17) | 63.1(6) |
| C(12) | Tm(1) | C(1) | 132.8(9) |
| C(12) | Tm(1) | C(2) | 163.9(10) |
| C(12) | Tm(1) | C(3) | 161.4(11) |
| C(12) | Tm(1) | C(4) | 135.8(10) |
| C(12) | Tm(1) | C(5) | 116.9(9) |
| C(12) | Tm(1) | C(6) | 101.7(8) |
| C(12) | Tm(1) | C(7) | 89.9(8) |
| C(12) | Tm(1) | C(8) | 88.0(9) |
| C(12) | Tm(1) | C(9) | 104.4(9) |
| C(12) | Tm(1) | C(13) | 32.6(4) |
| C(12) | Tm(1) | C(14) | 61.6(7) |
| C(12) | Tm(1) | C(15) | 84.2(8) |
| C(12) | Tm(1) | C(16) | 94.8(9) |
| C(12) | Tm(1) | C(17) | 86.3(8) |
| C(13) | Tm(1) | C(2) | 158.9(12) |
| C(13) | Tm(1) | C(3) | 130.5(10) |
| C(13) | Tm(1) | C(4) | 106.5(9) |
| C(13) | Tm(1) | C(5) | 92.9(8) |
| C(13) | Tm(1) | C(6) | 86.1(7) |
| C(13) | Tm(1) | C(7) | 85.6(7) |
| C(13) | Tm(1) | C(8) | 96.6(7) |
| C(13) | Tm(1) | C(9) | 122.8(7) |
| C(13) | Tm(1) | C(14) | 31.4(4) |
| C(14) | Tm(1) | C(3) | 104.9(8) |
| C(14) | Tm(1) | C(4) | 86.4(8) |
| C(14) | Tm(1) | C(5) | 82.3(7) |
| C(14) | Tm(1) | C(6) | 87.2(6) |
| C(14) | Tm(1) | C(7) | 97.9(5) |
| C(14) | Tm(1) | C(8) | 117.4(6) |
| C(15) | Tm(1) | C(2) | 111.8(7) |
| C(15) | Tm(1) | C(3) | 89.0(8) |
| C(15) | Tm(1) | C(4) | 81.5(7) |
| C(15) | Tm(1) | C(5) | 89.3(6) |
| C(15) | Tm(1) | C(6) | 103.8(5) |
| C(15) | Tm(1) | C(7) | 121.5(5) |
| C(15) | Tm(1) | C(8) | 144.7(5) |
| C(15) | Tm(1) | C(9) | 166.3(10) |
| C(15) | Tm(1) | C(13) | 60.2(6) |
| C(15) | Tm(1) | C(14) | 31.1(3) |
| C(16) | Tm(1) | C(1) | 122.4(7) |
| C(16) | Tm(1) | C(2) | 98.2(7) |
| C(16) | Tm(1) | C(3) | 88.6(8) |
| C(16) | Tm(1) | C(4) | 95.0(6) |
| C(16) | Tm(1) | C(5) | 111.4(5) |
| C(16) | Tm(1) | C(6) | 131.0(4) |
| C(16) | Tm(1) | C(7) | 152.0(4) |
| C(16) | Tm(1) | C(8) | 175.3(6) |
| C(16) | Tm(1) | C(9) | 152.5(7) |
| C(16) | Tm(1) | C(13) | 83.7(7) |

| | | | |
|---|---|---|---|
| C(16) | Tm(1) | C(14) | 61.0(5) |
| C(16) | Tm(1) | C(15) | 32.4(3) |
| C(16) | Tm(1) | C(17) | 32.6(3) |
| C(17) | Tm(1) | C(2) | 99.6(7) |
| C(17) | Tm(1) | C(3) | 105.7(7) |
| C(17) | Tm(1) | C(4) | 121.8(5) |
| C(17) | Tm(1) | C(5) | 141.9(5) |
| C(17) | Tm(1) | C(6) | 163.2(4) |
| C(17) | Tm(1) | C(7) | 174.6(4) |
| C(17) | Tm(1) | C(8) | 151.7(4) |
| C(17) | Tm(1) | C(9) | 128.3(6) |
| C(17) | Tm(1) | C(13) | 93.3(8) |
| C(17) | Tm(1) | C(14) | 83.7(6) |
| C(17) | Tm(1) | C(15) | 62.0(5) |
| C(2) | C(1) | Tm(1) | 78.4(16) |
| C(2) | C(1) | C(9) | 141(2) |
| C(9) | C(1) | Tm(1) | 77.6(15) |
| C(1) | C(2) | Tm(1) | 70.5(15) |
| C(1) | C(2) | C(3) | 136(2) |
| C(3) | C(2) | Tm(1) | 83(2) |
| C(2) | C(3) | Tm(1) | 67.3(18) |
| C(4) | C(3) | Tm(1) | 93(2) |
| C(4) | C(3) | C(2) | 141(2) |
| C(3) | C(4) | Tm(1) | 62(2) |
| C(5) | C(4) | Tm(1) | 96.0(15) |
| C(5) | C(4) | C(3) | 139.9(19) |
| C(4) | C(5) | Tm(1) | 61.8(14) |
| C(4) | C(5) | C(6) | 139.9(16) |
| C(6) | C(5) | Tm(1) | 83.6(10) |
| C(5) | C(6) | Tm(1) | 74.5(9) |
| C(5) | C(6) | C(7) | 139.8(14) |
| C(7) | C(6) | Tm(1) | 68.3(9) |
| C(6) | C(7) | Tm(1) | 89.7(9) |
| C(6) | C(7) | C(8) | 137.9(16) |
| C(8) | C(7) | Tm(1) | 57.6(11) |
| C(7) | C(8) | Tm(1) | 98.6(12) |
| C(7) | C(8) | C(9) | 137(2) |
| C(9) | C(8) | Tm(1) | 61.9(13) |
| C(1) | C(9) | Tm(1) | 70.9(15) |
| C(1) | C(9) | C(8) | 141(2) |
| C(8) | C(9) | Tm(1) | 89.4(14) |
| C(11) | C(10) | Tm(1) | 71.9(15) |
| C(11) | C(10) | C(17) | 133.3(19) |
| C(17) | C(10) | Tm(1) | 75.2(10) |
| C(10) | C(11) | Tm(1) | 74.8(15) |
| C(10) | C(11) | C(12) | 137(2) |
| C(12) | C(11) | Tm(1) | 75(2) |
| C(11) | C(12) | Tm(1) | 71(2) |
| C(11) | C(12) | C(13) | 134(3) |
| C(13) | C(12) | Tm(1) | 77(2) |
| C(12) | C(13) | Tm(1) | 70(2) |
| C(14) | C(13) | Tm(1) | 77.3(15) |
| C(14) | C(13) | C(12) | 136(3) |
| C(13) | C(14) | Tm(1) | 71.3(15) |

| | | | |
|---|---|---|---|
| C(15) | C(14) | Tm(1) | 70.2(11) |
| C(15) | C(14) | C(13) | 132(2) |
| C(14) | C(15) | Tm(1) | 78.6(11) |
| C(14) | C(15) | C(16) | 137.7(18) |
| C(16) | C(15) | Tm(1) | 71.7(11) |
| C(15) | C(16) | Tm(1) | 75.9(11) |
| C(17) | C(16) | Tm(1) | 74.4(11) |
| C(17) | C(16) | C(15) | 135.7(17) |
| C(10) | C(17) | Tm(1) | 71.9(11) |
| C(16) | C(17) | Tm(1) | 73.0(11) |
| C(16) | C(17) | C(10) | 134.1(17) |

**Table S29.** Bond lengths (Å) and angles (deg) for **2b**. (Continuing over several pages).

| Atom | Atom | Length/Å |
|---|---|---|
| Dy(1) | N(1) | 2.492(5) |
| Dy(1) | C(9) | 2.712(5) |
| Dy(1) | C(8) | 2.563(7) |
| Dy(1) | C(3) | 2.550(7) |
| Dy(1) | C(6) | 2.573(7) |
| Dy(1) | C(17) | 2.695(6) |
| Dy(1) | C(4) | 2.581(5) |
| Dy(1) | C(2) | 2.547(7) |
| Dy(1) | C(5) | 2.596(5) |
| Dy(1) | C(16) | 2.775(6) |
| Dy(1) | C(7) | 2.568(7) |
| Dy(1) | C(1) | 2.558(6) |
| N(1) | C(18) | 1.137(7) |
| C(15) | C(14) | 1.395(9) |
| C(15) | C(16) | 1.407(9) |
| C(9) | C(17) | 1.407(9) |
| C(9) | C(10) | 1.400(9) |
| C(8) | C(7) | 1.389(12) |
| C(8) | C(1) | 1.416(11) |
| C(12) | C(13) | 1.401(10) |
| C(12) | C(11) | 1.376(9) |
| C(3) | C(4) | 1.385(10) |
| C(3) | C(2) | 1.408(11) |
| C(13) | C(14) | 1.373(11) |
| C(6) | C(5) | 1.398(10) |
| C(6) | C(7) | 1.433(10) |
| C(17) | C(16) | 1.410(10) |
| C(4) | C(5) | 1.407(9) |
| C(10) | C(11) | 1.405(9) |
| C(19) | C(18) | 1.454(8) |
| C(2) | C(1) | 1.403(11) |
| Dy(2) | Dy(2)1 | 1.0868(14) |
| Dy(2) | C(22) | 2.55(2) |
| Dy(2) | C(23) | 2.60(2) |
| Dy(2) | C(24) | 2.537(19) |
| Dy(2) | C(26) | 2.52(2) |
| Dy(2) | C(27) | 2.50(3) |
| Dy(2) | C(21) | 2.47(5) |
| Dy(2) | C(28) | 2.60(4) |
| Dy(2) | C(25) | 2.485(19) |
| Dy(2) | C(31) | 2.68(2) |
| Dy(2) | C(30) | 2.670(19) |
| Dy(2) | C(32) | 2.63(5) |
| C(22) | C(23) | 1.40(2) |
| C(22) | C(21) | 1.45(3) |
| C(23) | C(24) | 1.42(3) |
| C(24) | C(25) | 1.40(3) |
| C(26) | C(27) | 1.39(4) |
| C(26) | C(25) | 1.44(3) |
| C(27) | C(28) | 1.48(4) |
| C(21) | C(28) | 1.43(4) |

| | | |
|---|---|---|
| Cl(1) | C(20) | 1.759(5) |
| Cl(2) | C(20) | 1.761(6) |
| C(36) | C(35) | 1.45(4) |
| C(36) | C(37) | 1.40(3) |
| C(31) | C(30) | 1.36(4) |
| C(31) | C(32) | 1.30(5) |
| C(30) | C(29) | 1.44(3) |
| C(35) | C(34) | 1.34(3) |
| C(34) | C(33) | 1.36(4) |
| C(33) | C(32) | 1.35(5) |
| C(29) | C(37) | 1.43(3) |

| Atom | Atom | Atom | Angle/° |
|---|---|---|---|
| N(1) | Dy(1) | C(9) | 71.36(17) |
| N(1) | Dy(1) | C(8) | 75.23(19) |
| N(1) | Dy(1) | C(3) | 127.1(2) |
| N(1) | Dy(1) | C(6) | 123.3(2) |
| N(1) | Dy(1) | C(17) | 70.03(18) |
| N(1) | Dy(1) | C(4) | 157.1(2) |
| N(1) | Dy(1) | C(2) | 96.4(2) |
| N(1) | Dy(1) | C(5) | 154.0(2) |
| N(1) | Dy(1) | C(16) | 89.35(19) |
| N(1) | Dy(1) | C(7) | 93.5(2) |
| N(1) | Dy(1) | C(1) | 75.94(17) |
| C(9) | Dy(1) | C(16) | 57.0(2) |
| C(8) | Dy(1) | C(9) | 146.5(2) |
| C(8) | Dy(1) | C(6) | 61.0(3) |
| C(8) | Dy(1) | C(17) | 134.7(2) |
| C(8) | Dy(1) | C(4) | 90.3(2) |
| C(8) | Dy(1) | C(5) | 82.0(2) |
| C(8) | Dy(1) | C(16) | 125.6(3) |
| C(8) | Dy(1) | C(7) | 31.4(3) |
| C(3) | Dy(1) | C(9) | 116.4(2) |
| C(3) | Dy(1) | C(8) | 82.3(3) |
| C(3) | Dy(1) | C(6) | 82.45(19) |
| C(3) | Dy(1) | C(17) | 142.4(2) |
| C(3) | Dy(1) | C(4) | 31.3(2) |
| C(3) | Dy(1) | C(5) | 60.2(2) |
| C(3) | Dy(1) | C(16) | 141.0(2) |
| C(3) | Dy(1) | C(7) | 91.0(2) |
| C(3) | Dy(1) | C(1) | 60.8(2) |
| C(6) | Dy(1) | C(9) | 144.0(2) |
| C(6) | Dy(1) | C(17) | 118.1(2) |
| C(6) | Dy(1) | C(4) | 60.3(2) |
| C(6) | Dy(1) | C(5) | 31.4(2) |
| C(6) | Dy(1) | C(16) | 88.6(2) |
| C(17) | Dy(1) | C(9) | 30.16(18) |
| C(17) | Dy(1) | C(16) | 29.8(2) |
| C(4) | Dy(1) | C(9) | 120.42(19) |
| C(4) | Dy(1) | C(17) | 130.66(19) |
| C(4) | Dy(1) | C(5) | 31.5(2) |
| C(4) | Dy(1) | C(16) | 113.6(2) |
| C(2) | Dy(1) | C(9) | 120.2(2) |
| C(2) | Dy(1) | C(8) | 61.1(3) |

| | | | |
|---|---|---|---|
| C(2) | Dy(1) | C(3) | 32.1(2) |
| C(2) | Dy(1) | C(6) | 92.4(2) |
| C(2) | Dy(1) | C(17) | 149.3(2) |
| C(2) | Dy(1) | C(4) | 60.8(2) |
| C(2) | Dy(1) | C(5) | 83.0(2) |
| C(2) | Dy(1) | C(16) | 172.4(2) |
| C(2) | Dy(1) | C(7) | 83.4(2) |
| C(2) | Dy(1) | C(1) | 31.9(3) |
| C(5) | Dy(1) | C(9) | 131.09(19) |
| C(5) | Dy(1) | C(17) | 121.3(2) |
| C(5) | Dy(1) | C(16) | 94.0(2) |
| C(7) | Dy(1) | C(9) | 152.5(2) |
| C(7) | Dy(1) | C(6) | 32.4(2) |
| C(7) | Dy(1) | C(17) | 123.6(2) |
| C(7) | Dy(1) | C(4) | 82.3(2) |
| C(7) | Dy(1) | C(5) | 60.6(2) |
| C(7) | Dy(1) | C(16) | 101.4(2) |
| C(1) | Dy(1) | C(9) | 132.1(2) |
| C(1) | Dy(1) | C(8) | 32.1(2) |
| C(1) | Dy(1) | C(6) | 83.6(2) |
| C(1) | Dy(1) | C(17) | 145.78(19) |
| C(1) | Dy(1) | C(4) | 82.38(19) |
| C(1) | Dy(1) | C(5) | 91.28(19) |
| C(1) | Dy(1) | C(16) | 155.6(2) |
| C(1) | Dy(1) | C(7) | 61.0(3) |
| C(18) | N(1) | Dy(1) | 176.1(5) |
| C(14) | C(15) | C(16) | 137.0(7) |
| C(17) | C(9) | Dy(1) | 74.2(4) |
| C(10) | C(9) | Dy(1) | 78.6(3) |
| C(10) | C(9) | C(17) | 135.4(6) |
| C(7) | C(8) | Dy(1) | 74.5(4) |
| C(7) | C(8) | C(1) | 136.3(7) |
| C(1) | C(8) | Dy(1) | 73.8(4) |
| C(11) | C(12) | C(13) | 138.8(6) |
| C(4) | C(3) | Dy(1) | 75.6(4) |
| C(4) | C(3) | C(2) | 136.7(7) |
| C(2) | C(3) | Dy(1) | 73.8(4) |
| C(14) | C(13) | C(12) | 136.3(5) |
| C(13) | C(14) | C(15) | 138.3(6) |
| C(5) | C(6) | Dy(1) | 75.2(4) |
| C(5) | C(6) | C(7) | 134.1(7) |
| C(7) | C(6) | Dy(1) | 73.6(4) |
| C(9) | C(17) | Dy(1) | 75.6(4) |
| C(9) | C(17) | C(16) | 136.9(6) |
| C(16) | C(17) | Dy(1) | 78.2(3) |
| C(3) | C(4) | Dy(1) | 73.1(3) |
| C(3) | C(4) | C(5) | 135.3(6) |
| C(5) | C(4) | Dy(1) | 74.8(3) |
| C(9) | C(10) | Dy(1) | 72.0(3) |
| C(9) | C(10) | C(11) | 138.3(7) |
| C(11) | C(10) | Dy(1) | 99.8(4) |
| C(3) | C(2) | Dy(1) | 74.1(4) |
| C(1) | C(2) | Dy(1) | 74.5(4) |
| C(1) | C(2) | C(3) | 133.6(7) |

| | | | |
|---|---|---|---|
| C(6) | C(5) | Dy(1) | 73.4(4) |
| C(6) | C(5) | C(4) | 134.8(7) |
| C(4) | C(5) | Dy(1) | 73.6(3) |
| N(1) | C(18) | C(19) | 177.6(8) |
| C(15) | C(16) | Dy(1) | 100.7(4) |
| C(15) | C(16) | C(17) | 138.6(6) |
| C(17) | C(16) | Dy(1) | 71.9(3) |
| C(8) | C(7) | Dy(1) | 74.1(4) |
| C(8) | C(7) | C(6) | 134.7(7) |
| C(6) | C(7) | Dy(1) | 74.0(4) |
| C(12) | C(11) | C(10) | 138.0(7) |
| C(8) | C(1) | Dy(1) | 74.1(4) |
| C(2) | C(1) | Dy(1) | 73.6(4) |
| C(2) | C(1) | C(8) | 134.3(7) |
| Dy(2)1 | Dy(2) | C(22) | 146.1(6) |
| Dy(2)1 | Dy(2) | C(23) | 157.6(5) |
| Dy(2)1 | Dy(2) | C(24) | 130.2(6) |
| Dy(2)1 | Dy(2) | C(26) | 72.8(6) |
| Dy(2)1 | Dy(2) | C(27) | 66.0(7) |
| Dy(2)1 | Dy(2) | C(21) | 113.0(8) |
| Dy(2)1 | Dy(2) | C(28) | 84.1(9) |
| Dy(2)1 | Dy(2) | C(25) | 98.2(5) |
| Dy(2)1 | Dy(2) | C(31) | 89.2(8) |
| Dy(2)1 | Dy(2) | C(30) | 87.9(5) |
| Dy(2)1 | Dy(2) | C(32) | 78.8(10) |
| C(22) | Dy(2) | C(23) | 31.5(6) |
| C(22) | Dy(2) | C(28) | 62.3(10) |
| C(23) | Dy(2) | C(28) | 84.2(10) |
| C(24) | Dy(2) | C(22) | 61.1(7) |
| C(24) | Dy(2) | C(23) | 32.2(6) |
| C(24) | Dy(2) | C(28) | 93.6(10) |
| C(26) | Dy(2) | C(22) | 93.9(7) |
| C(26) | Dy(2) | C(23) | 84.8(8) |
| C(26) | Dy(2) | C(24) | 62.4(8) |
| C(26) | Dy(2) | C(28) | 62.7(9) |
| C(27) | Dy(2) | C(22) | 85.9(9) |
| C(27) | Dy(2) | C(23) | 94.1(9) |
| C(27) | Dy(2) | C(24) | 85.2(10) |
| C(27) | Dy(2) | C(26) | 32.2(9) |
| C(27) | Dy(2) | C(28) | 33.6(10) |
| C(21) | Dy(2) | C(22) | 33.6(8) |
| C(21) | Dy(2) | C(23) | 62.7(10) |
| C(21) | Dy(2) | C(24) | 85.9(11) |
| C(21) | Dy(2) | C(26) | 86.5(10) |
| C(21) | Dy(2) | C(27) | 63.9(10) |
| C(21) | Dy(2) | C(28) | 32.5(9) |
| C(21) | Dy(2) | C(25) | 96.1(11) |
| C(25) | Dy(2) | C(22) | 84.6(6) |
| C(25) | Dy(2) | C(23) | 61.9(7) |
| C(25) | Dy(2) | C(24) | 32.2(6) |
| C(25) | Dy(2) | C(26) | 33.4(8) |
| C(25) | Dy(2) | C(27) | 63.1(10) |
| C(25) | Dy(2) | C(28) | 86.0(10) |
| C(30) | Dy(2) | C(31) | 29.4(9) |

| | | | |
|---|---|---|---|
| C(32) | Dy(2) | C(31) | 28.4(12) |
| C(32) | Dy(2) | C(30) | 56.3(13) |
| C(23) | C(22) | Dy(2) | 76.3(14) |
| C(23) | C(22) | C(21) | 136(2) |
| C(21) | C(22) | Dy(2) | 70(2) |
| C(22) | C(23) | Dy(2) | 72.3(13) |
| C(22) | C(23) | C(24) | 133(2) |
| C(24) | C(23) | Dy(2) | 71.5(12) |
| C(23) | C(24) | Dy(2) | 76.4(12) |
| C(25) | C(24) | Dy(2) | 71.8(11) |
| C(25) | C(24) | C(23) | 136(2) |
| C(27) | C(26) | Dy(2) | 72.8(15) |
| C(27) | C(26) | C(25) | 134(2) |
| C(25) | C(26) | Dy(2) | 71.8(11) |
| C(26) | C(27) | Dy(2) | 74.9(14) |
| C(26) | C(27) | C(28) | 137(3) |
| C(28) | C(27) | Dy(2) | 77.2(19) |
| C(22) | C(21) | Dy(2) | 76.2(19) |
| C(28) | C(21) | Dy(2) | 79(2) |
| C(28) | C(21) | C(22) | 136(3) |
| C(27) | C(28) | Dy(2) | 69.3(16) |
| C(21) | C(28) | Dy(2) | 69(2) |
| C(21) | C(28) | C(27) | 130(3) |
| C(24) | C(25) | Dy(2) | 75.9(11) |
| C(24) | C(25) | C(26) | 136(2) |
| C(26) | C(25) | Dy(2) | 74.8(13) |
| Cl(1) | C(20) | Cl(2) | 111.6(3) |
| C(37) | C(36) | C(35) | 136(2) |
| C(30) | C(31) | Dy(2) | 74.9(13) |
| C(32) | C(31) | Dy(2) | 74(2) |
| C(32) | C(31) | C(30) | 140(3) |
| C(31) | C(30) | Dy(2) | 75.7(12) |
| C(31) | C(30) | C(29) | 140(2) |
| C(29) | C(30) | Dy(2) | 77.1(11) |
| C(34) | C(35) | C(36) | 139(3) |
| C(35) | C(34) | C(33) | 136(3) |
| C(34) | C(33) | Dy(2) | 95(2) |
| C(32) | C(33) | Dy(2) | 62(3) |
| C(32) | C(33) | C(34) | 144(4) |
| C(30) | C(29) | Dy(2) | 72.1(11) |
| C(37) | C(29) | Dy(2) | 89.5(11) |
| C(37) | C(29) | C(30) | 132(2) |
| C(36) | C(37) | C(29) | 141(2) |
| C(31) | C(32) | Dy(2) | 78(2) |
| C(31) | C(32) | C(33) | 137(5) |
| C(33) | C(32) | Dy(2) | 91(3) |

## 6. Theoretical calculations

All wave function-based calculations were performed using the State-Averaged Complete Active Space Self-Consistent Field approach with Restricted-Active-Space-State-Interaction method (SA-CASSCF/RASSI-SO), as implemented in the *OpenMolcas* quantum-chemistry package. [16] In this approach, the relativistic effects are treated in two steps on the basis of the Douglas–Kroll Hamiltonian.[17] The scalar terms are included in the basis-set generation and are used to determine the CASSCF wave functions and energies.[18] Spin-orbit coupling is then added within the RASSI-SO method, which mixes the calculated CASSCF wave functions.[19-20] Spin−orbit (SO) integrals are calculated using the AMFI (atomic mean-field integrals) approximation.[21] The resulting spin-orbit wave functions and energies are used to compute the magnetic properties and g-tensors of the ground state multiplet following the pseudospin $\boldsymbol{S}$= 1/2 formalism, as implemented in the SINGLE_ANISO routine.[22] Cholesky decomposition of the bielectronic integrals was employed to save disk space and to speed up the calculations.[23]

The active space considered in the calculations consisted of the 4f electrons of the Ln(III) ions (eight for Tb(III), nine for Dy(III), ten for Ho(III), eleven for Er(III) and twelve for Tm(III)) spanning the seven 4f orbitals; that is CAS(8,7)SCF for **1 (COT)Tb(CNT)**, CAS(9,7)SCF for **2 (COT)Dy(CNT)**, CAS(10,7)SCF for **3 (COT)Ho(CNT)**, CAS(11,7)SCF for **4 (COT)Er(CNT)** and CAS(12,7)SCF for **5 (COT)Tm(CNT)**. State-averaged CASSCF calculations were performed for all of the septets (7 roots), all of the quintets (140 roots), 91 out of the 588 triplets and 77 out of the 490 singlets of the Tb(III) ion ; for all of the sextets (21 roots), all of the quartets (224 roots) and 224 out the 490 doublets of the Dy(III) ion ; for all of the quintets (35 roots), all of the triplets (210 roots) and all of the singlets (196) of the Ho(III) ion ; for all of the quartets (35 roots) and all of the doublets (112 roots) of the Er(III) ion and for all of the triplets (21 roots)and all of the singlets (28 roots) of the Tm(III) ion. In RASSI-SO, 7 septets, 140 quartets, 91 triplets and 77 singlets were mixed through spin–orbit coupling for the Tb(III) ion, 21 sextets, 224 quartets and 224 doublets were mixed through spin–orbit coupling for the Dy(III) ion, 35 quintets, 210 triplets and 196 doublets were mixed through spin–orbit coupling for the Ho(III) ion, 35 quartets and 112 doublets were mixed through spin–orbit coupling for the Er(III) ion and 21 triplets and 28 singlets were mixed through spin–orbit coupling for the Tm(III) ion. The Ln(III) ions and the carbon atoms were described with ANO-RCC-VTZP basis sets and the hydrogen atoms were described with ANO-RCC-VDZ basis sets.[24,25]

**Table S30.** Computed energy levels (the ground state is set at zero) and the main components (>10%) of the wavefunction for each $m_J$ state of the ground-state multiplet $^7F_6$ for all structures of **1**.

| | 150K | | 300K | |
|---|---|---|---|---|
| **State** | **Energy (cm$^{-1}$)** | **Wavefunction** | **Energy (cm$^{-1}$)** | **Wavefunction** |
| 1 | 0 | 98.1% \|0> | 0 | 99.8% \|0> |
| 2 | 27 | 99.9% \|±1> | 36 | 100% \|±1> |
| 3 | 61 | 99.5% \|±1> | 47 | 99.9% \|±1> |
| 4 | 167 | 99.9% \|±2> | 168 | 100% \|±2> |
| 5 | 179 | 98.0% \|±2> | 168 | 99.8% \|±2> |
| 6 | 386 | 99.8% \|±3> | 380 | 100% \|±3> |
| 7 | 387 | 99.5% \|±3> | 380 | 100% \|±3> |
| 8 | 669 | 99.3% \|±4> | 660 | 99.9% \|±4> |
| 9 | 669 | 99.3% \|±4> | 660 | 99.9% \|±4> |
| 10 | 726 | 98.7% \|±6> | 719 | 99.8% \|±6> |
| 11 | 726 | 98.7% \|±6> | 719 | 99.8% \|±6> |
| 12 | 873 | 99.1% \|±5> | 861 | 99.8% \|±5> |
| 13 | 873 | 99.1% \|±5> | 861 | 99.8% \|±5> |

**Table S31.** Computed energy levels (the ground state is set at zero), composition of the *g*-tensor ($g_x$, $g_y$, $g_z$) and the main components (>10%) of the wavefunction for each $m_J$ state of the ground-state multiplet $^6H_{15/2}$ for all structures of **2.**

| | **150K** | | | **300K** | | |
|---|---|---|---|---|---|---|
| **KD** | **Energy ($cm^{-1}$)** | **g** | **Wavefunction** | **Energy ($cm^{-1}$)** | **g** | **Wavefunction** |
| 1 | 0 | 0.1<br>0.2<br>17.0 | 64.8% \|±15/2> +<br>12.6% \|±11/2> | 0 | 0.2<br>0.1<br>18.7 | 83.1% \|±15/2> +<br>12.5% \|±11/2> |
| 2 | 24 | 1.5<br>2.6<br>12.1 | 21.8% \|±3/2> +<br>21.2% \|±1/2> +<br>17.8% \|±13/2> +<br>14.3% \|±5/2> +<br>10.1% \|±9/2> | 34 | 3.9<br>1.6<br>12.4 | 48.4% \|±13/2> +<br>24.6% \|±9/2> |
| 3 | 42 | 3.2<br>5.9<br>8.9 | 22.0% \|±11/2> +<br>18.6% \|±13/2> +<br>15.2% \|±5/2> +<br>15.1% \|±7/2> +<br>14.1% \|±9/2> | 54 | 6.2<br>6.6<br>3.8 | 25.2% \|±7/2> +<br>17.7% \|±3/2> +<br>14.9% \|±11/2> +<br>12.5% \|±1/2> +<br>11.8% \|±5/2> |
| 4 | 63 | 1.1<br>3.3<br>10.3 | 21.0% \|±3/2> +<br>19.9% \|±9/2> +<br>14.2% \|±5/2> +<br>13.5% \|±11/2> +<br>12.5% \|±13/2> | 81 | 0.6<br>0.1<br>10.7 | 20.4% \|±11/2> +<br>15.5% \|±9/2> +<br>14.9% \|±3/2> +<br>14.2% \|±5/2> +<br>12.7% \|±13/2> +<br>11.0% \|±1/2> |
| 5 | 81 | 0.8<br>1.0<br>14.8 | 31.4% \|±1/2> +<br>16.8% \|±9/2> +<br>15.3% \|±7/2> +<br>14.7% \|±3/2> | 118 | 0.5<br>0.3<br>13.6 | 22.6% \|±1/2> +<br>21.2% \|±7/2> +<br>17.4% \|±9/2> +<br>13.4% \|±11/2> +<br>10.1% \|±5/2> |
| 6 | 109 | 0.1<br>0.2<br>19.3 | 22.6% \|±5/2> +<br>20.7% \|±1/2> +<br>20.3% \|±7/2> +<br>19.7% \|±3/2> +<br>11.8% \|±9/2> | 149 | 0.1<br>0.1<br>17.7 | 26.7% \|±5/2> +<br>24.7% \|±3/2> +<br>16.9% \|±7/2> +<br>13.4% \|±1/2> |
| 7 | 261 | 0.0<br>0.0<br>17.4 | 20.9% \|±13/2> +<br>19.8% \|±5/2> +<br>16.2% \|±3/2> +<br>12.8% \|±15/2> +<br>11.7% \|±7/2> +<br>10.3% \|±1/2> | 327 | 0.0<br>0.0<br>17.5 | 28.8% \|±1/2> +<br>21.7% \|±3/2> +<br>16.7% \|±11/2> +<br>12.6% \|±13/2> |
| 8 | 910 | 0.0<br>0.0<br>19.9 | 26.3% \|±9/2> +<br>26.1% \|±11/2> +<br>17.1% \|±7/2> +<br>14.9% \|±13/2> | 989 | 0.0<br>0.0<br>19.9 | 24.3% \|±7/2> +<br>22.1% \|±9/2> +<br>18.5% \|±5/2> +<br>13.3% \|±11/2> +<br>10.5% \|±3/2> |

**Table S32.** Computed energy levels (the ground state is set at zero), composition of the *g*-tensor ($g_x$, $g_y$, $g_z$) of the ground state and the main components (>10%) of the wavefunction for each $m_J$ state of the ground-state multiplet $^5I_8$ for all structures of **3**.

| | **150K** | | **300K** | |
|---|---|---|---|---|
| **State** | **Energy (cm$^{-1}$)** | **Wavefunction** | **Energy (cm$^{-1}$)** | **Wavefunction** |
| 1 | 0 | 65.1% \|±8> + 14.0% \|±6> | 0 | 55.9% \|±8> + 13.3% \|±6> + 11.7% \|±7> |
| 2 | 1 | 67.1% \|±8> + 12.8% \|±6> | 2 | 58.5% \|±8> + 13.9% \|±6> + 11.8% \|±7> |
| 3 | 23 | 34.4% \|±2> + 26.2% \|±1> + 13.8% \|±4> + 13.0% \|±3> | 44 | 38.3% \|±2> + 17.2% \|±0> + 14.0% \|±5> |
| 4 | 24 | 30.9% \|±3> + 22.6% \|±2> + 14.7% \|±1> + 12.1% \|±0> | 49 | 34.8% \|±1> + 31.1% \|±3> |
| 5 | 75 | 33.2% \|±5> + 21.4% \|±7> + 13.0% \|±0> + 11.1% \|±2> | 87 | 21.6% \|±5> + 19.5% \|±1> + 17.5% \|±7> + 12.5% \|±4> + 10.4% \|±2> |
| 6 | 78 | 42.4% \|±3> + 24.0% \|±7> + 12.3% \|±5> | 99 | 35.1% \|±3> + 21.1% \|±7> + 18.6% \|±2> |
| 7 | 123 | 32.0% \|±4> + 21.7% \|±5> + 12.9% \|±2> + 11.2% \|±7> | 162 | 21.0% \|±2> + 20.1% \|±6> + 14.3% \|±7> + 14.0% \|±0> + 10.6% \|±3> |
| 8 | 125 | 30.2% \|±1> + 17.7% \|±7> + 12.7% \|±4> + 11.8% \|±6> + 10.0% \|±3> | 167 | 54.0% \|±4> + 14.5% \|±3> |
| 9 | 135 | 26.3% \|±4> + 18.4% \|±1> + 17.3% \|±2> + 12.7% \|±0> | 180 | 27.6% \|±1> + 19.0% \|±0> + 14.9% \|±8> + 12.2% \|±5> |
| 10 | 136 | 27.6% \|±1> + 18.3% \|±0> + 10.4% \|±4> | 180 | 29.3% \|±1> + 13.6% \|±0> + 13.5% \|±7> + 12.0% \|±4> + 11.6% \|±8> + 10.7% \|±2> |
| 11 | 173 | 18.2% \|±1> + 17.0% \|±2> + 15.9% \|±6> + 14.1% \|±4> + 12.2% \|±5> | 230 | 19.3% \|±6> + 18.7% \|±5> + 18.7% \|±1> + 14.3% \|±4> + 10.1% \|±3> |

| | | | | |
|---|---|---|---|---|
| 12 | 183 | 25.6% \|±6> +<br>20.9% \|±5> +<br>11.6% \|±7> +<br>11.4% \|±4> +<br>10.8% \|±3> | 241 | 18.9% \|±5> +<br>18.8% \|±4> +<br>18.2% \|±6> +<br>15.7% \|±1> +<br>12.0% \|±3> |
| 13 | 198 | 25.7% \|±5> +<br>25.2% \|±6> +<br>15.7% \|±3> | 264 | 30.6% \|±5> +<br>21.8% \|±6> +<br>15.9% \|±2> +<br>11.4% \|±3> |
| 14 | 269 | 21.6% \|±2> +<br>19.2% \|±7> +<br>16.1% \|±3> +<br>10.9% \|±6> | 357 | 22.2% \|±2> +<br>19.4% \|±7> +<br>15.4% \|±3> +<br>10.7% \|±6> +<br>10.5% \|±8> +<br>10.2% \|±0> |
| 15 | 269 | 18.9% \|±7> +<br>18.5% \|±1> +<br>17.7% \|±3> +<br>17.2% \|±2> +<br>11.5% \|±6> | 357 | 21.3% \|±1> +<br>19.0% \|±7> +<br>18.1% \|±3> +<br>13.7% \|±2> +<br>11.3% \|±6> +<br>10.6% \|±8> |
| 16 | 397 | 23.5% \|±5> +<br>22.1% \|±6> +<br>18.7% \|±4> +<br>13.3% \|±7> +<br>11.1% \|±3> | 475 | 24.4% \|±5> +<br>22.3% \|±6> +<br>18.4% \|±4> +<br>13.1% \|±7> +<br>11.0% \|±3> |
| 17 | 397 | 23.7% \|±5> +<br>22.0% \|±6> +<br>18.5% \|±4> +<br>13.3% \|±7> +<br>11.4% \|±3> | 475 | 24.1% \|±5> +<br>22.5% \|±6> +<br>18.9% \|±4> +<br>13.0% \|±7> +<br>10.7% \|±3> |
| | **g-tensor of the ground state** | | | |
| $g_x$ | 0.0 | | 0.0 | |
| $g_y$ | 0.0 | | 0.0 | |
| $g_z$ | 17.1 | | 16.5 | |

**Table S33.** Computed energy levels (the ground state is set at zero), composition of the *g*-tensor ($g_x$, $g_y$, $g_z$) and the main components (>10%) of the wavefunction for each $m_J$ state of the ground-state multiplet $^4I_{15/2}$ for all structures of **4**.

| | **150K** | | | **300K** | | |
|---|---|---|---|---|---|---|
| **KD** | **Energy ($cm^{-1}$)** | **g** | **Wavefunction** | **Energy ($cm^{-1}$)** | **g** | **Wavefunction** |
| 1 | 0 | 0.0<br>0.0<br>17.9 | 99.9% \|±15/2> | 0 | 0.0<br>0.0<br>17.9 | 99.9% \|±15/2> |
| 2 | 170 | 0.0<br>0.0<br>15.4 | 98.1% \|±13/2> | 160 | 0.0<br>0.0<br>15.5 | 98.0% \|±13/2> |
| 3 | 251 | 1.1<br>4.7<br>13.9 | 68.2% \|±1/2> + 19.9% \|±3/2> | 255 | 1.0<br>4.6<br>13.9 | 76.1% \|±1/2> + 15.2% \|±3/2> |
| 4 | 276 | 3.4<br>4.5<br>6.4 | 30.6% \|±3/2> + 26.2% \|±1/2> + 23.0% \|±11/2> + 11.4% \|±7/2> | 291 | 2.6<br>4.4<br>7.3 | 39.3% \|±3/2> + 21.2% \|±11/2> + 16.4% \|±1/2> + 10.8% \|±7/2> |
| 5 | 307 | 0.0<br>1.0<br>9.4 | 58.6% \|±11/2> + 33.0% \|±3/2> | 308 | 0.6<br>1.1<br>10.6 | 56.0% \|±11/2> + 25.9% \|±3/2> |
| 6 | 330 | 0.7<br>1.2<br>8.2 | 55.5% \|±5/2> + 20.9% \|±9/2> + 13.4% \|±3/2> | 348 | 0.4<br>1.1<br>9.0 | 49.6% \|±5/2> + 20.0% \|±9/2> + 14.2% \|±3/2> + 10.7% \|±11/2> |
| 7 | 395 | 0.4<br>2.1<br>12.8 | 40.0% \|±9/2> + 28.6% \|±7/2> + 26.2% \|±5/2> | 418 | 0.0<br>0.5<br>15.7 | 35.3% \|±7/2> + 30.9% \|±9/2> + 27.4% \|±5/2> |
| 8 | 403 | 0.4<br>2.4<br>13.4 | 53.6% \|±7/2> + 33.8% \|±9/2> | 449 | 0.1<br>0.1<br>16.1 | 45.2% \|±7/2> + 38.3% \|±9/2> |

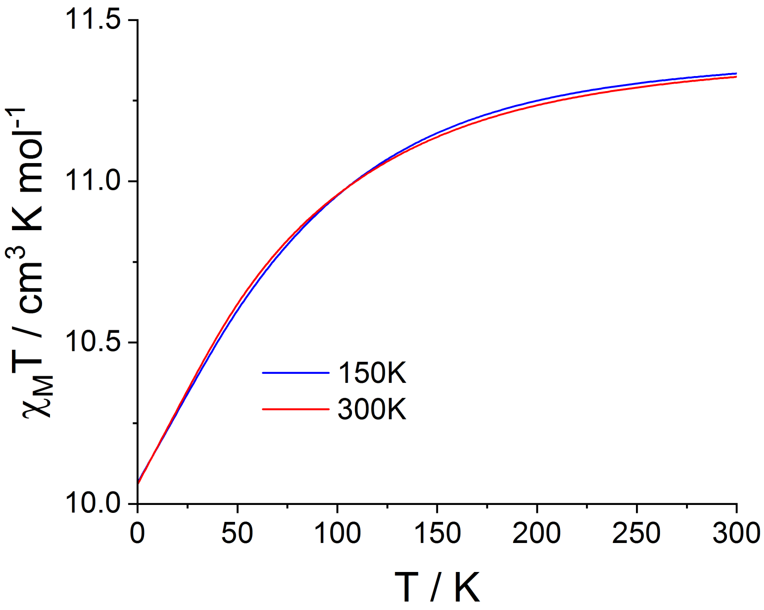


**Figure S27.** Calculated temperature variations of $\chi_M T$ for **4** from the structures computed at 150 K (blue) and 300 K (red).

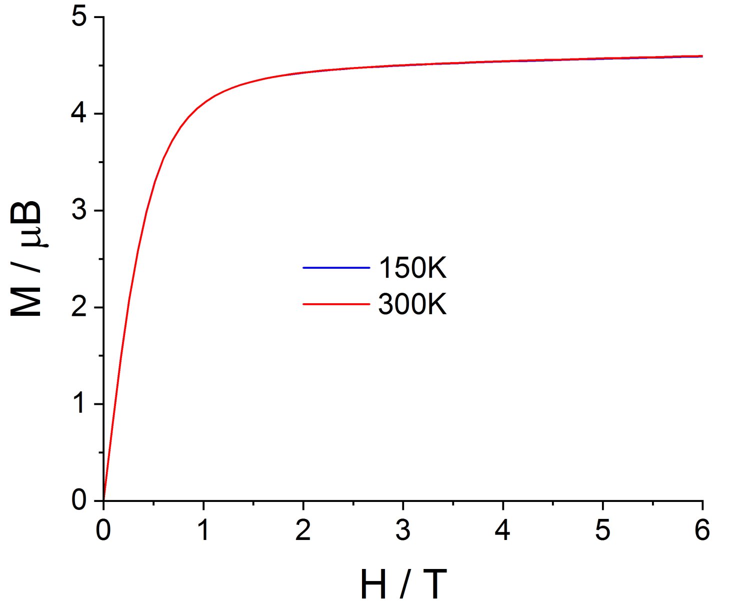


**Figure S28.** Calculated field dependence of magnetization at 2 K for **4** from the structures computed at 150 K (blue) and 300 K (red).

**Table S34.** Computed energy levels (the ground state is set at zero), composition of the *g*-tensor ($g_x$, $g_y$, $g_z$) of the ground state and the main components (>10%) of the wavefunction for each $m_J$ state of the ground-state multiplet $^3H_6$ for all structures of **5**.

| | **100K** | | **150K** | | **200K** | |
|---|---|---|---|---|---|---|
| **State** | **Energy ($cm^{-1}$)** | **Wavefunction** | **Energy ($cm^{-1}$)** | **Wavefunction** | **Energy ($cm^{-1}$)** | **Wavefunction** |
| 1 | 0 | 99.6% \|±6> | 0 | 99.5% \|±6> | 0 | 99.5% \|±6> |
| 2 | 0 | 99.6% \|±6> | 0 | 99.5% \|±6> | 0 | 99.5% \|±6> |
| 3 | 431 | 93.2% \|±5> | 443 | 91.5% \|±5> | 440 | 90.7% \|±5> |
| 4 | 432 | 93.6% \|±5> | 443 | 91.8% \|±5> | 441 | 91.2% \|±5> |
| 5 | 578 | 37.8% \|±4> + 26.5% \|±2> + 16.0% \|±3> | 585 | 30.2% \|±2> + 28.9% \|±4> + 15.8% \|±3> + 13.3% \|±0> | 579 | 27.7% \|±4> + 25.2% \|±2> + 18.1% \|±3> + 12.9% \|±1> |
| 6 | 584 | 34.0% \|±4> + 23.7% \|±3> + 18.6% \|±1> + 16.9% \|±2> | 589 | 25.5% \|±4> + 25.4% \|±1> + 22.7% \|±3> + 18.5% \|±2> | 582 | 26.5% \|±4> + 24.7% \|±2> + 19.2% \|±3> + 17.7% \|±1> |
| 7 | 636 | 52.0% \|±1> + 44.8% \|±4> | 646 | 49.4% \|±1> + 47.3% \|±4> | 646 | 51.0% \|±1> + 46.8% \|±4> |
| 8 | 648 | 41.3% \|±4> + 37.0% \|±0> + 17.0% \|±2> | 658 | 44.1% \|±4> + 36.3% \|±0> + 12.8% \|±2> | 656 | 46.6% \|±4> + 35.7% \|±0> + 11.6% \|±2> |
| 9 | 691 | 54.6% \|±3> + 17.0% \|±1> + 13.6% \|±4> + 13.5% \|±0> | 704 | 50.2% \|±3> + 19.1% \|±1> + 18.2% \|±4> | 700 | 50.1% \|±3> + 18.0% \|±1> + 16.5% \|±4> + 12.4% \|±0> |
| 10 | 718 | 38.5% \|±2> + 23.0% \|±1> + 20.5% \|±3> + 17.3% \|±4> | 737 | 45.7% \|±2> + 21.1% \|±4> + 16.7% \|±1> + 12.6% \|±3> | 735 | 43.1% \|±2> + 21.5% \|±4> + 19.6% \|±1> + 14.3% \|±3> |
| 11 | 734 | 51.6% \|±3> + 44.5% \|±2> | 752 | 56.6% \|±3> + 30.8% \|±2> + 10.5% \|±1> | 751 | 58.2% \|±3> + 34.1% \|±2> |
| 12 | 767 | 77.9% \|±1> + 15.2% \|±3> | 790 | 68.2% \|±1> + 17.0% \|±3> + 10.2% \|±2> | 792 | 70.0% \|±1> + 14.5% \|±3> + 10.2% \|±2> |
| 13 | 770 | 51.0% \|±2> + 37.9% \|±0> + 10.0% \|±3> | 792 | 47.8% \|±2> + 34.2% \|±0> + 14.1% \|±3> | 795 | 47.2% \|±2> + 34.3% \|±0> + 14.4% \|±3> |
| | **g-tensor of the ground state** | | | | | |
| $g_x$ | 0.0 | | 0.0 | | 0.0 | |
| $g_y$ | 0.0 | | 0.0 | | 0.0 | |
| $g_z$ | 14.0 | | 14.0 | | 14.0 | |

| | 250K | | 300K | |
|---|---|---|---|---|
| **State** | **Energy ($cm^{-1}$)** | **Wavefunction** | **Energy ($cm^{-1}$)** | **Wavefunction** |
| 1 | 0 | 99.2% \|±6> | 0 | 99.3% \|±6> |
| 2 | 0 | 99.2% \|±6> | 0 | 99.3% \|±6> |
| 3 | 435 | 89.1% \|±5> | 458 | 86.7% \|±5> |
| 4 | 436 | 89.4% \|±5> | 458 | 87.0% \|±5> |
| 5 | 561 | 33.6% \|±2> + 18.9% \|±4> + 18.4% \|±0> + 11.1% \|±3> + 11.0% \|±1> | 591 | 33.3% \|±2> + 20.6% \|±4> + 16.0% \|±0> + 13.0% \|±3> |
| 6 | 564 | 33.8% \|±1> + 19.4% \|±2> + 17.1% \|±4> + 16.8% \|±3> | 594 | 29.2% \|±1> + 19.7% \|±2> + 19.3% \|±4> + 18.8% \|±3> |
| 7 | 630 | 51.1% \|±4> + 40.4% \|±1> | 664 | 46.8% \|±1> + 46.3% \|±4> |
| 8 | 646 | 52.0% \|±4> + 32.6% \|±0> | 681 | 46.2% \|±4> + 34.9% \|±0> |
| 9 | 688 | 48.9% \|±3> + 22.4% \|±1> + 19.9% \|±4> | 726 | 47.9% \|±3> + 22.7% \|±4> + 20.1% \|±1> |
| 10 | 728 | 51.5% \|±2> + 26.0% \|±4> + 11.9% \|±1> | 767 | 51.5% \|±2> + 26.6% \|±4> + 12.4% \|±1> |
| 11 | 742 | 64.5% \|±3> + 21.5% \|±2> + 10.5% \|±1> | 781 | 65.1% \|±3> + 20.7% \|±2> + 10.6% \|±1> |
| 12 | 792 | 58.7% \|±1> + 18.0% \|±3> + 15.3% \|±2> | 840 | 58.3% \|±1> + 17.2% \|±2> + 15.1% \|±3> |
| 13 | 794 | 44.1% \|±2> + 29.8% \|±0> + 15.2% \|±3> | 841 | 41.0% \|±2> + 30.4% \|±0> + 15.4% \|±3> + 11.4% \|±1> |
| | **g-tensor of the ground state** | | | |
| $g_x$ | 0.0 | | 0.0 | |
| $g_y$ | 0.0 | | 0.0 | |
| $g_z$ | 13.9 | | 13.9 | |

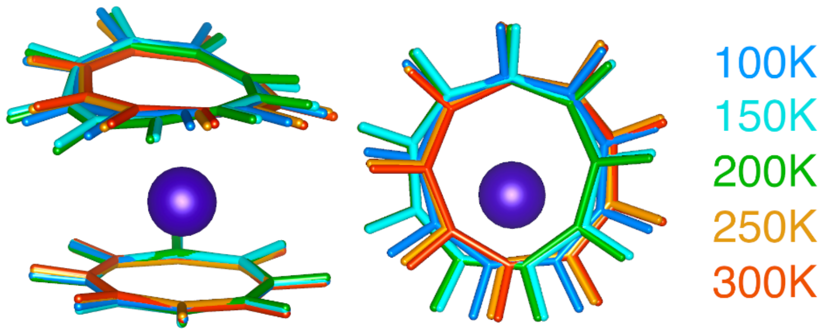


**Figure S29.** Computed structures of **5** at different temperatures.

## 5. References


[1] J. D. Hilgar, M. G. Bernbeck, B. S. Flores, J. D. Rinehart, *Chemical Science* **2018**, *9*, 7204-7209.

[2] S. M. Cendrowski-Guillaume, M. Nierlich, M. Lance, M. Ephritikhine, *Organometallics* **1998**, *17*, 786-788.

[3] S. M. Cendrowski-Guillaume, G. Le Gland, M. Nierlich, M. Ephritikhine, *Organometallics* **2000**, *19*, 5654-5660.

[4] I. L. Fedushkin, M. N. Bochkarev, S. Dechert, H. Schumann, *Chemistry-a European Journal* **2001**, *7*, 3558-3563.

[5] G. Sheldrick, *Acta Crystallogr., Sect. A* **2008**, *64*, 112-122.

[6] G. Sheldrick, *Acta Crystallographica Section A* **2015**, *71*, 3-8.

[7] G. Sheldrick, *Acta Crystallographica Section C* **2015**, *71*, 3-8.

[8] A. Spek, *Journal of Applied Crystallography* **2003**, *36*, 7-13.

[9] O. V. Dolomanov, L. J. Bourhis, R. J. Gildea, J. A. K. Howard, H. Puschmann, *J. Appl. Cryst.* **2009**, *42*, 339-341.

[10] C. F. Macrae, I. J. Bruno, J. A. Chisholm, P. R. Edgington, P. McCabe, E. Pidcock, L. Rodriguez-Monge, R. Taylor, J. Van De Streek, P. A. Wood, *Journal of Applied Crystallography* **2008**, *41*, 466-470.

[11] L. Farrugia, *Journal of Applied Crystallography* **1997**, *30*, 565.

[12] M. Xémard, S. Zimmer, M. Cordier, V. Goudy, L. Ricard, C. Clavaguéra, G. Nocton, *Journal of the American Chemical Society* **2018**, *140*, 14433-14439.

[13] G. Boche, D. Martens, W. Danzer, *Angewandte Chemie International Edition in English* **1969**, *8*, 984-984.

[14] G. Boche, H. Weber, D. Martens, A. Bieberbach, *Chemische Berichte* **1978**, *111*, 2480-2496.

[15] B. Gernot, B. Andreas, *Chemische Berichte* **1978**, *111*, 2850-2858.

[16]. I. F. Galván, M. Vacher, A. Alavi, C. Angeli, F. Aquilante, J. Autschbach, J. J. Bao, S. I. Bokarev, N. A Bogdanov, R. K Carlson, L. F. Chibotaru, J. Creutzberg, N., Dattani, M. G. Delcey, S. S. Dong, A. Dreuw, L. Freitag,L. M. Frutos, L Gagliardi, F. Gendron, A. Giussani, L. González, G. Grell, M. Guo, C. E. Hoyer, M. Johansson, E. Källman, S. Keller, S. Knecht, G. Kovacevic, G. Li Manni, M. Lundberg, Y. Ma, S. Mai, J. P. Malhado, P. Å. Malmqvist, P. Marquetand, S. A. Mewes, J. Norell, M. Olivucci, M. Oppel, Q. M. Phung, K. Pierloot, F. Plasser, M. Reiher, A. M. Sand, I. Schapiro, P. Sharma, L.K. Sørensen, C. Stein, D.G. Truhlar, M. Ugandi, L. Ungur, A. Valentini, S. Vancoillie, V. Veryazov, P.-O Widmark, S. Wouters, J. P. Zobel. and R. Lindh OpenMolcas: From Source Code to Insight. *J. Chem Theory Comput.* 2019, **15**, 5925–5964.

[17]. B. A. Hess *Phys. Rev. A: At., Mol., Opt. Phys.* 1986, **33**, 3742.

[18]. B. O. Roos, P. R. Taylor, P. E. M. Siegbahn, *Chem. Phys.* 1980, **48**, 157-173.

[19]. P.Å. Malmqvist, B.O.Roos, B. Schimmelpfennig, *Chem. Phys. Lett.* 2002, **357**, 230-240.

[20]. P.-Å. Malmqvist, B. O. Roos *Chem. Phys. Lett.* 1989, **155**, 189–194.

[21]. B. A. Hess, C. M. Marian, U. Wahlgren, O. A. Gropen, *Chem. Phys. Lett.* 1996, **251**, 365.

[22]. L. F. Chibotaru, L. Ungur, L. *J. Chem. Phys.* 2012, **137**, 064112.

[23]. L. Ungur, W. Van der Heuvel, L. Chibotaru, L. *New J. Chem.,* 2009, **33**, 1224- 1230.

[24]. B.O. Roos, R. Lindh, P. A. Malmqvist, V. Veryazov, P. O. Widmark, *J. Phys. Chem. A.* 2004, **108**, 2851- 2858.

[25]. B. O. Roos, R. Lindh, P. Malmqvist, V. Veryazov, P. O. Widmark, A. C. Borin, *J. Phys. Chem. A.* 2008, **112**, 11431-11435.
